# RNA structure-altering mutations underlying positive selection on Spike protein reveal novel putative signatures to trace crossing host-species barriers in *Betacoronavirus*

**DOI:** 10.1080/15476286.2022.2115750

**Published:** 2022-09-14

**Authors:** Alexis Felipe Rojas-Cruz, Juan Carlos Gallego-Gómez, Clara Isabel Bermúdez-Santana

**Affiliations:** a Theoretical and Computational RNomics Group, Department of Biology, Faculty of Sciences, National University of Colombia, Bogota Colombia; b Molecular and Translational Medicine Group, Faculty of Medicine, University of Antioquia, Medellin Colombia; c Center of Excellence in Scientific Computing, National University of Colombia, Bogota Colombia

**Keywords:** *Betacoronavirus*, jumping the species barrier, molecular evolution, cross-species horizontal transmission, natural selection, secondary RNA structures, viral RNA genome

## Abstract

Similar to other RNA viruses, the emergence of *Betacoronavirus* relies on cross-species viral transmission, which requires careful health surveillance monitoring of protein-coding information as well as genome-wide analysis. Although the evolutionary jump from natural reservoirs to humans may be mainly traced-back by studying the effect that hotspot mutations have on viral proteins, it is largely unexplored if other impacts might emerge on the structured RNA genome of *Betacoronavirus*. In this survey, the protein-coding and viral genome architecture were simultaneously studied to uncover novel insights into cross-species horizontal transmission events. We analysed 1,252,952 viral genomes of SARS-CoV, MERS-CoV, and SARS-CoV-2 distributed across the world in bats, intermediate animals, and humans to build a new landscape of changes in the RNA viral genome. Phylogenetic analyses suggest that bat viruses are the most closely related to the time of most recent common ancestor of *Betacoronavirus*, and missense mutations in viral proteins, mainly in the S protein S1 subunit: SARS-CoV (G > T; A577S); MERS-CoV (C > T; S746R and C > T; N762A); and SARS-CoV-2 (A > G; D614G) appear to have driven viral diversification. We also found that codon sites under positive selection on S protein overlap with non-compensatory mutations that disrupt secondary RNA structures in the RNA genome complement. These findings provide pivotal factors that might be underlying the eventual jumping the species barrier from bats to intermediate hosts. Lastly, we discovered that nearly half of the *Betacoronavirus* genomes carry highly conserved RNA structures, and more than 90% of these RNA structures show negative selection signals, suggesting essential functions in the biology of *Betacoronavirus* that have not been investigated to date. Further research is needed on negatively selected RNA structures to scan for emerging functions like the potential of coding virus-derived small RNAs and to develop new candidate antiviral therapeutic strategies.

## Introduction

Concerning a wide range of potential pathogens that are involved in cross-species transmissions, RNA viruses are a serious concern [1]. The sudden disease outbreak in 2019 (COVID-19), caused by the novel Severe Acute Respiratory Syndrome Coronavirus 2 (SARS-CoV-2), has recently emerged as a public health priority [2,3]. SARS-CoV-2, Severe Acute Respiratory Syndrome Coronavirus (SARS-CoV), and Middle East Respiratory Syndrome Coronavirus (MERS-CoV), are members of the *Betacoronavirus* (*Beta-CoVs*) genus [4]. They carry a large (~30 kb) positive-sense, single-stranded RNA (+ ssRNA) genome capped at the 5′ end and poly-A tail. ORF1a and ORF1b are translated from genomic RNA, and the translation of ORF1b depends on ribosomal frameshifting element (FSE) at the end of ORF1a. In contrast, the remaining genome serves as a template to produce subgenomic RNAs (sgRNAs) from the 3′ end, which are subsequently capped and translated into structural and accessory proteins [5,6]. It has been proven that *Beta-CoVs* are prone to accumulate mutations, owing to poor fidelity of RNA polymerases, making these viral populations typically contain genetic variants that form a heterogeneous virus pool, named quasispecies [7,8]. This phenomenon is considered to drive cross-species transmission and contributes to a rapid adaptation over a wide range of diverse hosts.

*Beta-CoVs* are zoonotic pathogens originating from animals and may be transmitted to humans by direct contact. A growing body of phylogenetic analysis has identified bats as the evolutionary sources of SARS-CoV, MERS-CoV, and the recent SARS-CoV-2 [9–11]. In addition, the majority of these viruses depend on an intermediate animal host to invade human cells [12–14]. Although the molecular mechanisms enabling cross-species transmission are not well elucidated, it has been proven that essential proteins under selection tend to increase viral fitness, and repeated transmissions may hasten novel strain emergence [15,16]. A hallmark is traced back to the receptor-binding domain (RBD) of the spike (S) protein, where amino acid changes for SARS-CoV and SARS-CoV-2 mediate invasion of host cells by binding to angiotensin-converting enzyme 2 (ACE2) [17], whereas MERS-CoV exploits dipeptidyl peptidase-4 (DPP4) [18]. Therefore, it is suggested that this protein has been under intense evolutionary pressures, which might be implied on propagation of *Beta-CoVs*. However, there are many studies on this topic motivated by developing vaccines and therapeutic strategies to prevent further spillover, relying on molecular processes reflected on protein sequence [9,19,20]. Since *Beta-CoVs* have RNA genomes, it is interesting to explore how its genome is folded and to what extent a mutation might disturb its stability. Such insights would provide novel ideas for studying the evolution, adaptation, and cross-species barriers of *Beta-CoVs*.

RNA structures are broadly accepted as critical modulators in regulating transcription, translation, and replication in *Beta-CoVs* as well as other RNA viruses [21–24]. Despite their importance, only a handful of functionally conserved structural RNA elements have been identified across *Beta-CoVs*, mainly located in the 5′ and 3′ untranslated regions (UTR) and in the FSE [25,26]. Still, the majority of regions in the whole genome of *Beta-CoVs* have been largely unexplored [27,28]. Even though predicting conserved and non-conserved RNA structures in viral genomes is challenging, upon estimation of structures, the apparent natural simplicity of an RNA secondary structure promises to be useful in describing selection pressures acting on the interactions of paired and unpaired bases [29]. A conserved structure implies compensatory substitutions (e.g. GC → CG or AU → UA), maintaining the patterns of paired bases, which indicate negative selection. Conversely, substitution events that disrupt paired bases (e.g. GC → AU or CG → UA) lead to relaxed structure constraints, which represent a positive selection [29–31]. This selection concept is not different from synonymous and non-synonymous substitutions that occur on protein-coding sequence subsets (CDS). However, codons occur locally on sequence and the selection effect is observed downstream at protein stability level, while selection on RNA secondary structure is directly seen on the RNA viral genome itself [31]. This means that by exploiting positive and negative evolutionary information predicted on an RNA structure, we may be a step closer towards characterizing how structurally conserved RNAs have evolved in different hosts of *Beta-CoVs.*

Considering the extraordinary plasticity of *Beta-CoVs* that allows its adaptation to diverse host species prior to cross-barrier transmission to humans [32], recent efforts in genomic surveillance and therapeutical design are centred on a systematic approach to detect novel variants in human hosts. However, these approaches exclude domestic animals found closely in contact with wild reservoirs and humans. In this work, a detailed evolutionary framework to estimate selection pressures on the genomic architecture of SARS-CoV, MERS-CoV and SARS-CoV-2 was used to develop a landscape of events tracing back to cross-species horizontal transmission spillovers from an exhaustive genome-wide analysis of *Beta-CoVs* circulating in different bat species, intermediate animals, and human hosts across the globe until May 2021. These analyses provide novel insights into molecular signatures applied to surveillance systems for detecting an eventual jump of these emerging viruses in advance.

## Material and methods

### Data collection

An exhaustive meta-search of *Beta-CoVs* genome sequences was performed using the following inclusion criteria: i) complete genomes; ii) high coverage level; and iii) unique sequences in the National Center for Biotechnology Information Virus (NCBI Virus) [33], Virus Pathogen Database and Analysis Resource (ViPR) [34] and ViruSurf [35]. As a supplement, the Global Initiative on Sharing All Influenza Data (GISAID) [36] to retrieve further information of SARS-CoV-2 was exploited (May 2021). Datasets were constructed from a variety of hosts for each *Beta-CoV*, labelling sequences into three groups as follows: i) *Bat* (natural host), all sequences reported in *Chiroptera* order; ii) *Intermediate* (intermediate host), all sequences defined in *Mammalia* class; and iii) *Human* (amplifier host), which included *Homo sapiens* species.

### Information preparation and curation

We conducted a meticulous preparation and curation of the data. This process involves several stages, namely: i) all viral sequences labelled as bat, intermediate and human host species were filtered out to detect any possible ambiguous characters (W, S, K, M, Y, R, V, H, D, B, N, -, =); ii) simultaneously to the previous process, it was compared sequence by sequence for each host across different sets of data retrieved from the databases, removing those with 100% similarity and keeping the longest representative sequence; iii) then the resulting sequences were sorted and fitted to reference lengths of SARS-CoV (NC_004718), MERS-CoV (NC_019843), and SARS-CoV-2 (NC_045512), containing 29,751 bp, 30,119 bp, and 29,903 bp in length, respectively; and iv) finally, to confirm a non-redundant data set, the Cluster Database at High Identity with Tolerance (CD-HIT; v4.8.1) software was used [37]. Given the large number of SARS-CoV-2 sequences circulating in humans, a threshold >0.99 was used with CD-HIT. It is worth mentioning that this careful curation method is paramount to avoid any possible ambiguous character affecting the RNA structure analysis and prediction.

### Alignments and retrieve metadata

The host’s full-length viral sequences were aligned with the default parameters using Clustal-Omega (v1.2.4) [38]. Multiple sequence alignments (MSA) were manually visualized, analysed, annotated, and edited with Aliview (v1.27) [39]. Once the datasets were curated, we retrieved for each sequence of SARS-CoV and MERS-CoV: i) associated host scientific name, ii) GenBank accession number, iii) collection date, iv) region, v) country, vi) length, and vii) collection source. Further data for SARS-CoV-2 were retrieved as follows: viii) GISAID accession number, ix) PANGO lineage and x) corresponding clade. Information not reported in databases was sought it through literature review.

### Prediction of viral open reading frames

The characterization of putative Open Reading Frames (ORFs) was performed through a modification of Gene prediction by Open reading Frame Identification using X motifs (GOFIX) program [40] using the MSA for each host. Then, ORFs were validated using BLASTN (v2.11.0) [41] from the referenced genomes of SARS-CoV, MERS-CoV, and SARS-CoV-2.

### Single nucleotide variant identification

For all downstream analysis, each host group’s data was concatenated and re-aligned with Clustal-Omega (v1.2.4) [38], resulting into a unique dataset for SARS-CoV, MERS-CoV, and SARS-CoV-2. This analysis aimed to highlight naturally possible occurring variants in *Beta-CoVs* populations. To identify variations in viral sequences, we used the Microbial Genomics Mutation Tracker software package (MicroGMT; v1.4) [42]. This package mainly uses Minimap2 (v2.21) [43] and Bcftools (v1.13) [44] to map individual sequences against the reference of SARS-CoV (NC_004718), MERS-CoV (NC_019843), and SARS-CoV-2 (NC_045512) and provides the results in a Variant Call Format (VCF) table. In addition, the program uses the SnpEff (v5.0e) tool (http://pcingola.github.io/SnpEff/) [45] to characterize all mutations detected at both nucleotide and amino acid levels in the whole viral genome. The annotated data were imported, manipulated, and plotted using R (v4.1.0) [46]. Tidyverse package (v1.3.1) [47] was used to filter, summarize and annotate data, while ggplot2 package (v3.3.5) [48] was used to align the identified variants and visualize the types of mutations.

### Time-scaled phylogenetic analysis

Full-length nucleotide sequences from each *Beta-CoV* dataset were aligned based on codons and then translated into nucleotide alignments using a combination of Clustal-Omega (v1.2.4) [38] and TranslatorX [49]. Time-scaled phylogenies for whole viral genomes were analysed through Bayesian Inference (BI) with Markov chain Monte Carlo (MCMC) methods using Bayesian Evolutionary Analysis Sampling Trees (BEAST) (v1.10.4) [50] on the CIPRES Science Gateway (v3.3) server (https://www.phylo.org/) [51]. BEAGLE (v4.0) library to enhance the speed of probability computations was used [52]. The statistical selection for the best-fit model of nucleotide substitution was performed with jModelTest (v2.1.10) [53] and Analysis of Phylogenetics and Evolution (APE) (v5.5) [54] implemented in R, considering the Bayesian information criterion (BIC). For each *Beta-CoV* dataset, we employed the tip-dating method under a General Time-Reverse model along with gamma distributed rates across invariable sites (GTR+Г + I). We ran Bayesian phylogenetic analyses using various clock model combinations (a strict clock and an uncorrelated relaxed clock with log-normal distribution (UCLN) [55]) and coalescent tree priors (constant size). The length of MCMC chain was run for 300 million steps, and the log parameter values were sampled at every 30,000 steps. Convergence of parameters was evaluated with Tracer (v1.7.132) [56], by inspecting the Effective Sample Sizes (ESS > 200), and the degree of uncertainty in each parameter estimate was provided by the 95% of Height Posterior Density (HPD) values. Trees were summarized as maximum-clade credibility (MCC) trees using Tree annotator (v1.10.0) after discarding 10% as burn-in and then visualized in FigTree (v1.4.4).

### Inference of selective pressures on protein-coding

Selective pressure analysis was performed on the CDSs for SARS-CoV, MERS-CoV, and SARS-CoV-2 through Datamonkey Adaptive Evolution Server 2.0 (https://www.datamonkey.org/) [57]. For sites statistically significant showing a positive value of non-synonymous to synonymous substitutions dN/dS >1, diversifying (positive) selection is inferred, whereas purifying (negative) selection is inferred when dN/dS <1 and neutrality as dN/dS = 1 [58]. These codon sites were analysed with a combination of four methods: i) Single-Likelihood Ancestor Counting (SLAC) [59]; ii) Fixed Effects Likelihood (FEL) [59]; iii) Fast, Unconstrained Bayesian AppRoximation (FUBAR) [60]; and iv) Mixed Effects Model of Evolution (MEME) [61]. SLAC, FEL, and FUBAR were used to identify sites that experienced both positive and negative selection, while MEME was used to detect sites that experienced positive selection [62]. We detect codon sites with positive selection signals if a specific site is overlapped by the four methods, while those with negative selection were selected from SLAC, FEL and FUBAR. Sites with a *p-value* <0.05 (SLAC, FEL and MEME) and a Bayesian posterior probability >0.95 (FUBAR) were considered statistically significant.

### Prediction of conserved RNA structures

To analyse the genomic architecture of *Beta-CoVs*, we employed the MSA from each host, which was screened in windows with a length of 120 nucleotides sliding by 40 nucleotides using RNAz v2.1 [63]. The RNAz method uses the RNAfold algorithm via RNA Vienna package to calculate secondary structures and Minimum Free Energy (MFE) for individual sequences. In addition, RNAz estimates three measures of structure conservation: i) the MFE z-score for each sequence, ii) the average MFE z-score across all sequences, and iii) the structure conservation index (SCI) of the entire alignment. Based on these criteria, RNAz determines a classification value designated as P class (P), indicating the probability for a particular region carrying a structure. We considered RNA structures with the following parameters: i) – no-reference; ii) – both-strands (±); iii) P > 0.9; and iv) – no-shuffle. As a result, RNAz hits at loci corresponding to regions with RNA structures. Therefore, the most representative structures for each host were filtered using P > 0.98 and z < −3. Lastly, these structured regions of *Beta-CoV* genomes were exploited to assess relevant RNA structures which were common, shared, or unique across the three hosts throughout its evolutionary trajectory.

### Inference of selective pressures on RNA structure

Selective pressures on RNA structures were statistically evaluated using the SSS-test (v1.0) [31] with default parameters. Scores were retrieved, imported and manipulated with tidyverse package (v1.3.1), implemented in R (v4.1.0). The constraints on RNA structures were selected following the thresholds adopted by [31]: i) *s* ≤ 2.99: negative selection; ii) *s* ≤ 4.99: weak selection; iii) *s* ≤ 9.99: moderate selection; and iv) *s* ≥ 10.0: positive selection.

### Statistics

To test whether synonymous and missense mutations detected in viruses collected from bats overperform to other host species, we carried out a two-way Analysis of Variance (ANOVA) followed by a Tukey–Kramer test using the R environment (v4.1.0) [46]. A *p-value* lower than 0.05 (p < 0.05) was considered as statistically significant.

## Results

### SARS-CoV-2 sequences from humans appear to have highly ambiguous bases

**Figure 1.**
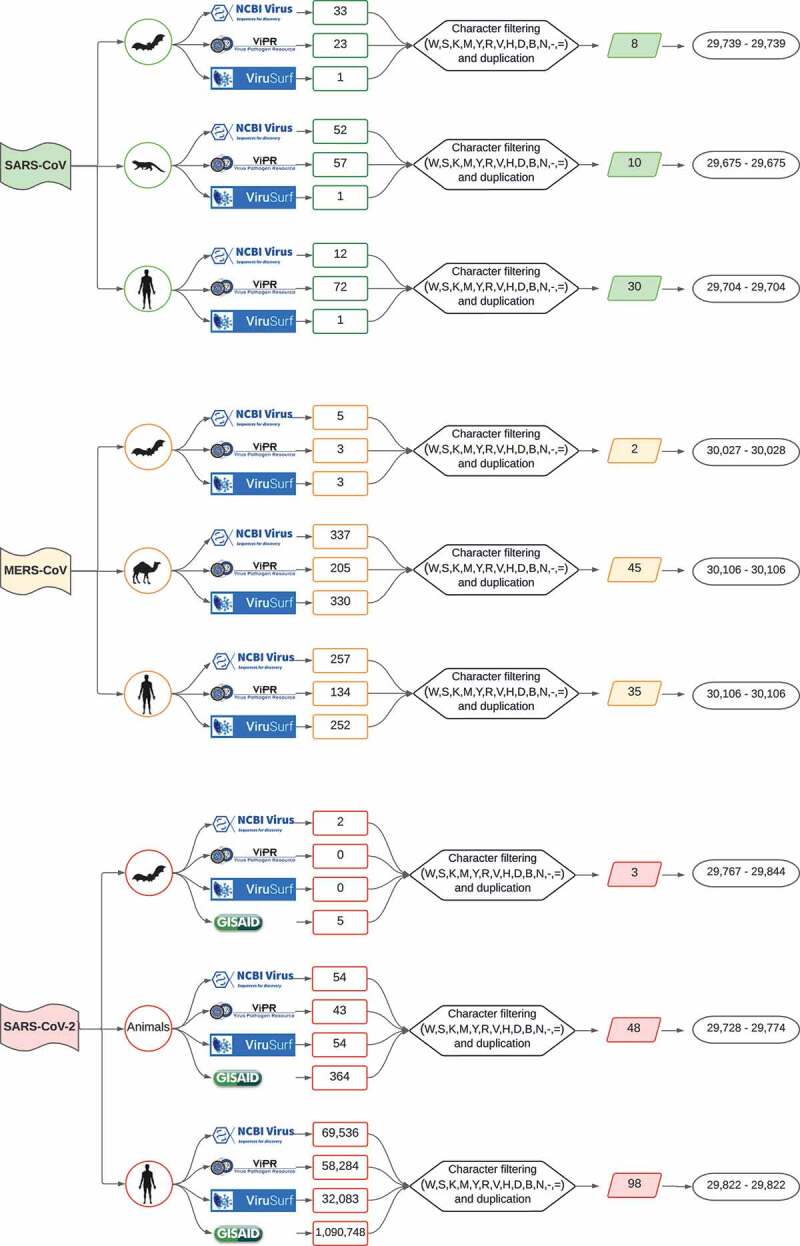
Flowchart showing approach to data collection, curation process and range of sequence lengths for the three *Beta-CoVs* analysed. First, raw viral sequences are retrieved from NCBI Virus, ViPR, ViruSurf (SARS-CoV and MERS-CoV), and further GISAID (SARS-CoV-2). Afterwards, data are labelled by host and, upon careful filtering and curation methods, the most representative viral sequences comprising each non-redundant host dataset.

A total of 1,252,952 raw genomic sequences were retrieved. Among them, 253 were from SARS-CoV, followed by 1,526 MERS-CoV and 1,251,173 SARS-CoV-2, which constituted 0.02%, 0.12% and 99.8% of all data, respectively (Supplementary Table S1). Fig. 1 provides a flowchart illustrating the workflow followed to retrieve, filter, and construct datasets for downstream analyses. Clearly, we obtained a larger number of SARS-CoV-2 sequences owing to the impact of the extensive surveillance genomic monitoring volume. Nevertheless, upon the curation of these sequences and, particularly, those isolated from humans, we detected a vast number of sequences with highly ambiguous bases, which needed to be removed given requirements for RNA structure analysis.

### ORF8 is a rapidly evolving region in SARS-CoV

Next, we predicted the ORFs for each viral genome, highlighting that non-structural (ORF1a, ORF1b) and structural proteins (S, E, M, N) are highly conserved in *Beta-CoVs* (Supplementary Fig. S1). SARS-CoV showed 15 potential ORFs bounded by start and stop codons. Interestingly, SARS-CoV sequences isolated from humans contained a 29 nucleotide deletion in the middle of ORF8, resulting in the splitting of ORF8 into two smaller ORFs, namely ORF8a and ORF8b (Supplementary Fig. S1A). Annotation of the MERS-CoV genomes identified 11 ORFs (Supplementary Fig. S1B), whereas SARS-CoV-2, 14 ORFs in the three hosts to be conserved (Supplementary Fig. S1C). Further information on each predicted ORF with the GOFIX method is provided in Supplementary Table S2.

### Synonymous and missense mutations are predominant in bat viral genomes

**Figure 2.**
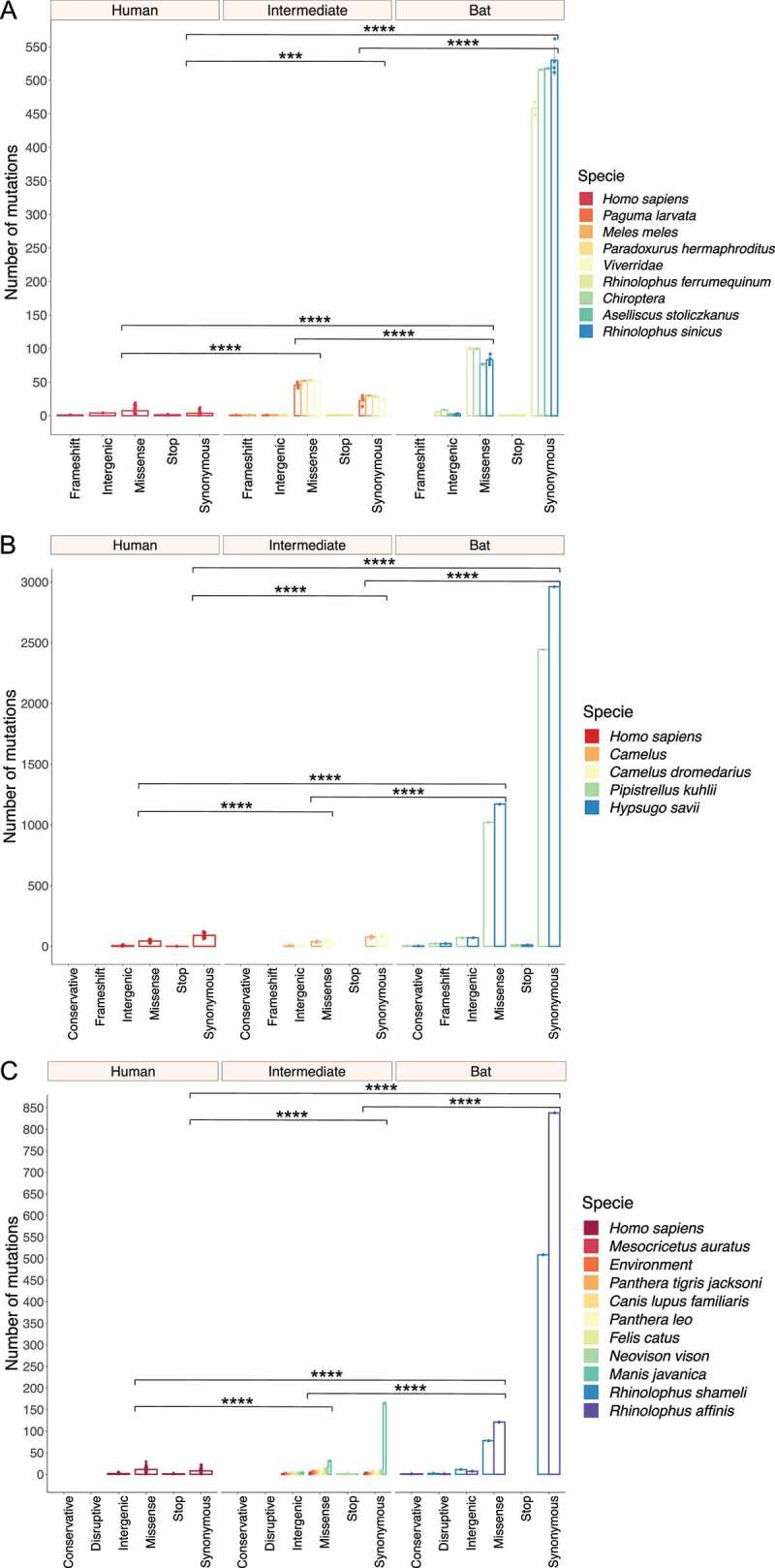
Prevalence and distribution of types of mutations found in *Beta-CoVs* circulating in diverse host species across the world. Synonymous and missense mutations occurred predominantly in bat-associated viruses. (a) A total of 5,874 mutations are detected in SARS-CoV, most of them affecting viruses found in bats, with a mean of 509 synonymous and 88.75 nonsense mutations; (b) Regarding MERS-CoV, 17,883 mutations are identified, where a mean of 2,702 are synonymous and 1,096 are missense mutations impacting the viruses collected from bats; (c) Lastly, SARS-CoV-2 registers the lowest number of mutations with 4,913, showing for viruses circulating in bats a mean of 618.6 and 92.3 are synonymous and missense mutations, respectively. Error dots denote standard errors of the mean (SEM). Statistical results represent the two-way ANOVA followed by Tukey-Kramer test. A single asterisk indicates p < 0.01 (*), a double asterisk p < 0.001 (**), a triple asterisk p < 0.0001 (***), and a fourth asterisk represents p < 0.00001 (****).

A total of 28,670 mutations were detected in the full-length viral sequences of SARS-CoV (n = 48), MERS-CoV (n = 82) and SARS-CoV-2 (n = 149). From these mutations, 5,874 (20%) were found in SARS-CoV (frameshift = 6; intergenic = 44; missense = 1,405; stop = 7; and synonymous = 4,412). For MERS-CoV 17,883 (62%) (conservative = 6; frameshift = 44; intergenic = 591; missense = 5,365; stop = 23; and synonymous = 11,854), and for SARS-CoV-2 4,913 (17%) (conservative = 1; disruptive = 5; intergenic = 250; missense = 1,691; stop = 12; and synonymous = 2,954) (Fig. 2). In terms of host, an ANOVA was conducted and determined that synonymous and missense mutations of bat viruses were statistically different compared to representative viruses infecting intermediate and human species for each *Beta-CoV* (Fig. 2). Tukey’s test showed that SARS-CoV sampled from bats had a significantly higher number of synonymous and missense mutations in comparison of those circulating in intermediate animals and humans, showing both mutations a p < 0.00001 (Fig. 2A). For MERS-CoV and SARS-CoV-2, statistical analysis also showed that viruses hosting in bats have significantly greater synonymous and missense mutations than those infecting animals and humans (p < 0.00001) (Fig. 2B and Fig. 2C).

### Hotspot mutations within the S protein S1 subunit are pivotal to cross-species transmission

**Figure 3.**
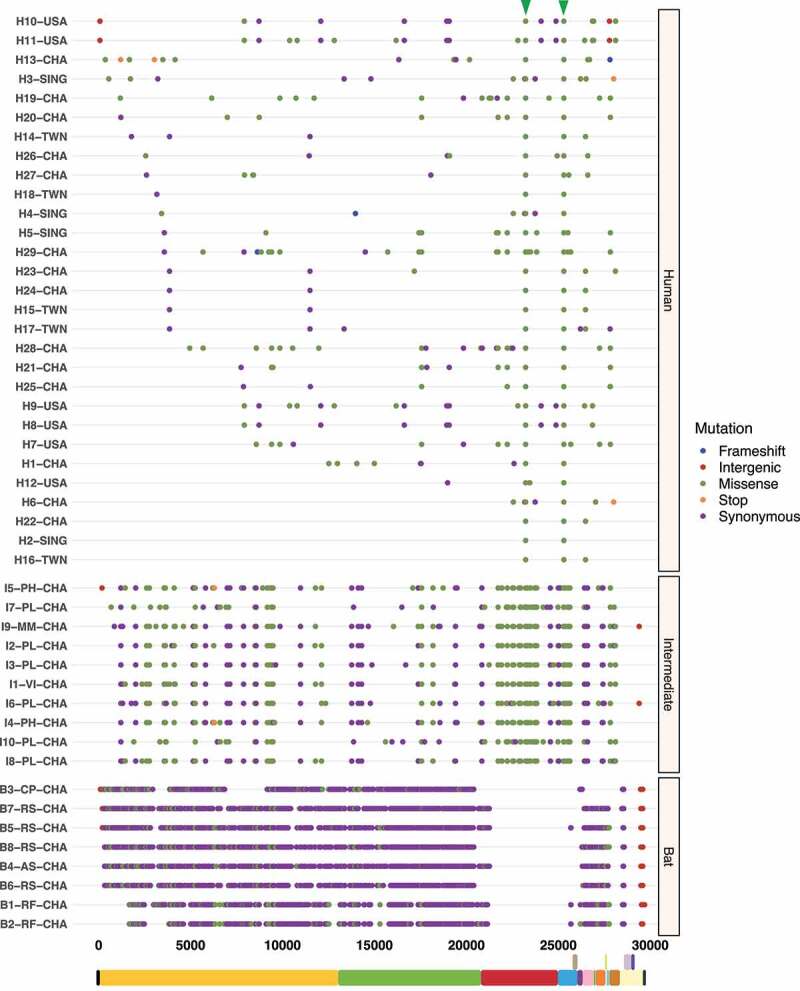
Graphical representation of hotspot mutations found in SARS-CoV genomes. The main changes are shown as a triangle at the top and concern two missense mutations in viruses circulating in intermediate animals and humans at positions: 23,220 (G > T; A577S) and 25,298 (A > G; R11G) located in S protein S1 subunit and ORF3a, respectively. Each substitution is coloured depending on mutation type (frameshift, intergenic, missense, stop, and synonymous). Viral sequences are clustered by host (human, intermediate, and bat), and genome structure is shown at the bottom.

**Figure 4.**
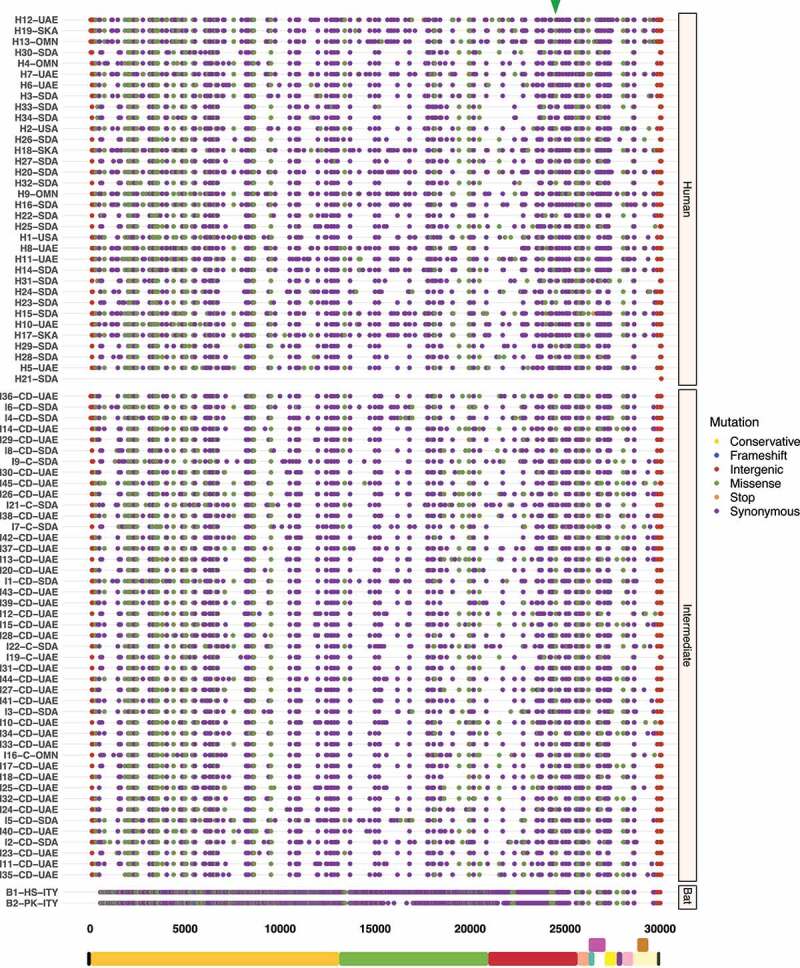
Graphical representation of hotspot mutations found in MERS-CoV genomes. While mutations show a smooth distribution, two missense mutations are highlighted with a triangle at the top corresponding to viruses found in intermediate animals and humans at positions: 23,756 (C > T; S746R) and 23,804 (C > T; N762A) within S protein S1 subunit. Each substitution is coloured depending on mutation type (conservative, frameshift, intergenic, missense, stop and, synonymous). Viral sequences are clustered by host (human, intermediate, and bat), and genome structure is shown at the bottom.

**Figure 5.**
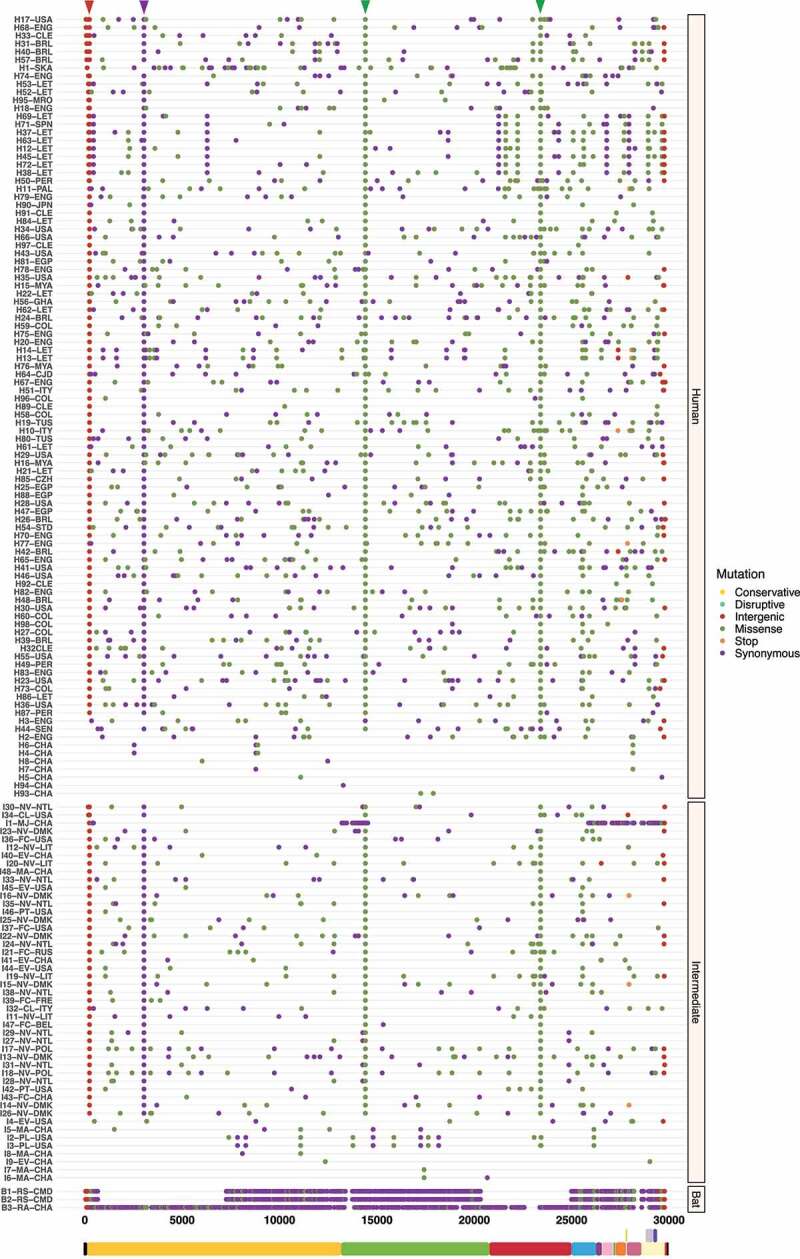
Graphical representation of hotspot mutations found in SARS-CoV-2 genomes. Unlike the other viruses, there are mutations of great interest in SARS-CoV-2 that are shared in various hosts: i) among viruses belonging to all hosts, a synonymous mutation at position 3,037 (C > T; F924F) within ORF1a is striking; while ii) those found in intermediate animals and humans, an intergenic mutation 241 (C > T) in 5’-UTR region is detected as well two missense mutations 14,408 (C > T; P4715L) and 23,403 (A > G; D614G) located in ORF1b and S protein S1 subunit, respectively. Changes are highlighted as a triangle at the top. Each substitution is coloured depending on mutation type (conservative, disruptive, intergenic, missense, stop, and synonymous). Viral sequences are clustered by host (human, intermediate, and bat), and genome structure is shown at the bottom.

To better understand the spread dynamics and the jumping of species barrier, a comparative analysis of hotspot mutations across the three hosts was performed for each viral genome. Even though different tracings were observed during the evolutionary trajectory of SARS-CoV, only two missense mutations appear to be shared between viruses found in intermediate animals and humans. These hotspot mutations were detected at positions 23,220 (G > T; A577S) and 25,298 (A > G; R11G), corresponding to S protein S1 subunit and ORF3a, respectively (Fig. 3). Regarding MERS-CoV, two missense mutations were also detected at positions 23,756 (C > T; S746R) and 23,804 (C > T; N762A) within S protein S1 subunit in viruses sampled from animals and humans (Fig. 4). Interestingly, SARS-CoV-2 showed important hotspot mutations that were reported by different hosts, namely: i) among the three host, a synonymous mutation was detected at position 3,037 (C > T; F924F) of ORF1a and ii) for viral genomes associated with intermediate and human hosts, we observed an intergenic mutation located mainly at position 241 (C > T) within 5’-UTR, and two missense mutations at positions 14,408 (C > T; P4715L) and 23,403 (A > G; D614G) within ORF1b and S protein S1 subunit, respectively (Fig. 5).

### Bats as the most plausible evolutionary sources of *Beta-CoVs*

**Figure 6.**
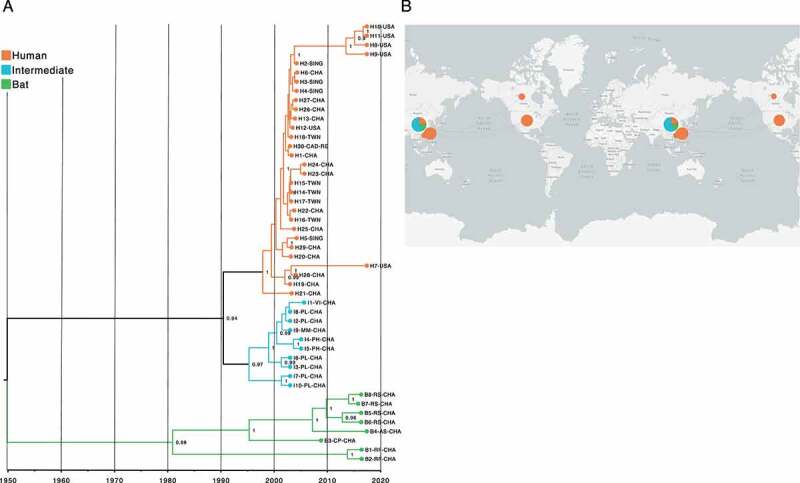
Time-scaled phylogenetic tree and spatial-dynamics of SARS-CoV. (a) Bayesian analyses was inferred from 48 SARS-CoV sequences using the tip-dating method with UCLN through BEAST. The estimated TMRCA date for SARS-CoV was at 1950-02-23 (95% HPD interval = [1898-01-14, 2002-06-21]), revealing that the most divergent are bat-associated viruses, followed by those within intermediate and human species. Each node is coded to indicate the posterior probability (PP) value. Branch lengths show divergence, and colour codes indicate host; (b) Map shows the spatial-dynamics of SARS-CoV and provides insight into the possible geographic origin for each host with sampling dates between 2003–01-01 and 2017–05-30, indicating a complex and interconnected network of viral genomes. Map was created using the data integration and visualization provided by Nexstrain using metadata related to SARS-CoV (Supplementary Table S3).

Next, we sought to trace-back the phylogenetic and epidemiological characteristics of the SARS-CoV, MERS-CoV, and SARS-CoV-2 outbreaks with time-scaled phylogenetic analysis. The best trees were inferred using the tip-dating method with UCLN. Fig. 6A reveals the possible evolutionary history of SARS-CoV with an estimated TMRCA at 1950–02-23 (95% HPD interval = [1898–01-14, 2002–06-21]), supported by a posterior probability (PP) value greater than 0.9 (PP > 0.9). At first glance, topology revealed that viruses circulating in bats were the earliest clade in the tree, and those infecting animals emerged as an early sister-clade of the human group with an estimated TMRCA at 1991–06-14 (95% HPD interval = [1967–04-12, 2015–19-03]).

**Figure 7.**
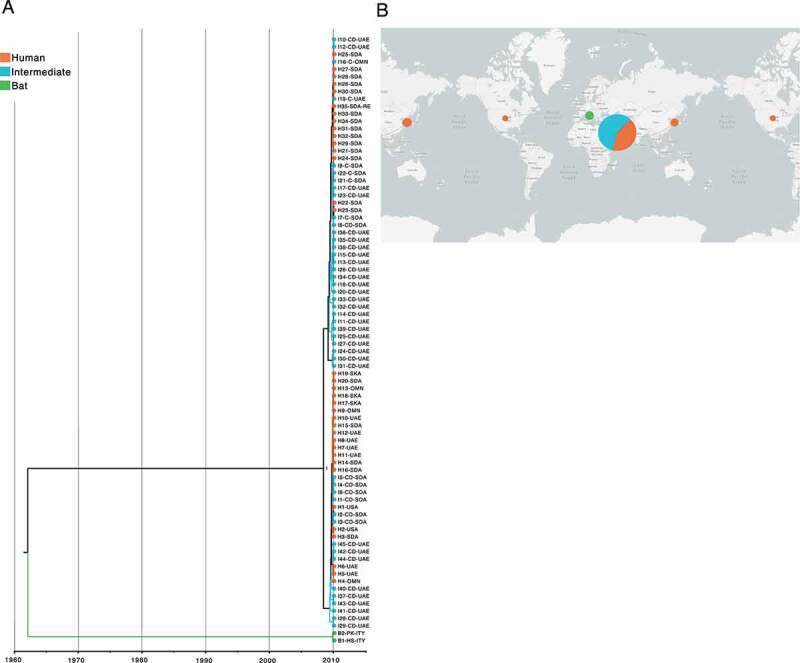
Time-scaled phylogenetic tree and spatial-dynamics of MERS-CoV. (a) Bayesian analyses was inferred from 82 MERS-CoV sequences using the tip-dating method with UCLN through BEAST. The estimated TMRCA date for MERS-CoV was at 1962–05-17 (95% HPD interval = [1913-08-22, 2011-09-27]), being the most closely related to bat viruses. Tree topology suggests that viruses isolated from intermediate and human hosts appear to be closely related. Each node is coded to indicate the posterior probability (PP) value. Branch lengths show divergence, and colour codes indicate host; (b) Map shows the spatial-dynamics of MERS-CoV and provides insight into the possible geographic origin for each host with sampling dates between 2012–06-13 and 2019–03-27, indicating a complex and interconnected network of viral genomes. Map was created using the data integration and visualization provided by Nexstrain using metadata related to MERS-CoV (Supplementary Table S3).

**Figure 8.**
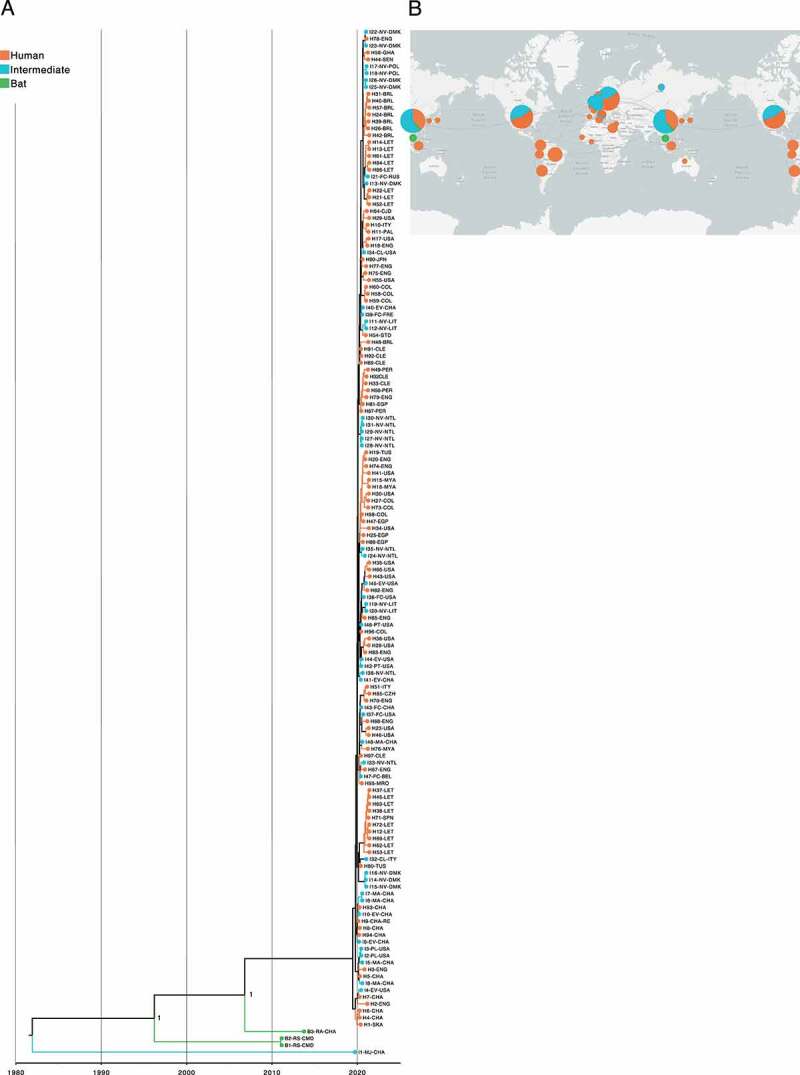
Time-scaled phylogenetic tree and spatial-dynamics of SARS-CoV-2. (a) Bayesian analyses was inferred from 149 SARS-CoV-2 sequences using the tip-dating method with UCLN through BEAST. The estimated TMRCA date for SARS-CoV-2 was at 1983–01-28 (95% HPD interval = [1961–07-13, 2005-12-09]). Tree topology reveals that virus isolated from *Manis javanica* is the most closely related to TMRCA and represents part of the basal tree with bat-associated viruses. Similar to MERS-CoV, viruses found in intermediate animals and human are highly related. Each node is coded to indicate the posterior probability (PP) value. Branch lengths show divergence, and colour codes indicate host; (b) Map analysis represents the propagation and evolution of SARS-CoV-2 genomes and provides insight into the possible geographic origin for each host with sampling dates between 2010–12-06 and 2021–04-02, indicating a complex and interconnected network of viral genomes. Map was created using the data integration and visualization provided by Nexstrain using metadata related to SARS-CoV-2 (Supplementary Table S3).

Interestingly, MERS-CoV and SARS-CoV-2 showed a topology with slightly divergent traces compared to SARS-CoV, supported by (PP > 0.9) and (PP > 1), respectively. Although bat-associated MERS-CoV was the most closely related to TMRCA, estimated at 1962–05-17 (95% HPD interval = [1913–08-22, 2011–09-27]) (Fig. 7A), it was found that SARS-CoV-2 with a TMRCA at 1983–01-28 (95% HPD interval = [1961–07-13, 2005-12-09]) was a virus isolated from *Manis javanica* as the most basal in the tree (Fig. 8A). Additionally, it is worth mentioning that the phylogenetic relationships found among viruses circulating in intermediate and human hosts of MERS-CoV and SARS-CoV-2 were pretty closely related, showing an estimated TMRCA at 2008-10-27 (95% HPD interval = [1995-12-28, 2018–07-19]) and 2019-10-04 (95% HPD interval = [2018-11-10, 2021-03-17]), respectively.

With regard to geographic distributions, the MERS-CoV map showed that viruses sampled from bats were located in Italy (Fig. 7B), whereas those belonging to SARS-CoV and SARS-CoV-2 were isolated from China (Fig. 6B and Fig. 8B). Furthermore, the majority of MERS-CoV intermediate and human hosts were from the United Arab Emirates and Saudi, rather than those associated with SARS-CoV and SARS-CoV-2 that had a more diverse geographic distribution across the globe. The tip-dating and full metadata for estimation of time-scaled phylogenies of SARS-CoV, MERS-CoV and SARS-CoV-2 are provided in Supplementary Table S3.

### Codon sites in the S protein S1 subunit are positively selected in the MERS-CoV and SARS-CoV-2 genomes

A combination of diverse algorithms based on a phylogenetic codon framework was used to detect specific sites evolving under natural selection on *Beta-CoVs* CDSs. We found evidence of progressive synonymous mutation fixation (dN/dS < 1) (i.e. negative selection) in 30 codons located within five CDSs (S:24 > ORF1a:2 > ORF1b:1 = ORF3a:1 = M:1 = N:1) of SARS-CoV. MERS-CoV registered 138 codons in seven CDSs (ORF1a:65 > S:31 > ORF1b:29 > N:7 > ORF4b:2 = ORF5:2 = M:2); and SARS-CoV-2 obtained 86 codons in the same CDSs as SARS-CoV (ORF1a:35 > S:20 > ORF1b:15 > N:10 > M:4 > ORF3a:2) (Supplementary Table S4).

**Figure 9.**
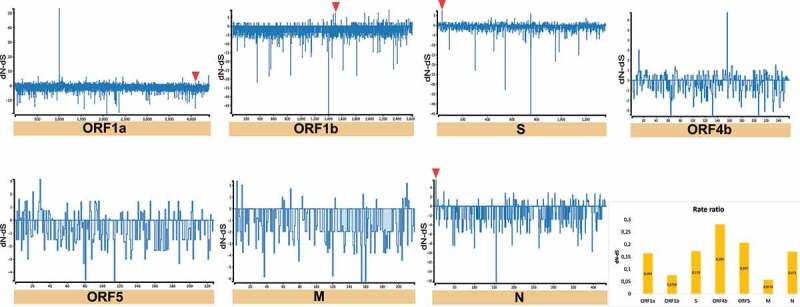
Estimation of positive and negative selection on MERS-CoV. General overview obtained by SLAC analysis, showing the evolutionary rate (dN-dS or dN/dS) at individual genes of MERS-CoV. Statistically significant codons with positive signals were inferred by overlapping of four evolutionary tests (SLAC, FEL, MEME, and FUBAR), whereas significant negative codons by (SLAC, FEL, and FUBAR). Red triangles represent codons with significant evidence for positive selection shown in Table 1.

**Figure 10.**
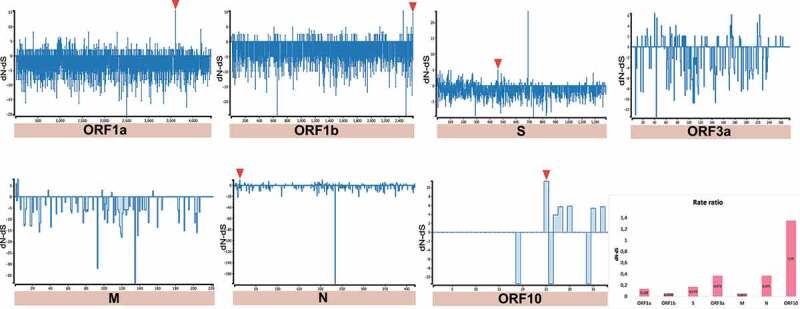
Estimation of positive and negative selection on SARS-CoV-2. General overview obtained by SLAC analysis, showing the evolutionary rate (dN-dS or dN/dS) at individual genes of SARS-CoV-2. Statistically significant codons with positive signals were inferred by overlapping of four evolutionary tests (SLAC, FEL, MEME, and FUBAR), whereas significant negative codons by (SLAC, FEL, and FUBAR). Red triangles represent codons with significant evidence for positive selection shown in Table 2.

On the other hand, we detected sites that have a higher number of synonymous mutations accumulated (dN/dS > 1) (i.e. positive selection) in MER-CoV and SARS-CoV-2 genomes. In the case of MERS-CoV, 4 codons were detected in four CDSs (ORF1a:1 = ORF1b:1 = S:1 = N:1) (Fig. 9 and Table 1) and for SARS-CoV-2, 4 of the 5 codons were also found in the same CDSs as MERS-CoV, along with the newly discovered CDS corresponding to ORF10 (ORF1a:1 = ORF1b:1 = S:1 = N:1 = ORF10:1) (Fig. 10 and Table 2). Regarding neutrality (dN/dS = 1), SARS-CoV registered the highest number of CDS under this selection (Supplementary Fig. S2), otherwise it was considerably variable among CDS for the three *Beta-CoVs*.

**Table 1.** Codons evolving at positive diversifying selection in MERS-CoV using four evolutionary tests: SLAC, FEL, MEME (*p*-value <0.05) and FUBAR (posterior probability >0.95).

Gene		SLAC		FEL		MEME		FUBAR		Inferred substitution			Amino acid
	Gene codon position	dN-dS	P[dN/dS < 1]	dN/dS	*p*-value	dN/Neutral evolution	*p-*value	dN-dS	Prob[dS< dN]	Bat	Intermediate	Human	
ORF1a	4,390	6.73	0.043	Infinity	0.009	25.09	0.02	44.23	0.997	GTG	GCA, GTA	GCA, GTA	V | A, V | A, V
ORF1b	1,503	7.01	0.021	37.05	0.028	96.1	0.0	41.70	0.992	GTC	ATC, GTC	ATC, GTC	V | I, V | I, V
S	30	7.22	0.017	Infinity	0.028	30.27	0.04	31.37	0.989	ACT	GTT, TTT	GTT, CTT, ATT, TTT	T | V, F | V, L, I, F
N	3	5.54	0.034	25.68	0.046	15.65	0.045	37.67	0.995	ACT	GCC, TCC, CCC	GCC, TCC	T | A, S, P | A, S

The criterion for considering a site positively or negatively selected was based on its identification by the four tests.

**Table 2.** Codons evolving at positive diversifying selection in SARS-CoV-2 using four evolutionary tests: SLAC, FEL, MEME (*p*-value <0.05) and FUBAR (posterior probability >0.95).

Gene		SLAC		FEL		MEME		FUBAR		Inferred substitution			Amino acid
	Gene codon position	dN-dS	P[dN/dS < 1]	dN/dS	*p-*value	dN/Neutral evolution	*p-*value	dN-dS	Prob[dS< dN]	Bat	Intermediate	Human	
ORF1a	3607	5.40	0.025	Infinity	0.01	56.19	0.0	39.71	0.999	GTG	GTG, TTG, TTT	TTG, TTT	V | V, L, F | L, F
ORF1b	2595	9.45	0.013	Infinity	0.007	120.31	0.0	30.88	0.998	AAC, TTC	AAC	AAC, CTC	N, F | N | N, L
S	461	4.97	0.0096	Infinity	0.015	55.98	0.0	39.94	0.986	TAT, CTT	CTT, CTG	CTG, ATG, CGG, CAG, CAG	Y, L | L, L | L, L, M, R, Q
N	13	9.45	0.038	Infinity	0.033	45.38	0.02	10.77	0.998	CCC	CCC	CCC, CTC, TCC	P | P | P, L, S
ORF10	25	11.43	0.025	Infinity	0.018	49.64	0.03	28.98	0.989	AAC, GAC	AGC	AAC	N, D | Y | N

The criterion for considering a site positively or negatively selected was based on its identification by the four tests.

### Nearly half of the *Beta-CoVs* genomes carry highly conserved RNA structures

The principal evidence for conserved RNA structures in *Beta-CoVs* genomes was derived from the detection of multiple loci with P > 0.98 and z < −3 in the whole genome. A total of 848 conserved loci scattered across genomes for the three *Beta-CoVs* were predicted by the RNAz approach. Among these conserved RNA structures, SARS-CoV carried 353 (42%), followed by MERS-CoV 287 (34%), and SARS-CoV-2 208 (24%) (Table 3). Additionally, we estimated the percentage of conserved RNA structures throughout the viral genome coverage by analysing the number of structured loci for each host. Following viral genome coverage, viruses belonging to intermediate and human groups were found to carry slightly more conserved loci compared to those from bats.

**Table 3.** Number of conserved loci predicted by RNAz for each host and proportion of virus genome coverage.

Virus	Host	RNAz (*p* > 0.98)	
		Number of loci	Genome coverage (%)^a^
SARS-CoV	Bat	91	36.41
	Intermediate	120	48.4
	Human	142	57.3
MERS-CoV	Bat	98	51.5
	Intermediate	94	44.72
	Human	95	44.85
SARS-CoV-2	Bat	44	22
	Intermediate	83	42.04
	Human	81	38.62

^a^Genome coverage percentage was calculated by multiplying the total number of nucleotides of all predicted loci by 100 and then dividing the viral genome length of a given host shown in Fig. 1.

### Conserved RNA structures of *Beta-CoVs* are unique for each host

**Figure 11.**
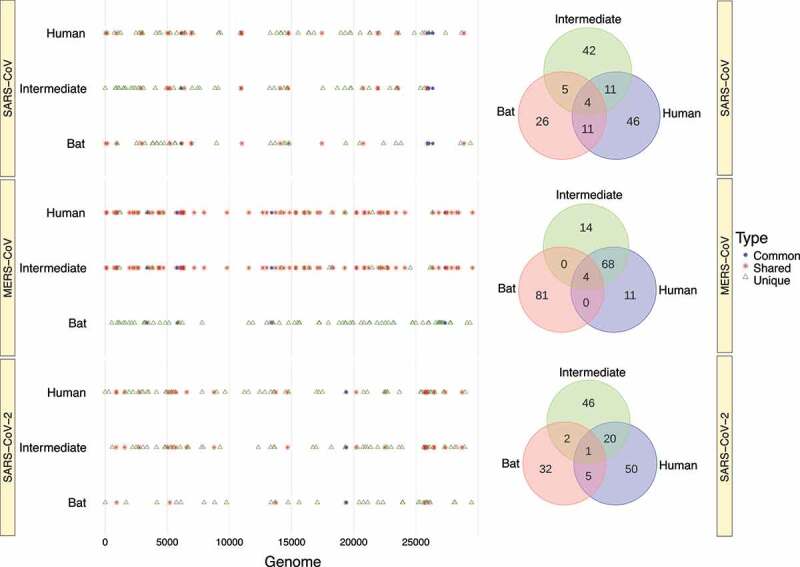
Graphical representation of regions carrying conserved RNA structures in the *Beta-CoVs* genomes. (a) The vast number of conserved RNA structures in SARS-CoV are unique across all hosts. Still, four conserved RNA structures have been common during time: ORF1a (6,121–6,240 bp); ORF3a (25,961–26,080 bp); E (26,041–26,160 bp); and M (26,361–26,480); (b) Whilst MERS-CoV circulating in bats have no shared RNA structures with those infecting intermediate animals and humans, four conserved RNA structures are found in common comprising: ORF1a (3,361–3,480; 5,801–5,920); FSE (13,401–13,520 bp); and ORF5 (27,361–27,480); and (c) SARS-CoV-2 presents a similar pattern to SARS-CoV, but exhibiting a common structure in ORF1b (19,401–19,520 bp). Venn diagrams show conserved RNA structures that are common, shared, and unique across the three hosts for each virus.

To unravel to what extent RNA structure is conserved in the same region during the passage from bats to humans, we aligned the conserved loci across the three hosts based on their genome positions. At first glance, most conserved RNA structures in *Beta-CoVs* were unique for each host (Fig. 11). Indeed, the only virus that shared a higher number of conserved RNA structures was MERS-CoV isolated from intermediate and human hosts, which showed 68 regions. However, we focused on structured regions that were common across the three hosts. For instance, we detected four conserved RNA structures in SARS-CoV that have been common during the evolutionary trajectory: ORF1a (6,121–6,240 bp); ORF3a (25,961–26,080 bp); E (26,041–26,160 bp); and M (26,361–26,480) (Fig. 11A). Similarity, for MERS-CoV, four conserved RNA structures were also found: ORF1a (3,361–3,480; 5,801–5,920); FSE (13,401–13,520 bp); and ORF5 (27,361–27,480) (Fig. 11B), whereas SARS-CoV-2, a common structure was found in ORF1b (19,401–19,520 bp) (Fig. 11C). Regions in the genome of SARS-CoV, MERS-CoV and SARS-CoV-2 with conserved RNA structures which were common, shared or unique across all three hosts are available in Supplementary Table S5.

### RNA structures in the S protein S1 subunit are positively selected in the MERS-CoV and SARS-CoV-2 genomes

**Figure 12.**
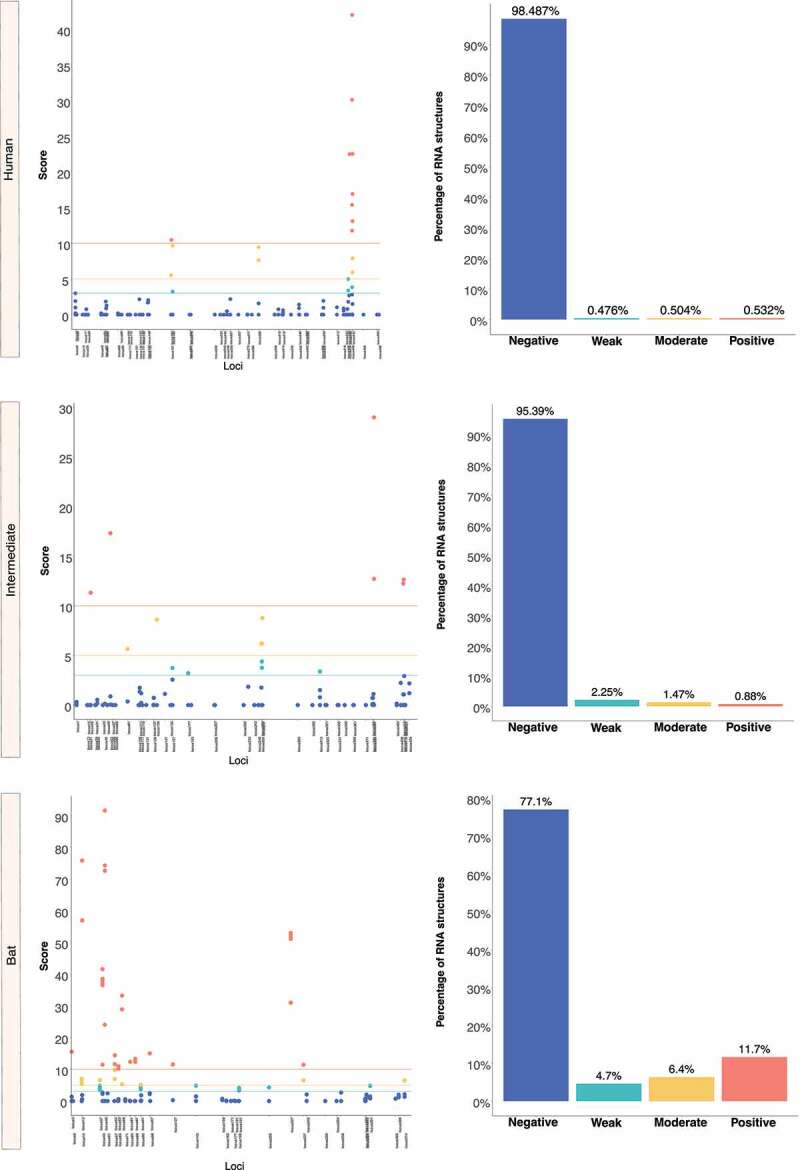
Estimation of natural selection acting on SARS-CoV RNA structures. A total of 5,102 RNA structures under selection in SARS-CoV are detected, of which 4,884 (96%) show negative selection signals. (a) In terms of each host, human-associated viruses register 3,516 (98%) RNA structures with negative selection, (b-c) while for those circulating in intermediate animals and bats, 973 (95%), and 395 (77%), respectively. The dot graph shows score obtained for RNA structure at a given loci in the genome and the bar chart represents the number of RNA structures depending on the selective restriction for each host. Global frequency of negative selection of RNA structures was calculated by multiplying the total number of RNA structures with negative selection signals across all hosts by 100, and then dividing the total number of RNA structures corresponding to all types of restrictive selection.

**Figure 13.**
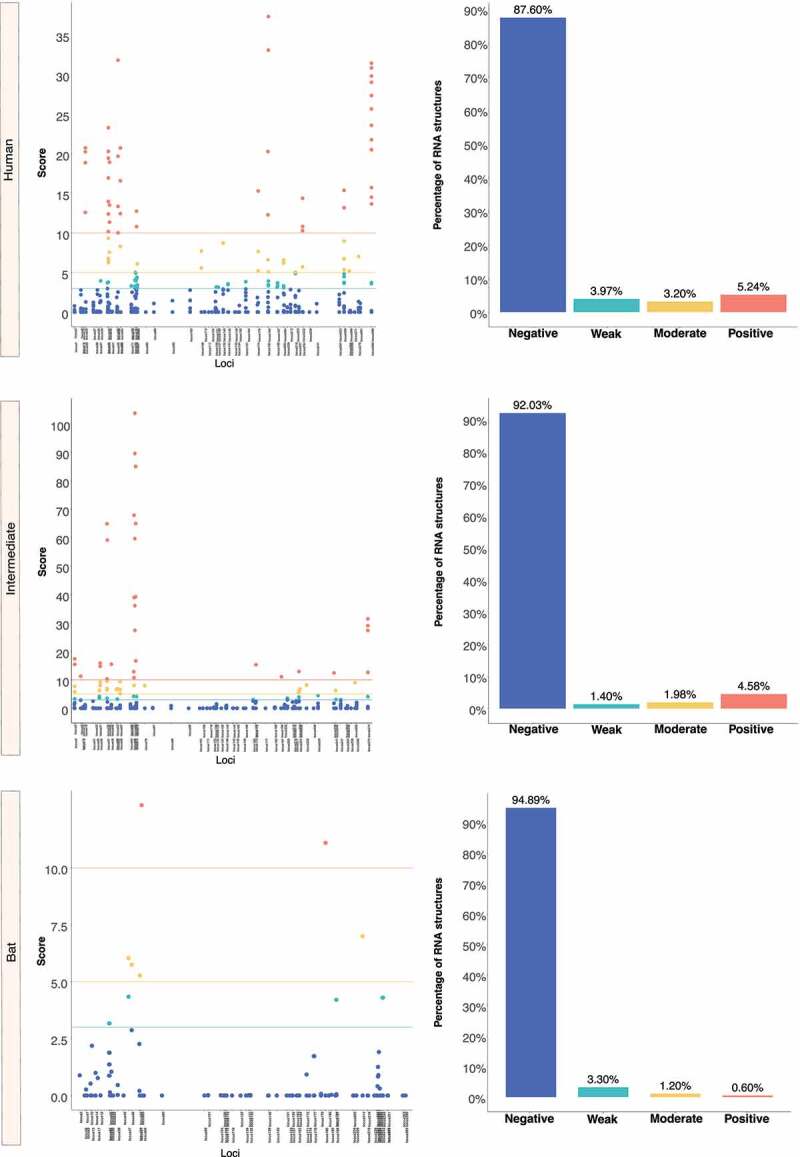
Estimation of natural selection acting on MERS-CoV RNA structures. For MERS-CoV, a total of 10,593 RNA structures under selection are identified, where 9,563 (90%) of them correspond to RNA structures with negative selection. (a) On other hand, viruses found in humans show 3,863 (88%) RNA structures with negative signals and (b-c) regarding intermediate animals and bats are 5,384 (92%), and 316 (95%), respectively. The dot graph shows score obtained for RNA structure at a given loci in the genome and the bar chart represents the number of RNA structures depending on the selective restriction for each host. Global frequency of negative selection of RNA structures was calculated by multiplying the total number of RNA structures with negative selection signals across all hosts by 100, and then dividing the total number of RNA structures corresponding to all types of restrictive selection.

**Figure 14.**
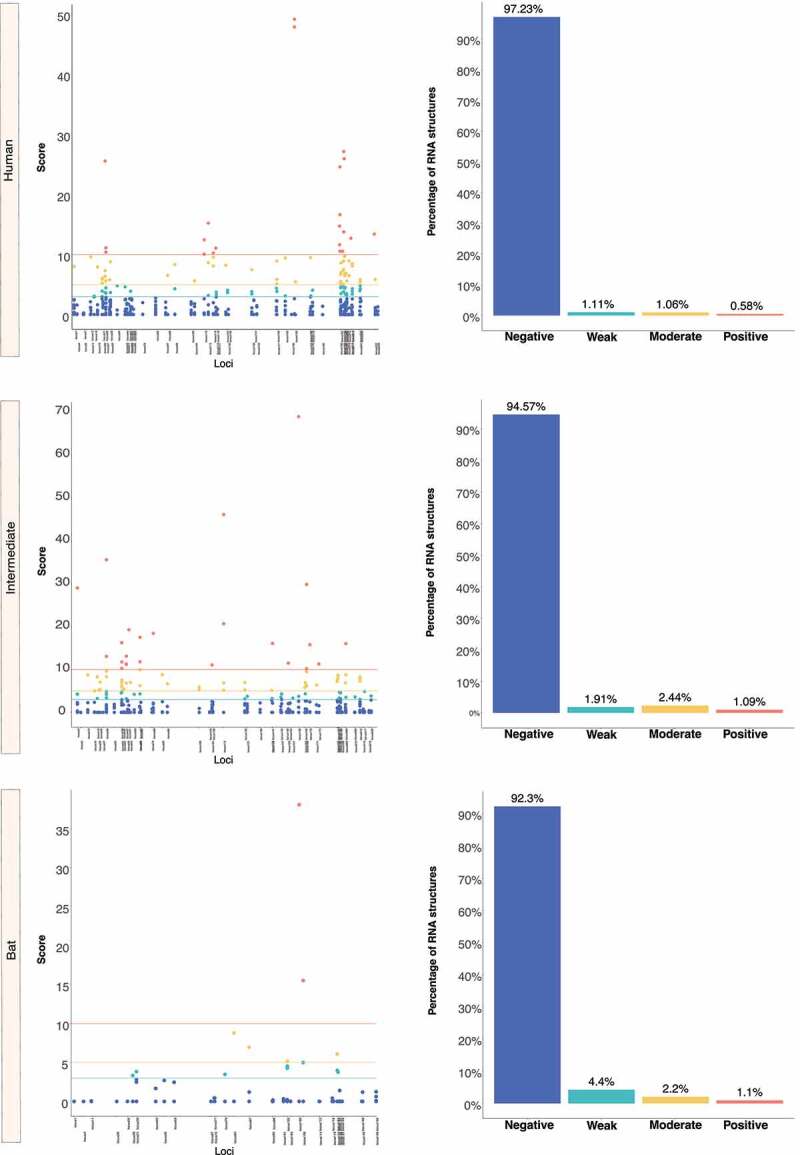
Estimation of natural selection acting on SARS-CoV-2 RNA structures. SARS-CoV-2 carries the highest number of RNA structures with natural selection 17,265. Among them, 16,629 (96%) are RNA structures showing negative signals. (a) More concretely, for viruses belonging to humans, 11,149 (97%) RNA structures are identified under negative selection, (b-c) in contrast, those infecting intermediate animals and bats show 5,311 (94%), and 169 (92%), respectively. The dot graph shows score obtained for RNA structure at a given loci in the genome and the bar chart represents the number of RNA structures depending on the selective restriction for each host. Global frequency of negative selection of RNA structures was calculated by multiplying the total number of RNA structures with negative selection signals across all hosts by 100, and then dividing the total number of RNA structures corresponding to all types of restrictive selection.

We retrieved the conserved loci for each *Beta-CoV* and assessed them for estimating natural selection with the SSS test. Surprisingly, a total of 31,076 of conserved RNA structures showed negative selection signals (s ≤ 2.99) throughout the three *Beta-CoVs*, which are crucial for the functionality of RNA molecules [29] (Fig. 12–14). Among conserved RNA structures under negative selection, 4,884 (96%) SARS-CoV were detected (Fig. 12), while MERS-CoV and SARS-CoV-2 carried a higher number, showing 9,563 (90%) (Fig. 13) and 16,629 (96%) (Fig. 14), respectively. In contrast, conserved RNA structures showing positive selection signals (*s* ≥ 10.0) were relatively low, with a total of 719, of which 88 (1.7%) were evidenced for SARS-CoV (Fig. 12), 501 (4.7%) for MERS-CoV (Fig. 13) and 130 (0.8%) for SARS-CoV-2 (Fig. 14).

**Figure 15.**
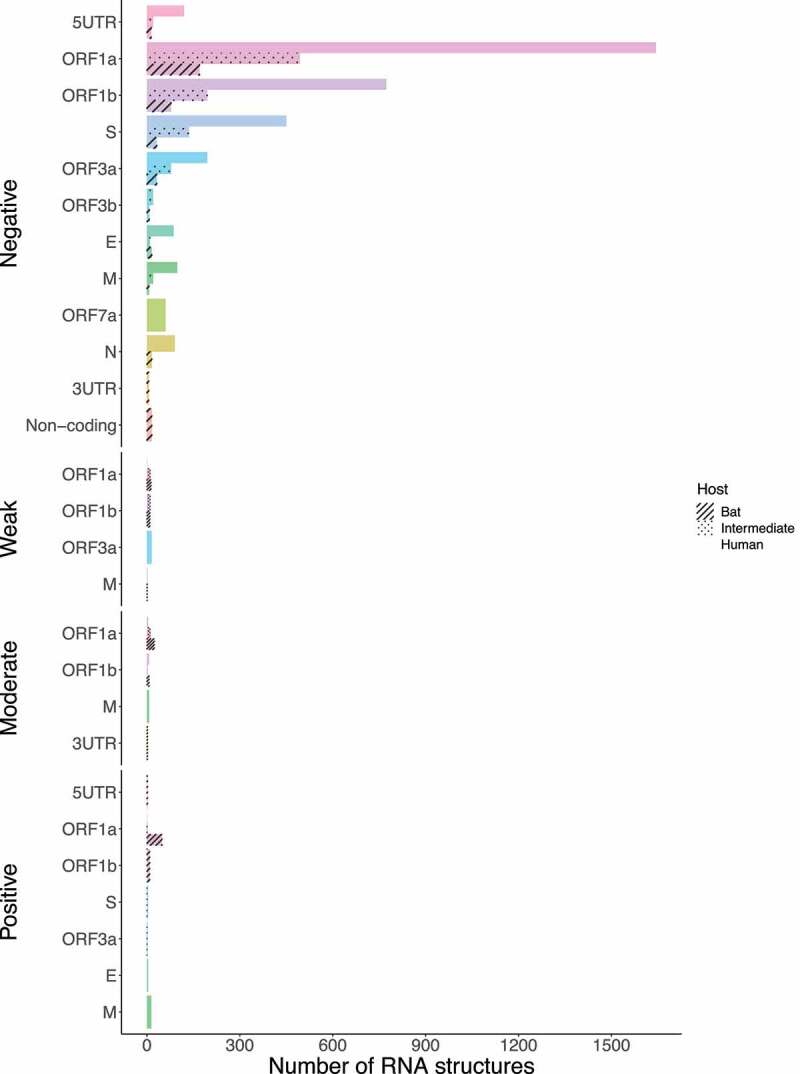
Bar chart showing frequencies of selected RNA structures across the three hosts of SARS-CoV acting on each ORF. ORF1a carries RNA structures with positive selection signals that are being disrupted from viruses circulating in bats to humans. (ORF1a: bat = 49, intermediate = 3, and human = 1). Specific number of RNA structures is available in Supplementary Table S6.

**Figure 16.**
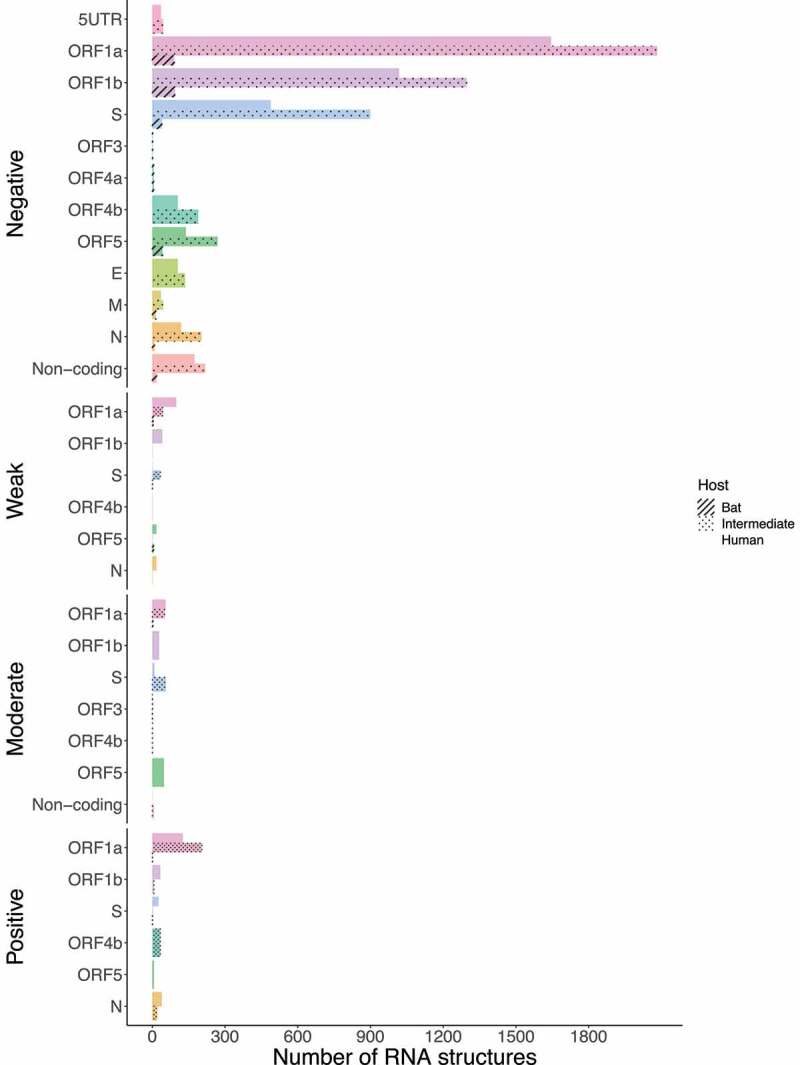
Bar chart showing frequencies of selected RNA structures across the three hosts of MERS-CoV acting on each ORF. RNA structures found in ORF1a, and S are experiencing positive selection in viruses infecting all host species (ORF1a: bat = 1, intermediate = 205, and human = 126; S: bat = 1, intermediate = 2, and human = 26). Specific number of RNA structures is available in Supplementary Table S6.

**Figure 17.**
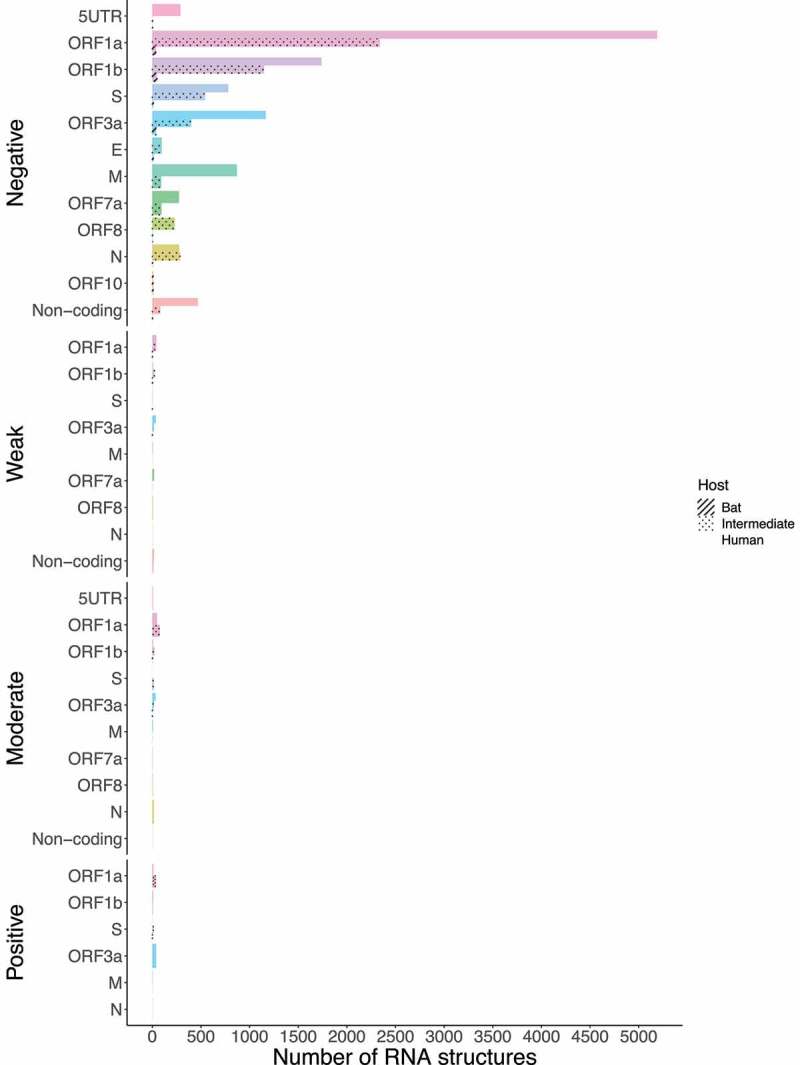
Bar chart showing frequencies of selected RNA structures across the three hosts of SARS-CoV-2 acting on each ORF. While ORF1a, ORF1b, and M contain positive RNA structures in viruses isolated from intermediate animals and humans, S shows RNA structures under positive selection for bat-to-human viral transmission (S: bat = 1, intermediate = 16, and human = 2). Specific number of RNA structures is available in Supplementary Table S6.

Next, we asked how many of these conserved RNA structures have driven the evolution of positively selected RNA structures in the S region during jumping the species barrier. Whilst SARS-CoV revealed RNA structures with positive selection on ORF1a for all hosts (bat = 49, intermediate = 3, and human = 1) (Fig. 15), MERS-CoV (bat = 1, intermediate = 2, and human = 26), and SARS-CoV-2 (bat = 1, intermediate = 16, and human = 2) were shown in the S region (Fig. 16 and Fig. 17), consistent with codon sites under positive selection in the S protein (Fig. 9 and Fig. 10).

## Discussion

Novel variants of *Beta-CoVs* are rapidly emerging, and current surveillance systems are overwhelmed, reducing the effectiveness of existing vaccines and test kits. Therefore, it is essential to scan these variations to identify where they will evolve, and which regions of the genome are most prone to mutation, useful for monitoring changes in transmissibility, virulence, and disease pathology. To cope with this, we retrieved 1,252,952 viral genomes of SARS-CoV, MERS-CoV and SARS-CoV-2 from bats and a large diversity of intermediate animals as well as from human hosts, publicly available in the most prominent virus databases, NCBI Virus [33], ViPR [34], ViruSurf [35] and GISAID [36] across the globe (May 2021). We used this information to unravel novel insights into tracing cross-species horizontal transmission in *Beta-CoVs*. First, to identify emerging variations on viral protein-coding, and second to detect if these hotspot mutations might impact the functionality of conserved structural RNA during the evolutionary process of jumping from bats to humans.

The ongoing COVID-19 pandemic was initially reported in Wuhan (China), in 2019 [64,65], though its pathogenic origin, SARS-CoV-2, remains unclear. The time-resolved tree based on UCLN points out an estimated TMRCA at 1983–01-28 (95% HPD interval = [1961–07-13, 2005-12-09]), revealing that a virus isolated from *M. javanica* is the most closely related to TMRCA, rather than those circulating in bats as has been suggested in previous reports [66–68]. Pangolins have been listed in the Convention on International Trade in Endangered Species (CITES) of Wild Fauna and Flora since its inception in 1975 through diverse Chinese wet markets [69,70], a date very close to estimated SARS-CoV-2 TMRCA, in which people already consumed Asian pangolins, and probably became infected with an ancestral pangolin virus that evidently has not been traced since 2019, when viral sequencing was undertaken in an attempt to determine the SARS-CoV-2 origin. Despite this, tree topology showed that the majority of bat-associated SARS-CoV-2 are part of the basal tree (Fig. 8A), as has been suggested for SARS-CoV and MERS-CoV, where are closely related to their TMRCA, being at 1950–02-23 (95% HPD interval = [1898–01-14, 2002–06-21]), and 1962–05-17 (95% HPD interval = [1913–08-22, 2011–09-27]), respectively (Fig. 6A and Fig. 7A) [71–73].

RaTG13 was initially considered as the closest ‘relative’ of SARS-CoV-2 [74]; a bat coronavirus detected in *Rhinolophus affinis* from Yunnan province (China) (Fig. 8B), which exhibits 96.2% genome sequence similarity to SARS-CoV-2 [74]. The fact that viruses from *M. javanica* and some bat species are highly related may suggest that SARS-CoV-2 is the result of recombination of the two viruses [16,68]. This assumption is recently gaining credibility, given a possible intermediate animal has not been identified for SARS-CoV-2 to date, as has been demonstrated for SARS-CoV and MERS-CoV, where viruses circulating in *Paguma larvata* and *Camelus dromedarius* might interact with humans, respectively [71,75]. Our SARS-CoV-2 phylogeny also fails to point out viruses infecting *M. javanica* as the primary animal acting an intermediate host for several reasons: i) phylogenetic relationships fail to cluster *M. javanica* with other animal species; ii) viruses isolated in *M. javanica* are different from SARS-CoV-2 and are, even more diverse than those found in bats, showing a closer relationship to TMRCA; iii) *M. javanica* and SARS-CoV-2 only share more than 99% sequence similarity with the RBD region [71,76]; iv) all viruses that are members of the intermediate group, including *M. javanica* possess the missense mutation (A > G; D614G), also located in the S protein S1 subunit; and v) recent evidence supports recognition of ACE2 receptors expressed in fish, amphibians, reptiles, birds and mammals [77]. More interestingly, our time-resolved trees coupled with single nucleotide variant analysis suggest that *Beta-CoVs* have been incubated for years inside bats, accumulating statistically a higher number of synonymous and missense mutations compared to representative viruses infecting intermediate and human species (p < 0.00001) (Fig. 2), leading to heterogeneous pooled viruses termed quasispecies with fitness for jumping the species barrier [62,78]. Hence, genomic variability confers an advantage to the viral population, providing a rapid adaptation to a changing environment.

Recent studies have already showed that MERS-CoV and SARS-CoV-2 are possibly under strong positive selection [79,80]. Notably, it has been suggested that amino acid changes in the S protein may considerably alter viral function and provide a route for host switching from bats to intermediate animals and humans [14]. From this concept, missense mutations detected in the S protein S1 subunit were mainly highlighted: SARS-CoV (G > T; A577S) (Fig. 3); MERS-CoV (C > T; S746R and C > T; N762A) (Fig. 4); and SARS-CoV-2 (A > G; D614G) (Fig. 5). To the best of our knowledge, this study reports that these hotspot mutations have only been appreciated in viruses circulating in intermediate animals and humans for the three *Beta-CoVs*, making them a potential evolutionary pattern to trace cross-species horizontal transmission events. Additionally, for MERS-CoV and SARS-CoV-2, recurrent positive selection was detected at codon sites on the S protein (Fig. 9 and Fig. 10) [16,62,81,82], and more suppressively, acting on the S RNA structures (Fig. 16 and Fig. 17). Our hypothesis suggests that since the S protein shows evidence of increased fixation of non-synonymous mutations (dN/dS >1), these changes may possibly disrupt base pairs in its RNA structures, hinting at a relaxation of constraints, which means positive selection [29,31]. To date, most studies have only provided a static snapshot of RNA structures in *Beta-CoVs* genomes [21,24,27,83], failing to understand how natural selection might affect the functionally of conserved RNA structures across different host in highly interesting regions as S. Therefore, this plausible scenario includes that the S protein S1 subunit of MERS-CoV and SARS-CoV-2 is both on protein-coding and structural under positive selection, providing novel insights into how some pathogenic SARS-CoV-2 variants, such as (A > G; D614G), might enable a viral fitness advantage at the RNA structure level for increased viral load, and thus have the capability to evade immune system and jump to intermediate hosts [20,84]. Considering previous evolutionary events [85], certainly, the S protein is a probable candidate driver for viral genome evolution, and possibly contributes to jump from bat viruses to intermediate animals and humans, resulting in a high zoonotic potential.

Many viruses belonging to intermediate animals with the capability to infect humans are waiting for the chance to jump the species barrier. Based on our phylogenetic analyses and previous evidence, the earliest TMRCA between intermediate animal and human viruses in *Beta-CoVs* was SARS-CoV at 1991–06-14 (95% HPD interval = [1967-04-12, 2015-19-03]), reporting an outbreak period between 2002 and 2005 [86–88] (Fig. 6A). After 17 years, the emerging TMRCA for MERS-CoV was at 2008-10-27 (95% HPD interval = [1995-12-28, 2018–07-19]) with an outbreak period ranging from 2010 to 2013 [89–92] (Fig. 7A), and the most recent SARS-CoV-2 in 2019–10-04 (95% HPD interval = [2018-, 2021–03-17]), ongoing outbreak from 2020, consistent with a bulk of time-resolved phylogenetic studies [15,93–96] (Fig. 8A). It is suggested that SARS-CoV passage from intermediate animals to humans involved a 29-nucleotide deletion in the middle of ORF8, leading to cleavage of ORF8 into two smaller ORFs found only in human viruses, namely ORF8a and ORF8b [88,97,98] (Fig. Supplementary S1A). Conversely, MERS-CoV and SARS-CoV-2 still remain unknown, but it has been supposed that hotspot mutations in the S protein lead to an increased affinity for DPP4 [99,100], and ACE2 [101–103] receptors, respectively. Considering the rapid evolution of *Beta-CoVs*, leading to changes in the sequence and structure of viral proteins, the existence of conserved RNA structures provides an opportunity to shed light on crossing from intermediate viruses to humans.

Interestingly, Fig. 11 shows that *Beta-CoV* genomes isolated from intermediate animals and humans share the most conserved RNA structures in relation to those found in bats, preserving 11, 68 and 20 regions for SARS-CoV, MERS-CoV and SARS-CoV-2, respectively. This remarkable biological peculiarity might suggest that jumping from virus circulating in an intermediate animal to human cells is probably related to how its single-stranded RNA genome folds back on itself to form intricate secondaries that have been proven essential for viral replication [23,104] and enhanced by the functionality of the S protein and its interaction with the ACE2 (SARS-CoV and SARS-CoV-2) and DPP4 (MERS-CoV) receptors. In addition, it is clearly important to account that a high-degree of evolutionary conservation of the RNA structure may represent a pivotal strategy to improve viral genome stability, given the important role of conserved RNA structures in virus life cycle, such as *cis*-acting RNA elements with structures in 5’ and 3’ UTRs and FSE [24,105,106]. Although these conserved RNA structures have been validated *in vivo* through click selective 2-hydroxyl acylation and profiling experiment (icSHAPE), nuclear magnetic resonance (NMR) and cryo–electron microscopy (cryo-EM) [26,107–110], none of the RNA structures we found in common across the viruses sampled from bats, intermediate animals and humans have been tested experimentally, except for the MERS-CoV FSE, which was the only common RNA structure predicted and validated for all three hosts (Fig. 11B). On the other hand, we discovered that nearly half of the *Beta-CoV* genomes carry highly conserved RNA structures (Table 3), and greater than 90% of these RNA structures show negative selection signals (Fig. 12–14), making them potential candidates as a model for the prediction of virus-derived small RNAs hidden in viral genomes that might contribute to modulate the transcriptional reprogramming of host upon infection.

## Conclusions

In summary, we report a significant landscape of potential signatures associated with jumping the species barrier of relevance for a molecular surveillance system using not only protein-coding information but also enriched by conserved RNA structures of *Beta-CoVs* circulating in bats, intermediate animals, and humans across the globe through a horizontal transmission approach. Our time-resolved phylogenies suggest that bat viruses are the most closely related to *Beta-CoVs* TMRCA, which have incubated for years inside bats with a high mutation rate compared to those circulating in intermediate and human hosts. This event might trigger the emergence of quasispecies groups, driving the onset of pivotal missense mutations in the S protein S1 subunit of SARS-CoV (G > T; A577S), MERS-CoV (C > T; S746R and C > T; N762A), and SARS-CoV-2 (A > G; D614G). In addition, the S protein S1 subunit is both on protein-coding and structural under positive selection, suggesting that it might mediate the entry of bat viruses into intermediate animals. Although transmission of virus from wild animals to human cells remains unclear, the existence of conserved RNA structures in viral genomes is a step towards unravelling this puzzle. We found that viruses isolated from intermediate animals and humans share more conserved RNA structures than those from bats, and greater than 90% of these RNA structures show negative selection signals, which remain largely unexplored. We encourage future studies to scan for emerging functions of viral conserved structures as potential coding of small RNAs and as targets of antiviral therapeutic strategies.

## Supplementary Material

Supplemental Material

## Data Availability

The data that support the findings of this study are openly available in Figshare at: http://doi.org/10.6084/m9.figshare.20439810, reference number 20439810.
