# Supplementary material for: RNA structure-altering mutations underlying positive selection on Spike protein reveal novel putative signatures to trace crossing host-species barriers in Betacoronavirus
Source: RNA Biol. 2022 Sep 14;19(1):1019–44. doi: 10.1080/15476286.2022.2115750 (PMC9481089; doi:10.1080/15476286.2022.2115750)
Supplement: Supplemental Material [file KRNB_A_2115750_SM9797.zip › SupplementaryMaterialVersion2.pdf]

RNA structure-altering mutations underlying positive selection on Spike protein might be signatures to trace crossing host-species barriers in *Betacoronavirus*

Alexis Felipe Rojas-Cruz [1], Juan Carlos Gallego-Gómez [2] & Clara Isabel Bermúdez-Santana [1,3]

[1] Grupo RNomica Teórica y Computacional, Department of Biology, Faculty of Sciences, National University of Colombia, Bogota, 111311, Colombia.

[2] Grupo Medicina Molecular y de Translación, Faculty of Medicine, University of Antioquia, Medellin, 050010, Colombia.

[3] Center of Excellence in Scientific Computing, National University of Colombia, Bogota, 111311, Colombia.

Supplementary Material

Figures

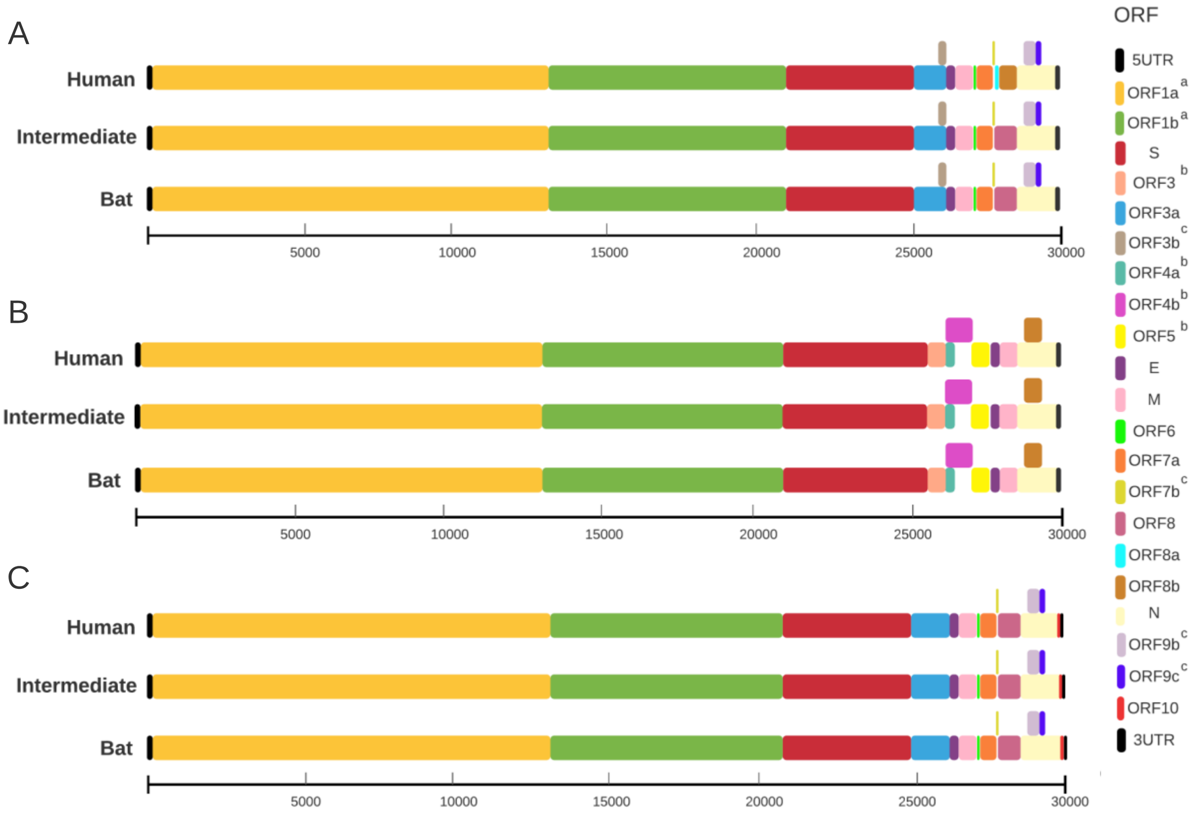

Figure S1. Prediction of representative ORFs in the *Beta-CoVs* genomes circulating in bat, intermediate and human species. Schematic representation of genomes ORFs annotated in Genbank and the putative ones predicted in this work. **(A)** SARS-CoV, bat, and intermediate hosts contain polyproteins (ORF1a and ORF1b), structural proteins (S, E, M, and N), and accessory proteins (ORF3a, ORF3b, ORF6, ORF7a, ORF7b, ORF8, ORF9b, and ORF9c), but human ORF8 is cleaved into ORF8a and ORF8b; **(B)** MERS-CoV, all hosts have polyproteins (ORF1a and ORF1b), structural proteins (S, E, M, and N) and accessory proteins (ORF3, OFR4a, ORF4b, ORF5, and ORF8b); and **(C)** SARS-CoV-2, all three host types possess polyproteins (ORF1a and ORF1b), structural proteins (S, E, M, and N) and accessory proteins (ORF3a, ORF6, ORF7a, ORF7b, ORF8, ORF9b, ORF9c and ORF10). Each ORF is annotated with a signature color as follows:

- **a.** ORF1ab is divided into two regions corresponding to the upstream (ORF1a) and downstream (ORF1b) regions of the frameshift.
- **b.** Specific ORFs for MERS-CoV genomes.
- **c.** SARS-CoV annotation for ORF3b, ORF7b, ORF9b, and ORF9c was obtained from Genbank entry AY274119: SARS-CoV isolates Tor2, where ORF9c is annotated as ORF14.

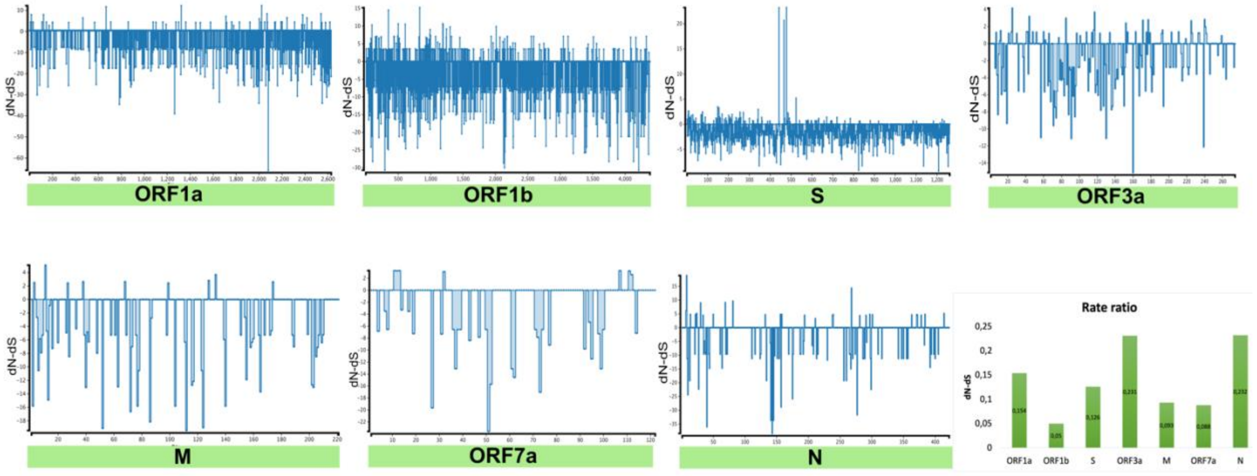

Figure S2. Estimation of negative selection on SARS-CoV. General overview obtained by SLAC analysis, showing the evolutionary rate (dN-dS or dN/dS) at individual genes of SARS-CoV. Statistically significant codons with negative signals were inferred by overlapping of three evolutionary tests (SLAC, FEL, and FUBAR).

Tables

Table S1. Detailed report of viral sequences found in diverse species analyzed in the study.

Virus	Host	Database	Specie	Sequences per specie	Raw sequences	Total raw sequences	Total
SARS-CoV	Bat	NCBI Virus	*Aselliscus stoliczkanus*	1	33	57	1252952
			Chaerephon plicatus	1			
			Chiroptera	2			
			Rhinolophus affinis	1			
			Rhinolophus ferrumequinu	7			
			Rhinolophus macrotis	1			
			Rhinolophus pusillus	3			
			Rhinolophus sinicus	16			
			Rhinolophus	1			
		ViPR	*Aselliscus stoliczkanus*	1	23		
			Bat	1			
			Rhinolophus affinis	1			
			Rhinolophus ferrumequinu	3			
			Rhinolophus monoceros	1			
			Rhinolophus pusillus	2			
			Rhinolophus sinicus	13			
			Rhinolophus	1			
		ViruSurf	*Chiroptera*	1	1		
	Intermediate	NCBI Virus	*Chlorocebus aethiops*	1	52	110	
			Mus musculus	41			
			Paguma larvata	1			
			Paradoxurus hermaphrod	6			
			Viverridae	3			
		ViPR	*Chlorocebus aethiops*	1	57		
			Badger	1			
			Mus musculus	40			
			Paguma larvata	15			
		ViruSurf	*Palm civet*	1	1		
	Human	NCBI Virus	*Homo sapiens*	12	12	86	
		ViPR	*Homo sapiens*	73	73		
		ViruSurf	*Homo sapiens*	1	1		
MERS-CoV	Bat	NCBI Virus	*Hypsugo savii*	1	5	11	
			Neoromicia capensis	2			
			Pipistrellus kuhlii	1			
			Vespertilio sinensis	1			
		ViPR	*Hypsugo savii*	1	3		
			Neoromicia capensis	1			
			Pipistrellus kuhlii	1			
		ViruSurf	*Hypsugo savii*	1	3		
			Neoromicia capensis	1			
			Pipistrellus kuhlii	1			
	Intermediate	NCBI Virus	*Camelus bactrianus*	1	337	872	
			Camelus dromedarius	258			
			Camelus	77			
			Lama glama	1			
		ViPR	*Camelus dromedarius*	200	205		
			Camelus	5			
		ViruSurf	*Camelus dromedarius*	253	330		
			Camelus	76			
			Lama glama	1			
	Human	NCBI Virus	*Homo sapiens*	257	257	643	
		ViPR	*Homo sapiens*	134	134		
		ViruSurf	*Homo sapiens*	252	252		

SARS-CoV-2	Bat	NCBI Virus	*Rhinolophus affinis*	1	2	7	
			Rhinolophus cornutus	1			
		GISAID	*Rhinolophus affinis*	1	5		
			Rhinolophus malayanus	2			
			Rhinolophus shameli	2			
	Intermediate	NCBI Virus	*Canis lupus*	2	54	515	
			Felis catus	10			
			Feliformia	1			
			Mesocricetus auratus	5			
			Mus musculus	1			
			Mustela lutreola	13			
			Neovision vision	12			
			Panthera leo	4			
			Panthera tigris	6			
		ViPR	*Canis famiaris*	1	43		
			Canino	1			
			Cat	2			
			Felis catus	4			
			Mesocricetus auratus	4			
			Mustela lutreola	13			
			Neovision vision	9			
			Panthera leo	4			
			Panthera tigris	5			
		ViruSurf	*Canis familiaris*	1	54		
			Canino	1			
			Environment	1			
			Felis catus	10			
			Feline	1			
			Mesocricetus auratus	5			
			Mustela lutreola	13			
			Neovision vision	12			
			Panthera leo	4			
			Panthera tigris	6			
		GISAID	*Canis lupus*	4	364		
			Chlorocebus sabaeus	1			
			Environment	24			
			Felis catus	16			
			Gorilla	1			
			Manis javanica	12			
			Mink	7			
			Mustela lutreola	13			
			Mus musculus	1			
			Neovision vision	275			
			Panthera leo	4			
			Panthera tigris	6			
	Human	NCBI Virus	*Homo sapiens*	69536	69536	1250651	
		ViPR	*Homo sapiens*	58284	58284		
		ViruSurf	*Homo sapiens*	32083	32083		
		GISAID	*Homo sapiens*	1090748	1090748		

Table S2. Prediction performance of the GOFIX method on the known sets of ORFs in the *Beta-CoVs* genome.

CDS annotations extracted from Genbank, with ORF names standardized according to the nomenclature for each virus

**Virus**	**ORF1a**	**ORF1b**	**S**	**ORF3**	**ORF3a**	**ORF3b**	**ORF4a**	**ORF4b**	**ORF5**	**E**	**M**	**ORF6**	**ORF7a**	**ORF7b**	**ORF8**	**ORF8a**	**ORF8b**	**N**	**ORF9b**	**ORF9c**	**ORF10**
**SARS-CoV ***	13134	7887	3768		825	465				231	666	192	369	135		129	255	1269	297	211	
**MERS-CoV ****	13176	7872	4062	312			330	741	675	249	660						339	1242			
**SARS-CoV-2 *****	13218	7788	3849		828	172				228	669	186	366	132	366			1260	294	222	117

* NCBI Reference Sequence: NC_004718.3 (https://www.ncbi.nlm.nih.gov/nuccore/NC_004718)

** NCBI Reference Sequence: NC_019843.3 (https://www.ncbi.nlm.nih.gov/nuccore/NC_019843)

*** NCBI Reference Sequence: NC_045512.2 (https://www.ncbi.nlm.nih.gov/nuccore/1798174254)

SARS-CoV: Prediction performance of the GOFIX method on the known sets of ORFs in the SARS-CoV genome

Bat			
**ORF**	**Start**	**End**	**Length**
5UTR	0	264	264
ORF1a	265	13413	13149
ORF1b	13599	21485	7887
S	21492	25217	3726
ORF3a	25227	26051	825
ORF3b	25648	26112	465
E	26076	26306	231
M	26357	27022	666
ORF6	27033	27224	192
ORF7a	27232	27600	369
ORF7b	27597	27731	135
ORF8	27800	28103	303
N	28118	29386	1269
ORF9b	28128	28424	297
ORF9c	28581	28793	213
3UTR	29387	29725	339

Intermediate			
**ORF**	**Start**	**End**	**Length**
5UTR	0	213	213
ORF1a	217	13365	13149
ORF1b	13551	21437	7887
S	21444	25211	3768
ORF3a	25220	26044	825
ORF3b	25641	26105	465
E	26069	26299	231
M	26350	27015	666
ORF6	27026	27217	192
ORF7a	27225	27593	369
ORF7b	27590	27724	135
ORF8	27778	28015	237
N	28100	29368	1269
ORF9b	28110	28406	297
ORF9c	28563	28775	213
3UTR	29369	29707	339

Human			
**ORF**	**Start**	**End**	**Length**
5UTR	0	264	264
ORF1a	265	13413	13149
ORF1b	13599	21485	7887
S	21492	25259	3768
ORF3a	25268	26092	825
ORF3b	25689	26153	465
E	26117	26347	231
M	26398	27063	666
ORF6	27074	27265	192
ORF7a	27273	27641	369
ORF7b	27638	27772	135
ORF8a	27778	27907	129
ORF8b	27863	28121	258
N	28120	29388	1269
ORF9b	28130	28426	297
ORF9c	28583	28795	213
3UTR	29389	29727	339

MERS-CoV: Prediction performance of the GOFIX method on the known sets of ORFs in the MERS-CoV genome

Bat			
**ORF**	**Start**	**End**	**Length**
5UTR	0	206	206
ORF1a	207	13376	13170
ORF1b	13565	21436	7872
S	21379	25416	4038
ORF3	25428	25739	312
ORF4a	25748	26036	288
ORF4b	25953	26714	762
ORF5	26722	27405	684
E	27484	27741	258
M	27746	28402	657
ORF8b	28643	28982	339
N	28450	29739	1290
3UTR	29740	30028	289

Intermediate			
**ORF**	**Start**	**End**	**Length**
5UTR	0		278
ORF1a	279	13454	13176
ORF1b	13643	21514	7872
S	21456	25517	4062
ORF3	25532	25843	312
ORF4a	25852	26181	330
ORF4b	26093	26833	741
ORF5	26840	27514	675
E	27590	27838	249
M	27853	28512	660
ORF8b	28762	29100	339
N	28566	29807	1242
3UTR	29808	30106	300

Human			
**ORF**	**Start**	**End**	**Length**
5UTR	0	278	278
ORF1a	279	13454	13176
ORF1b	13643	21514	7872
S	21456	25517	4062
ORF3	25532	25843	312
ORF4a	25852	26181	330
ORF4b	26093	26833	741
ORF5	26840	27514	675
E	27590	27838	249
M	27853	28512	660
ORF8b	28762	29100	339
N	28566	29807	1242
3UTR	29808	30106	299

SARS-CoV-2: Prediction performance of the GOFIX method on the known sets of ORFs in the SARS-CoV-2 genome

Bat			
**ORF**	**Start**	**End**	**Length**
5UTR	0	259	259
ORF1a	260	13456	13197
ORF1b	13741	21528	7788
S	21535	25290	3756
ORF3a	25299	26126	828
E	26151	26378	228
M	26429	27094	666
ORF6	27105	27290	186
ORF7a	27297	27662	366
ORF7b	27659	27790	132
ORF8	27869	28234	366
N	28249	29508	1260
ORF9b	28259	28552	294
ORF9c	28709	28930	222
ORF10	29533	29661	129
3UTR	29662	29844	183

Intermediate			
**ORF**	**Start**	**End**	**Length**
5UTR	0	211	211
ORF1a	212	13429	13218
ORF1b	13714	21501	7788
S	21482	25330	3849
ORF3a	25339	26166	828
E	26191	26418	228
M	26469	27137	669
ORF6	27148	27333	186
ORF7a	27340	27705	366
ORF7b	27702	27833	132
ORF8	27840	28205	366
N	28220	29479	1260
ORF9b	28230	28523	294
ORF9c	28680	28901	222
ORF10	29504	29632	129
3UTR	29633	29774	142

Human			
**ORF**	**Start**	**End**	**Length**
5UTR	0	237	237
ORF1a	238	13455	13218
ORF1b	13740	21527	7788
S	21535	25356	3849
ORF3a	25365	26192	828
E	26217	26444	228
M	26495	27163	669
ORF6	27174	27359	186
ORF7a	27366	27731	366
ORF7b	27728	27859	132
ORF8	27866	28231	366
N	28246	29505	1260
ORF9b	28256	28549	294
ORF9c	28706	28927	222
ORF10	29530	29658	129
3UTR	29659	29822	164

Table S3. Metadata retrieved from analyzed *Beta-CoVs*

SARS-CoV: Metadata

strain	specie	virus	genbank_accessio	date	region	country	region_exposure	country_exposure	source	segment	length	host
B1-RF-CHA	*Rhinolophus ferrumequinum*	SARS-CoV	KP886808	2013-05-23	Asia	China	Asia	China		genome	29739	Bat
B2-RF-CHA	*Rhinolophus ferrumequinum*	SARS-CoV	KP886809	2013-05-23	Asia	China	Asia	China		genome	29739	Bat
B3-CP-CHA	*Chiroptera*	SARS-CoV	DQ071615	2005-10-05	Asia	China	Asia	China		genome	29739	Bat
B4-AS-CHA	*Aselliscus stoliczkanus*	SARS-CoV	KY417142	2014-05-12	Asia	China	Asia	China	Fecal	genome	29739	Bat
B5-RS-CHA	*Rhinolophus sinicus*	SARS-CoV	KY417147	2013-04-17	Asia	China	Asia	China	Urine and fecal	genome	29739	Bat
B6-RS-CHA	*Rhinolophus sinicus*	SARS-CoV	KY417148	2013-04-17	Asia	China	Asia	China	Urine and fecal	genome	29739	Bat
B7-RS-CHA	*Rhinolophus sinicus*	SARS-CoV	KY417143	2012-09-18	Asia	China	Asia	China	Urine and fecal	genome	29739	Bat
B8-RS-CHA	*Rhinolophus sinicus*	SARS-CoV	KY417149	2013-04-17	Asia	China	Asia	China	Urine and fecal	genome	29739	Bat
I1-VI-CHA	*Viverridae*	SARS-CoV	AY572038	2005-08-23	Asia	China	Asia	China	Feces	genome	29675	Intermediate
I2-PL-CHA	*Paguma larvata*	SARS-CoV	AY515512	2003-01-01	Asia	China	Asia	China		genome	29675	Intermediate
I3-PL-CHA	*Paguma larvata*	SARS-CoV	AY545914	2003-01-01	Asia	China	Asia	China		genome	29675	Intermediate
I4-PH-CHA	*Paradoxurus hermaphroditus*	SARS-CoV	AY613949	2005-01-28	Asia	China	Asia	China		genome	29675	Intermediate
I5-PH-CHA	*Paradoxurus hermaphroditus*	SARS-CoV	AY613950	2005-01-28	Asia	China	Asia	China		genome	29675	Intermediate
I6-PL-CHA	*Paguma larvata*	SARS-CoV	AY545917	2003-01-01	Asia	China	Asia	China	Feces	genome	29675	Intermediate
I7-PL-CHA	*Paguma larvata*	SARS-CoV	AY545916	2003-01-01	Asia	China	Asia	China	Feces	genome	29675	Intermediate
I8-PL-CHA	*Paguma larvata*	SARS-CoV	AY545918	2003-01-01	Asia	China	Asia	China	Feces	genome	29675	Intermediate
I9-MM-CHA	*Meles meles*	SARS-CoV	AY545919	2003-01-01	Asia	China	Asia	China	Feces	genome	29675	Intermediate
I10-PL-CHA	*Paguma larvata*	SARS-CoV	AY304488	2003-01-01	Asia	China	Asia	China	Feces	genome	29675	Intermediate
H1-CHA	*Homo sapiens*	SARS-CoV	AY278554	2003-04-17	Asia	China	Asia	China	Oronasopharynx	genome	29704	Human
H2-SING	*Homo sapiens*	SARS-CoV	AY559086	2004-03-28	Asia	Singapure	Asia	Singapure	Oronasopharynx	genome	29704	Human
H3-SING	*Homo sapiens*	SARS-CoV	AY559085	2004-03-28	Asia	Singapure	Asia	Singapure	Oronasopharynx	genome	29704	Human
H4-SING	*Homo sapiens*	SARS-CoV	AY559094	2004-03-28	Asia	Singapure	Asia	Singapure	Oronasopharynx	genome	29704	Human
H5-SING	*Homo sapiens*	SARS-CoV	AY559081	2004-03-28	Asia	Singapure	Asia	Singapure	Oronasopharynx	genome	29704	Human
H6-CHA	*Homo sapiens*	SARS-CoV	AY613947	2004-04-28	Asia	China	Asia	China	Oronasopharynx	genome	29704	Human
H7-USA	*Homo sapiens*	SARS-CoV	MK062179	2017-05-27	North America	USA	North America	USA	Cell culture	genome	29704	Human
H8-USA	*Homo sapiens*	SARS-CoV	MK062180	2017-05-28	North America	USA	North America	USA	Cell culture	genome	29704	Human
H9-USA	*Homo sapiens*	SARS-CoV	MK062181	2017-05-29	North America	USA	North America	USA	Cell culture	genome	29704	Human
H10-USA	*Homo sapiens*	SARS-CoV	MK062182	2017-05-30	North America	USA	North America	USA	Cell culture	genome	29704	Human
H11-USA	*Homo sapiens*	SARS-CoV	MK062183	2017-05-30	North America	USA	North America	USA	Cell culture	genome	29704	Human
H12-USA	*Homo sapiens*	SARS-CoV	AY714217	2003-06-25	North America	USA	North America	USA	Oronasopharynx	genome	29704	Human
H13-CHA	*Homo sapiens*	SARS-CoV	AY313906	2003-12-03	Asia	China	Asia	China		genome	29704	Human
H14-TWN	*Homo sapiens*	SARS-CoV	AY502930	2003-03-15	Asia	Taiwan	Asia	Taiwan	Throat swab	genome	29704	Human
H15-TWN	*Homo sapiens*	SARS-CoV	AY502929	2003-03-15	Asia	Taiwan	Asia	Taiwan	Throat swab	genome	29704	Human
H16-TWN	*Homo sapiens*	SARS-CoV	AY502928	2003-03-15	Asia	Taiwan	Asia	Taiwan	Throat swab	genome	29704	Human
H17-TWN	*Homo sapiens*	SARS-CoV	AY502923	2003-03-15	Asia	Taiwan	Asia	Taiwan	Throat swab	genome	29704	Human
H18-TWN	*Homo sapiens*	SARS-CoV	AY291451	2003-03-15	Asia	Taiwan	Asia	Taiwan	Throat swab	genome	29704	Human
H19-CHA	*Homo sapiens*	SARS-CoV	AY310120	2003-01-01	Asia	China	Asia	China		genome	29704	Human
H20-CHA	*Homo sapiens*	SARS-CoV	AY278491	2003-03-01	Asia	Hong Kong	Asia	Hong Kong	Cell culture	genome	29704	Human
H21-CHA	*Homo sapiens*	SARS-CoV	AY278487	2003-04-21	Asia	China	Asia	China	Oronasopharynx	genome	29704	Human
H22-CHA	*Homo sapiens*	SARS-CoV	AY427439	2003-10-13	Asia	China	Asia	China	Oronasopharynx	genome	29704	Human
H23-CHA	*Homo sapiens*	SARS-CoV	AY395003	2005-09-13	Asia	China	Asia	China	Oronasopharynx	genome	29704	Human
H24-CHA	*Homo sapiens*	SARS-CoV	DQ182595	2005-09-13	Asia	China	Asia	China	Oronasopharynx	genome	29704	Human
H25-CHA	*Homo sapiens*	SARS-CoV	AY394850	2003-09-30	Asia	China	Asia	China	Oronasopharynx	genome	29704	Human
H26-CHA	*Homo sapiens*	SARS-CoV	AY395002	2004-01-29	Asia	China	Asia	China	Oronasopharynx	genome	29704	Human
H27-CHA	*Homo sapiens*	SARS-CoV	AY394991	2004-01-29	Asia	China	Asia	China	Oronasopharynx	genome	29704	Human
H28-CHA	*Homo sapiens*	SARS-CoV	AY394995	2004-01-29	Asia	China	Asia	China	Oronasopharynx	genome	29704	Human
H29-CHA	*Homo sapiens*	SARS-CoV	AY278491	2003-04-18	Asia	China	Asia	China	Oronasopharynx	genome	29704	Human
H30-CAD-RE	*Homo sapiens*	SARS-CoV	NC_004718	2003-01-01	North America	Canada	North America	Canada	Cell culture	genome	29704	Human

MERS-CoV: Metadata

strain	specie	virus	genbank_accession	date	region	country	region_exposure	country_exposure	source	segment	length	host
B1-HS-ITY	*Hypsugo savii*	MERS-CoV	MG596802	2014-07-03	Europe	Italy	Europe	Italy	Pools of viscera	genome	30027	Bat
B2-PK-ITY	*Pipistrellus kuhlii*	MERS-CoV	MG596803	2014-07-03	Europe	Italy	Europe	Italy	Pools of viscera	genome	30028	Bat
I1-CD-SDA	*Camelus dromedarius*	MERS-CoV	MN654975	2016-07-01	Asia	Saudi Arabia	Asia	Saudi Arabia	Oronasopharynx	genome	30106	Intermediate
I2-CD-SDA	*Camelus dromedarius*	MERS-CoV	MN654988	2016-07-02	Asia	Saudi Arabia	Asia	Saudi Arabia	Oronasopharynx	genome	30106	Intermediate
I3-CD-SDA	*Camelus dromedarius*	MERS-CoV	MT226600	2017-02-01	Asia	Saudi Arabia	Asia	Saudi Arabia	Oronasopharynx	genome	30106	Intermediate
I4-CD-SDA	*Camelus dromedarius*	MERS-CoV	MT226602	2017-02-01	Asia	Saudi Arabia	Asia	Saudi Arabia	Oronasopharynx	genome	30106	Intermediate
I5-CD-SDA	*Camelus dromedarius*	MERS-CoV	MT226605	2017-02-01	Asia	Saudi Arabia	Asia	Saudi Arabia	Oronasopharynx	genome	30106	Intermediate
I6-CD-SDA	*Camelus dromedarius*	MERS-CoV	MT226604	2017-02-01	Asia	Saudi Arabia	Asia	Saudi Arabia	Oronasopharynx	genome	30106	Intermediate
I7-C-SDA	*Camelus*	MERS-CoV	KT368830	2014-12-01	Asia	Saudi Arabia	Asia	Saudi Arabia	Oronasopharynx	genome	30106	Intermediate
I8-CD-SDA	*Camelus dromedarius*	MERS-CoV	KJ713297	2013-11-01	Asia	Saudi Arabia	Asia	Saudi Arabia	Oronasopharynx	genome	30106	Intermediate
I9-C-SDA	*Camelus*	MERS-CoV	KT368824	2013-11-01	Asia	Saudi Arabia	Asia	Saudi Arabia	Oronasopharynx	genome	30106	Intermediate
I10-CD-UAE	*Camelus dromedarius*	MERS-CoV	MF598637	2015-03-01	Asia	United Arab Emirates	Asia	United Arab Emirates	Oronasopharynx	genome	30106	Intermediate
I11-CD-UAE	*Camelus dromedarius*	MERS-CoV	MF598649	2015-03-01	Asia	United Arab Emirates	Asia	United Arab Emirates	Oronasopharynx	genome	30106	Intermediate
I12-CD-UAE	*Camelus dromedarius*	MERS-CoV	MF598635	2015-03-01	Asia	United Arab Emirates	Asia	United Arab Emirates	Oronasopharynx	genome	30106	Intermediate
I13-CD-UAE	*Camelus dromedarius*	MERS-CoV	MF598653	2015-03-01	Asia	United Arab Emirates	Asia	United Arab Emirates	Oronasopharynx	genome	30106	Intermediate
I14-CD-UAE	*Camelus dromedarius*	MERS-CoV	MF598656	2015-03-01	Asia	United Arab Emirates	Asia	United Arab Emirates	Oronasopharynx	genome	30106	Intermediate
I15-CD-UAE	*Camelus dromedarius*	MERS-CoV	MF598629	2015-03-01	Asia	United Arab Emirates	Asia	United Arab Emirates	Oronasopharynx	genome	30106	Intermediate
I16-C-OMN	*Camelus*	MERS-CoV	KY673149	2015-01-01	Asia	Oman	Asia	Oman	Oronasopharynx	genome	30106	Intermediate
I17-CD-UAE	*Camelus dromedarius*	MERS-CoV	MF598621	2015-03-01	Asia	United Arab Emirates	Asia	United Arab Emirates	Oronasopharynx	genome	30106	Intermediate
I18-CD-UAE	*Camelus dromedarius*	MERS-CoV	MF598696	2015-03-01	Asia	United Arab Emirates	Asia	United Arab Emirates	Oronasopharynx	genome	30106	Intermediate
I19-C-UAE	*Camelus*	MERS-CoV	KY581695	2014-06-01	Asia	United Arab Emirates	Asia	United Arab Emirates	Oronasopharynx	genome	30106	Intermediate
I20-CD-UAE	*Camelus dromedarius*	MERS-CoV	MF598654	2015-03-01	Asia	United Arab Emirates	Asia	United Arab Emirates	Oronasopharynx	genome	30106	Intermediate
I21-C-SDA	*Camelus*	MERS-CoV	KT368825	2014-07-01	Asia	Saudi Arabia	Asia	Saudi Arabia	Oronasopharynx	genome	30106	Intermediate
I22-C-SDA	*Camelus*	MERS-CoV	KT368890	2015-03-01	Asia	Saudi Arabia	Asia	Saudi Arabia	Oronasopharynx	genome	30106	Intermediate
I23-CD-UAE	*Camelus dromedarius*	MERS-CoV	MF598669	2015-03-01	Asia	United Arab Emirates	Asia	United Arab Emirates	Oronasopharynx	genome	30106	Intermediate
I24-CD-UAE	*Camelus dromedarius*	MERS-CoV	MF598641	2015-03-01	Asia	United Arab Emirates	Asia	United Arab Emirates	Oronasopharynx	genome	30106	Intermediate
I25-CD-UAE	*Camelus dromedarius*	MERS-CoV	MF598679	2015-03-01	Asia	United Arab Emirates	Asia	United Arab Emirates	Oronasopharynx	genome	30106	Intermediate
I26-CD-UAE	*Camelus dromedarius*	MERS-CoV	MF598599	2015-03-01	Asia	United Arab Emirates	Asia	United Arab Emirates	Oronasopharynx	genome	30106	Intermediate
I27-CD-UAE	*Camelus dromedarius*	MERS-CoV	MF598638	2015-03-01	Asia	United Arab Emirates	Asia	United Arab Emirates	Oronasopharynx	genome	30106	Intermediate
I28-CD-UAE	*Camelus dromedarius*	MERS-CoV	MF598652	2015-03-01	Asia	United Arab Emirates	Asia	United Arab Emirates	Oronasopharynx	genome	30106	Intermediate
I29-CD-UAE	*Camelus dromedarius*	MERS-CoV	MF598676	2015-03-01	Asia	United Arab Emirates	Asia	United Arab Emirates	Oronasopharynx	genome	30106	Intermediate
I30-CD-UAE	*Camelus dromedarius*	MERS-CoV	MF598597	2015-03-01	Asia	United Arab Emirates	Asia	United Arab Emirates	Oronasopharynx	genome	30106	Intermediate
I31-CD-UAE	*Camelus dromedarius*	MERS-CoV	MF598644	2015-03-01	Asia	United Arab Emirates	Asia	United Arab Emirates	Oronasopharynx	genome	30106	Intermediate
I32-CD-UAE	*Camelus dromedarius*	MERS-CoV	MF598624	2015-03-01	Asia	United Arab Emirates	Asia	United Arab Emirates	Oronasopharynx	genome	30106	Intermediate
I33-CD-UAE	*Camelus dromedarius*	MERS-CoV	MF598695	2015-03-01	Asia	United Arab Emirates	Asia	United Arab Emirates	Oronasopharynx	genome	30106	Intermediate
I34-CD-UAE	*Camelus dromedarius*	MERS-CoV	MF598651	2015-03-01	Asia	United Arab Emirates	Asia	United Arab Emirates	Oronasopharynx	genome	30106	Intermediate
I35-CD-UAE	*Camelus dromedarius*	MERS-CoV	MF598609	2015-03-01	Asia	United Arab Emirates	Asia	United Arab Emirates	Oronasopharynx	genome	30106	Intermediate
I36-CD-UAE	*Camelus dromedarius*	MERS-CoV	MF598712	2015-03-01	Asia	United Arab Emirates	Asia	United Arab Emirates	Oronasopharynx	genome	30106	Intermediate
I37-CD-UAE	*Camelus dromedarius*	MERS-CoV	MF598616	2015-03-01	Asia	United Arab Emirates	Asia	United Arab Emirates	Oronasopharynx	genome	30106	Intermediate
I38-CD-UAE	*Camelus dromedarius*	MERS-CoV	MF598702	2015-03-01	Asia	United Arab Emirates	Asia	United Arab Emirates	Oronasopharynx	genome	30106	Intermediate
I39-CD-UAE	*Camelus dromedarius*	MERS-CoV	MF598632	2015-03-01	Asia	United Arab Emirates	Asia	United Arab Emirates	Oronasopharynx	genome	30106	Intermediate
I40-CD-UAE	*Camelus dromedarius*	MERS-CoV	MF598617	2015-03-01	Asia	United Arab Emirates	Asia	United Arab Emirates	Oronasopharynx	genome	30106	Intermediate
I41-CD-UAE	*Camelus dromedarius*	MERS-CoV	MF598668	2015-03-01	Asia	United Arab Emirates	Asia	United Arab Emirates	Oronasopharynx	genome	30106	Intermediate
I42-CD-UAE	*Camelus dromedarius*	MERS-CoV	MF598701	2015-03-01	Asia	United Arab Emirates	Asia	United Arab Emirates	Oronasopharynx	genome	30106	Intermediate
I43-CD-UAE	*Camelus dromedarius*	MERS-CoV	MF598620	2015-03-01	Asia	United Arab Emirates	Asia	United Arab Emirates	Oronasopharynx	genome	30106	Intermediate
I44-CD-UAE	*Camelus dromedarius*	MERS-CoV	MF598594	2015-03-01	Asia	United Arab Emirates	Asia	United Arab Emirates	Oronasopharynx	genome	30106	Intermediate
I45-CD-UAE	*Camelus dromedarius*	MERS-CoV	MF598643	2015-03-01	Asia	United Arab Emirates	Asia	United Arab Emirates	Oronasopharynx	genome	30106	Intermediate
H1-USA	*Homo sapiens*	MERS-CoV	KP223131	2014-06-01	North America	USA	North America	USA	Lung, Oronasopharynx	genome	30106	Human
H2-USA	*Homo sapiens*	MERS-CoV	MK039552	2014-04-21	Asia	Jordan	Asia	Jordan	Lung, Oronasopharynx	genome	30106	Human
H3-SDA	*Homo sapiens*	MERS-CoV	KF186567	2013-05-09	Asia	Saudi Arabia	Asia	Saudi Arabia		genome	30106	Human
H4-OMN	*Homo sapiens*	MERS-CoV	KY673148	2015-01-06	Asia	Oman	Asia	Oman	Oronasopharynx	genome	30106	Human
H5-UAE	*Homo sapiens*	MERS-CoV	KP209310	2014-03-07	Asia	United Arab Emirates	Asia	United Arab Emirates	Lung, Oronasopharynx	genome	30106	Human
H6-UAE	*Homo sapiens*	MERS-CoV	KY581686	2014-04-10	Asia	United Arab Emirates	Asia	United Arab Emirates	Lung, Oronasopharynx	genome	30106	Human
H7-UAE	*Homo sapiens*	MERS-CoV	KP209307	2014-04-10	Asia	United Arab Emirates	Asia	United Arab Emirates	Lung, Oronasopharynx	genome	30106	Human
H8-UAE	*Homo sapiens*	MERS-CoV	KY581694	2014-01-01	Asia	United Arab Emirates	Asia	United Arab Emirates	Lung, Oronasopharynx	genome	30106	Human
H9-OMN	*Homo sapiens*	MERS-CoV	KT156561	2013-12-28	Asia	Oman	Asia	Oman	Oronasopharynx	genome	30106	Human
H10-UAE	*Homo sapiens*	MERS-CoV	KY581684	2013-07-10	Asia	United Arab Emirates	Asia	United Arab Emirates	Lung, Oronasopharynx	genome	30106	Human
H11-UAE	*Homo sapiens*	MERS-CoV	KP209312	2013-11-15	Asia	United Arab Emirates	Asia	United Arab Emirates	Lung	genome	30106	Human
H12-UAE	*Homo sapiens*	MERS-CoV	KY581687	2013-12-23	Asia	United Arab Emirates	Asia	United Arab Emirates	Lung, Oronasopharynx	genome	30106	Human
H13-OMN	*Homo sapiens*	MERS-CoV	KT156560	2013-10-28	Asia	Oman	Asia	Oman	Oronasopharynx	genome	30106	Human
H14-SDA	*Homo sapiens*	MERS-CoV	MH013216	2015-10-15	Asia	Saudi Arabia	Asia	Saudi Arabia		genome	30106	Human
H15-SDA	*Homo sapiens*	MERS-CoV	MG912603	2017-06-12	Asia	Saudi Arabia	Asia	Saudi Arabia	Oronasopharynx	genome	30106	Human
H16-SDA	*Homo sapiens*	MERS-CoV	MG546331	2015-10-15	Asia	Saudi Arabia	Asia	Saudi Arabia	Oronasopharynx	genome	30106	Human
H17-SKA	*Homo sapiens*	MERS-CoV	MH029552	2015-08-26	Asia	Saudi Arabia	Asia	Saudi Arabia	Oronasopharynx	genome	30106	Human
H18-SKA	*Homo sapiens*	MERS-CoV	KX034099	2015-06-26	Asia	South Korea	Asia	South Korea	Lung, Oronasopharynx	genome	30106	Human
H19-SKA	*Homo sapiens*	MERS-CoV	KX034095	2015-06-04	Asia	South Korea	Asia	South Korea	Lung, Oronasopharynx	genome	30106	Human
H20-SDA	*Homo sapiens*	MERS-CoV	KT026454	2015-03-01	Asia	Saudi Arabia	Asia	Saudi Arabia	Oronasopharynx	genome	30106	Human
H21-SDA	*Homo sapiens*	MERS-CoV	MN120513	2019-03-27	Asia	Saudi Arabia	Asia	Saudi Arabia		genome	30106	Human
H22-SDA	*Homo sapiens*	MERS-CoV	MN120514	2019-03-27	Asia	Saudi Arabia	Asia	Saudi Arabia		genome	30106	Human
H23-SDA	*Homo sapiens*	MERS-CoV	MK462249	2018-07-09	Asia	Saudi Arabia	Asia	Saudi Arabia	Oronasopharynx	genome	30106	Human
H24-SDA	*Homo sapiens*	MERS-CoV	MK483839	2018-08-16	Asia	Saudi Arabia	Asia	Saudi Arabia	Oronasopharynx	genome	30106	Human
H25-SDA	*Homo sapiens*	MERS-CoV	MK462250	2018-07-12	Asia	Saudi Arabia	Asia	Saudi Arabia	Oronasopharynx	genome	30106	Human
H26-SDA	*Homo sapiens*	MERS-CoV	MK462248	2018-06-13	Asia	Saudi Arabia	Asia	Saudi Arabia	Oronasopharynx	genome	30106	Human
H27-SDA	*Homo sapiens*	MERS-CoV	MN365232	2019-01-28	Asia	Saudi Arabia	Asia	Saudi Arabia	Oronasopharynx	genome	30106	Human
H28-SDA	*Homo sapiens*	MERS-CoV	MN723544	2018-08-30	Asia	Saudi Arabia	Asia	Saudi Arabia		genome	30106	Human
H29-SDA	*Homo sapiens*	MERS-CoV	MK462254	2018-08-31	Asia	Saudi Arabia	Asia	Saudi Arabia	Oronasopharynx	genome	30106	Human
H30-SDA	*Homo sapiens*	MERS-CoV	MK462251	2018-07-18	Asia	Saudi Arabia	Asia	Saudi Arabia	Oronasopharynx	genome	30106	Human
H31-SDA	*Homo sapiens*	MERS-CoV	MK462247	2018-06-08	Asia	Saudi Arabia	Asia	Saudi Arabia	Oronasopharynx	genome	30106	Human
H32-SDA	*Homo sapiens*	MERS-CoV	MK462256	2018-09-15	Asia	Saudi Arabia	Asia	Saudi Arabia	Oronasopharynx	genome	30106	Human
H33-SDA	*Homo sapiens*	MERS-CoV	MK462252	2018-08-03	Asia	Saudi Arabia	Asia	Saudi Arabia	Oronasopharynx	genome	30106	Human
H34-SDA	*Homo sapiens*	MERS-CoV	MK462244	2017-08-07	Asia	Saudi Arabia	Asia	Saudi Arabia	Oronasopharynx	genome	30106	Human
H35-SDA-RE	*Homo sapiens*	MERS-CoV	NC_019843	2012-06-13	Asia	Saudi Arabia	Asia	Saudi Arabia	Oronasopharynx	genome	30106	Human

SARS-CoV-2: Metadata

strain	specie	virus	gisaid_epi_isl	date	region	country	region_exposure	country_exposure	source	segment	length	host	pango_lineag	clade
B1-RS-CMD	*Rhinolophus shameli*	SARS-CoV-2	EPI_ISL_852604	2010-12-06	Asia	Cambodia	Asia	Cambodia	Feces	genome	29767	Bat	A	S
B2-RS-CMD	*Rhinolophus shameli*	SARS-CoV-2	EPI_ISL_852605	2010-12-06	Asia	Cambodia	Asia	Cambodia	Feces	genome	29767	Bat	A	S
B3-RA-CHA	*Rhinolophus affinis*	SARS-CoV-2	EPI_ISL_402131	2013-07-24	Asia	China	Asia	China	Feces	genome	29844	Bat	B.1.177	S
I1-MJ-CHA	*Manis javanica*	SARS-CoV-2	EPI_ISL_410721	2019-07-11	Asia	China	Asia	China	Lung	genome	29728	Intermediate	A	S
I2-PL-USA	*Panthera leo*	SARS-CoV-2	EPI_ISL_566038	2020-04-04	North America	USA	North America	USA	Feces	genome	29774	Intermediate	B	V
I3-PL-USA	*Panthera leo*	SARS-CoV-2	EPI_ISL_566044	2020-04-04	North America	USA	North America	USA	Feces	genome	29774	Intermediate	B	V
I4-EV-USA	*Environment*	SARS-CoV-2	EPI_ISL_434677	2020-03-25	North America	USA	North America	USA	Air	genome	29774	Intermediate	A	S
I5-MA-CHA	*Mesocricetus auratus*	SARS-CoV-2		2020-05-15	Asia	China	Asia	China	Oronasopharynx	genome	29774	Intermediate	B.39	
I6-MA-CHA	*Mesocricetus auratus*	SARS-CoV-2		2020-05-15	Asia	China	Asia	China	Oronasopharynx	genome	29774	Intermediate	B	
I7-MA-CHA	*Mesocricetus auratus*	SARS-CoV-2		2020-05-15	Asia	China	Asia	China	Oronasopharynx	genome	29774	Intermediate	B	
I8-MA-CHA	*Mesocricetus auratus*	SARS-CoV-2		2020-05-15	Asia	China	Asia	China	Oronasopharynx	genome	29774	Intermediate	B	
I9-EV-CHA	*Environment*	SARS-CoV-2	EPI_ISL_408515	2020-01-01	Asia	China	Asia	China	Huanan Seafood Market	genome	29774	Intermediate	B	L
I10-EV-CHA	*Environment*	SARS-CoV-2	EPI_ISL_408514	2020-01-01	Asia	China	Asia	China	Huanan Seafood Market	genome	29774	Intermediate	B	L
I11-NV-LIT	*Neovison vison*	SARS-CoV-2	EPI_ISL_851056	2020-11-01	Europe	Lithuania	Europe	Lithuania	Throat swab	genome	29774	Intermediate	B.1.1.464¬¨,Ä†	GR
I12-NV-LIT	*Neovison vison*	SARS-CoV-2	EPI_ISL_851057	2020-11-01	Europe	Lithuania	Europe	Lithuania	Throat swab	genome	29774	Intermediate	B.1.1.464¬¨,Ä†	GR
I13-NV-DMK	*Neovison vison*	SARS-CoV-2	EPI_ISL_683175	2020-11-13	Europe	Denmark	Europe	Denmark	Throat swab	genome	29774	Intermediate	B.1.1.219	GR
I14-NV-DMK	*Neovison vison*	SARS-CoV-2	EPI_ISL_641422	2020-10-16	Europe	Denmark	Europe	Denmark	Throat swab	genome	29774	Intermediate	B.1.536	G
I15-NV-DMK	*Neovison vison*	SARS-CoV-2	EPI_ISL_683023	2020-11-04	Europe	Denmark	Europe	Denmark	Throat swab	genome	29774	Intermediate	B.1.536	G
I16-NV-DMK	*Neovison vison*	SARS-CoV-2	EPI_ISL_683005	2020-11-05	Europe	Denmark	Europe	Denmark	Throat swab	genome	29774	Intermediate	B.1.536	G
I17-NV-POL	*Neovison vison*	SARS-CoV-2	EPI_ISL_732949	2020-11-17	Europe	Poland	Europe	Poland	Throat swab	genome	29774	Intermediate	B.1.1	GR
I18-NV-POL	*Neovison vison*	SARS-CoV-2	EPI_ISL_732955	2020-11-17	Europe	Poland	Europe	Poland	Throat swab	genome	29774	Intermediate	B.1.1	GR
I19-NV-LIT	*Neovison vison*	SARS-CoV-2	EPI_ISL_851058	2020-11-01	Europe	Lithuania	Europe	Lithuania	Throat swab	genome	29774	Intermediate	B.1.343	GH
I20-NV-LIT	*Neovison vison*	SARS-CoV-2	EPI_ISL_851060	2020-11-01	Europe	Lithuania	Europe	Lithuania	Throat swab	genome	29774	Intermediate	B.1.343	GH
I21-FC-RUS	*Felis catus*	SARS-CoV-2	EPI_ISL_811147	2021-01-06	Asia	Russia	Asia	Russia	Oropharyngeal swab	genome	29774	Intermediate	B.1.1.317	GR
I22-NV-DMK	*Neovison vison*	SARS-CoV-2	EPI_ISL_641414	2020-10-26	Europe	Denmark	Europe	Denmark	Throat swab	genome	29774	Intermediate	B.1.1.170	GR
I23-NV-DMK	*Neovison vison*	SARS-CoV-2	EPI_ISL_683215	2020-10-23	Europe	Denmark	Europe	Denmark	Throat swab	genome	29774	Intermediate	B.1.1.170¬¨,Ä†	GR
I24-NV-NTL	*Neovison vison*	SARS-CoV-2	EPI_ISL_577802	2020-09-02	Europe	Netherlands	Europe	Netherlands	Throat swab	genome	29774	Intermediate	B.1.22	GH
I25-NV-DMK	*Neovison vison*	SARS-CoV-2	EPI_ISL_683216	2020-10-23	Europe	Denmark	Europe	Denmark	Throat swab	genome	29774	Intermediate	B.1.1.294	GR
I26-NV-DMK	*Neovison vison*	SARS-CoV-2	EPI_ISL_683218	2020-10-23	Europe	Denmark	Europe	Denmark	Throat swab	genome	29774	Intermediate	B.1.1.294	GR
I27-NV-NTL	*Neovison vison*	SARS-CoV-2	EPI_ISL_431778	2020-04-24	Europe	Netherlands	Europe	Netherlands	Lung	genome	29774	Intermediate	B.1.8	G
I28-NV-NTL	*Neovison vison*	SARS-CoV-2	EPI_ISL_522992	2020-04-28	Europe	Netherlands	Europe	Netherlands	Throat swab	genome	29774	Intermediate	B.1.8	G
I29-NV-NTL	*Neovison vison*	SARS-CoV-2	EPI_ISL_447625	2020-04-29	Europe	Netherlands	Europe	Netherlands	Throat swab	genome	29774	Intermediate	B.1.8	G
I30-NV-NTL	*Neovison vison*	SARS-CoV-2	EPI_ISL_523009	2020-06-07	Europe	Netherlands	Europe	Netherlands	Throat swab	genome	29774	Intermediate	B.1.8	G
I31-NV-NTL	*Neovison vison*	SARS-CoV-2	EPI_ISL_523006	2020-06-07	Europe	Netherlands	Europe	Netherlands	Throat swab	genome	29774	Intermediate	B.1.8	G
I32-CL-ITY	*Canis lupus familiaris*	SARS-CoV-2	EPI_ISL_730652	2020-11-04	Europe	Italy	Europe	Italy	Nasopharyngeal swab	genome	29774	Intermediate	B.1.177	GV
I33-NV-NTL	*Neovison vison*	SARS-CoV-2	EPI_ISL_523083	2020-07-26	Europe	Netherlands	Europe	Netherlands	Throat swab	genome	29774	Intermediate	B.1.149	G
I34-CL-USA	*Canis lupus familiaris*	SARS-CoV-2	EPI_ISL_699508	2020-07-28	North America	USA	North America	USA	Respiratory swab	genome	29774	Intermediate	B.1.1	GR
I35-NV-NTL	*Neovison vison*	SARS-CoV-2	EPI_ISL_523106	2020-05-30	Europe	Netherlands	Europe	Netherlands	Throat swab	genome	29774	Intermediate	B.1.22	GH
I36-FC-USA	*Felis catus*	SARS-CoV-2	EPI_ISL_699507	2020-07-17	North America	USA	North America	USA	Rectal swab	genome	29774	Intermediate	B.1.571	GH
I37-FC-USA	*Felis catus*	SARS-CoV-2	EPI_ISL_699506	2020-06-28	North America	USA	North America	USA	Respiratory swab	genome	29774	Intermediate	B.1.234	G
I38-NV-NTL	*Neovison vison*	SARS-CoV-2	EPI_ISL_522995	2020-06-07	Europe	Netherlands	Europe	Netherlands	Throat swab	genome	29774	Intermediate	B.1	G
I39-FC-FRE	*Felis catus*	SARS-CoV-2	EPI_ISL_483063	2020-05-14	Europe	France	Europe	France	Home Environment	genome	29774	Intermediate	B.1.1.254	GR
I40-EV-CHA	*Environment*	SARS-CoV-2	EPI_ISL_469256	2020-06-11	Asia	China	Asia	China	Xinfadi Wholesale Market	genome	29774	Intermediate	B.1.1	GR
I41-EV-CHA	*Environment*	SARS-CoV-2	EPI_ISL_430743	2020-03-14	Asia	China	Asia	China	Enviromental swab	genome	29774	Intermediate	B.1	G
I42-PT-USA	*Panthera tigris jacksoni*	SARS-CoV-2	EPI_ISL_566040	2020-04-04	North America	USA	North America	USA	Feces	genome	29774	Intermediate	B.1	GH
I43-FC-CHA	*Felis catus*	SARS-CoV-2	EPI_ISL_759858	2020-03-30	Asia	China	Asia	China		genome	29774	Intermediate	B.1	G
I44-EV-USA	*Environment*	SARS-CoV-2	EPI_ISL_455682	2020-04-23	North America	USA	North America	USA	Air	genome	29774	Intermediate	B.1.595	GH
I45-EV-USA	*Environment*	SARS-CoV-2	EPI_ISL_477160	2020-10-16	North America	USA	North America	USA	Hospital Air	genome	29774	Intermediate	B.1	GH
I46-PT-USA	*Panthera tigris jacksoni*	SARS-CoV-2	EPI_ISL_420293	2020-04-02	North America	USA	North America	USA	Nasal swab	genome	29774	Intermediate	B.1	GH
I47-FC-BEL	*Felis catus*	SARS-CoV-2	EPI_ISL_487275	2020-03-11	Europe	Belgium	Europe	Belgium	Vomit fluid	genome	29774	Intermediate	B.1	G
I48-MA-CHA	*Mesocricetus auratus*	SARS-CoV-2		2020-05-15	Asia	China	Asia	China	Oronasopharynx	genome	29774	Intermediate	B.1	
H1-SKA	*Homo sapiens*	SARS-CoV-2		2020-03-11	Asia	South Korea	Asia	South Korea	Oronasopharynx	genome	29822	Human	A	
H2-ENG	*Homo sapiens*	SARS-CoV-2		2020-12-17	Europe	England	Europe	England	Oropharyngeal swab	genome	29822	Human	B.1.1.7¬¨,Ä†	
H3-ENG	*Homo sapiens*	SARS-CoV-2		2020-08-23	Europe	England	Europe	England	Oropharyngeal swab	genome	29822	Human	B.1.177	
H4-CHA	*Homo sapiens*	SARS-CoV-2		2020-01-22	Asia	China	Asia	China	Oropharyngeal swabs	genome	29822	Human	B	
H5-CHA	*Homo sapiens*	SARS-CoV-2		2020-02-10	Asia	China	Asia	China	Oronasopharynx	genome	29822	Human	B.5	
H6-CHA	*Homo sapiens*	SARS-CoV-2		2020-01-22	Asia	China	Asia	China	Oronasopharynx	genome	29822	Human	B	
H7-CHA	*Homo sapiens*	SARS-CoV-2		2020-01-22	Asia	China	Asia	China	Oronasopharynx	genome	29822	Human	A	
H8-CHA	*Homo sapiens*	SARS-CoV-2		2020-02-02	Asia	China	Asia	China	Oronasopharynx	genome	29822	Human	B	
H9-CHA-RE	*Homo sapiens*	SARS-CoV-2		2019-12-01	Asia	China	Asia	China	Oronasopharynx	genome	29822	Human	B	
H10-ITY	*Homo sapiens*	SARS-CoV-2		2020-12-27	Europe	Italy	Europe	Italy	Oronasopharynx	genome	29822	Human	B.1.1.7	
H11-PAL	*Homo sapiens*	SARS-CoV-2	EPI_ISL_1502574	2021-02-06	Asia	Palestine	Asia	Palestine	Nasal swab	genome	29822	Human	B.1.1.7	GR
H12-LET	*Homo sapiens*	SARS-CoV-2	EPI_ISL_1590998	2021-03-17	Europe	Latvia	Europe	Latvia	Nasal swab	genome	29822	Human	B.1.177.79	GV
H13-LET	*Homo sapiens*	SARS-CoV-2	EPI_ISL_1590430	2021-03-09	Europe	Latvia	Europe	Latvia	Nasal swab	genome	29822	Human	S.1	GR
H14-LET	*Homo sapiens*	SARS-CoV-2	EPI_ISL_1590432	2021-03-09	Europe	Latvia	Europe	Latvia	Nasal swab	genome	29822	Human	S.1	GR
H15-MYA	*Homo sapiens*	SARS-CoV-2	EPI_ISL_1424467	2021-03-02	Asia	Malaysia	Asia	Malaysia	Nasal swab	genome	29822	Human	B.1.466.2	GH
H16-MYA	*Homo sapiens*	SARS-CoV-2	EPI_ISL_1424470	2021-03-01	Asia	Malaysia	Asia	Malaysia	Nasal swab	genome	29822	Human	B.1.466.2	GH
H17-USA	*Homo sapiens*	SARS-CoV-2		2021-02-13	North America	USA	North America	USA	Oronasopharynx	genome	29822	Human	B.1.1.519	
H18-ENG	*Homo sapiens*	SARS-CoV-2		2020-12-19	Europe	England	Europe	England	Oropharyngeal swab	genome	29822	Human	B.1.1.519	
H19-TUS	*Homo sapiens*	SARS-CoV-2	EPI_ISL_635062	2020-11-02	Africa	Tunisia	Africa	Tunisia	Nasopharyngeal swab	genome	29822	Human	B.1.160	GH
H20-ENG	*Homo sapiens*	SARS-CoV-2		2020-09-18	Europe	England	Europe	England	Oropharyngeal swab	genome	29822	Human	B.1.160	
H21-LET	*Homo sapiens*	SARS-CoV-2	EPI_ISL_1590520	2021-03-09	Europe	Latvia	Europe	Latvia	Nasal swab	genome	29822	Human	B.1.1.374	GR
H22-LET	*Homo sapiens*	SARS-CoV-2	EPI_ISL_1591003	2021-03-17	Europe	Latvia	Europe	Latvia	Nasal swab	genome	29822	Human	B.1.1.374	GR
H23-USA	*Homo sapiens*	SARS-CoV-2		2021-02-18	North America	USA	North America	USA	Oronasopharynx	genome	29822	Human	B.1.561	
H24-BRL	*Homo sapiens*	SARS-CoV-2	EPI_ISL_1358305	2021-03-02	South America	Brazil	South America	Brazil	Nasopharyngeal swab	genome	29822	Human	B.1.1.28	GR
H25-EGP	*Homo sapiens*	SARS-CoV-2		2020-07-12	Africa	Egypt	Africa	Egypt	Oronasopharynx	genome	29822	Human	B.1.561	
H26-BRL	*Homo sapiens*	SARS-CoV-2	EPI_ISL_1078991	2021-01-22	South America	Brazil	South America	Brazil	Nasopharyngeal swab	genome	29822	Human	B.1.1.28	GR
H27-COL	*Homo sapiens*	SARS-CoV-2	EPI_ISL_1235687	2021-01-30	South America	Colombia	South America	Colombia	Oropharyngeal swab	genome	29822	Human	B.1.111	GH
H28-USA	*Homo sapiens*	SARS-CoV-2		2021-02-16	North America	USA	North America	USA	Oronasopharynx	genome	29822	Human	B.1.2	
H29-USA	*Homo sapiens*	SARS-CoV-2		2021-03-08	North America	USA	North America	USA	Oronasopharynx	genome	29822	Human	B.1.1.274	
H30-USA	*Homo sapiens*	SARS-CoV-2		2021-02-22	North America	USA	North America	USA	Oronasopharynx	genome	29822	Human	B.1	
H31-BRL	*Homo sapiens*	SARS-CoV-2	EPI_ISL_1293079	2021-02-02	South America	Brazil	South America	Brazil	Nasopharyngeal swab	genome	29822	Human	P.2	GH
H32CLE	*Homo sapiens*	SARS-CoV-2	EPI_ISL_1167710	2020-11-15	South America	Chile	South America	Chile	Nasopharyngeal swab	genome	29822	Human	B.1.1¬¨,Ä†	GR
H33-CLE	*Homo sapiens*	SARS-CoV-2	EPI_ISL_1167701	2020-10-11	South America	Chile	South America	Chile	Nasopharyngeal swab	genome	29822	Human	C.26¬¨,Ä†	GR
H34-USA	*Homo sapiens*	SARS-CoV-2		2021-02-24	North America	USA	North America	USA	Oronasopharynx	genome	29822	Human	B.1.478	
H35-USA	*Homo sapiens*	SARS-CoV-2		2021-03-12	North America	USA	North America	USA	Oronasopharynx	genome	29822	Human	B.1.429	
H36-USA	*Homo sapiens*	SARS-CoV-2		2021-03-04	North America	USA	North America	USA	Oronasopharynx	genome	29822	Human	B.1.2	
H37-LET	*Homo sapiens*	SARS-CoV-2	EPI_ISL_1591001	2021-03-17	Europe	Latvia	Europe	Latvia	Nasal swab	genome	29822	Human	B.1.177¬¨,Ä†	GV
H38-LET	*Homo sapiens*	SARS-CoV-2	EPI_ISL_1590465	2021-03-16	Europe	Latvia	Europe	Latvia	Nasal swab	genome	29822	Human	B.1.177¬¨,Ä†	GV
H39-BRL	*Homo sapiens*	SARS-CoV-2	EPI_ISL_1121317	2021-02-09	South America	Brazil	South America	Brazil	Nasopharyngeal swab	genome	29822	Human	B.1.1.28	GR
H40-BRL	*Homo sapiens*	SARS-CoV-2	EPI_ISL_1533705	2021-02-21	South America	Brazil	South America	Brazil	Nasopharyngeal swab	genome	29822	Human	P.2	GR
H41-USA	*Homo sapiens*	SARS-CoV-2		2021-02-22	North America	USA	North America	USA	Oronasopharynx	genome	29822	Human	B.1.36.31	
H42-BRL	*Homo sapiens*	SARS-CoV-2	EPI_ISL_1445177	2021-03-10	South America	Brazil	South America	Brazil	Nasopharyngeal swab	genome	29822	Human	B.1.1¬¨,Ä†	GR
H43-USA	*Homo sapiens*	SARS-CoV-2		2021-04-02	North America	USA	North America	USA	Oronasopharynx	genome	29822	Human	B.1	
H44-SEN	*Homo sapiens*	SARS-CoV-2	EPI_ISL_1167174	2021-01-25	Africa	Senegal	Africa	Senegal	Oropharyngeal swab	genome	29822	Human	B.1.1.420	O

H45-LET	*Homo sapiens*	SARS-CoV-2	EPI_ISL_1590519	2021-03-09	Europe	Latvia	Europe	Latvia	Nasal swab	genome	29822	Human	B.1.177	GV
H46-USA	*Homo sapiens*	SARS-CoV-2		2021-02-19	North America	USA	North America	USA	Oronasopharynx	genome	29822	Human	B.1.561	
H47-EGP	*Homo sapiens*	SARS-CoV-2		2020-07-04	Africa	Egypt	Africa	Egypt	Oronasopharynx	genome	29822	Human	B.1	
H48-BRL	*Homo sapiens*	SARS-CoV-2	EPI_ISL_1079159	2021-02-03	South America	Brazil	South America	Brazil	Nasopharyngeal swab	genome	29822	Human	N.9¬¨,Ä†	GR
H49-PER	*Homo sapiens*	SARS-CoV-2	EPI_ISL_1111317	2021-01-17	South America	Peru	South America	Peru	Oropharyngeal swab	genome	29822	Human	B.1.1.1	GR
H50-PER	*Homo sapiens*	SARS-CoV-2	EPI_ISL_1111348	2021-01-16	South America	Peru	South America	Peru	Oropharyngeal swab	genome	29822	Human	C.4	GR
H51-ITY	*Homo sapiens*	SARS-CoV-2		2020-12-10	Europe	Italy	Europe	Italy	Oronasopharynx	genome	29822	Human	B.1.258	
H52-LET	*Homo sapiens*	SARS-CoV-2	EPI_ISL_1590935	2021-03-09	Europe	Latvia	Europe	Latvia	Nasal swab	genome	29822	Human	B.1.1.374	GR
H53-LET	*Homo sapiens*	SARS-CoV-2	EPI_ISL_1590995	2021-03-16	Europe	Latvia	Europe	Latvia	Nasal swab	genome	29822	Human	B.1.177.12¬¨,Ä†	GV
H54-STD	*Homo sapiens*	SARS-CoV-2		2020-10-14	Europe	Scotland	Europe	Scotland	Oropharyngeal swab	genome	29822	Human	B.1.177.57	
H55-USA	*Homo sapiens*	SARS-CoV-2		2021-03-03	North America	USA	North America	USA	Oronasopharynx	genome	29822	Human	B.1.1.432	
H56-GHA	*Homo sapiens*	SARS-CoV-2	EPI_ISL_1018073	2021-01-10	Africa	Ghana	Africa	Ghana	Nasopharyngeal swab	genome	29822	Human	L.3¬¨,Ä†	GR
H57-BRL	*Homo sapiens*	SARS-CoV-2	EPI_ISL_1445201	2021-03-15	South America	Brazil	South America	Brazil	Nasopharyngeal swab	genome	29822	Human	B.1.1.28	GR
H58-COL	*Homo sapiens*	SARS-CoV-2	EPI_ISL_1577390	2021-01-08	South America	Colombia	South America	Colombia	Oropharyngeal swab	genome	29822	Human	B.1.1.348	GR
H59-COL	*Homo sapiens*	SARS-CoV-2	EPI_ISL_1577026	2020-10-27	South America	Colombia	South America	Colombia	Oropharyngeal swab	genome	29822	Human	B.1.1.348	GR
H60-COL	*Homo sapiens*	SARS-CoV-2	EPI_ISL_1576835	2020-10-25	South America	Colombia	South America	Colombia	Oropharyngeal swab	genome	29822	Human	B.1.1.348	GR
H61-LET	*Homo sapiens*	SARS-CoV-2	EPI_ISL_1590933	2021-03-09	Europe	Latvia	Europe	Latvia	Nasal swab	genome	29822	Human	B.1.1.429	GR
H62-LET	*Homo sapiens*	SARS-CoV-2	EPI_ISL_1590947	2021-03-15	Europe	Latvia	Europe	Latvia	Nasal swab	genome	29822	Human	Z.1¬¨,Ä†	GV
H63-LET	*Homo sapiens*	SARS-CoV-2	EPI_ISL_1590894	2021-03-09	Europe	Latvia	Europe	Latvia	Nasal swab	genome	29822	Human	B.1.177	GV
H64-CJD	*Homo sapiens*	SARS-CoV-2		2020-11-29	Asia	West Blank	Asia	West Blank	Oronasopharynx	genome	29822	Human	B.1.1.50	
H65-ENG	*Homo sapiens*	SARS-CoV-2		2020-08-28	Europe	England	Europe	England	Oropharyngeal swab	genome	29822	Human	B.1.367	
H66-USA	*Homo sapiens*	SARS-CoV-2		2021-03-10	North America	USA	North America	USA	Oronasopharynx	genome	29822	Human	B.1.427	
H67-ENG	*Homo sapiens*	SARS-CoV-2		2020-09-07	Europe	England	Europe	England	Oropharyngeal swab	genome	29822	Human	B.1.218	
H68-ENG	*Homo sapiens*	SARS-CoV-2		2020-12-17	Europe	England	Europe	England	Oropharyngeal swab	genome	29822	Human	B.1.408	
H69-LET	*Homo sapiens*	SARS-CoV-2	EPI_ISL_1590991	2021-03-16	Europe	Latvia	Europe	Latvia	Nasal swab	genome	29822	Human	B.1.177.79	GV
H70-ENG	*Homo sapiens*	SARS-CoV-2		2020-11-12	Europe	England	Europe	England	Oropharyngeal swab	genome	29822	Human	B.1.258.3	
H71-SPN	*Homo sapiens*	SARS-CoV-2		2021-02-07	Europe	Spain	Europe	Spain	Oronasopharynx	genome	29822	Human	B.1.177	
H72-LET	*Homo sapiens*	SARS-CoV-2	EPI_ISL_1590874	2021-03-10	Europe	Latvia	Europe	Latvia	Nasal swab	genome	29822	Human	U.3¬¨,Ä†	GV
H73-COL	*Homo sapiens*	SARS-CoV-2	EPI_ISL_1235686	2021-01-26	South America	Colombia	South America	Colombia	Nasopharyngeal swab	genome	29822	Human	B.1.111	GH
H74-ENG	*Homo sapiens*	SARS-CoV-2		2020-11-02	Europe	England	Europe	England	Oropharyngeal swab	genome	29822	Human	B.1	
H75-ENG	*Homo sapiens*	SARS-CoV-2		2020-09-25	Europe	England	Europe	England	Oropharyngeal swab	genome	29822	Human	AD.2¬¨,Ä†	
H76-MYA	*Homo sapiens*	SARS-CoV-2	EPI_ISL_1424065	2021-01-11	Asia	Malaysia	Asia	Malaysia	Left lung	genome	29822	Human	B.1.524¬¨,Ä†	G
H77-ENG	*Homo sapiens*	SARS-CoV-2		2020-11-01	Europe	England	Europe	England	Oropharyngeal swab	genome	29822	Human	B.1.1.303	
H78-ENG	*Homo sapiens*	SARS-CoV-2		2020-11-12	Europe	England	Europe	England	Oropharyngeal swab	genome	29822	Human	B.1.1.170	
H79-ENG	*Homo sapiens*	SARS-CoV-2		2020-11-12	Europe	England	Europe	England	Oropharyngeal swab	genome	29822	Human	B.1.177	
H80-TUS	*Homo sapiens*	SARS-CoV-2	EPI_ISL_699657	2020-03-16	Africa	Tunisia	Africa	Tunisia	Nasopharyngeal swab	genome	29822	Human	B.1.177	GV
H81-EGP	*Homo sapiens*	SARS-CoV-2	EPI_ISL_1141525	2020-06-08	Africa	Egypt	Africa	Egypt	Oropharyngeal swab	genome	29822	Human	C.36	GR
H82-ENG	*Homo sapiens*	SARS-CoV-2		2020-12-18	Europe	England	Europe	England	Oropharyngeal swab	genome	29822	Human	B.1.568	
H83-ENG	*Homo sapiens*	SARS-CoV-2		2020-09-19	Europe	England	Europe	England	Oropharyngeal swab	genome	29822	Human	B.1.2	
H84-LET	*Homo sapiens*	SARS-CoV-2	EPI_ISL_1590957	2021-03-16	Europe	Latvia	Europe	Latvia	Nasal swab	genome	29822	Human	B.1.1.67	GR
H85-CZH	*Homo sapiens*	SARS-CoV-2	EPI_ISL_1588539	2021-03-15	Europe	Czech Republic	Europe	Czech Republic	Nasopharyngeal swab	genome	29822	Human	B.1.258	G
H86-LET	*Homo sapiens*	SARS-CoV-2	EPI_ISL_1590463	2021-03-09	Europe	Latvia	Europe	Latvia	Nasal swab	genome	29822	Human	B.1.1.141	GR
H87-PER	*Homo sapiens*	SARS-CoV-2	EPI_ISL_593774	2020-04-07	South America	Peru	South America	Peru	Nasopharyngeal swab	genome	29822	Human	C.14	GR
H88-EGP	*Homo sapiens*	SARS-CoV-2	EPI_ISL_1109627	2020-06-13	Africa	Egypt	Africa	Egypt	Oropharyngeal swab	genome	29822	Human	B.1¬¨,Ä†	GH
H89-CLE	*Homo sapiens*	SARS-CoV-2	EPI_ISL_445359	2020-03-16	South America	Chile	South America	Chile	Throat swab	genome	29822	Human	B.1.1¬¨,Ä†	GR
H90-JPN	*Homo sapiens*	SARS-CoV-2		2020-06-01	Asia	Japan	Asia	Japan	Oronasopharynx	genome	29822	Human	B.1.1.283	
H91-CLE	*Homo sapiens*	SARS-CoV-2	EPI_ISL_445362	2020-03-17	South America	Chile	South America	Chile	Throat swab	genome	29822	Human	B.1.1.33¬¨,Ä†	GR
H92-CLE	*Homo sapiens*	SARS-CoV-2	EPI_ISL_445370	2020-04-05	South America	Chile	South America	Chile	Throat swab	genome	29822	Human	B.1.1.33	GR
H93-CHA	*Homo sapiens*	SARS-CoV-2		2020-01-22	Asia	China	Asia	China	Lung, Oronasopharynx	genome	29822	Human	B	
H94-CHA	*Homo sapiens*	SARS-CoV-2		2020-01-03	Asia	China	Asia	China	Lung, Oronasopharynx	genome	29822	Human	B	
H95-MRO	*Homo sapiens*	SARS-CoV-2	EPI_ISL_451400	2020-04-23	Asia	China	Asia	China	Nasopharyngeal swab	genome	29822	Human	B.1.528	G
H96-COL	*Homo sapiens*	SARS-CoV-2	EPI_ISL_445219	2020-04-02	Asia	China	Asia	China	Nasopharyngeal swab	genome	29822	Human	B.1	GH
H97-CLE	*Homo sapiens*	SARS-CoV-2	EPI_ISL_445295	2020-03-21	Asia	China	Asia	China	Throat swab	genome	29822	Human	B.1¬¨,Ä†	G
H98-COL	*Homo sapiens*	SARS-CoV-2	EPI_ISL_456153	2020-04-22	Asia	China	Asia	China	Nasopharyngeal swab	genome	29822	Human	B.1¬¨,Ä†	GH

Table S4. Natural selection and inferred substitutions in the predicted CDSs for *Beta-CoVs*.

SARS-CoV: Natural selection and inferred substitutions in the predicted CDSs for SARS-CoV

**CDS**	**Test**	**Negative/Purifyng**			**Positive/Diversifying**		
		**Sites**	**Model**	**Ratio dN/dS**	**Sites**	**Model**	**Ratio dN/dS**
ORF1a	SLAC	44	GTR	0.154			
	FEL	227	GTR	0.12	1	GTR	0.12
	MEME				36	GTR	0.132
	FUBAR	514	GTR		1	GTR	
ORF1b	SLAC	42	GTR	0.0509	0	GTR	0.0509
	FEL	173	GTR	0.0464	1	GTR	0.0464
	MEME				13	GTR	0.0409
	FUBAR	433	GTR		3	GTR	
S	SLAC	117	GTR	0.126			
	FEL	381	GTR	0.121			
	MEME				14	GTR	0.0982
	FUBAR	604	GTR		1	GTR	
ORF3a	SLAC	8	GTR	0.231			
	FEL	33	GTR	0.191			
	MEME				4	GTR	0.206
	FUBAR	27	GTR				
ORF3b *	SLAC						
	FEL						
	MEME						
	FUBAR						
E	SLAC	0	GTR	0.00			
	FEL	0	GTR	0.00			
	MEME				0	GTR	0.00
	FUBAR	0	GTR				
M	SLAC	9	GTR	0.0932			
	FEL	21	GTR	0.121			
	MEME				0	GTR	0.0831
	FUBAR	22	GTR				
ORF6	SLAC	0	GTR	0.109			
	FEL	0	GTR	0.101			
	MEME				0	GTR	0.101
	FUBAR	0	GTR				
ORF7a	SLAC	2	GTR	0.0889			
	FEL	9	GTR	0.0770			
	MEME				0	GTR	0.0770
	FUBAR	10	GTR				
ORF7b	SLAC	0	GTR	0.506			
	FEL	1	GTR	0.493			

ORF7a	SLAC	2	GTR	0.0889			
	FEL	9	GTR	0.0770			
	MEME				0	GTR	0.0770
	FUBAR	10	GTR				
ORF7b	SLAC	0	GTR	0.506			
	FEL	1	GTR	0.493			
	MEME				0	GTR	0.493
	FUBAR	0	GTR				
ORF8 ***	SLAC						
	FEL						
	MEME						
	FUBAR						
N	SLAC	5	GTR	0.232			
	FEL	14	GTR	0.175			
	MEME				1	GTR	0.198
	FUBAR	14	GTR				
ORF9b **	SLAC						
	FEL						
	MEME						
	FUBAR						
ORF9c	SLAC	0	GTR	2.61			
	FEL	0	GTR	1.47			
	MEME				0	GTR	2.82
	FUBAR	0	GTR				

Mutation
No sites
Cleave

* Reported mutation: (343C => 343T; 344T => 344A) in (all virus found in bats).
** Reported mutacion: 55C => 55T in (I1-PL-CHA, I2-PL-CHA)
*** In Human ORF8 is cleaved into ORF8a and ORF8b.

Negative/Purifyng										
**Gene**	**Gene codon position**	**SLAC**		**FEL**		**FUBAR**		**Inferred substitution**			**Amino acid**
		**dN-dS**	**P[dN/dS < 1]**	**dN/dS**	**p-value**	**dN-dS**	**Prob[dS>dN]**	**Bat**	**Intermediate**	**Human**	
**ORF1a**	81	-24.13930956912419	0.0249922180952569	0.0	0.0069	-12.026	0.998				
	106	-28.23057797041663	0.0123456790123455	0.0	0.0003	-35.931	1.0				
	294	-30.6732163159245	0.0142021972305495	0.0	0.0009	-29.353	0.999				
	309	-21.17293347781247	0.0370370370370369	0.0	0.0011	-24.544	1.0				
	335	-24.49840948305111	0.0239092342237452	0.0	0.0028	-17.391	0.998				
	348	-24.85926234098167	0.0228830875624109	0.0	0.002	-18.15	0.998				
	514	-21.17293347781247	0.042082292077243	0.011	0.0021	-24.752	0.999				
	596	-22.57701556468167	0.0305477858762903	0.0	0.0019	-18.984	0.999				
	616	-24.62538777943012	0.0386345839618453	0.0	0.0019	-30.098	0.999				
	799	-24.41406181347019	0.0276712834909069	0.0	0.0154	-9.872	0.996				
	836	-21.17293347781247	0.0370370370370369	0.0	0.0105	-10.409	0.997				
	1153	-24.62064808986355	0.0271870322199383	0.0	0.0025	-19.896	0.998				
	1808	-24.36146300694938	0.024314718006546	0.0	0.0101	-9.589	0.997				
	2126	-28.61492674472402	0.0433068238730766	0.0	0.0013	-30.205	0.999				
	2140	-30.00053096297545	0.0375791909299058	0.0	0.0027	-27.899	0.998				
	2145	-25.46026591891944	0.0213005358507094	0.0	0.0029	-16.543	0.998				
	2161	-21.17293347781247	0.0370370370370369	0.0	0.002	-19.491	0.999				
	2274	-24.41406181347019	0.0241579020174801	0.0	0.0115	-9.882	0.995				
	2275	-21.17293347781247	0.0386529158132706	0.0	0.001	-27.15	0.999				
	2613	-21.17293347781247	0.0403316287846334	0.0	0.0034	-22.006	0.999				
	2780	-23.87364326045715	0.028993666147896	0.0	0.0052	-15.794	0.997				
	2938	-21.17293347781247	0.0370370370370369	0.0	0.0038	-15.632	0.999				
	3099	-21.17293347781247	0.0411040127002626	0.0	0.0024	-25.245	0.999				
	3179	-20.35800177440919	0.0416652403351465	0.0	0.0038	-17.13	0.999				
	3200	-25.94100523742966	0.0201381202954527	0.0	0.0008	-23.662	0.999				
	3233	-21.17293347781247	0.0370370370370369	0.0	0.0011	-25.147	0.999				
	3374	-25.77774615646029	0.0205231730362016	0.0	0.0008	-22.788	0.999				
	3394	-25.64933023463363	0.0208329728341989	0.0	0.0028	-17.728	0.998				
	3405	-24.20058601378715	0.0248028557491637	0.0	0.001	-20.828	0.999				
	3573	-21.17293347781247	0.0370370370370369	0.0	0.0052	-15.643	0.998				
	3726	-21.17293347781247	0.0370370370370369	0.0	0.0032	-17.619	0.999				
	3751	-28.23057797041663	0.0123456790123455	0.0	0.0047	-15.785	0.998				
	3765	-21.17293347781247	0.0370370370370369	0.0	0.0169	-9.077	0.996				
	3771	-23.8450973506852	0.0290658386120159	0.0	0.0041	-17.038	0.998				
	3887	-27.55131651229303	0.0195977594951293	0.0	0.0027	-24.674	0.999				
	4048	-21.17293347781247	0.0370370370370369	0.12	0.0049	-13.667	0.999				
	4066	-21.17293347781247	0.0370370370370369	0.0	0.0107	-10.093	0.998				
	4131	-28.23057797041663	0.0126046042838681	0.0	0.0007	-27.477	1.0				
	4184	-24.15077408226862	0.0283052603386562	0.0	0.0151	-11.067	0.997				
	4210	-30.70203023835273	0.0152175665562375	0.0	0.0006	-29.375	0.999				
	4246	-21.17293347781247	0.0370370370370369	0.0	0.0113	-10.446	0.998				
	4249	-21.17293347781247	0.0370370370370369	0.0	0.0009	-24.619	0.999				
	4292	-21.17293347781247	0.0370370370370369	0.0	0.0047	-14.117	0.998				
	4362	-26.18536658912956	0.0195795794801583	0.0	0.0004	-26.277	0.999				
**ORF1b**	67	-30.27268101368192	0.0201549634987746	0.0	0.0009	-34.266	1.0				
	88	-25.70625740897184	0.0282865795516011	0.0	0.0059	-17.6	0.999				
	161	-26.50204459549228	0.0289768171617613	0.0	0.0025	-25.122	1.0				
	340	-25.84470834672087	0.027834414128267	0.0	0.0024	-22.03	0.999				
	581	-25.3898210243624	0.0293574326207265	0.0	0.011	-14.494	0.999				
	783	-34.72779505333509	0.008177905644727	0.0	0.0002	-31.929	1.0				
	790	-23.49742390350566	0.0370370370370369	0.0	0.0122	-13.272	0.999				
	792	-31.32989853800754	0.0123456790123455	0.0	0.001	-26.813	1.0				
	873	-23.49742390350566	0.0370370370370369	0.0	0.0036	-19.416	0.999				
	882	-26.50204459549228	0.0278365387742216	0.0	0.001	-25.043	1.0				

	888	-25.32321015416528	0.0295897103410239	0.0	0.0108	-15.452	0.999
	908	-23.49742390350566	0.0370370370370369	0.0	0.0024	-23.586	1.0
	1264	-39.16237317250943	0.004115226337448	0.0	0.0	-38.722	1.0
	1601	-25.3898210243624	0.0293574326207265	0.0	0.0119	-14.51	0.998
	1654	-33.71133311115516	0.0101307691371166	0.0	0.0027	-19.731	1.0
	1817	-23.49742390350566	0.0370370370370369	0.0	0.003	-22.429	1.0
	1818	-25.70762267191753	0.0308599302238412	0.11	0.0091	-17.905	0.999
	1860	-23.49742390350566	0.0370370370370369	0.0	0.0027	-31.948	1.0
	1867	-25.42581145806849	0.0292329419825566	0.0	0.0111	-13.258	0.999
	1872	-25.86526221610855	0.0277681108674575	0.0	0.0052	-19.013	0.999
	1955	-26.5080868059447	0.0289630896898704	0.0	0.0023	-25.384	1.0
	1975	-25.32872673772763	0.0295703806808738	0.0	0.0104	-15.156	0.999
	1980	-26.34276890024118	0.0262852871358866	0.0	0.0021	-22.251	1.0
	1992	-26.10521365715158	0.0270094185657225	0.0	0.0016	-24.04	0.999
	1999	-34.19545504320532	0.008699161338293	0.0	0.002	-22.332	1.0
	2004	-23.49742390350566	0.0370370370370369	0.0	0.0057	-16.734	0.999
	2024	-23.49742390350566	0.0370370370370369	0.0	0.0051	-27.309	1.0
	2058	-25.87902685087278	0.0277238262063395	0.0	0.0037	-19.647	0.999
	2089	-23.49742390350566	0.0370370370370369	0.0	0.0034	-19.256	1.0
	2160	-25.88478828554248	0.0277053180236317	0.15	0.0037	-19.126	1.0
	2278	-23.49742390350566	0.0370370370370369	0.0	0.0062	-20.474	1.0
	2281	-23.49742390350566	0.0370370370370369	0.0	0.0141	-12.243	0.999
	2340	-23.49742390350566	0.0370370370370369	0.0	0.0032	-27.917	1.0
	2455	-25.3653592389788	0.029442449511548	0.0	0.012	-14.27	0.999
	2538	-23.49742390350566	0.0374471149236845	0.0	0.0017	-29.352	1.0
	2550	-34.18285225949904	0.0087119975163445	0.0	0.0025	-22.229	1.0
	2567	-24.21582516679137	0.0338375705247668	0.0	0.005	-18.002	0.999
	2585	-25.25755856262448	0.0318958991631839	0.0	0.0011	-31.565	1.0
	2589	-31.32989853800754	0.0123456790123455	0.0	0.0003	-37.081	1.0
	2603	-23.49742390350566	0.0370370370370369	0.0	0.0149	-12.272	0.999
	2605	-26.16836876050775	0.0311027499794406	0.0	0.0028	-28.651	1.0
	2613	-25.25755856262448	0.0318958991631839	0.0	0.0011	-31.565	1.0
**S**	19	-5.630145871312018	0.01675830233598	0.0	0.001	-5.331	0.998
	38	-5.385365949005194	0.0219546763394653	0.0	0.0003	-7.974	0.999
	40	-4.040451853322936	0.0370370370370372	0.0	0.0007	-8.101	0.999
	41	-5.612856555118544	0.0185395827222728	0.0	0.0021	-6.378	0.998
	53	-7.166106046610601	0.0126872567523189	0.0	0.0042	-9.876	0.997
	59	-5.798459090195722	0.0125310727570185	0.0	0.0039	-4.455	0.997
	60	-4.368686229835476	0.0377940103411511	0.0	0.002	-7.492	1.0
	84	-4.18459493115358	0.0460392240277662	0.0	0.0018	-6.516	0.997
	85	-4.040451853322936	0.0370370370370372	0.0	0.0007	-7.318	0.999
	91	-5.791493806045795	0.0125763395741376	0.0	0.0018	-6.415	0.997
	96	-4.040451853322936	0.0370370370370372	0.0	0.0002	-9.239	1.0
	119	-4.040451853322936	0.0454445736123716	0.0	0.0066	-5.767	0.999
	124	-5.630145871312018	0.01368885434678	0.0	0.0011	-5.312	0.998
	134	-5.798459090195722	0.015607775398049	0.0	0.0033	-4.512	0.996
	162	-4.040451853322936	0.0370370370370372	0.0	0.0003	-9.733	0.999
	163	-7.075693344103648	0.0125286259203666	0.0	0.0036	-12.235	0.997
	194	-5.258470735237914	0.0380277948505523	0.0	0.0018	-7.828	0.998
	203	-5.798459090195722	0.0172503800839887	0.0	0.0061	-4.55	0.995
	213	-5.600024827046981	0.0173220010940093	0.0	0.0001	-13.536	1.0
	218	-4.040451853322936	0.0370370370370372	0.0	0.0022	-6.466	0.998
	229	-4.040451853322936	0.0370370370370372	0.14	0.0044	-4.187	0.998
	264	-4.777887593988988	0.0460625746001091	0.0	0.001	-10.937	0.999
	268	-4.318733687490878	0.0432237055292649	0.0	0.0013	-6.604	0.998
	278	-4.424076854473581	0.0282135707953883	0.0	0.0036	-4.191	0.996
	307	-5.4612941184404	0.047857695571134	0.0	0.0043	-7.903	0.997
	313	-4.040451853322936	0.0370370370370372	0.0	0.0004	-12.081	0.999
	323	-4.040451853322936	0.0370370370370372	0.0	0.0006	-8.062	0.999
	331	-4.415891503446125	0.0413426257092989	0.0	0.0	-20.672	0.999
	343	-4.040451853322936	0.0370370370370372	0.0	0.0014	-9.103	0.999
	345	-4.040451853322936	0.0370370370370372	0.0	0.001	-9.855	0.999

347	-8.225294857218953	0.0055228102459768	0.0	0.0002	-14.611	1.0
348	-5.958249543643965	0.0160738610523705	0.0	0.0006	-9.721	1.0
367	-4.366139852749114	0.0422901825004463	0.0	0.0006	-8.885	0.999
370	-4.317547107659707	0.0432474669028659	0.0	0.0016	-7.607	0.998
372	-6.311981275905117	0.0126877733336621	0.0	0.0003	-9.997	0.999
396	-5.726057318611495	0.0446047034759913	0.0	0.0027	-15.14	0.999
415	-4.187280767355624	0.0459801813392007	0.0	0.0017	-5.994	0.997
416	-5.742648790570871	0.0176251202710984	0.0	0.0066	-4.623	0.995
417	-5.975117731070752	0.029452836820057	0.0	0.0004	-14.438	0.999
420	-4.317547107659707	0.0432474669028659	0.0	0.0013	-7.752	0.997
421	-4.187280767355624	0.0459801813392007	0.011	0.0016	-6.023	0.997
422	-6.302996269210748	0.0097562943288611	0.0	0.0002	-9.951	1.0
426	-5.387269137763916	0.0123456790123457	0.0	0.0002	-10.266	1.0
428	-5.387269137763916	0.0123456790123457	0.0	0.0013	-6.111	0.999
430	-7.617075485184021	0.0030891331907974	0.0	0.0001	-10.921	1.0
450	-8.002038803820065	0.0107681033902549	0.0	0.0	-40.216	1.0
453	-5.576635196963982	0.0338123685447533	0.0	0.0029	-8.923	0.998
467	-5.387269137763916	0.0128317352749536	0.0	0.0002	-13.141	1.0
504	-4.040451853322936	0.0370370370370372	0.1	0.0018	-7.317	0.999
508	-5.482797435187623	0.03125283688189	0.0	0.0009	-10.634	0.998
514	-4.040451853322936	0.0370370370370372	0.0	0.001	-7.502	0.999
521	-4.406260720359506	0.0415949981998187	0.0	0.0004	-10.95	1.0
533	-5.687932238756619	0.0132758642228597	0.0	0.0017	-5.7	0.999
534	-6.442572256777162	0.0091358329451483	0.0	0.0001	-12.363	1.0
547	-4.040451853322936	0.038249503780487	0.0	0.0005	-11.604	1.0
558	-4.040451853322936	0.0392035285084597	0.0	0.001	-6.807	0.999
559	-5.957761461935266	0.0327244747850574	0.0	0.0	-30.382	1.0
560	-5.723184756642842	0.0130320502413355	0.0	0.003	-5.622	0.996
564	-4.986136345252022	0.0461097052850822	0.0	0.0142	-7.206	0.991
566	-5.929060552096182	0.0117211004191015	0.0	0.0001	-12.225	0.999
567	-4.040451853322936	0.038137893384568	0.0	0.0062	-5.463	0.998
568	-4.040451853322936	0.0370370370370372	0.0	0.0013	-11.206	0.999
571	-4.040451853322936	0.0370370370370372	0.0	0.0036	-5.752	0.999
590	-4.220369049623882	0.0452620255634994	0.0	0.0066	-4.943	0.996
598	-5.387269137763916	0.0123456790123457	0.0	0.0011	-6.947	0.999
608	-5.473902895680864	0.0148948034587588	0.1	0.0002	-11.451	0.999
612	-4.040451853322936	0.0370370370370372	0.0	0.0121	-3.765	0.993
616	-4.220369049623882	0.0452620255634994	0.0	0.0066	-4.943	0.996
635	-4.2077897398893	0.0455330543651713	0.0	0.0066	-4.835	0.995
639	-4.040451853322936	0.0370370370370372	0.0	0.0018	-7.564	0.999
649	-5.641096272905633	0.0341019015142975	0.0	0.014	-8.551	0.984
651	-5.387269137763916	0.0125266840123308	0.0	0.0005	-13.425	1.0
656	-4.43968142990423	0.0279171203496283	0.0	0.0093	-3.623	0.994
695	-5.577513879360104	0.0187995118063704	0.0	0.0022	-5.796	0.998
698	-5.577513879360104	0.0140800457138874	0.0	0.0011	-5.725	0.998
733	-4.186141231429783	0.0460052178571526	0.0	0.0017	-6.02	0.997
739	-5.770343856085113	0.0127151346315354	0.0	0.0006	-8.304	0.999
743	-6.974617331736501	0.0169639504676656	0.0	0.0004	-14.552	1.0
746	-4.237963990369726	0.0448869730754419	0.0	0.0017	-6.958	0.997
754	-4.040451853322936	0.0370370370370372	0.0	0.0004	-10.299	1.0
755	-4.040451853322936	0.0370370370370372	0.0	0.0046	-4.428	0.998
768	-4.489016732927984	0.0466220535244767	0.0	0.0068	-5.65	0.996
770	-4.162639553922778	0.0465261622870812	0.0	0.0349	-3.529	0.979
808	-5.539661508864795	0.0143706481944534	0.0	0.0002	-9.727	0.999
821	-4.162639553922778	0.0465261622870812	0.0	0.0349	-3.529	0.979
824	-9.127662877708918	0.0046964256036532	0.0	0.0	-21.27	1.0
836	-5.744078419805892	0.0128903575149268	0.11	0.0035	-4.465	0.995
838	-4.432155426024034	0.0280595756369413	0.0	0.0047	-4.313	0.997
840	-4.040451853322936	0.0370370370370372	0.0	0.0009	-8.39	1.0
841	-8.220763088265763	0.0103598090813434	0.0	0.0003	-15.612	1.0
849	-5.047972566927641	0.0267800270619289	0.0	0.0001	-18.876	1.0
854	-4.040451853322936	0.0370370370370372	0.0	0.0003	-10.506	1.0

	873	-4.040451853322936	0.0386537802238141	0.0	0.0045	-4.981	0.997
	875	-5.387269137763916	0.0123456790123457	0.0	0.0006	-11.22	1.0
	894	-6.26192886781483	0.0099495091100513	0.0	0.0002	-9.351	1.0
	902	-4.352076910556822	0.0425639300815249	0.0	0.0002	-10.433	0.999
	911	-4.416599537641493	0.0283571105253719	0.0	0.0037	-4.425	0.998
	913	-6.281335530215974	0.0098575744466669	0.0	0.0005	-7.735	0.999
	918	-4.040451853322936	0.0370370370370372	0.0	0.0027	-6.476	0.999
	948	-4.179154056645206	0.0461591797397834	0.0	0.0054	-4.914	0.995
	953	-5.479777062725202	0.0475353980239852	0.0	0.0012	-10.647	0.998
	973	-5.721853449816913	0.0130411488811355	0.0	0.0015	-6.254	0.998
	990	-5.89003350668799	0.0411440827037985	0.0	0.0009	-12.291	0.998
	1036	-4.162639553922778	0.0465261622870812	0.0	0.0308	-3.533	0.979
	1037	-4.047973659799387	0.0406766247365907	0.0	0.0016	-10.035	0.999
	1043	-4.040451853322936	0.0370370370370372	0.0	0.0015	-6.216	0.999
	1106	-4.2077897398893	0.0455330543651713	0.0	0.0055	-4.911	0.994
	1136	-8.273958034243842	0.0022188932874782	0.0	0.0001	-12.917	1.0
	1150	-4.040451853322936	0.0370370370370372	0.0	0.0001	-15.4	0.999
	1154	-4.714352809245437	0.0452533344703988	0.0	0.0123	-7.836	0.995
	1166	-4.2077897398893	0.0455330543651713	0.0	0.0057	-4.888	0.994
	1175	-4.398636677805785	0.0416676168474746	0.0	0.0001	-14.725	0.999
	1201	-4.040451853322936	0.0370370370370372	0.0	0.0001	-13.936	1.0
	1208	-9.308074364547156	0.0008156201244766	0.0	0.0	-21.243	1.0
	1247	-5.651669125875856	0.0135330553713021	0.0	0.0014	-7.07	0.998
	1251	-4.040451853322936	0.0370370370370372	0.0	0.0001	-15.754	1.0
	1254	-8.340366775827556	0.00627261201839	0.0	0.0001	-21.654	1.0
**ORF3a**	9	-8.321777514547437	0.0370370370370369	0.0	0.0012	-19.434	0.998
	71	-9.6133463625681	0.0219096767545208	0.0	0.0031	-10.598	0.998
	88	-8.321777514547437	0.0370370370370369	0.0	0.0032	-16.285	0.998
	91	-11.13617676544641	0.020124909406711	0.0	0.0044	-15.627	0.99
	95	-8.577097736364697	0.0490130479341243	0.0	0.0033	-15.274	0.999
	130	-11.11437856608503	0.0186922074333985	0.1	0.0029	-14.69	0.991
	160	-15.12737686014967	0.0051843950950546	0.0	0.0007	-21.641	0.999
	239	-12.11138960711901	0.0120144145404246	0.0	0.0005	-22.134	0.999
**M**	2	-15.8111144527264	0.0370370370370372	0.0	0.0014	-29.844	1.0
	52	-19.12680217658693	0.020921681427046	0.0	0.0019	-19.842	0.997
	72	-16.63439765248334	0.0318055159661155	0.0	0.0044	-20.701	0.998
	77	-15.8111144527264	0.0370370370370372	0.0	0.0281	-15.406	0.992
	86	-18.156218761015	0.024459493672834	0.0	0.0054	-18.677	0.998
	104	-15.8111144527264	0.0370370370370372	0.0	0.0161	-13.243	0.995
	112	-19.43751910879265	0.0199343088730514	0.0	0.0067	-16.412	0.996
	124	-19.01300821628886	0.0232472230141476	0.0	0.0038	-33.022	0.999
	140	-15.8111144527264	0.0391082343283916	0.0	0.0208	-16.982	0.994
**ORF7a**	27	-19.67826996083158	0.0370370370370369	0.0	0.0025	-33.543	0.999
	51	-23.59921303041497	0.0214735781225587	0.0	0.0036	-30.855	0.998
**N**	141	-33.60907775569749	0.0233477181976282	0.0	0.0092	-22.54	0.996
	143	-38.42360134334388	0.0123456790123455	0.10	0.0008	-35.888	1.0
	145	-33.63861822759213	0.0232862623040994	0.0	0.012	-21.053	0.995
	157	-28.81770100750791	0.0370370370370369	0.0	0.021	-17.98	0.994
	277	-31.6385014563747	0.0311380529259446	0.0	0.0008	-39.248	1.0

MERS-CoV: Natural selection and inferred substitutions in the predicted CDSs for MERS-CoV

CDS	Test	Negative/Purifyng			Positive/Diversifying		
		Sites	Model	Ratio dN/dS	Sites	Model	Ratio dN/dS
ORF1a	SLAC	65	GTR	0.164	1	GTR	0.164
	FEL	495	GTR	0.133	3	GTR	0.133
	MEME				32	GTR	0.133
	FUBAR	1064	GTR		9	GTR	
ORF1b	SLAC	29	GTR	0.0754	1	GTR	0.0754
	FEL	190	GTR	0.0580	2	GTR	0.0580
	MEME				12	GTR	0.0580
	FUBAR	857	GTR		4	GTR	
S	SLAC	31	GTR	0.173	1	GTR	0.173
	FEL	183	GTR	0.114	2	GTR	0.114
	MEME				10	GTR	0.114
	FUBAR	137	GTR		4	GTR	
ORF3	SLAC	0	GTR	0.366			
	FEL	7	GTR	0.346			
	MEME				0	GTR	0.346
	FUBAR	1	GTR		1	GTR	
ORF4a	SLAC	0	GTR	0.270			
	FEL	**10**	GTR	0.185			
	MEME				1	GTR	0.185
	FUBAR	4	GTR		1	GTR	
ORF4b	SLAC	2	GTR	0.281			
	FEL	29	GTR	0.223			
	MEME				0	GTR	0.223
	FUBAR	10	GTR		1	GTR	
ORF5	SLAC	2	GTR	0.207			
	FEL	22	GTR	0.163			
	MEME				0	GTR	0.163
	FUBAR	12	GTR		2	GTR	
E	SLAC	0	GTR	0.209			
	FEL	6	GTR	0.199			
	MEME				0	GTR	0.199
	FUBAR	0	GTR				
M	SLAC	2	GTR	0.0575			
	FEL	37	GTR	0.0367			
	MEME				2	GTR	0.0367
	FUBAR	12	GTR		1	GTR	
ORF8b	SLAC	0	GTR	3.09			
	FEL	0	GTR	3.24			
	MEME				0	GTR	3.24
	FUBAR	0	GTR				

N	SLAC	7	GTR	0.171	1	GTR	0.171
	FEL	38	GTR	0.143	1	GTR	0.143
	MEME				1	GTR	0.143
	FUBAR	21	GTR		1	GTR	

	Positive
	No sites

Negative/Purifyng										
**Gene**	**Gene codon positio**	**SLAC**		**FEL**		**FUBAR**		**Inferred substitution**			**Amino acid**
		**dN-dS**	**P[dN/dS < 1]**	**dN/dS**	**p-value**	**dN-dS**	**Prob[dS>dN]**	**Bat**	**Intermediate**	**Human**	
**ORF1a**	47	-1.34	0.00045	0.0	0.0	-47.93	1.0	CCA	CCA, CCG	CCA	P
	88	-115.47	0.0013	0.0	0.0	-44.53	1.0	GTC, GTT	GTG, GTA	GTG, GTA	V
	103	-602.34	0.045	0.0	0.007	-4.366	0.984	TTT	TTC	TTC, TTT	F
	118	-1.13	0.0017	0.0	0.0	-46.699	1.0	CTC, CTT	CTG, TTG, CT	CTG, TTG, CTT	L
	132	-6.02	0.045	0.0	0.007	-4.37	0.984	TTT	TTT, TTC	TTC	F
	175	-6.12	0.043	0.0	0.004	-5.945	0.982	GAC	GAT	GAT, GAC	D
	301	-577.37	0.037	0.0	0.001	-6.926	0.991	CGT	CGC, CGT	CGC, CGT	R
	320	-9.03	0.0096	0.0	0.001	-10.951	0.993	ACA	ACC	ACC	T
	334	-6.02	0.045	0.0	0.005	-4.354	0.987	CAT	CAC	CAT, CAC	H
	370	-7.69	0.012	0.0	0.001	-20.344	0.996	GTT	GTC, GTT	GTC, GTT	V
	413	-7.74	0.033	0.0	0.002	-9.541	0.987	GAG	GAA	GAA, GAG	E
	414	-577.37	0.037	0.0	0.0	-19.819	0.997	CGT	CGT, CGC	CGT, CGC	R
	431	-577.37	0.037	0.0	0.001	-26.006	0.996	GTA	GTG, GTA	GTG, GTA	V
	444	-1.40	0.0010	0.0	0.0	-43.962	1.0	TAC	TAT, TAC	TAT, TAC	Y
	471	-7.69	0.012	0.0	0.001	-18.804	0.995	GTT	GTC, GTT	GTC, GTT	V
	669	-9.03	0.0096	0.0	0.001	-11.086	0.993	AAT, AAC	AAT, AAC	AAC	N
	733	-8.78	0.0072	0.0	0.001	-22.138	0.998	ATT, ATA	ATC	ATC, ATT	I
	834	-7.70	0.049	0.029	0.001	-38.791	0.995	ACT I	TTT, TTC	TTC, TTT	T I F
	864	-577.37	0.037	0.0	0.001	-14.845	0.993	GGC	GGC	GGC, GGA, GGT	G
	949	-9.62	0.0041	0.0	0.0	-40.53	1.0	GCA	GCT, GCA	GCT	A
	1323	-602.36	0.045	0.0	0.007	-4.403	0.981	GAT	GAC	GAT, GAC	D
	1402	-6.64	0.044	0.0	0.0	-20.142	0.994	TGT	TGT	TGC, TGT	C
	1429	-120.76	0.0020	0.0	0.0	-27.903	0.999	CAT	CAC	CAC, CAT	H
	1568	-1.54	0.0031	0.005	0.0	-46.063	1.0	CAG I	GAG, GAA	GAG	Q I E
	1669	-6.12	0.043	0.0	0.003	-5.79	0.988	CAC	CAC, CAT	CAT	H
	1800	-1.20	0.0020	0.0	0.0	-27.036	0.998	GAT	GAT, GAC	GAC	D
	1827	-6.02	0.045	0.0	0.007	-4.409	0.981	GAT	GAT, GAC	GAC	D
	1915	-7.69	0.012	0.0	0.001	-19.261	0.996	GGT	GGT, GGC	GGT, GGC	G
	1944	-976.23	0.0076	0.0	0.0	-26.7	0.998	AAT	AAT, AAC	AAT, AAC	N
	2018	-9.03	0.0096	0.0	0.001	-10.989	0.993	TTT	TTC	TTC, TTT	F
	2035	-6.14	0.043	0.0	0.003	-7.01	0.983	GAT	GAT, GAC	GAT, GAC	D
	2083	-1.90	0.00024	0.0	0.0	-45.551	1.0	AAA	AAA, AAG	AAA, AAG	K
	2258	-9.62	0.0050	0.0	0.0	-36.355	1.0	TCG	TCA, TCC	TCA, TCC	S
	2275	-1.20	0.0028	0.0	0.0	-26.385	0.998	TGT	TGC, TGT	TGC	C
	2280	-6.12	0.043	0.0	0.004	-5.838	0.982	GAC	GAT	GAT, GAC	D
	2284	-1.09	0.0067	0.012	0.0	-34.856	0.998	CGT I	CAC, CAT	CAC	R I H
	2299	-1.34	0.00045	0.0	0.0	-48.115	1.0	GTT	GTG, GTT	GTG, GTT	V
	2358	-181.04	0.0000092	0.0	0.0	-44.109	1.0	TTT	TTT, TTC	TTC, TTT	F
	2644	-6.02	0.045	0.0	0.007	-4.405	0.981	GAT	GAC, GAT	GAC	D
	2972	-9.17	0.0092	0.0	0.0	-18.368	0.996	AGC	AGT, AGC	AGT, AGC	S
	2992	-9.97	0.011	0.0	0.0	-37.526	0.999	TAT	TAT, TAC	TAT	Y
	3021	-9.62	0.0041	0.0	0.0	-40.176	1.0	GGC, GGA	GGC, GGT	GGC, GGT	G
	3036	-6.02	0.0453	0.0	0.007	-4.367	0.984	TTT	TTC	TTT, TTC	F
	3056	-1.25	0.0025	0.015	0.0	-45.329	1.0	GGC I	GCG, GCA	GCG, GCA	G I A
	3171	-6.61	0.037	0.0	0.0	-17.431	0.992	GAT	GAC, GAT	GAT, GAC	D
	3243	-6.02	0.045	0.0	0.005	-4.374	0.986	AGT	AGC	AGC, AGT	S
	3376	-577.37	0.037	0.0	0.001	-14.85	0.993	GGC	GGC, GGT	GGC	G
	3476	-903.92	0.014	0.0	0.001	-11.298	0.99	TAT	TAC, TAT	TAC, TAT	Y
	3618	-7.34	0.015	0.0	0.0	-29.756	0.999	CTA	CTC, CTT	CTC	L
	3625	-819.00	0.036	0.03	0.017	-13.807	0.955	TGT I	TTC, TTT	TTC, TTT	C I F
	3674	-7.69	0.012	0.0	0.0	-18.538	0.998	CCT	CCC	CCT, CCC	P
	3716	-9.03	0.012	0.0	0.0	-11.006	0.992	TGT	TGC, TGT	TGC, TGT	C
	3795	-9.04	0.012	0.0	0.0	-11.313	0.992	TGT	TGC	TGC, TGT	C
	3875	-9.80	0.012	0.0	0.0	-40.415	1.0	TTA	CTC, CTA	CTC, CTA	L
	3900	-6.12	0.043	0.0	0.004	-5.936	0.982	GAC	GAT	GAT, GAC	D
	3907	-115.47	0.0013	0.0	0.0	-40.101	1.0	CCT	CCT, CCC	CCC	P

	3956	-1.07	0.0072	0.024	0.003	-32.256	0.995	AGT |	ACC, ACG, A	ACC, ACG	S | T
	3959	-7.50	0.035	0.0	0.003	-8.803	0.986	GAA	GAG, GAA	GAG	E
	4024	-8.39	0.012	0.0	0.0	-24.954	0.997	GAC, GAT	GAC, GAT	GAC, GAT	D
	4045	-6.02	0.045	0.0	0.007	-4.417	0.981	GAT	GAC	GAC, GAT	D
	4068	-5.59	0.044	0.0	0.002	-19.444	0.997	CTT	CTG, TTG	CTG	L
	4094	-1.50	0.00043	0.0	0.0	-38.925	1.0	AAT	AAT, AAC	AAT, AAC	N
	4107	-6.12	0.043	0.0	0.004	-5.901	0.985	AAC	AAT	AAT, AAC	N
	4245	-7.69	0.012	0.0	0.0	-32.988	0.999	GGT	GGG	GGG, GGT	G
	4284	-6.02	0.045	0.0	0.007	-4.358	0.984	TTT	TTC	TTC, TTT	F
**ORF1b**	69	-1.37	0.016	0.0	0.002	-13.555	0.999	ATA	ATC, ATT	ATC, ATT	I
	175	-1.17	0.040	0.0	0.002	-24.765	1.0	TTA	CTG, TTG	CTG, TTG	L
	359	-3.17	0.0000050	0.0	0.0	-49.12	1.0	GTG	GTG, GTT	GTG, GTT	V
	533	-2.81	0.00015	0.0	0.0	-48.006	1.0	GTC	GTT, GTC	GTT	V
	557	-241.81	0.00084	0.0	0.0	-37.041	1.0	TTT	TTT, TTC	TTC	F
	651	-1.11	0.044	0.0	0.0	-36.513	1.0	TTG	TTG, TTA	TTG, TTA	L
	684	-114.86	0.041	0.0	0.0	-19.561	0.999	GAT	GAT	GAT, GAC	D
	736	-1.44	0.014	0.0	0.001	-12.595	0.999	TTT	TTT, TTC	TTT, TTC	F
	845	-2.75	0.00083	0.0	0.0	-44.319	1.0	TAT	TAC, TAT	TAC, TAT	Y
	1017	-176.13	0.0041	0.0	0.0	-30.298	1.0	CGT	CGC, CGT	CGC	R
	1065	-1.40	0.012	0.0	0.001	-31.087	1.0	GTC	GTA, GTG	GTA, GTG, GTT	V
	1146	-1.72	0.0085	0.0	0.0	-34.714	1.0	CAT	CAT, CAC	CAT, CAC	H
	1150	-1.40	0.012	0.0	0.0	-41.844	1.0	GGG, GGT	GGG	GGG, GGT	G
	1152	-1.05	0.037	0.0	0.003	-16.826	0.999	GCA	GCG	GCG, GCT	A
	1154	-1.44	0.019	0.0	0.002	-12.643	0.999	TAT	TAC	TAC, TAT	Y
	1308	-1.05	0.037	0.0	0.003	-18.997	0.999	GTA, GTT	GTG	GTG, GTT	V
	1383	-1.40	0.012	0.0	0.0	-37.855	1.0	GGT	GGT, GGC	GGT, GGC	G
	1399	-4.93	0.00015	0.0	0.0	-49.086	1.0	TAT	TAC, TAT	TAC, TAT	Y
	1451	-1.45	0.019	0.0	0.002	-12.932	0.999	TAT	TAC, TAT	TAC	Y
	1453	-176.13	0.0041	0.0	0.0	-32.453	1.0	CTT	CTT, CTC	CTT, CTC	L
	1612	-2.46	0.00045	0.0	0.0	-47.727	1.0	GCT	GCA, GCG	GCA, GCG	A
	1708	-1.05	0.037	0.0	0.0	-27.274	1.0	GCT	GCA, GCC, G	GCA, GCC, GCT	A
	1812	-2.79	0.0015	0.008	0.0	-47.231	1.0	AAA, AAG	AGA, AGG	AGA, AGG	K | R
	2051	-144.73	0.014	0.0	0.001	-12.432	0.999	CAT	CAC, CAT	CAC	H
	2120	-1.49	0.0097	0.0	0.0	-36.379	1.0	ATC	ATC, ATT	ATC, ATT	I
	2151	-1.45	0.014	0.0	0.001	-12.618	0.999	TTT	TTC, TTT	TTC, TTT	F
	2351	-2.41	0.0013	0.0	0.0	-36.759	1.0	TAT	TAC, TAT	TAC, TAT	Y
	2381	-1.05	0.037	0.0	0.003	-10.207	0.999	GCT	GCC	GCC, GCT	A
	2403	-2.11	0.0013	0.0	0.0	-45.099	1.0	ACT	ACA, ACC	ACA, ACC	T
**S**	90	-1.22	0.0015	0.01	0.0	-46.435	1.0	TTA |	GTT, GTC	GTT, GTC	L | V
	273	-4.39	0.044	0.0	0.002	-12.525	0.996	TCT	TCA, TCT	TCA, TCT	S
	299	-2.28	0.000000071	0.0	0.0	-48.955	1.0	TAC	TAT, TAC	TAT, TAC	Y
	319	-4.52	0.046	0.0	0.007	-3.837	0.983	TTT	TTC	TTC, TTT	F
	391	-4.60	0.044	0.0	0.007	-5.692	0.978	TTT, TTC	TTT	TTT	F
	451	-1.29	0.00026	0.0	0.0	-46.889	1.0	TAC	TAC, TAT	TAC, TAT	Y
	476	-6.87	0.026	0.0	0.001	-12.409	0.992	AAG	AAA	AAA, AAG	K
	483	-4.39	0.037	0.0	0.001	-13.115	0.997	ACT	ACT, ACA	ACT, ACA	T
	689	-7.32	0.019	0.07	0.002	-40.426	0.999	ACT |	TCT, TCC	TCT, TCC | TTC	T | S | F
	704	-8.20	0.036	0.003	0.0	-44.386	1.0	CGC |	AAA, AAG	AAA, AAG	R | K
	711	-8.78	0.0017	0.0	0.0	-40.116	1.0	GGA	GGT, GGC	GGT, GGC	G
	751	-450.26	0.043	0.0	0.0065	-28.693	0.959		CTC, CTT	CTC, CTT | TTC	L | F
	773	-5.85	0.012	0.0	0.0	-21.35	0.999	CCA	CCT, CCC	CCT, CCC	P
	777	-4.60	0.044	0.0	0.007	-5.811	0.974	GAC, GAT	GAT	GAT	D
	794	-4.60	0.044	0.0	0.007	-5.692	0.978	TTT, TTC	TTT	TTT	F
	817	-6.37	0.014	0.0	0.0	-16.533	0.998	TGC, TGT	TGC, TGT	TGC, TGT	C
	820	-4.52	0.046	0.0	0.007	-3.835	0.983	TTT	TTC	TTT, TTC	F
	909	-4.39	0.037	0.0	0.0	-12.863	0.997	CCC, CCA	CCT	CCC, CCT	P
	953	-182.09	0.000012	0.0	0.0	-47.405	1.0	TAC	TAC, TAT	TAC, TAT	Y
	954	-5.85	0.012	0.0	0.0	-26.197	1.0	ACA, ACG	ACC, ACT	ACC, ACT	T
	982	-1.32	0.0000056	0.0	0.0	-46.869	1.0	ATC	ATC, ATT	ATC, ATT	I
	1027	-6.66	0.028	0.0	0.002	-10.799	0.991	AAA	AAG	AAA, AAG	K
	1037	-6.66	0.036	0.0	0.002	-10.903	0.991	CAA	CAG	CAG, CAA	Q
	1046	-6.31	0.013	0.0	0.001	-23.184	1.0	TTG	CTA	CTA, TTA	L

<table>
<tr><td></td><td>1084</td><td>-5.25</td><td>0.027</td><td>0.0</td><td>0.001</td><td>-13.686</td><td>0.998</td><td>TTG</td><td>CTA, TTA</td><td>CTA, TTA</td><td>L</td></tr>
<tr><td></td><td>1087</td><td>-4.90</td><td>0.039</td><td>0.0</td><td>0.0</td><td>-17.954</td><td>0.996</td><td>TTT</td><td>TTT</td><td>TTT, TTC</td><td>F</td></tr>
<tr><td></td><td>1142</td><td>-1.50</td><td>0.000031</td><td>0.0</td><td>0.0</td><td>-47.643</td><td>1.0</td><td>TTC</td><td>TTT, TTC</td><td>TTT, TTC</td><td>F</td></tr>
<tr><td></td><td>1206</td><td>-7.33</td><td>0.040</td><td>0.03</td><td>0.001</td><td>-37.655</td><td>0.999</td><td>ATT</td><td>ATC</td><td>ATC, ATT</td><td>I</td></tr>
<tr><td></td><td>1237</td><td>-4.53</td><td>0.046</td><td>0.0</td><td>0.008</td><td>-3.885</td><td>0.979</td><td>GAT</td><td>GAC</td><td>GAC, GAT</td><td>D</td></tr>
<tr><td></td><td>1287</td><td>-6.79</td><td>0.014</td><td>0.0</td><td>0.002</td><td>-8.564</td><td>0.994</td><td>TAT</td><td>TAC, TAT</td><td>TAC</td><td>Y</td></tr>
<tr><td></td><td>1310</td><td>-6.79</td><td>0.0099</td><td>0.0</td><td>0.001</td><td>-8.522</td><td>0.996</td><td>TTT</td><td>TTC</td><td>TTC, TTT</td><td>F</td></tr>
<tr><td>ORF4b</td><td>132</td><td>-3.45</td><td>0.039</td><td>0.0</td><td>0.003</td><td>-12.222</td><td>0.97</td><td>TTT, TTC</td><td>TTT</td><td>TTT</td><td>F</td></tr>
<tr><td></td><td>231</td><td>-3.09</td><td>0.037</td><td>0.0</td><td>0.002</td><td>-14.667</td><td>0.993</td><td>GTT</td><td>GTC, GTT</td><td>GTC</td><td>V</td></tr>
<tr><td>ORF5</td><td>81</td><td>-4.58</td><td>0.046</td><td>0.0</td><td>0.003</td><td>-11.698</td><td>0.98</td><td>AAT</td><td>AAT, AAC</td><td>AAC</td><td>N</td></tr>
<tr><td></td><td>114</td><td>-4.64</td><td>0.045</td><td>0.0</td><td>0.002</td><td>-15.152</td><td>0.984</td><td>GAC</td><td>GAC, GAT</td><td>GAT</td><td>D</td></tr>
<tr><td>M</td><td>37</td><td>-584.02</td><td>0.042</td><td>0.0</td><td>0.001</td><td>-17.388</td><td>0.998</td><td>GGT, GGC</td><td>GGA, GGT</td><td>GGA</td><td>G</td></tr>
<tr><td></td><td>155</td><td>-6.23</td><td>0.043</td><td>0.0</td><td>0.003</td><td>-9.959</td><td>0.986</td><td>TTT</td><td>TTC, TTT</td><td>TTC</td><td>F</td></tr>
<tr><td>N</td><td>46</td><td>-8.32</td><td>0.037</td><td>0.0</td><td>0.002</td><td>-20.441</td><td>0.999</td><td>GGC, GGT</td><td>GGG</td><td>GGG, GGT</td><td>G</td></tr>
<tr><td></td><td>50</td><td>-845.44</td><td>0.047</td><td>0.0</td><td>0.001</td><td>-15.929</td><td>0.991</td><td>CAT, CAC</td><td>CAC, CAT</td><td>CAC</td><td>H</td></tr>
<tr><td></td><td>100</td><td>-8.93</td><td>0.042</td><td>0.0</td><td>0.004</td><td>-9.117</td><td>0.986</td><td>TTT</td><td>TTC</td><td>TTT, TTC</td><td>F</td></tr>
<tr><td></td><td>155</td><td>-162.22</td><td>0.0071</td><td>0.0</td><td>0.0</td><td>-29.584</td><td>1.0</td><td>AAA</td><td>AAA, AAG</td><td>AAA, AAG</td><td>K</td></tr>
<tr><td></td><td>195</td><td>-8.32</td><td>0.037</td><td>0.0</td><td>0.002</td><td>-15.334</td><td>0.997</td><td>AAT</td><td>AAC</td><td>AAC</td><td>N</td></tr>
<tr><td></td><td>263</td><td>-8.94</td><td>0.042</td><td>0.0</td><td>0.004</td><td>-9.238</td><td>0.986</td><td>TTT</td><td>TTT, TTC</td><td>TTC</td><td>F</td></tr>
<tr><td></td><td>397</td><td>-9.71</td><td>0.045</td><td>0.054</td><td>0.018</td><td>-29.618</td><td>0.99</td><td>GCG |</td><td>CCA, CCT</td><td>CCA, CCT</td><td>A | P</td></tr>
</table>

SARS-CoV-2: Natural selection and inferred substitutions in the predicted CDSs for SARS-CoV-2

CDS	Test	Negative/Purifyng			Positive/Diversifying		
		Sites	Model	Ratio dN/dS	Sites	Model	Ratio dN/dS
ORF1a	SLAC	35	GTR	0.136	1	GTR	0.136
	FEL	451	GTR	0.118	1	GTR	0.118
	MEME				47	GTR	0.118
	FUBAR	1182	GTR		1	GTR	
ORF1b	SLAC	15	GTR	0.0595	1	GTR	0.0595
	FEL	240	GTR	0.0464	1	GTR	0.0464
	MEME				13	GTR	0.0464
	FUBAR	719	GTR		2	GTR	
S	SLAC	20	GTR	0.173	1	GTR	0.173
	FEL	270	GTR	0.142	2	GTR	0.142
	MEME				23	GTR	0.142
	FUBAR	431	GTR		1	GTR	
ORF3a	SLAC	2	GTR	0.371			
	FEL	21	GTR	0.330			
	MEME				3	GTR	0.330
	FUBAR	16	GTR		1	GTR	
E	SLAC	0	GTR	0.300			
	FEL	1	GTR	0.288			
	MEME				0	GTR	0.288
	FUBAR	1	GTR				
M	SLAC	4	GTR	0.0527			
	FEL	24	GTR	0.0408			
	MEME				0	GTR	0.0408
	FUBAR	18	GTR				
ORF6	SLAC	0	GTR	0.349			
	FEL	0	GTR	0.331			
	MEME				0	GTR	0.331
	FUBAR	0	GTR				
ORF7a	SLAC	0	GTR	0.194			
	FEL	7	GTR	0.182			
	MEME				0	GTR	0.182
	FUBAR	5	GTR				
ORF7b	SLAC	0	GTR	0.355			
	FEL	1	GTR	0.312			
	MEME				0	GTR	0.312
	FUBAR	1	GTR				
ORF8	SLAC						
	FEL						
	MEME						
	FUBAR						

N	SLAC	10	GTR	0.371	1	GTR	0.371
	FEL	25	GTR	0.175	1	GTR	0.328
	MEME				5	GTR	0.328
	FUBAR	26	GTR		2	GTR	
ORF9b	SLAC	0	GTR	1.33			
	FEL	2	GTR	1.30			
	MEME				0	GTR	1.30
	FUBAR				2	GTR	
ORF9c	SLAC						
	FEL						
	MEME						
	FUBAR						
ORF10	SLAC	0	GTR	1.35	1	GTR	1.35
	FEL	0	GTR	1.30	1	GTR	1.40
	MEME				1	GTR	1.40
	FUBAR				1	GTR	

Positive
No sites
No report

Negative/Purifyng											
**Gene**	**Gene codon position**	**SLAC**		**FEL**		**FUBAR**		**Inferred substitution**			**Amino acid**
		**dN-dS**	**P[dN/dS < 1]**	**dN/dS**	**p-value**	**dN-dS**	**Prob[dS>dN]**	**Bat**	**Intermediate**	**Human**	
**ORF1a**	142	-1.21	0.038	0.0	0.003	-9.684	0.999	TCC, TCG	TCC, TCA	TCA	S
	180	-1.21	0.041	0.0	0.002	-12.906	0.999	GGA	GGG, GGA	GGA	G
	206	-1.21	0.037	0.0	0.002	-11.142	0.999	GCT, GCC	GCG, GCA	GCA	A
	225	-1.60	0.021	0.0	0.002	-13.368	0.999	AAA	AAG	AAA, AAG	K
	491	-1.21	0.037	0.0	0.004	-10.04	0.999	GTG, GTA	GTC	GTG, GTT	V
	599	-1.21	0.037	0.0	0.002	-7.354	0.999	GCA, GCT	GCA, GCT, GCC	GCC, GCT	A
	802	-1.13	0.045	0.0	0.001	-11.704	0.999	CTT, CTA	CTT	CTT, CTA, CTC	L
	875	-1.21	0.037	0.0	0.005	-8.527	0.999	GTA, GTT	GTC, GTG	GTG	V
	924	-1.43	0.022	0.0	0.0	-13.835	0.999	TTT, TTC	TTC, TTT	TTC. TTT	F
	1253	-1.39	0.024	0.0	0.01	-5.655	0.997	AAC, AAT	AAT, AAC	AAT, AAC	N
	1331	-1.21	0.037	0.0	0.001	-14.446	1.0	CCT, CCA	CCT, CCG,	CCG	P
	1427	-1.62	0.012	0.0	0.001	-9.076	1.0	ACC, ACT	ACC, ACT	ACC, ACT	T
	1536	-1.21	0.037	0.0	0.001	-17.719	0.999	GTG, GTT	GTT, GTA	GTG, GTA	V
	1762	-1.21	0.037	0.0	0.002	-7.44	0.999	ACT, ACA	ACT, ACC	ACT, ACC	T
	1908	-1.35	0.026	0.0	0.006	-5.934	0.998	TTT, TTC	TTT, TTC	TTT, TTC	F
	1936	-1.25	0.033	0.0	0.016	-4.87	0.997	ATC	ATT, ATC	ATT, ATC	I
	2008	-1.21	0.037	0.0	0.012	-5.106	0.998	ACC, ACT	ACC, ACT	ACC, ACT	T
	2335	-1.54	0.018	0.0	0.001	-9.018	0.999	TTT	TTT, TTC	TTT, TTC	F
	2339	-138.06	0.025	0.0	0.011	-5.214	0.998	TTC	TTT, TTC	TTT, TTC	F
	2386	-1.47	0.020	0.0	0.003	-7.416	0.999	ATT	ATC, ATA	ATC, ATT	I
	2422	-1.21	0.037	0.0	0.003	-7.375	0.999	GTT	GTC, GTT	GTC, GTT	V
	2501	-2.02	0.0041	0.0	0.001	-10.173	1.0	TCC	TCC, TCT	TCC, TCT	S
	2527	-1.39	0.024	0.0	0.01	-5.657	0.997	AAT, AAC	AAT, AAC	AAC, AAT	N
	2626	-1.21	0.037	0.0	0.018	-4.765	0.997	TCC	TCC, TCT	TCC	S
	2709	-1.39	0.024	0.0	0.01	-5.66	0.997	AAC, AAT	AAC, AAT	AAC, AAT	N
	2840	-1.54	0.018	0.0	0.001	-9.324	0.999	AGT	AGC, AGT	AGC, AGT	S
	3019	-1.39	0.024	0.0	0.007	-5.605	0.998	TTT, TTC	TTT, TTC	TTT, TTC	F
	3048	-162.86	0.027	0.0	0.002	-9.3	0.997	ATA, ATT	ATA, ATT	ATA	I
	3322	-1.21	0.037	0.0	0.009	-5.313	0.998	CTT	CTC	CTC, CTT	L
	3339	-1.62	0.012	0.0	0.003	-6.97	0.999	CTC, CTT	CTC, CTT	CTC, CTT	L
	3378	-1.21	0.037	0.0	0.009	-7.402	0.999	GTG	GTA, GTG	GTT, GTA	V
	3731	-1.21	0.037	0.0	0.013	-5.079	0.998	GCC, GCT	GCC, GCT	GCC, GCT	A
	3832	-1.21	0.037	0.0	0.008	-5.6	0.998	CCC, CCA	CCT, CCC	CCC	P
	3854	-1.75	0.0091	0.0	0.0	-12.623	1.0	ATT	ATC, ATT	ATC, ATT	I
	4059	-1.21	0.037	0.0	0.003	-7.016	0.999	CCA	CCG, CCC	CCT, CCC	P
**ORF1b**	346	-1.58	0.031	0.0	0.008	-8.571	0.999	TAC	TAC, TAT	TAC, TAT	Y
	504	-1.68	0.024	0.0	0.006	-8.104	0.999	CAT, CAC	CAT, CAC	CAT, CAC	H
	657	-2.44	0.0051	0.0	0.0	-16.385	1.0	TTT, TTC	TTT, TTC	TTT, TTC	F
	887	-1.58	0.031	0.0	0.009	-8.558	0.999	GTC	GTC	GTC, GTT	V
	1261	-1.46	0.037	0.0	0.011	-7.263	0.999	CTT, CTC	CTT, CTC	CTT, CTC	L
	1318	-1.46	0.037	0.0	0.01	-11.116	1.0	GTG	GTG, GTA	GTA, GTT	V
	1326	-1.68	0.024	0.0	0.008	-8.112	0.999	AAT, AAC	AAT, AAC	AAC, AAT	N
	1468	-1.46	0.037	0.0	0.009	-7.398	0.999	CCT	CCA, CCT	CCT	P
	1495	-1.78	0.020	0.0	0.005	-8.702	0.999	AAT	AAC	AAC, AAT	N
	1687	-1.46	0.037	0.0	0.003	-9.745	1.0	GTT	GTA, GTC	GTC, GTT	V
	1919	-1.46	0.037	0.0	0.002	-9.828	1.0	CTT	CTT, CTC	CTC, CTT	L
	1945	-1.68	0.024	0.0	0.008	-8.023	0.998	AAT, AAC	AAT, AAC	AAT, AAC	N
	2496	-2.44	0.0041	0.0	0.0	-39.287	1.0	GCA	GCG, GCC	GCG, GCC, GCT	A
	2555	-1.46	0.037	0.0	0.001	-15.703	1.0	GCA	GCT	GCT, GCC	A
	2583	-178.42	0.020	0.0	0.005	-8.706	0.999	AAT	AAC	AAC, AAT	N
**S**	64	-6.43	0.039	0.0	0.001	-6.971	0.998	TTT	TTT, TTC	TTC	F
	290	-937.79	0.022	0.0	0.002	-10.014	0.997	GAG	GAA	GAA	E
	356	-6.31	0.041	0.0	0.005	-5.473	0.994	TTC	TTT	TTT	F
	419	-6.82	0.022	0.0	0.001	-7.103	0.999	ATA, ATT	ATC, ATT	ATC, ATT	I
	482	-848.26	0.016	0.0	0.001	-11.981	0.999	TAT, TAC	TAT, TAC	TAT, TAC	Y
	484	-5.75	0.037	0.0	0.001	-7.827	0.999	GCA	GCT, GCC	GCT, GCC	A
	501	-6.57	0.033	0.0	0.0	-20.518	1.0	TTG, CTT	CTA, TTA	TTA, CTA	L
	552	-6.43	0.039	0.0	0.001	-6.984	0.998	TTT	TTC, TTT	TTC, TTT	F
	636	-6.31	0.041	0.0	0.006	-5.598	0.993	GAC	GAT	GAT	D
	732	-9.59	0.0041	0.0	0.0	-27.923	1.0	ACC	ACC	ACC, ACT	T

	773	-6.43	0.039	0.0	0.001	-7.076	0.997	AAT	AAT, AAC	AAT, AAC	N
	787	-5.75	0.037	0.0	0.001	-6.936	0.999	ACT	ACA, ACC	ACC, ACT	T
	799	-754.66	0.039	0.0	0.003	-9.71	0.997	AAA, AAG	AAA, AAG	AAA	K
	828	-937.79	0.022	0.0	0.002	-10.014	0.997	GAG	GAA	GAA	E
	833	-8.28	0.012	0.0	0.001	-6.783	0.998	AAT	AAT, AAC	AAC	N
	864	-6.61	0.037	0.0	0.0	-11.338	0.998	TTT, TTC	TTT	TTT, TTC	F
	865	-8.17	0.012	0.0	0.001	-7.113	0.998	AAC, AAT	AAC, AAT	AAC, AAT	N
	988	-6.49	0.038	0.0	0.001	-9.037	0.996	GAT	GAT, GAC	GAT, GAC	D
	1125	-5.75	0.037	0.0	0.001	-9.889	0.999	ACA, ACT	ACC, ACT	ACC, ACT	T
	1259	-66.17	0.046	0.0	0.0	-11.603	0.998	TGC, TGT	TGT	TGC, TGT	C
**ORF3a**	7	-1.30	0.031	0.0	0.012	-11.483	0.994	ATC, ATT	ATC, ATT	ATC, ATT	I
	43	-1.33	0.028	0.0	0.006	-13.878	0.995	TTC	TTC	TTC, TTT	F
**M**	93	-3.18	0.0022	0.0	0.0	-39.662	1.0	CTG, CTC	CTT, CTC, CTG	CTT, CTC, CTG	L
	120	-1.79	0.034	0.0	0.003	-27.115	0.999	CTC, CTA	TTG, CTC, CTT	CTC	L
	135	-3.83	0.0049	0.0	0.0	-38.729	1.0	GAA, GAG	GAA, GAG	GAA, GAG	E
	138	-1.72	0.037	0.0	0.009	-16.63	0.997	CTC, CTT	CTC, CTT	CTC	L
**N**	8	-2.13	0.010	0.0	0.007	-16.598	0.996	AAC	AAT	AAC, AAT	N
	9	-1.99	0.045	0.0	0.01	-21.552	0.992	CAA	CAG	CAG	Q
	35	-1.41	0.037	0.0	0.009	-19.338	0.998	GCA	GCT, GCG	GCC, GCG	A
	110	-1.96	0.013	0.0	0.001	-15.622	0.998	TTT	TTT, TTC	TTT, TTC	F
	192	-1.84	0.016	0.0	0.017	-12.376	0.993	AAC, AAT	AAC	AAC, AAT	N
	214	-165.66	0.045	0.138	0.045	-16.173	0.98	GGC, GGT	GGC, GGT	GGC, GGT ǀ TGC	G ǀ C
	234	-1.77	0.0028	0.018	0.001	-33.084	0.978	ATG	ATG	ATG ǀ ATC, ATT, ATA	M ǀ I
	289	-1.99	0.045	0.0	0.01	-22.028	0.992	CAG, CAA	CAG, CAA	CAG	Q
	348	-1.43	0.048	0.0	0.022	-10.381	0.975	GAT, GAC	GAT	GAT, GAC	D
	374	-1.99	0.030	0.0	0.008	-21.56	0.992	AAA	AAA, AAG	AAG	K

Table S5. RNAz values showing RNA structures that are common, shared or unique across three hosts for each *Beta-CoV*.

SARS-CoV: RNAz values

Host	ORF	Locus_ID	Strand	Start	End	P	Type
Intermediate	5UTR	locus1	Forward	1	120	0.99662	Unique
Human	5UTR	locus3	Forward	41	160	0.997725	Shared
Bat	5UTR	locus3	Forward	41	160	0.997445	Shared
Human	5UTR	locus5	Forward	81	200	0.998361	Unique
Human	ORF1a	locus9	Forward	161	280	0.996077	Shared
Bat	ORF1a	locus9	Forward	161	280	0.997289	Shared
Human	ORF1a	locus13	Forward	721	840	0.995648	Unique
Intermediate	ORF1a	locus13	Forward	841	960	0.998649	Unique
Intermediate	ORF1a	locus15	Forward	881	1000	0.99774	Unique
Human	ORF1a	locus17	Forward	921	1040	0.998601	Shared
Bat	ORF1a	locus13	Forward	921	1040	0.996947	Shared
Bat	ORF1a	locus15	Forward	961	1080	0.990563	Unique
Intermediate	ORF1a	locus21	Forward	1041	1160	0.996295	Unique
Human	ORF1a	locus23	Forward	1081	1200	0.997182	Unique
Intermediate	ORF1a	locus25	Forward	1121	1240	0.997229	Unique
Human	ORF1a	locus29	Forward	1241	1360	0.997385	Unique
Intermediate	ORF1a	locus29	Forward	1481	1600	0.99555	Unique
Intermediate	ORF1a	locus31	Forward	1521	1640	0.99757	Unique
Intermediate	ORF1a	locus35	Forward	1641	1760	0.997942	Unique
Intermediate	ORF1a	locus43	Forward	2081	2200	0.998298	Unique
Intermediate	ORF1a	locus45	Forward	2241	2360	0.997507	Unique
Intermediate	ORF1a	locus49	Forward	2441	2560	0.998527	Unique
Human	ORF1a	locus49	Forward	2481	2600	0.998621	Unique
Bat	ORF1a	locus37	Forward	2521	2640	0.998038	Unique
Intermediate	ORF1a	locus53	Forward	2681	2800	0.997017	Unique
Human	ORF1a	locus55	Forward	2721	2840	0.998371	Unique
Bat	ORF1a	locus43	Forward	2761	2880	0.998823	Unique
Intermediate	ORF1a	locus57	Forward	2841	2960	0.998199	Unique
Human	ORF1a	locus57	Forward	2881	3000	0.99825	Shared
Intermediate	ORF1a	locus59	Forward	2881	3000	0.996628	Shared
Human	ORF1a	locus59	Forward	2921	3040	0.99725	Unique
Human	ORF1a	locus61	Forward	2961	3080	0.996631	Shared
Bat	ORF1a	locus49	Forward	2961	3080	0.990113	Shared
Human	ORF1a	locus63	Forward	3001	3120	0.998056	Unique
Intermediate	ORF1a	locus65	Forward	3081	3200	0.99689	Unique
Intermediate	ORF1a	locus66	Reverse	3081	3240	0.997945	Unique
Bat	ORF1a	locus51	Forward	3201	3320	0.98317	Unique
Bat	ORF1a	locus55	Forward	3801	3920	0.997882	Unique
Bat	ORF1a	locus57	Forward	3841	3960	0.997612	Unique
Intermediate	ORF1a	locus81	Forward	4041	4160	0.996071	Unique
Human	ORF1a	locus83	Forward	4081	4200	0.996631	Unique
Bat	ORF1a	locus63	Forward	4121	4240	0.997413	Unique

Bat	ORF1a	locus65	Forward	4161	4280	0.998085	Unique
Human	ORF1a	locus89	Forward	4321	4440	0.997517	Unique
Human	ORF1a	locus95	Forward	4441	4560	0.996808	Unique
Bat	ORF1a	locus69	Forward	4481	4600	0.996779	Unique
Bat	ORF1a	locus71	Forward	4761	4880	0.998652	Unique
Intermediate	ORF1a	locus109	Forward	4921	5040	0.997596	Unique
Human	ORF1a	locus109	Forward	5001	5120	0.998225	Shared
Intermediate	ORF1a	locus111	Forward	5001	5120	0.997331	Shared
Human	ORF1a	locus111	Forward	5121	5240	0.998033	Shared
Intermediate	ORF1a	locus117	Forward	5121	5240	0.998216	Shared
Bat	ORF1a	locus81	Forward	5161	5280	0.9987	Unique
Intermediate	ORF1a	locus119	Forward	5201	5320	0.997042	Shared
Bat	ORF1a	locus83	Forward	5201	5320	0.997503	Shared
Human	ORF1a	locus115	Forward	5241	5360	0.997784	Unique
Intermediate	ORF1a	locus121	Forward	5521	5640	0.997048	Unique
Bat	ORF1a	locus87	Forward	5601	5720	0.99734	Unique
Bat	ORF1a	locus89	Forward	5641	5760	0.99856	Unique
Human	ORF1a	locus127	Forward	5761	5880	0.996589	Unique
Intermediate	ORF1a	locus127	Forward	6081	6200	0.998303	Unique
Human	ORF1a	locus133	Forward	6121	6240	0.998451	Common
Intermediate	ORF1a	locus129	Forward	6121	6240	0.997158	Common
Bat	ORF1a	locus91	Forward	6121	6240	0.998101	Common
Human	ORF1a	locus135	Forward	6161	6280	0.998261	Shared
Bat	ORF1a	locus93	Forward	6161	6280	0.99867	Shared
Human	ORF1a	locus139	Forward	6361	6480	0.997868	Shared
Intermediate	ORF1a	locus135	Forward	6361	6480	0.997168	Shared
Human	ORF1a	locus141	Forward	6401	6520	0.997404	Unique
Human	ORF1a	locus143	Forward	6441	6560	0.996928	Unique
Human	ORF1a	locus147	Forward	6921	7040	0.997697	Shared
Bat	ORF1a	locus97	Forward	6921	7040	0.995198	Shared
Human	ORF1a	locus149	Forward	6961	7080	0.995892	Shared
Bat	ORF1a	locus99	Forward	6961	7080	0.997017	Shared
Intermediate	ORF1a	locus147	Forward	7001	7120	0.997535	Unique
Human	ORF1a	locus153	Forward	7041	7160	0.997281	Unique
Intermediate	ORF1a	locus155	Forward	7441	7560	0.997216	Unique
Intermediate	ORF1a	locus157	Forward	7601	7720	0.996725	Unique
Intermediate	ORF1a	locus177	Forward	8841	8960	0.996157	Unique
Bat	ORF1a	locus127	Forward	8961	9080	0.980919	Unique
Intermediate	ORF1a	locus183	Forward	9081	9200	0.997249	Unique
Human	ORF1a	locus185	Forward	9121	9240	0.998219	Unique
Human	ORF1a	locus187	Forward	9161	9280	0.99639200000000006	Unique
Human	ORF1a	locus189	Forward	9281	9400	0.986915	Unique
Human	ORF1a	locus207	Forward	10881	11000	0.998171	Unique
Human	ORF1a	locus209	Forward	10921	11040	0.997149	Shared
Intermediate	ORF1a	locus207	Forward	10921	11040	0.997964	Shared
Human	ORF1a	locus211	Forward	10961	11080	0.996542	Shared
Intermediate	ORF1a	locus209	Forward	10961	11080	0.996889	Shared
Human	ORF1a	locus213	Forward	11001	11120	0.997945	Shared

Bat	ORF1a	locus143	Forward	11001	11120	0.99875	Shared
Intermediate	ORF1a	locus225	Forward	13241	13360	0.998434	Unique
Human	ORF1a	locus225	Forward	13281	13400	0.99867	Unique
Bat	NA	locus159	Forward	13321	13440	0.997282	Unique
Intermediate	ORF1b	locus233	Forward	13601	13720	0.995426	Unique
Bat	ORF1b	locus163	Forward	13681	13800	0.996274	Unique
Human	ORF1b	locus233	Forward	13801	13920	0.998381	Unique
Intermediate	ORF1b	locus243	Forward	14081	14200	0.998171	Shared
Bat	ORF1b	locus171	Forward	14081	14200	0.995567	Shared
Human	ORF1b	locus243	Forward	14121	14240	0.998608	Unique
Human	ORF1b	locus245	Forward	14161	14280	0.995582	Unique
Bat	ORF1b	locus177	Forward	14361	14480	0.997181	Unique
Intermediate	ORF1b	locus249	Forward	14401	14520	0.997374	Unique
Human	ORF1b	locus249	Forward	14441	14560	0.997519	Unique
Bat	ORF1b	locus181	Forward	14481	14600	0.997932	Unique
Intermediate	ORF1b	locus253	Forward	14641	14760	0.997092	Unique
Intermediate	ORF1b	locus255	Forward	14681	14800	0.998562	Unique
Human	ORF1b	locus257	Forward	14721	14840	0.997048	Shared
Intermediate	ORF1b	locus257	Forward	14721	14840	0.997841	Shared
Human	ORF1b	locus259	Forward	14761	14880	0.997987	Shared
Bat	ORF1b	locus189	Forward	14761	14880	0.998337	Shared
Bat	ORF1b	locus191	Forward	14801	14920	0.998332	Unique
Human	ORF1b	locus267	Forward	15521	15640	0.996397	Unique
Human	ORF1b	locus275	Forward	16361	16480	0.998361	Unique
Human	ORF1b	locus277	Forward	16401	16520	0.996447	Unique
Human	ORF1b	locus280	Forward	16801	16920	0.998225	Unique
Human	ORF1b	locus288	Forward	17441	17560	0.993841	Shared
Bat	ORF1b	locus205	Forward	17441	17560	0.996955	Shared
Intermediate	ORF1b	locus293	Forward	17521	17640	0.997646	Unique
Intermediate	ORF1b	locus305	Forward	18681	18800	0.995582	Unique
Human	ORF1b	locus306	Forward	18961	19080	0.996426	Unique
Intermediate	ORF1b	locus313	Forward	19281	19400	0.997267	Unique
Human	ORF1b	locus310	Forward	19321	19440	0.998655	Unique
Bat	ORF1b	locus227	Forward	19361	19480	0.998073	Unique
Human	ORF1b	locus316	Forward	19681	19800	0.997313	Unique
Intermediate	ORF1b	locus321	Forward	19721	19840	0.996495	Unique
Human	ORF1b	locus318	Forward	19761	19880	0.995226	Unique
Intermediate	ORF1b	locus325	Forward	19801	19920	0.998185	Unique
Bat	ORF1b	locus237	Forward	20481	20600	0.994619	Unique
Human	ORF1b	locus330	Forward	20521	20640	0.997344	Unique
Intermediate	ORF1b	locus335	Forward	20681	20800	0.995648	Unique
Intermediate	ORF1b	locus337	Forward	20761	20880	0.997078	Shared
Bat	ORF1b	locus245	Forward	20761	20880	0.99893	Shared
Intermediate	ORF1b	locus349	Forward	21241	21360	0.998417	Unique
Human	ORF1b	locus348	Forward	21281	21400	0.99851	Unique
Human	ORF1b	locus350	Forward	21321	21440	0.997313	Unique
Human	S	locus360	Forward	21921	22040	0.998429	Shared
Intermediate	S	locus359	Forward	21921	22040	0.996723	Shared

Human	S	locus362	Forward	21961	22080	0.998225	Shared
Intermediate	S	locus361	Forward	21961	22080	0.998501	Shared
Human	S	locus366	Forward	22041	22160	0.99805	Unique
Bat	S	locus250	Forward	22401	22520	0.993106	Unique
Intermediate	S	locus371	Forward	22921	23040	0.998631	Unique
Bat	S	locus254	Forward	23401	23520	0.997611	Unique
Intermediate	S	locus385	Forward	23441	23600	0.998092	Unique
Human	S	locus398	Forward	23441	23680	0.998646	Unique
Intermediate	S	locus386	Reverse	23481	23600	0.998092	Unique
Intermediate	S	locus387	Forward	23521	23640	0.998691	Unique
Human	S	locus399	Reverse	23561	23680	0.998646	Shared
Intermediate	S	locus389	Forward	23561	23680	0.997963	Shared
Human	S	locus400	Forward	23601	23720	0.998463	Unique
Bat	S	locus258	Forward	23801	23920	0.992708	Unique
Human	S	locus412	Forward	24881	25000	0.99583	Unique
Intermediate	ORF3a	locus403	Forward	25361	25480	0.998411	Unique
Human	ORF3a	locus418	Forward	25521	25640	0.998459	Unique
Intermediate	ORF3a	locus409	Forward	25681	25800	0.996239	Unique
Human	ORF3a	locus422	Forward	25721	25840	0.997042	Unique
Human	ORF3a	locus426	Forward	25881	26000	0.997852	Shared
Intermediate	ORF3a	locus413	Forward	25881	26000	0.997522	Shared
Intermediate	ORF3a	locus415	Forward	25921	26040	0.995463	Shared
Bat	ORF3a	locus278	Forward	25921	26040	0.998533	Shared
Human	ORF3a	locus430	Forward	25961	26080	0.997649	Common
Intermediate	ORF3a	locus417	Forward	25961	26080	0.991483	Common
Bat	ORF3a	locus280	Forward	25961	26080	0.998128	Common
Intermediate	ORF3b	locus419	Forward	26001	26120	0.998569	Shared
Bat	ORF3b	locus282	Forward	26001	26120	0.994239	Shared
Human	E	locus434	Forward	26041	26160	0.997781	Common
Intermediate	E	locus421	Forward	26041	26160	0.995777	Common
Bat	E	locus284	Forward	26041	26160	0.998635	Common
Human	E	locus436	Forward	26081	26200	0.998683	Unique
Human	M	locus440	Forward	26321	26440	0.997815	Unique
Human	M	locus442	Forward	26361	26480	0.997882	Common
Intermediate	M	locus429	Forward	26361	26480	0.995479	Common
Bat	M	locus291	Reverse	26361	26480	0.980714	Common
Human	ORF7a	locus450	Forward	27401	27520	0.996447	Unique
Bat	N	locus302	Forward	28601	28720	0.995615	Unique
Human	N	locus460	Forward	28681	28800	0.998626	Unique
Human	N	locus466	Forward	28881	29000	0.996077	Shared
Bat	N	locus306	Forward	28881	29000	0.996571	Shared
Bat	3UTR	locus314	Forward	29401	29520	0.996741	Unique

MERS-CoV: RNAz values

Host	ORF	Locus_II	Strand	Start	End	P	Type
Human	5UTR	locus2	Forward	81	200	0.99815	Shared
Intermediate	5UTR	locus2	Forward	81	200	0.997948	Shared
Human	ORF1a	locus4	Forward	121	280	0.998428	Shared
Intermediate	ORF1a	locus4	Forward	121	280	0.997858	Shared
Bat	ORF1a	locus2	Forward	521	920	0.998757	Unique
Human	ORF1a	locus9	Forward	721	960	0.998671	Shared
Intermediate	ORF1a	locus9	Forward	721	960	0.99865	Shared
Human	ORF1a	locus11	Forward	881	1000	0.998151	Shared
Intermediate	ORF1a	locus11	Forward	881	1000	0.998157	Shared
Human	ORF1a	locus13	Forward	921	1040	0.998639	Shared
Intermediate	ORF1a	locus13	Forward	921	1040	0.998648	Shared
Human	ORF1a	locus15	Forward	961	1080	0.998618	Shared
Intermediate	ORF1a	locus15	Forward	961	1080	0.998628	Shared
Bat	ORF1a	locus6	Forward	961	1200	0.996946	Unique
Bat	ORF1a	locus7	Reverse	1081	1200	0.997311	Unique
Human	ORF1a	locus23	Forward	1161	1280	0.995978	Unique
Intermediate	ORF1a	locus19	Forward	1161	1360	0.996092	Unique
Bat	ORF1a	locus9	Reverse	1161	1480	0.997005	Unique
Human	ORF1a	locus25	Forward	1201	1360	0.996219	Unique
Bat	ORF1a	locus10	Forward	1481	1640	0.998754	Unique
Bat	ORF1a	locus12	Forward	1601	1840	0.998123	Unique
Bat	ORF1a	locus14	Forward	1921	2040	0.996814	Unique
Human	ORF1a	locus27	Forward	1961	2080	0.996565	Shared
Intermediate	ORF1a	locus21	Forward	1961	2080	0.996655	Shared
Bat	ORF1a	locus17	Forward	2121	2320	0.998564	Unique
Human	ORF1a	locus28	Forward	2201	2320	0.997763	Shared
Intermediate	ORF1a	locus22	Forward	2201	2320	0.997671	Shared
Human	ORF1a	locus32	Forward	2361	2480	0.99601	Shared
Intermediate	ORF1a	locus26	Forward	2361	2480	0.996467	Shared
Bat	ORF1a	locus18	Forward	2361	2520	0.990368	Unique
Human	ORF1a	locus37	Forward	2601	2800	0.997067	Shared
Intermediate	ORF1a	locus31	Forward	2601	2800	0.981289	Shared
Human	ORF1a	locus38	Reverse	2681	2800	0.998181	Shared
Intermediate	ORF1a	locus32	Reverse	2681	2800	0.998608	Shared
Bat	ORF1a	locus25	Reverse	3121	3240	0.998426	Unique
Bat	ORF1a	locus26	Forward	3161	3280	0.998143	Unique
Bat	ORF1a	locus28	Forward	3201	3440	0.99839	Unique
Bat	ORF1a	locus29	Reverse	3321	3440	0.998286	Unique
Human	ORF1a	locus41	Reverse	3361	3480	0.996951	Common
Intermediate	ORF1a	locus35	Reverse	3361	3480	0.986116	Common
Bat	ORF1a	locus30	Forward	3361	3480	0.996447	Common

Human	ORF1a	locus43	Reverse	3401	3520	0.998454	Unique
Intermediate	ORF1a	locus37	Reverse	3401	3560	0.997626	Unique
Human	ORF1a	locus45	Reverse	3441	3640	0.998493	Unique
Bat	ORF1a	locus32	Forward	3481	3760	0.998417	Unique
Human	ORF1a	locus46	Forward	3561	3680	0.998221	Shared
Intermediate	ORF1a	locus40	Forward	3561	3680	0.998532	Shared
Human	ORF1a	locus51	Forward	3801	3920	0.995697	Shared
Intermediate	ORF1a	locus45	Forward	3801	3920	0.996233	Shared
Intermediate	ORF1a	locus46	Reverse	3801	4000	0.998356	Unique
Bat	ORF1a	locus38	Forward	3881	4240	0.998017	Unique
Human	ORF1a	locus56	Forward	4281	4400	0.998188	Shared
Intermediate	ORF1a	locus50	Forward	4281	4400	0.997926	Shared
Human	ORF1a	locus58	Forward	4321	4480	0.997129	Shared
Intermediate	ORF1a	locus52	Forward	4321	4480	0.997367	Shared
Human	ORF1a	locus59	Reverse	4401	4520	0.99223	Shared
Intermediate	ORF1a	locus53	Reverse	4401	4520	0.998317	Shared
Bat	ORF1a	locus42	Forward	4401	4560	0.997676	Unique
Human	ORF1a	locus63	Reverse	4641	4760	0.994506	Unique
Human	ORF1a	locus64	Forward	4681	4800	0.99757	Shared
Intermediate	ORF1a	locus57	Forward	4681	4800	0.997425	Shared
Human	ORF1a	locus65	Reverse	4681	4880	0.998649	Shared
Intermediate	ORF1a	locus58	Reverse	4681	4880	0.997684	Shared
Bat	ORF1a	locus47	Forward	4841	5040	0.998157	Unique
Bat	ORF1a	locus48	Forward	5121	5240	0.998508	Unique
Human	ORF1a	locus70	Forward	5721	5840	0.998478	Shared
Intermediate	ORF1a	locus63	Forward	5721	5840	0.998442	Shared
Human	ORF1a	locus71	Reverse	5721	5880	0.998461	Shared
Intermediate	ORF1a	locus64	Reverse	5721	5880	0.998482	Shared
Human	ORF1a	locus73	Reverse	5801	5920	0.997593	Common
Intermediate	ORF1a	locus66	Reverse	5801	5920	0.997591	Common
Bat	ORF1a	locus54	Reverse	5801	5920	0.9974	Common
Bat	ORF1a	locus56	Reverse	5841	5960	0.996019	Unique
Bat	ORF1a	locus57	Forward	5881	6040	0.996188	Unique
Bat	ORF1a	locus60	Reverse	6001	6120	0.996928	Unique
Human	ORF1a	locus75	Reverse	6041	6160	0.996617	Shared
Intermediate	ORF1a	locus68	Reverse	6041	6160	0.996618	Shared
Human	ORF1a	locus76	Forward	6081	6200	0.997347	Shared
Intermediate	ORF1a	locus69	Forward	6081	6200	0.996601	Shared
Human	ORF1a	locus78	Forward	6161	6320	0.997607	Shared
Intermediate	ORF1a	locus71	Forward	6161	6320	0.993046	Shared
Bat	ORF1a	locus65	Forward	6201	6320	0.997982	Unique
Human	ORF1a	locus80	Forward	6241	6400	0.998671	Shared
Intermediate	ORF1a	locus73	Forward	6241	6400	0.998299	Shared
Human	ORF1a	locus81	Reverse	6281	6400	0.998717	Shared
Intermediate	ORF1a	locus74	Reverse	6281	6400	0.998747	Shared

Human	ORF1a	locus82	Forward	6321	6440	0.998133	Shared
Intermediate	ORF1a	locus75	Forward	6321	6440	0.998268	Shared
Human	ORF1a	locus85	Reverse	7161	7280	0.998113	Shared
Intermediate	ORF1a	locus78	Reverse	7161	7280	0.998155	Shared
Bat	ORF1a	locus80	Forward	7801	7920	0.997941	Unique
Human	ORF1a	locus88	Forward	7961	8200	0.997408	Shared
Intermediate	ORF1a	locus81	Forward	7961	8200	0.996242	Shared
Human	ORF1a	locus95	Forward	9801	9920	0.996783	Shared
Intermediate	ORF1a	locus88	Forward	9801	9920	0.99683600000000006	Shared
Bat	ORF1a	locus99	Forward	11561	11680	0.997114	Unique
Human	ORF1a	locus103	Reverse	11561	11800	0.99751	Shared
Intermediate	ORF1a	locus96	Reverse	11561	11800	0.998082	Shared
Bat	ORF1a	locus101	Forward	11881	12040	0.998701	Unique
Human	ORF1a	locus108	Reverse	12681	12800	0.996065	Shared
Intermediate	ORF1a	locus101	Reverse	12681	12800	0.995777	Shared
Human	ORF1a	locus112	Reverse	13001	13120	0.998056	Shared
Intermediate	ORF1a	locus106	Reverse	13001	13120	0.998017	Shared
Bat	ORF1a	locus104	Reverse	13001	13160	0.997712	Unique
Bat	NA	locus105	Forward	13241	13480	0.997111	Unique
Bat	NA	locus106	Reverse	13361	13480	0.996397	Unique
Human	NA	locus117	Forward	13401	13520	0.998225	Common
Intermediate	NA	locus111	Forward	13401	13520	0.998239	Common
Bat	NA	locus107	Forward	13401	13520	0.997114	Common
Bat	NA	locus112	Reverse	13481	13600	0.998536	Unique
Bat	ORF1b	locus113	Forward	13521	13800	0.99774	Unique
Human	ORF1b	locus124	Reverse	13721	13840	0.998238	Shared
Intermediate	ORF1b	locus118	Reverse	13721	13840	0.998238	Shared
Bat	ORF1b	locus118	Reverse	14001	14120	0.996495	Unique
Human	ORF1b	locus128	Reverse	14081	14200	0.997495	Shared
Intermediate	ORF1b	locus123	Reverse	14081	14200	0.998381	Shared
Human	ORF1b	locus131	Forward	14161	14320	0.996751	Unique
Intermediate	ORF1b	locus126	Forward	14201	14320	0.996849	Unique
Human	ORF1b	locus137	Forward	14361	14480	0.998371	Shared
Intermediate	ORF1b	locus132	Forward	14361	14480	0.9984	Shared
Human	ORF1b	locus138	Reverse	14361	14520	0.998644	Shared
Intermediate	ORF1b	locus133	Reverse	14361	14520	0.998668	Shared
Bat	ORF1b	locus122	Reverse	14761	14880	0.998157	Unique
Human	ORF1b	locus139	Forward	14841	14960	0.997179	Shared
Intermediate	ORF1b	locus134	Forward	14841	14960	0.997183	Shared
Human	ORF1b	locus140	Reverse	14841	15000	0.998036	Shared
Intermediate	ORF1b	locus135	Reverse	14841	15000	0.998033	Shared
Bat	ORF1b	locus126	Forward	15241	15440	0.998572	Unique
Human	ORF1b	locus143	Forward	15321	15440	0.998157	Shared
Intermediate	ORF1b	locus138	Forward	15321	15440	0.998033	Shared
Human	ORF1b	locus145	Forward	15361	15480	0.998534	Shared

Intermediate	ORF1b	locus140	Forward	15361	15480	0.998537	Shared
Bat	ORF1b	locus127	Forward	15481	15720	0.998521	Unique
Bat	ORF1b	locus128	Reverse	15601	15720	0.997486	Unique
Bat	ORF1b	locus130	Reverse	15641	15760	0.997945	Unique
Human	ORF1b	locus148	Forward	16001	16160	0.998426	Shared
Intermediate	ORF1b	locus143	Forward	16001	16160	0.998426	Shared
Human	ORF1b	locus149	Reverse	16041	16160	0.998591	Shared
Intermediate	ORF1b	locus144	Reverse	16041	16160	0.998591	Shared
Human	ORF1b	locus154	Reverse	16321	16520	0.998128	Unique
Intermediate	ORF1b	locus149	Reverse	16401	16520	0.998128	Unique
Human	ORF1b	locus155	Forward	16441	16560	0.997543	Shared
Intermediate	ORF1b	locus150	Forward	16441	16560	0.997621	Shared
Human	ORF1b	locus161	Forward	17081	17200	0.997784	Shared
Intermediate	ORF1b	locus155	Forward	17081	17200	0.998645	Shared
Bat	ORF1b	locus138	Forward	17201	17320	0.99791	Unique
Bat	ORF1b	locus140	Forward	17241	17480	0.997024	Unique
Human	ORF1b	locus164	Forward	17281	17400	0.996889	Shared
Intermediate	ORF1b	locus158	Forward	17281	17400	0.996889	Shared
Bat	ORF1b	locus145	Forward	17961	18080	0.998515	Unique
Intermediate	ORF1b	locus164	Reverse	18041	18160	0.996256	Unique
Intermediate	ORF1b	locus168	Reverse	18241	18360	0.996928	Unique
Human	ORF1b	locus174	Reverse	18241	18400	0.996961	Unique
Intermediate	ORF1b	locus170	Reverse	18281	18400	0.998061	Unique
Human	ORF1b	locus176	Reverse	18321	18440	0.994847	Shared
Intermediate	ORF1b	locus172	Reverse	18321	18440	0.997758	Shared
Bat	ORF1b	locus151	Forward	18841	18960	0.998613	Unique
Bat	ORF1b	locus153	Forward	19121	19360	0.998507	Unique
Human	ORF1b	locus183	Forward	19281	19440	0.997941	Shared
Intermediate	ORF1b	locus177	Forward	19281	19440	0.997942	Shared
Bat	ORF1b	locus156	Reverse	19321	19440	0.997939	Unique
Human	ORF1b	locus184	Reverse	19321	19520	0.995473	Unique
Bat	ORF1b	locus157	Forward	19361	19480	0.996823	Unique
Bat	ORF1b	locus160	Forward	19721	19840	0.997413	Unique
Bat	ORF1b	locus162	Reverse	19881	20000	0.996019	Unique
Bat	ORF1b	locus164	Reverse	19961	20080	0.998466	Unique
Human	ORF1b	locus196	Reverse	20201	20320	0.996218	Shared
Intermediate	ORF1b	locus189	Reverse	20201	20320	0.996498	Shared
Human	ORF1b	locus197	Forward	20241	20360	0.99783	Shared
Intermediate	ORF1b	locus190	Forward	20241	20360	0.997881	Shared
Bat	ORF1b	locus171	Forward	20601	20720	0.995648	Unique
Bat	ORF1b	locus172	Reverse	20601	20760	0.997005	Unique
Human	ORF1b	locus203	Forward	20841	21000	0.998666	Shared
Intermediate	ORF1b	locus196	Forward	20841	21000	0.998568	Shared
Bat	ORF1b	locus175	Forward	20841	21120	0.996077	Unique
Human	ORF1b	locus204	Reverse	20881	21000	0.998064	Shared

Intermediate	ORF1b	locus197	Reverse	20881	21000	0.994534	Shared
Human	S	locus209	Forward	21201	21520	0.998646	Shared
Intermediate	S	locus202	Forward	21201	21520	0.998644	Shared
Bat	ORF1b	locus177	Forward	21241	21400	0.998587	Unique
Bat	S	locus178	Reverse	21281	21640	0.997489	Unique
Intermediate	S	locus203	Reverse	21481	21640	0.996661	Unique
Human	S	locus212	Reverse	21521	21640	0.996984	Unique
Bat	S	locus179	Forward	21881	22000	0.997543	Unique
Human	S	locus219	Forward	22001	22160	0.997902	Shared
Intermediate	S	locus210	Forward	22001	22160	0.998044	Shared
Human	S	locus223	Forward	22161	22280	0.996953	Shared
Intermediate	S	locus214	Forward	22161	22280	0.996701	Shared
Intermediate	S	locus215	Reverse	22161	22320	0.998648	Unique
Bat	S	locus180	Forward	22281	22400	0.995734	Unique
Human	S	locus227	Reverse	22361	22520	0.998427	Shared
Intermediate	S	locus217	Reverse	22361	22520	0.998547	Shared
Bat	S	locus186	Forward	22561	22680	0.998549	Unique
Intermediate	S	locus220	Forward	22641	22840	0.99848	Unique
Human	S	locus232	Forward	22721	22840	0.998706	Shared
Intermediate	S	locus221	Reverse	22721	22840	0.997714	Shared
Human	S	locus234	Forward	22761	22880	0.997305	Shared
Intermediate	S	locus222	Forward	22761	22880	0.998001	Shared
Bat	S	locus190	Forward	22801	22920	0.997582	Unique
Bat	S	locus194	Forward	23161	23320	0.998212	Unique
Bat	S	locus196	Forward	23241	23360	0.996123	Unique
Bat	S	locus197	Reverse	23241	23400	0.997493	Unique
Human	S	locus238	Forward	23401	23520	0.996942	Shared
Intermediate	S	locus225	Forward	23401	23520	0.984869	Shared
Human	S	locus241	Reverse	24121	24240	0.997939	Shared
Intermediate	S	locus228	Reverse	24121	24240	0.997898	Shared
Intermediate	S	locus233	Forward	24561	24800	0.99642	Unique
Bat	S	locus204	Forward	24721	24880	0.995961	Unique
Bat	S	locus205	Reverse	24761	24880	0.996495	Unique
Bat	S	locus208	Forward	25001	25240	0.997945	Unique
Bat	ORF3	locus214	Reverse	25561	25680	0.996865	Unique
Bat	ORF4a	locus218	Reverse	26001	26120	0.997302	Unique
Bat	ORF4a	locus219	Forward	26041	26160	0.99562	Unique
Intermediate	ORF4b	locus239	Forward	26161	26320	0.995688	Unique
Intermediate	ORF4b	locus241	Forward	26321	26480	0.998648	Unique
Human	ORF4b	locus253	Forward	26361	26480	0.998654	Shared
Intermediate	ORF4b	locus242	Reverse	26361	26480	0.996113	Shared
Human	ORF4b	locus254	Reverse	26361	26600	0.995808	Unique
Bat	ORF5	locus226	Forward	26601	26920	0.997552	Unique
Bat	ORF5	locus227	Reverse	26801	26920	0.997281	Unique
Bat	ORF5	locus228	Forward	26841	26960	0.997926	Unique

Human	ORF5	locus259	Forward	26841	27200	0.998486	Shared
Intermediate	ORF5	locus247	Forward	26841	27200	0.998303	Shared
Bat	ORF5	locus230	Forward	26881	27000	0.998605	Unique
Bat	ORF5	locus232	Forward	26921	27040	0.998066	Unique
Bat	ORF5	locus234	Forward	26961	27080	0.998508	Unique
Bat	ORF5	locus236	Forward	27001	27160	0.997909	Unique
Bat	ORF5	locus238	Forward	27081	27200	0.997983	Unique
Bat	ORF5	locus241	Reverse	27121	27240	0.997868	Unique
Bat	ORF5	locus242	Forward	27161	27400	0.99678	Unique
Bat	ORF5	locus243	Reverse	27321	27440	0.998263	Unique
Human	ORF5	locus265	Reverse	27361	27480	0.996581	Common
Intermediate	ORF5	locus253	Reverse	27361	27480	0.997225	Common
Bat	ORF5	locus244	Forward	27361	27480	0.998263	Common
Human	E	locus266	Forward	27441	27600	0.99807	Shared
Intermediate	E	locus254	Forward	27441	27600	0.998033	Shared
Human	E	locus268	Forward	27521	27640	0.995714	Shared
Intermediate	E	locus256	Forward	27521	27640	0.995753	Shared
Bat	M	locus249	Forward	27761	27880	0.995628	Unique
Bat	M	locus251	Forward	27801	27920	0.996953	Unique
Bat	M	locus252	Reverse	27801	27960	0.991868	Unique
Human	M	locus271	Reverse	27881	28000	0.998263	Shared
Intermediate	M	locus258	Reverse	27881	28000	0.998288	Shared
Human	NA	locus279	Forward	28281	28520	0.995631	Shared
Intermediate	NA	locus266	Forward	28281	28520	0.995648	Shared
Human	N	locus281	Forward	28441	28640	0.998236	Shared
Intermediate	N	locus268	Forward	28441	28640	0.998263	Shared
Bat	N	locus263	Forward	29121	29320	0.99846	Unique
Bat	N	locus265	Forward	29361	29480	0.993477	Unique
Bat	N	locus266	Reverse	29361	29720	0.998578	Unique
Human	N	locus285	Forward	29561	29680	0.987408	Shared
Intermediate	N	locus274	Forward	29561	29680	0.985906	Shared
Human	N	locus286	Reverse	29561	29720	0.99768	Shared
Intermediate	N	locus275	Reverse	29561	29720	0.997384	Shared

SARS-CoV-2: RNAz values

Host	ORF	Locus_ID	Strand	Start	End	P	Type
Bat	5UTR	locus1	Forward	1	120	0.99882	Unique
Human	5UTR	locus1	Forward	1	200	0.998042	Unique
Human	ORF1a	locus4	Forward	281	400	0.990817	Unique
Intermediate	ORF1a	locus3	Forward	561	960	0.997512	Unique
Human	ORF1a	locus7	Forward	881	1000	0.998334	Shared
Intermediate	ORF1a	locus5	Forward	881	1000	0.998333	Shared
Human	ORF1a	locus9	Forward	921	1040	0.995626	Shared
Bat	ORF1a	locus4	Forward	921	1040	0.998509	Shared
Human	ORF1a	locus12	Reverse	1561	1680	0.997707	Shared
Intermediate	ORF1a	locus10	Forward	1561	1680	0.996908	Shared
Human	ORF1a	locus14	Reverse	1601	1720	0.998177	Unique
Bat	ORF1a	locus11	Forward	1681	1920	0.997714	Unique
Human	ORF1a	locus18	Forward	1921	2040	0.998514	Unique
Intermediate	ORF1a	locus16	Forward	2201	2360	0.996414	Unique
Human	ORF1a	locus19	Forward	2241	2400	0.997803	Unique
Intermediate	ORF1a	locus19	Forward	2481	2600	0.997443	Unique
Intermediate	ORF1a	locus23	Forward	2641	2840	0.996842	Unique
Human	ORF1a	locus21	Forward	2681	2880	0.998543	Unique
Human	ORF1a	locus22	Reverse	2761	2880	0.997503	Shared
Intermediate	ORF1a	locus25	Forward	2761	2880	0.997208	Shared
Human	ORF1a	locus25	Forward	2961	3080	0.997439	Unique
Intermediate	ORF1a	locus27	Forward	3041	3160	0.997332	Unique
Human	ORF1a	locus27	Forward	3041	3280	0.997875	Unique
Intermediate	ORF1a	locus29	Forward	3361	3720	0.99812	Unique
Human	ORF1a	locus28	Forward	3401	3560	0.998006	Unique
Human	ORF1a	locus30	Forward	3481	3680	0.996693	Unique
Intermediate	ORF1a	locus33	Reverse	4121	4280	0.998717	Unique
Human	ORF1a	locus34	Reverse	4121	4320	0.997381	Unique
Bat	ORF1a	locus20	Reverse	4161	4280	0.995934	Unique
Intermediate	ORF1a	locus39	Forward	4801	4920	0.998782	Unique
Intermediate	ORF1a	locus41	Forward	4841	5120	0.998568	Unique
Human	ORF1a	locus42	Forward	4881	5000	0.997451	Unique
Human	ORF1a	locus44	Forward	4921	5120	0.998653	Unique
Intermediate	ORF1a	locus42	Reverse	5001	5120	0.998708	Unique
Human	ORF1a	locus47	Reverse	5041	5160	0.997932	Shared
Intermediate	ORF1a	locus43	Forward	5041	5160	0.998074	Shared
Human	ORF1a	locus51	Reverse	5201	5320	0.997196	Shared
Bat	ORF1a	locus25	Forward	5201	5320	0.995898	Shared
Intermediate	ORF1a	locus47	Forward	5281	5480	0.998738	Unique
Human	ORF1a	locus54	Forward	5321	5480	0.998033	Unique
Human	ORF1a	locus55	Reverse	5361	5480	0.996542	Unique

Human	ORF1a	locus56	Forward	5401	5600	0.997922	Shared
Intermediate	ORF1a	locus49	Forward	5401	5600	0.997942	Shared
Intermediate	ORF1a	locus51	Forward	5521	5640	0.996647	Unique
Human	ORF1a	locus59	Reverse	5521	5680	0.997773	Unique
Human	ORF1a	locus60	Forward	5641	5760	0.996295	Unique
Human	ORF1a	locus62	Forward	5681	5800	0.997338	Shared
Intermediate	ORF1a	locus55	Forward	5681	5800	0.998408	Shared
Bat	ORF1a	locus29	Forward	5721	5840	0.996842	Unique
Intermediate	ORF1a	locus60	Forward	6041	6160	0.996993	Unique
Bat	ORF1a	locus30	Forward	6081	6200	0.997544	Unique
Bat	ORF1a	locus31	Reverse	6081	6240	0.995117	Unique
Human	ORF1a	locus70	Forward	6561	6680	0.997625	Shared
Intermediate	ORF1a	locus65	Forward	6561	6680	0.998015	Shared
Intermediate	ORF1a	locus67	Forward	6601	6720	0.998735	Unique
Intermediate	ORF1a	locus68	Reverse	6601	6760	0.998429	Unique
Intermediate	ORF1a	locus69	Forward	6681	6800	0.997023	Unique
Intermediate	ORF1a	locus79	Reverse	7761	7880	0.996231	Unique
Human	ORF1a	locus85	Forward	7801	8040	0.996676	Unique
Intermediate	ORF1a	locus80	Forward	7881	8040	0.998678	Unique
Bat	ORF1a	locus42	Forward	7961	8080	0.997344	Unique
Human	ORF1a	locus87	Forward	8761	8920	0.996016	Shared
Intermediate	ORF1a	locus82	Forward	8761	8920	0.998732	Shared
Bat	ORF1a	locus45	Forward	8801	8960	0.998735	Unique
Human	ORF1a	locus88	Reverse	8961	9120	0.997806	Unique
Intermediate	ORF1a	locus85	Forward	9241	9360	0.997991	Unique
Human	ORF1a	locus93	Reverse	9641	9760	0.995733	Unique
Bat	ORF1a	locus53	Reverse	9761	9880	0.994664	Unique
Human	ORF1a	locus102	Forward	11201	11320	0.996215	Unique
Human	ORF1a	locus104	Forward	11521	11640	0.998154	Unique
Intermediate	ORF1a	locus100	Forward	12321	12560	0.995868	Unique
Human	ORF1a	locus110	Reverse	12441	12560	0.997606	Unique
Human	ORF1a	locus112	Forward	12841	13000	0.996958	Unique
Intermediate	ORF1a	locus104	Forward	13321	13440	0.998171	Unique
Human	NA	locus113	Forward	13321	13480	0.996529	Unique
Bat	NA	locus67	Forward	13361	13480	0.997506	Unique
Human	NA	locus115	Forward	13561	13680	0.998005	Unique
Intermediate	ORF1b	locus107	Forward	13561	13760	0.995822	Unique
Human	NA	locus118	Reverse	13601	13720	0.995934	Unique
Intermediate	ORF1b	locus108	Reverse	13641	13760	0.997486	Unique
Bat	ORF1b	locus71	Reverse	13681	13800	0.997207	Unique
Human	ORF1b	locus121	Forward	13721	13840	0.998623	Shared
Bat	ORF1b	locus72	Forward	13721	13840	0.99815	Shared
Human	ORF1b	locus127	Forward	14521	14640	0.99744	Unique
Human	ORF1b	locus128	Forward	14681	14840	0.996292	Shared
Intermediate	ORF1b	locus113	Forward	14681	14840	0.995687	Shared

Bat	ORF1b	locus79	Forward	14721	14880	0.995612	Unique
Human	ORF1b	locus130	Forward	14761	14880	0.996073	Unique
Bat	ORF1b	locus84	Reverse	15601	15720	0.994162	Unique
Intermediate	ORF1b	locus126	Reverse	16721	16960	0.997734	Unique
Intermediate	ORF1b	locus127	Forward	16961	17200	0.994855	Unique
Human	ORF1b	locus138	Forward	17001	17200	0.998685	Unique
Bat	ORF1b	locus87	Forward	17081	17360	0.998644	Unique
Human	ORF1b	locus141	Reverse	17201	17320	0.996039	Unique
Human	ORF1b	locus142	Forward	17481	17640	0.997599	Unique
Intermediate	ORF1b	locus136	Forward	18201	18320	0.99607	Unique
Intermediate	ORF1b	locus139	Forward	19321	19440	0.998695	Unique
Intermediate	ORF1b	locus141	Forward	19361	19480	0.998613	Shared
Bat	ORF1b	locus94	Forward	19361	19480	0.998668	Shared
Human	ORF1b	locus150	Forward	19361	19520	0.998647	Unique
Human	ORF1b	locus151	Forward	19401	19520	0.995753	Common
Intermediate	ORF1b	locus144	Forward	19401	19520	0.996525	Common
Bat	ORF1b	locus96	Forward	19401	19520	0.997026	Common
Human	ORF1b	locus152	Forward	20201	20360	0.998509	Shared
Intermediate	ORF1b	locus145	Forward	20201	20360	0.99875	Shared
Intermediate	ORF1b	locus147	Forward	20281	20520	0.996655	Unique
Bat	ORF1b	locus101	Forward	20441	20600	0.998269	Unique
Intermediate	ORF1b	locus149	Reverse	20761	20880	0.998144	Unique
Human	ORF1b	locus158	Forward	20801	20920	0.998271	Unique
Bat	ORF1b	locus103	Forward	20801	21240	0.998766	Unique
Intermediate	ORF1b	locus150	Forward	20921	21080	0.997263	Unique
Intermediate	ORF1b	locus152	Forward	21081	21200	0.998404	Unique
Human	ORF1b	locus160	Forward	21081	21240	0.998312	Unique
Bat	ORF1b	locus104	Reverse	21121	21240	0.998156	Unique
Intermediate	ORF1b	locus157	Reverse	21361	21480	0.997372	Unique
Intermediate	S	locus158	Forward	21921	22040	0.996007	Unique
Bat	S	locus106	Reverse	21961	22080	0.992849	Unique
Bat	S	locus108	Forward	22361	22600	0.995938	Unique
Human	S	locus168	Forward	22481	22640	0.998582	Unique
Human	S	locus169	Reverse	22521	22640	0.998398	Shared
Intermediate	S	locus162	Forward	22521	22640	0.996589	Shared
Human	S	locus171	Reverse	22561	22680	0.998592	Unique
Intermediate	S	locus165	Reverse	22561	22720	0.997898	Unique
Human	S	locus176	Forward	22681	22800	0.998641	Unique
Intermediate	S	locus168	Forward	22681	23000	0.99733	Unique
Human	S	locus178	Forward	22721	22880	0.996028	Unique
Intermediate	S	locus170	Forward	23001	23120	0.995534	Unique
Intermediate	S	locus171	Forward	23641	23800	0.998515	Unique
Human	S	locus180	Forward	23681	23800	0.998532	Unique
Intermediate	S	locus174	Reverse	23881	24000	0.995462	Unique
Bat	S	locus112	Forward	23921	24040	0.998439	Unique

Bat	S	locus114	Forward	23961	24080	0.998802	Unique
Bat	ORF3a	locus118	Forward	25201	25440	0.997858	Unique
Bat	ORF3a	locus119	Reverse	25321	25440	0.996749	Unique
Human	ORF3a	locus190	Forward	25401	25560	0.998332	Unique
Human	ORF3a	locus191	Reverse	25441	25560	0.991688	Unique
Intermediate	ORF3a	locus182	Forward	25641	25800	0.995939	Unique
Human	ORF3a	locus192	Forward	25681	25800	0.997046	Shared
Intermediate	ORF3a	locus183	Reverse	25681	25800	0.996231	Shared
Human	ORF3a	locus193	Reverse	25681	25840	0.997059	Shared
Bat	ORF3a	locus121	Forward	25681	25840	0.998736	Shared
Intermediate	ORF3a	locus184	Forward	25721	25840	0.998559	Shared
Bat	ORF3a	locus122	Reverse	25721	25840	0.998777	Shared
Human	ORF3a	locus194	Forward	25761	25880	0.984922	Shared
Intermediate	ORF3a	locus186	Forward	25761	25880	0.998631	Shared
Bat	ORF3a	locus123	Forward	25761	25960	0.998832	Unique
Human	ORF3a	locus196	Forward	25801	25920	0.996034	Shared
Intermediate	ORF3a	locus188	Forward	25801	25920	0.998621	Shared
Human	ORF3a	locus198	Forward	25841	26000	0.998386	Shared
Intermediate	ORF3a	locus190	Forward	25841	26000	0.997869	Shared
Bat	ORF3a	locus125	Forward	25881	26000	0.998946	Unique
Human	ORF3a	locus200	Forward	25921	26040	0.998262	Shared
Intermediate	ORF3a	locus192	Forward	25921	26040	0.995968	Shared
Bat	ORF3a	locus127	Forward	25921	26120	0.998676	Unique
Human	ORF3a	locus202	Forward	25961	26080	0.993777	Unique
Intermediate	NA	locus194	Forward	25961	26200	0.992455	Unique
Human	ORF3a	locus204	Forward	26001	26120	0.99577	Shared
Bat	ORF3a	locus128	Reverse	26001	26120	0.998443	Shared
Bat	E	locus129	Forward	26121	26240	0.998296	Unique
Intermediate	E	locus196	Forward	26121	26280	0.998641	Unique
Human	E	locus210	Forward	26161	26280	0.998473	Unique
Bat	E	locus131	Forward	26161	26360	0.998649	Unique
Human	M	locus212	Forward	26201	26560	0.997338	Unique
Human	M	locus213	Reverse	26441	26560	0.996091	Shared
Intermediate	M	locus200	Forward	26441	26560	0.996856	Shared
Human	M	locus214	Forward	26481	26600	0.998155	Shared
Intermediate	M	locus202	Forward	26481	26600	0.998516	Shared
Human	M	locus216	Forward	26521	26640	0.997636	Unique
Human	M	locus217	Reverse	26521	26680	0.997245	Unique
Human	NA	locus222	Reverse	27241	27360	0.997392	Unique
Human	ORF7a	locus224	Reverse	27281	27440	0.997791	Unique
Intermediate	ORF7a	locus209	Reverse	27321	27440	0.997744	Unique
Human	ORF7a	locus226	Reverse	27361	27480	0.998475	Shared
Intermediate	ORF7a	locus210	Forward	27361	27480	0.998621	Shared
Intermediate	ORF8	locus215	Forward	27841	28120	0.997663	Unique
Bat	ORF8	locus148	Reverse	28081	28200	0.998741	Unique

Bat	N	locus149	Forward	28161	28280	0.994912	Unique
Intermediate	N	locus216	Forward	28161	28400	0.997703	Unique
Intermediate	N	locus217	Reverse	28281	28400	0.991812	Unique
Human	N	locus232	Forward	28721	28840	0.995337	Shared
Intermediate	N	locus219	Forward	28721	28840	0.994692	Shared
Intermediate	N	locus220	Forward	28921	29080	0.998539	Unique
Human	N	locus233	Forward	28961	29120	0.998651	Unique
Bat	ORF10	locus155	Forward	29481	29600	0.995642	Unique
Bat	ORF10	locus156	Reverse	29481	29640	0.996997	Unique

Table S6. RNA structures frequencies by selective restriction acting on each ORF in the three *Beta-CoVs*.

SARS-CoV: Selected RNA structures acting on each ORF

Host	ORF	Selection	Number of RNA structures
Bat	3UTR	Moderate	2
Bat	3UTR	Negative	6
Bat	5UTR	Negative	14
Bat	5UTR	Positive	2
Bat	E	Negative	16
Bat	M	Negative	7
Bat	M	Weak	1
Bat	N	Negative	16
Bat	ORF1a	Moderate	24
Bat	ORF1a	Negative	170
Bat	ORF1a	Positive	49
Bat	ORF1a	Weak	13
Bat	ORF1b	Moderate	7
Bat	ORF1b	Negative	78
Bat	ORF1b	Positive	9
Bat	ORF1b	Weak	10
Bat	ORF3a	Negative	32
Bat	ORF3b	Negative	8
Bat	S	Negative	32
Bat	Non-coding	Negative	16
Intermediate	5UTR	Negative	20
Intermediate	E	Negative	10
Intermediate	M	Negative	20
Intermediate	ORF1a	Moderate	12
Intermediate	ORF1a	Negative	494
Intermediate	ORF1a	Positive	3
Intermediate	ORF1a	Weak	11
Intermediate	ORF1b	Moderate	3
Intermediate	ORF1b	Negative	195
Intermediate	ORF1b	Weak	12
Intermediate	ORF3a	Negative	78
Intermediate	ORF3a	Positive	2
Intermediate	ORF3b	Negative	20
Intermediate	S	Negative	136
Intermediate	S	Positive	4
Human	5UTR	Negative	120
Human	E	Negative	86
Human	E	Positive	4

Human	M	Moderate	7
Human	M	Negative	98
Human	M	Positive	14
Human	M	Weak	1
Human	N	Negative	90
Human	ORF1a	Moderate	4
Human	ORF1a	Negative	1644
Human	ORF1a	Positive	1
Human	ORF1a	Weak	1
Human	ORF1b	Moderate	7
Human	ORF1b	Negative	773
Human	ORF3a	Negative	195
Human	ORF3a	Weak	15
Human	ORF7a	Negative	60
Human	S	Negative	450

MERS-CoV: Selected RNA structures acting on each ORF

Host	ORF	Selection	Number of RNA structures
Bat	M	Negative	15
Bat	N	Negative	9
Bat	ORF1a	Moderate	3
Bat	ORF1a	Negative	91
Bat	ORF1a	Positive	1
Bat	ORF1a	Weak	4
Bat	ORF1b	Negative	93
Bat	ORF3	Moderate	1
Bat	ORF3	Negative	2
Bat	ORF4a	Negative	6
Bat	ORF5	Negative	42
Bat	ORF5	Weak	6
Bat	S	Negative	40
Bat	S	Positive	1
Bat	S	Weak	1
Bat	Non-coding	Negative	18
Intermediate	5UTR	Negative	45
Intermediate	E	Negative	135
Intermediate	M	Negative	45
Intermediate	N	Negative	203
Intermediate	N	Positive	21
Intermediate	N	Weak	1
Intermediate	ORF1a	Moderate	52
Intermediate	ORF1a	Negative	2083
Intermediate	ORF1a	Positive	205
Intermediate	ORF1a	Weak	45
Intermediate	ORF1b	Negative	1297
Intermediate	ORF1b	Positive	7
Intermediate	ORF1b	Weak	1
Intermediate	ORF4b	Moderate	1
Intermediate	ORF4b	Negative	190
Intermediate	ORF4b	Positive	33
Intermediate	ORF4b	Weak	1
Intermediate	ORF5	Negative	269
Intermediate	ORF5	Weak	1
Intermediate	S	Moderate	56
Intermediate	S	Negative	899
Intermediate	S	Positive	2
Intermediate	S	Weak	33
Intermediate	Non-coding	Moderate	7

Intermediate	Non-coding	Negative	218
Human	5UTR	Negative	35
Human	E	Negative	105
Human	M	Negative	35
Human	N	Negative	119
Human	N	Positive	39
Human	N	Weak	17
Human	ORF1a	Moderate	55
Human	ORF1a	Negative	1645
Human	ORF1a	Positive	126
Human	ORF1a	Weak	99
Human	ORF1b	Moderate	28
Human	ORF1b	Negative	1018
Human	ORF1b	Positive	33
Human	ORF1b	Weak	41
Human	ORF4b	Negative	105
Human	ORF5	Moderate	48
Human	ORF5	Negative	138
Human	ORF5	Positive	7
Human	ORF5	Weak	17
Human	S	Moderate	9
Human	S	Negative	489
Human	S	Positive	26
Human	S	Weak	1
Human	Non-coding	Moderate	1
Human	Non-coding	Negative	174

SARS-CoV-2: Selected RNA structures acting on each ORF

Host	ORF	Selection	Number of RNA structures
Bat	5UTR	Negative	3
Bat	E	Negative	12
Bat	N	Negative	3
Bat	ORF10	Negative	9
Bat	ORF1a	Negative	37
Bat	ORF1a	Weak	2
Bat	ORF1b	Moderate	3
Bat	ORF1b	Negative	48
Bat	ORF1b	Weak	3
Bat	ORF3a	Moderate	1
Bat	ORF3a	Negative	39
Bat	ORF3a	Weak	2
Bat	ORF8	Negative	3
Bat	S	Negative	12
Bat	S	Positive	2
Bat	S	Weak	1
Bat	Non-coding	Negative	3
Intermediate	E	Negative	96
Intermediate	M	Moderate	2
Intermediate	M	Negative	92
Intermediate	M	Positive	1
Intermediate	M	Weak	1
Intermediate	N	Negative	286
Intermediate	N	Weak	2
Intermediate	ORF1a	Moderate	76
Intermediate	ORF1a	Negative	2339
Intermediate	ORF1a	Positive	39
Intermediate	ORF1a	Weak	42
Intermediate	ORF1b	Moderate	23
Intermediate	ORF1b	Negative	1148
Intermediate	ORF1b	Positive	5
Intermediate	ORF1b	Weak	24
Intermediate	ORF3a	Moderate	16
Intermediate	ORF3a	Negative	399
Intermediate	ORF3a	Weak	17
Intermediate	ORF7a	Negative	95
Intermediate	ORF7a	Weak	1
Intermediate	ORF8	Moderate	4
Intermediate	ORF8	Negative	231
Intermediate	ORF8	Weak	5

Intermediate	S	Moderate	16
Intermediate	S	Negative	541
Intermediate	S	Positive	16
Intermediate	S	Weak	3
Intermediate	Non-coding	Negative	84
Intermediate	Non-coding	Weak	12
Human	5UTR	Moderate	6
Human	5UTR	Negative	288
Human	E	Negative	98
Human	M	Moderate	6
Human	M	Negative	869
Human	M	Positive	3
Human	M	Weak	4
Human	N	Moderate	15
Human	N	Negative	277
Human	N	Positive	2
Human	ORF1a	Moderate	48
Human	ORF1a	Negative	5190
Human	ORF1a	Positive	12
Human	ORF1a	Weak	42
Human	ORF1b	Moderate	9
Human	ORF1b	Negative	1739
Human	ORF1b	Positive	9
Human	ORF1b	Weak	7
Human	ORF3a	Moderate	33
Human	ORF3a	Negative	1165
Human	ORF3a	Positive	39
Human	ORF3a	Weak	37
Human	ORF7a	Moderate	2
Human	ORF7a	Negative	274
Human	ORF7a	Weak	18
Human	S	Moderate	1
Human	S	Negative	780
Human	S	Positive	2
Human	S	Weak	3
Human	Non-coding	Moderate	2
Human	Non-coding	Negative	469
Human	Non-coding	Weak	17
