# Supplementary material for: RNA structure-altering mutations underlying positive selection on Spike protein reveal novel putative signatures to trace crossing host-species barriers in Betacoronavirus
Source: RNA Biol. 2022 Sep 14;19(1):1019–44. doi: 10.1080/15476286.2022.2115750 (PMC9481089; doi:10.1080/15476286.2022.2115750)
Supplement: Supplemental Material [file KRNB_A_2115750_SM9797.zip › Supplementary_Material.pdf]

# Non-compensatory mutations in Spike protein positively selected on RNA structures might be signatures to trace cross-species barrier jumps of *Betacoronavirus*

Alexis Felipe Rojas-Cruz [1], Juan Carlos Gallego-Gómez [2] & Clara Isabel Bermúdez-Santana [1,3]

[1] Grupo RNomica Teórica y Computacional, Department of Biology, Faculty of Sciences, National University of Colombia, Bogota, 111311, Colombia.

[2] Grupo Medicina Molecular y de Translación, Faculty of Medicine, University of Antioquia, Medellin, 050010, Colombia.

[3] Center of Excellence in Scientific Computing, National University of Colombia, Bogota, 111311, Colombia.

## Supplementary Material

### Figures

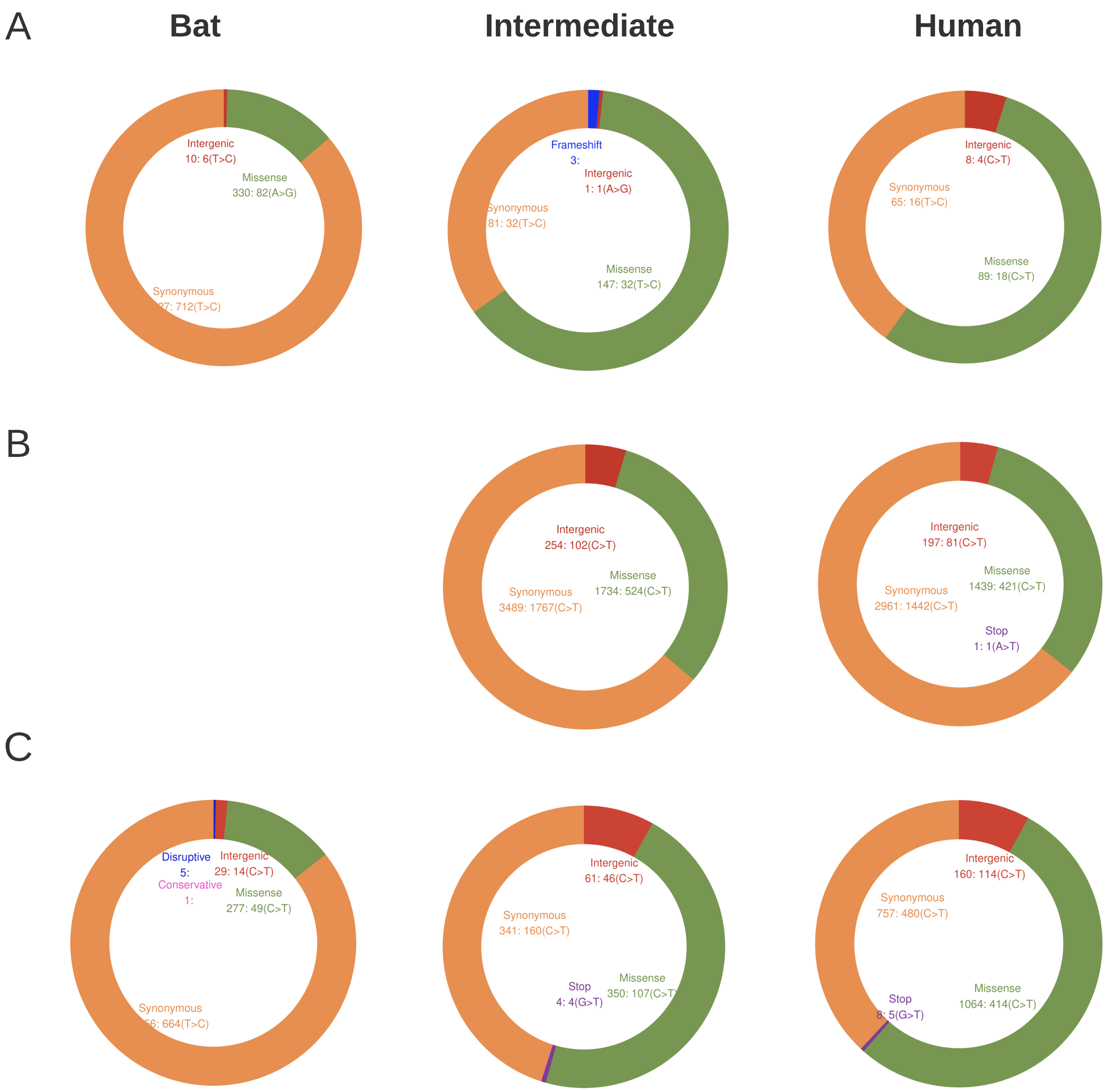


**Figure S1.** Prevalence and distribution of mutation types in the representative *Beta-CoVs* genomes for different hosts. **(A)** Donut plots show the stratified distribution of variations in SARS-CoV, 86.2% of total mutations correspond for bat-associated virus, being synonymous mutations the majority (93.6%); **(B)** MERS-CoV genomes harbor the highest number of mutations detected relative to the reference sequence NC_019843 comprising mostly of synonymous and missense for intermediate and human host, and **(C)** SARS-CoV-2 shows that, like SARS-CoV, virus found in bats still represents the highest percentage of mutations (44.12%), followed by human (40.9%). The color codes represent the type of mutations as well as nucleotide change frequency.

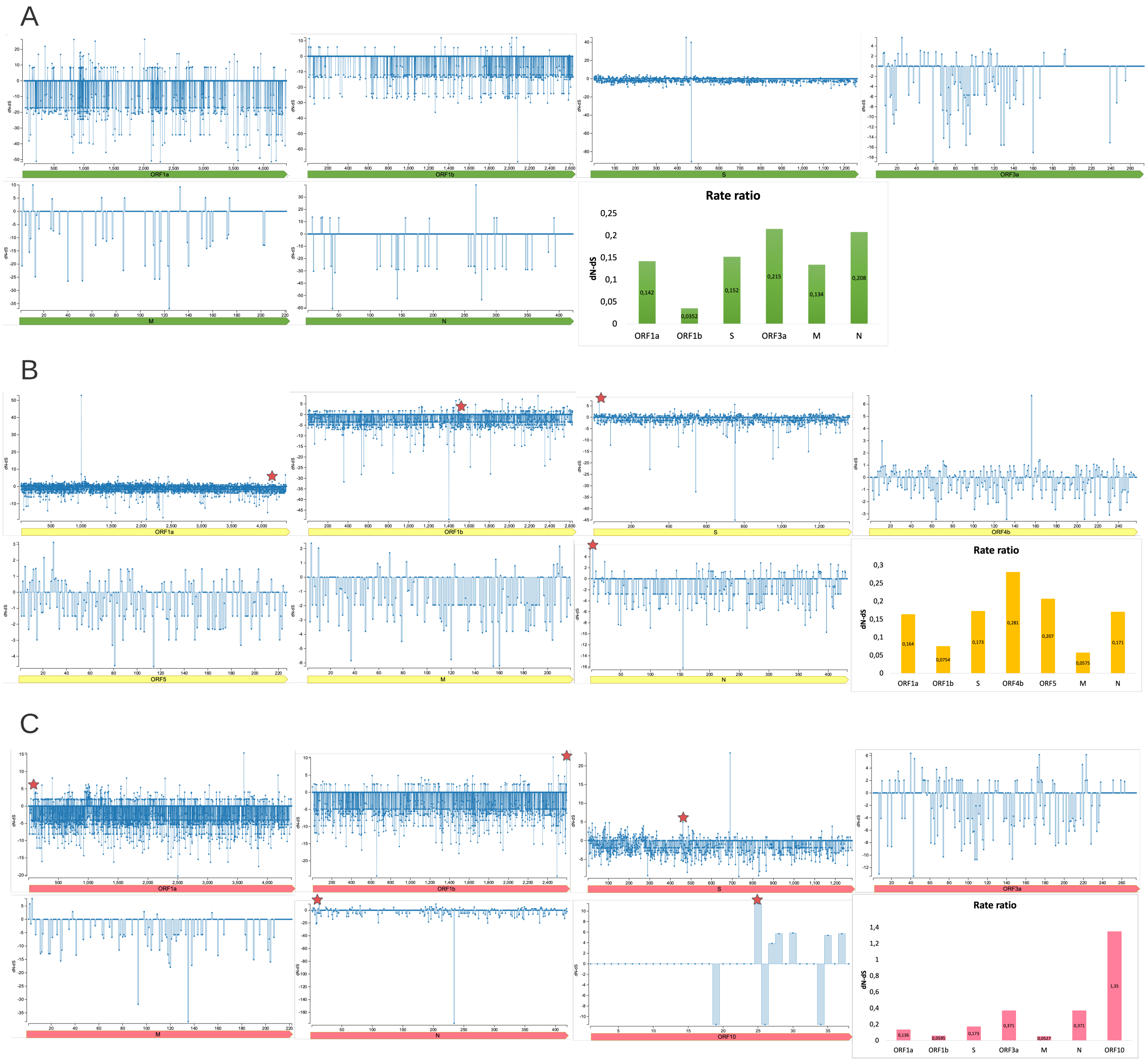


**Figure S2.** Diversifying and purifying selection in representative *Beta-CoV* genomes. Overview obtained by SLAC analysis, illustrating the evolution rate (dN-dS or dN/dS) for individual genes of **(A)** SARS-CoV; **(B)** MERS-CoV; and **(C)** SARS-CoV-2. Statistically significant codons are inferred from the multiple evolutionary tests used in this study. Horizontal bar illustrates CDS under some natural selection and red asterisks represent codons with significant evidence of positive selection whose values are found in Tables 1 and 2.

# Tables

**Table S1**. Detailed report of viral sequences found in all organisms analyzed in the study

| Virus | Host | Database | Specie | Sequences per specie | Raw sequences | Total raw sequences | Total |
|---|---|---|---|---|---|---|---|
| SARS-CoV | Bat | NCBI Virus | *Aselliscus stoliczkanus* | 1 | 33 | 57 | 1252952 |
| | | | *Chaerephon plicatus* | 1 | | | |
| | | | *Chiroptera* | 2 | | | |
| | | | *Rhinolophus affinis* | 1 | | | |
| | | | *Rhinolophus ferrumequinu* | 7 | | | |
| | | | *Rhinolophus macrotis* | 1 | | | |
| | | | *Rhinolophus pusillus* | 3 | | | |
| | | | *Rhinolophus sinicus* | 16 | | | |
| | | | *Rhinolophus* | 1 | | | |
| | | ViPR | *Aselliscus stoliczkanus* | 1 | 23 | | |
| | | | *Bat* | 1 | | | |
| | | | *Rhinolophus affinis* | 1 | | | |
| | | | *Rhinolophus ferrumequinu* | 3 | | | |
| | | | *Rhinolophus monoceros* | 1 | | | |
| | | | *Rhinolophus pusillus* | 2 | | | |
| | | | *Rhinolophus sinicus* | 13 | | | |
| | | | *Rhinolophus* | 1 | | | |
| | | ViruSurf | *Chiroptera* | 1 | 1 | | |
| | Intermediate | NCBI Virus | *Chlorocebus aethiops* | 1 | 52 | 110 | |
| | | | *Mus musculus* | 41 | | | |
| | | | *Paguma larvata* | 1 | | | |
| | | | *Paradoxurus hermaphrod* | 6 | | | |
| | | | *Viverridae* | 3 | | | |
| | | ViPR | *Chlorocebus aethiops* | 1 | 57 | | |
| | | | *Badger* | 1 | | | |
| | | | *Mus musculus* | 40 | | | |
| | | | *Paguma larvata* | 15 | | | |
| | | ViruSurf | *Palm civet* | 1 | 1 | | |
| | Human | NCBI Virus | *Homo sapiens* | 12 | 12 | 86 | |
| | | ViPR | *Homo sapiens* | 73 | 73 | | |
| | | ViruSurf | *Homo sapiens* | 1 | 1 | | |
| MERS-CoV | Bat | NCBI Virus | *Hypsugo savii* | 1 | 5 | 11 | |
| | | | *Neoromicia capensis* | 2 | | | |
| | | | *Pipistrellus kuhlii* | 1 | | | |
| | | | *Vespertilio sinensis* | 1 | | | |
| | | ViPR | *Hypsugo savii* | 1 | 3 | | |
| | | | *Neoromicia capensis* | 1 | | | |
| | | | *Pipistrellus kuhlii* | 1 | | | |
| | | ViruSurf | *Hypsugo savii* | 1 | 3 | | |
| | | | *Neoromicia capensis* | 1 | | | |
| | | | *Pipistrellus kuhlii* | 1 | | | |
| | Intermediate | NCBI Virus | *Camelus bactrianus* | 1 | 337 | 872 | |
| | | | *Camelus dromedarius* | 258 | | | |
| | | | *Camelus* | 77 | | | |
| | | | *Lama glama* | 1 | | | |
| | | ViPR | *Camelus dromedarius* | 200 | 205 | | |
| | | | *Camelus* | 5 | | | |
| | | ViruSurf | *Camelus dromedarius* | 253 | 330 | | |
| | | | *Camelus* | 76 | | | |
| | | | *Lama glama* | 1 | | | |
| | Human | NCBI Virus | *Homo sapiens* | 257 | 257 | 643 | |
| | | ViPR | *Homo sapiens* | 134 | 134 | | |

| | | | | | | | |
|---|---|---|---|---|---|---|---|
| | | ViruSurf | *Homo sapiens* | 252 | 252 | | |
| SARS-CoV-2 | Bat | NCBI Virus | *Rhinolophus affinis* | 1 | 2 | 7 | |
| | | | *Rhinolophus cornutus* | 1 | | | |
| | | GISAID | *Rhinolophus affinis* | 1 | 5 | | |
| | | | *Rhinolophus malayanus* | 2 | | | |
| | | | *Rhinolophus shameli* | 2 | | | |
| | Intermediate | NCBI Virus | *Canis lupus* | 2 | 54 | 515 | |
| | | | *Felis catus* | 10 | | | |
| | | | *Feliformia* | 1 | | | |
| | | | *Mesocricetus auratus* | 5 | | | |
| | | | *Mus musculus* | 1 | | | |
| | | | *Mustela lutreola* | 13 | | | |
| | | | *Neovision vision* | 12 | | | |
| | | | *Panthera leo* | 4 | | | |
| | | | *Panthera tigris* | 6 | | | |
| | | ViPR | *Canis famiaris* | 1 | 43 | | |
| | | | *Canino* | 1 | | | |
| | | | *Cat* | 2 | | | |
| | | | *Felis catus* | 4 | | | |
| | | | *Mesocricetus auratus* | 4 | | | |
| | | | *Mustela lutreola* | 13 | | | |
| | | | *Neovision vision* | 9 | | | |
| | | | *Panthera leo* | 4 | | | |
| | | | *Panthera tigris* | 5 | | | |
| | | ViruSurf | *Canis familiaris* | 1 | 54 | | |
| | | | *Canino* | 1 | | | |
| | | | *Environment* | 1 | | | |
| | | | *Felis catus* | 10 | | | |
| | | | *Feline* | 1 | | | |
| | | | *Mesocricetus auratus* | 5 | | | |
| | | | *Mustela lutreola* | 13 | | | |
| | | | *Neovision vision* | 12 | | | |
| | | | *Panthera leo* | 4 | | | |
| | | | *Panthera tigris* | 6 | | | |
| | | GISAID | *Canis lupus* | 4 | 364 | | |
| | | | *Chlorocebus sabaeus* | 1 | | | |
| | | | *Environment* | 24 | | | |
| | | | *Felis catus* | 16 | | | |
| | | | *Gorilla* | 1 | | | |
| | | | *Manis javanica* | 12 | | | |
| | | | *Mink* | 7 | | | |
| | | | *Mustela lutreola* | 13 | | | |
| | | | *Mus musculus* | 1 | | | |
| | | | *Neovision vision* | 275 | | | |
| | | | *Panthera leo* | 4 | | | |
| | | | *Panthera tigris* | 6 | | | |
| | Human | NCBI Virus | *Homo sapiens* | 69536 | 69536 | 1250651 | |
| | | ViPR | *Homo sapiens* | 58284 | 58284 | | |
| | | ViruSurf | *Homo sapiens* | 32083 | 32083 | | |
| | | GISAID | *Homo sapiens* | 1090748 | 1090748 | | |

**Table S2**. Prediction performance of the GOFIX method on the known ORFs in the Beta-CoVs genomes

CDS annotations extracted from Genbank, with ORF names standardized according to the nomenclature for each virus

| **Virus** | **ORF1a** | **ORF1b** | **S** | **ORF3** | **ORF3a** | **ORF3b** | **ORF4a** | **ORF4b** | **ORF5** | **E** | **M** | **ORF6** | **ORF7a** | **ORF7b** | **ORF8** | **ORF8a** | **ORF8b** | **N** | **ORF9b** | **ORF9c** | **ORF10** |
|---|---|---|---|---|---|---|---|---|---|---|---|---|---|---|---|---|---|---|---|---|---|
| **SARS-CoV *** | 13134 | 7887 | 3768 | | 825 | 465 | | | | 231 | 666 | 192 | 369 | 135 | | 129 | 255 | 1269 | 297 | 211 | |
| **MERS-CoV **** | 13176 | 7872 | 4062 | 312 | | | 330 | 741 | 675 | 249 | 660 | | | | | | 339 | 1242 | | | |
| **SARS-CoV-2 ***** | 13218 | 7788 | 3849 | | 828 | 172 | | | | 228 | 669 | 186 | 366 | 132 | 366 | | | 1260 | 294 | 222 | 117 |

* NCBI Reference Sequence: NC_004718.3 (https://www.ncbi.nlm.nih.gov/nuccore/NC_004718)

** NCBI Reference Sequence: NC_019843.3 (https://www.ncbi.nlm.nih.gov/nuccore/NC_019843)

*** NCBI Reference Sequence: NC_045512.2 (https://www.ncbi.nlm.nih.gov/nuccore/1798174254)

Prediction performance of the GOFIX method on the set of known ORFs in the SARS-CoV genome

| Bat | | | |
|---|---|---|---|
| **ORF** | **Start** | **End** | **Length** |
| 5UTR | 0 | 264 | 264 |
| ORF1a | 265 | 13413 | 13149 |
| ORF1b | 13599 | 21485 | 7887 |
| S | 21492 | 25217 | 3726 |
| ORF3a | 25227 | 26051 | 825 |
| ORF3b | 25648 | 26112 | 465 |
| E | 26076 | 26306 | 231 |
| M | 26357 | 27022 | 666 |
| ORF6 | 27033 | 27224 | 192 |
| ORF7a | 27232 | 27600 | 369 |
| ORF7b | 27597 | 27731 | 135 |
| ORF8 | 27800 | 28103 | 303 |
| | | | |
| N | 28118 | 29386 | 1269 |
| ORF9b | 28128 | 28424 | 297 |
| ORF9c | 28581 | 28793 | 213 |
| 3UTR | 29387 | 29725 | 339 |

| Intermediate | | | |
|---|---|---|---|
| **ORF** | **Start** | **End** | **Length** |
| 5UTR | 0 | 213 | 213 |
| ORF1a | 217 | 13365 | 13149 |
| ORF1b | 13551 | 21437 | 7887 |
| S | 21444 | 25211 | 3768 |
| ORF3a | 25220 | 26044 | 825 |
| ORF3b | 25641 | 26105 | 465 |
| E | 26069 | 26299 | 231 |
| M | 26350 | 27015 | 666 |
| ORF6 | 27026 | 27217 | 192 |
| ORF7a | 27225 | 27593 | 369 |
| ORF7b | | 27724 | 135 |
| ORF8 | 27778 | 28015 | 237 |
| | | | |
| N | 28100 | 29368 | 1269 |
| ORF9b | 28110 | 28406 | 297 |
| ORF9c | 28563 | 28775 | 213 |
| 3UTR | 29369 | 29707 | 339 |

| Human | | | |
|---|---|---|---|
| **ORF** | **Start** | **End** | **Length** |
| 5UTR | 0 | 264 | 264 |
| ORF1a | 265 | 13413 | 13149 |
| ORF1b | 13599 | 21485 | 7887 |
| S | 21492 | 25259 | 3768 |
| ORF3a | 25268 | 26092 | 825 |
| ORF3b | 25689 | 26153 | 465 |
| E | 26117 | 26347 | 231 |
| M | 26398 | 27063 | 666 |
| ORF6 | 27074 | 27265 | 192 |
| ORF7a | 27273 | 27641 | 369 |
| ORF7b | 27638 | 27772 | 135 |
| ORF8a | 27778 | 27907 | 129 |
| ORF8b | 27863 | 28121 | 258 |
| N | 28120 | 29388 | 1269 |
| ORF9b | 28130 | 28426 | 297 |
| ORF9c | 28583 | 28795 | 213 |
| 3UTR | 29389 | 29727 | 339 |

Prediction performance of the GOFIX method on the set of known ORFs in the MERS-CoV genome

| Bat | | | |
|---|---|---|---|
| **ORF** | **Start** | **End** | **Length** |
| 5UTR | 0 | 206 | 206 |
| ORF1a | 207 | 13376 | 13170 |
| ORF1b | 13565 | 21436 | 7872 |
| S | 21379 | 25416 | 4038 |
| ORF3 | 25428 | 25739 | 312 |
| ORF4a | 25748 | 26036 | 288 |
| ORF4b | 25953 | 26714 | 762 |
| ORF5 | 26722 | 27405 | 684 |
| E | 27484 | 27741 | 258 |
| M | 27746 | 28402 | 657 |
| ORF8b | 28643 | 28982 | 339 |
| N | 28450 | 29739 | 1290 |
| 3UTR | 29740 | 30028 | 289 |

| Intermediate | | | |
|---|---|---|---|
| **ORF** | **Start** | **End** | **Length** |
| 5UTR | 0 | | 278 |
| ORF1a | 279 | 13454 | 13176 |
| ORF1b | 13643 | 21514 | 7872 |
| S | 21456 | 25517 | 4062 |
| ORF3 | 25532 | 25843 | 312 |
| ORF4a | 25852 | 26181 | 330 |
| ORF4b | 26093 | 26833 | 741 |
| ORF5 | 26840 | 27514 | 675 |
| E | 27590 | 27838 | 249 |
| M | 27853 | 28512 | 660 |
| ORF8b | | 29100 | 339 |
| N | 28566 | 29807 | 1242 |
| 3UTR | 29808 | 30106 | 300 |

| Human | | | |
|---|---|---|---|
| **ORF** | **Start** | **End** | **Length** |
| 5UTR | 0 | 278 | 278 |
| ORF1a | 279 | 13454 | 13176 |
| ORF1b | 13643 | 21514 | 7872 |
| S | 21456 | 25517 | 4062 |
| ORF3 | 25532 | 25843 | 312 |
| ORF4a | 25852 | 26181 | 330 |
| ORF4b | 26093 | 26833 | 741 |
| ORF5 | 26840 | 27514 | 675 |
| E | 27590 | 27838 | 249 |
| M | 27853 | 28512 | 660 |
| ORF8b | 28762 | 29100 | 339 |
| N | 28566 | 29807 | 1242 |
| 3UTR | 29808 | 30106 | 299 |

Prediction performance of the GOFIX method on the set of known ORFs in the SARS-CoV-2 genome

| Bat | | | |
|---|---|---|---|
| **ORF** | **Start** | **End** | **Length** |
| 5UTR | 0 | 259 | 259 |
| ORF1a | 260 | 13456 | 13197 |
| ORF1b | 13741 | 21528 | 7788 |
| S | 21535 | 25290 | 3756 |
| ORF3a | 25299 | 26126 | 828 |
| E | 26151 | 26378 | 228 |
| M | 26429 | 27094 | 666 |
| ORF6 | 27105 | 27290 | 186 |
| ORF7a | 27297 | 27662 | 366 |
| ORF7b | 27659 | 27790 | 132 |
| ORF8 | 27869 | 28234 | 366 |
| N | 28249 | 29508 | 1260 |
| ORF9b | 28259 | 28552 | 294 |
| ORF9c | 28709 | 28930 | 222 |
| ORF10 | 29533 | 29661 | 129 |
| 3UTR | 29662 | 29844 | 183 |

| Intermediate | | | |
|---|---|---|---|
| **ORF** | **Start** | **End** | **Length** |
| 5UTR | 0 | 211 | 211 |
| ORF1a | 212 | 13429 | 13218 |
| ORF1b | 13714 | 21501 | 7788 |
| S | 21482 | 25330 | 3849 |
| ORF3a | 25339 | 26166 | 828 |
| E | 26191 | 26418 | 228 |
| M | 26469 | 27137 | 669 |
| ORF6 | 27148 | 27333 | 186 |
| ORF7a | 27340 | 27705 | 366 |
| ORF7b | 27702 | 27833 | 132 |
| ORF8 | 27840 | 28205 | 366 |
| N | | 29479 | 1260 |
| ORF9b | 28230 | 28523 | 294 |
| ORF9c | 28680 | 28901 | 222 |
| ORF10 | 29504 | 29632 | 129 |
| 3UTR | 29633 | 29774 | 142 |

| Human | | | |
|---|---|---|---|
| **ORF** | **Start** | **End** | **Length** |
| 5UTR | 0 | 237 | 237 |
| ORF1a | 238 | 13455 | 13218 |
| ORF1b | 13740 | 21527 | 7788 |
| S | 21535 | 25356 | 3849 |
| ORF3a | 25365 | 26192 | 828 |
| E | 26217 | 26444 | 228 |
| M | 26495 | 27163 | 669 |
| ORF6 | 27174 | 27359 | 186 |
| ORF7a | 27366 | 27731 | 366 |
| ORF7b | 27728 | 27859 | 132 |
| ORF8 | 27866 | 28231 | 366 |
| N | 28246 | 29505 | 1260 |
| ORF9b | 28256 | 28549 | 294 |
| ORF9c | 28706 | 28927 | 222 |
| ORF10 | 29530 | 29658 | 129 |
| 3UTR | 29659 | 29822 | 164 |

**Table S3.** Natural selection and inferred substitutions of predicted CDSs for *Beta-CoVs*

Sites under natural selection within the SARS-CoV CDSs

| CDS | Test | Negative/Purifyng | | | Positive/Diversifying | | |
|---|---|---|---|---|---|---|---|
| | | **Sites** | **Model** | **Ratio dN/dS** | **Sites** | **Model** | **Ratio dN/dS** |
| ORF1a | SLAC | 2 | GTR | 0.142 | | | |
| | FEL | 94 | GTR | 0.12 | | | |
| | MEME | | | | 10 | GTR | 0.12 |
| | FUBAR | 151 | GTR | | | | |
| ORF1b | SLAC | 1 | GTR | 0.0352 | | | |
| | FEL | 139 | GTR | 0.0281 | | | |
| | MEME | | | | 5 | GTR | 0.0281 |
| | FUBAR | 311 | GTR | | | | |
| S | SLAC | 24 | GTR | 0.152 | | | |
| | FEL | 182 | GTR | 0.121 | | | |
| | MEME | | | | 6 | GTR | 0.121 |
| | FUBAR | 350 | GTR | | | | |
| ORF3a | SLAC | 1 | GTR | 0.215 | | | |
| | FEL | 17 | GTR | 0.191 | | | |
| | MEME | | | | 1 | GTR | 0.191 |
| | FUBAR | 15 | GTR | | | | |
| ORF3b * | SLAC | | | | | | |
| | FEL | | | | | | |
| | MEME | | | | | | |
| | FUBAR | | | | | | |
| E | SLAC | 0 | GTR | 0.00 | | | |
| | FEL | 0 | GTR | 0.00 | | | |
| | MEME | | | | 0 | GTR | 0.00 |
| | FUBAR | 0 | GTR | | | | |
| M | SLAC | 1 | GTR | 0.134 | | | |
| | FEL | 11 | GTR | 0.121 | | | |
| | MEME | | | | 0 | GTR | 0.121 |
| | FUBAR | 11 | GTR | | | | |
| ORF6 | SLAC | 0 | GTR | 0.109 | | | |
| | FEL | 0 | GTR | 0.101 | | | |
| | MEME | | | | 0 | GTR | 0.101 |
| | FUBAR | 0 | GTR | | | | |
| ORF7a | SLAC | 0 | GTR | 0.0876 | | | |
| | FEL | 4 | GTR | 0.0770 | | | |
| | MEME | | | | 0 | GTR | 0.0770 |
| | FUBAR | 3 | GTR | | | | |
| ORF7b | SLAC | 0 | GTR | 0.506 | | | |
| | FEL | 1 | GTR | 0.493 | | | |
| | MEME | | | | 0 | GTR | 0.493 |
| | FUBAR | 0 | GTR | | | | |
| ORF8 *** | SLAC | | | | | | |
| | FEL | | | | | | |
| | MEME | | | | | | |
| | FUBAR | | | | | | |
| N | SLAC | 0 | GTR | 0.208 | | | |
| | FEL | 5 | GTR | 0.175 | | | |
| | MEME | | | | 1 | GTR | 0.175 |
| | FUBAR | 3 | GTR | | 1 | GTR | |
| ORF9b ** | SLAC | | | | | | |
| | FEL | | | | | | |
| | MEME | | | | | | |
| | FUBAR | | | | | | |
| ORF9c | SLAC | 0 | GTR | 1.42 | | | |
| | FEL | 0 | GTR | 1.47 | | | |
| | MEME | | | | 0 | GTR | 1.47 |
| | FUBAR | 0 | GTR | | | | |

| Colour | Legend |
|---|---|
| (green) | Mutation |
| (yellow) | No sites |
| (orange) | Cleave |

* Reported mutation: (343C => 343T; 344T => 344A) in (B1-AS-CHA, B2-RS-CHA, B3-RS-CHA, B4-RS-CHA).
** Reported mutacion: 55C => 55T in (I1-PL-CHA, I2-PL-CHA)
*** In Human ORF8 is cleaved into ORF8a and ORF8b.

| Negative/Purifyng | | | | | | | | | | | |
|---|---|---|---|---|---|---|---|---|---|---|---|
| **Gene** | **Gene codon position** | **SLAC** | | **FEL** | | **FUBAR** | | **Inferred substitution** | | | **Amino acid** |
| | | **dN-dS** | **P[dN/dS < 1]** | **dN/dS** | **p-value** | **dN-dS** | **Prob[dS>dN]** | **Bat** | **Intermediate** | **Human** | |
| **ORF1a** | 2275 | -514.34 | 0.037 | 0.0 | 0.002 | -32.846 | 0.999 | TCT, TCG | TCC | TCC | S |
| | 4131 | -514.34 | 0.037 | 0.0 | 0.005 | -27.241 | 0.999 | TCT, TCC, TCA | TCC | TCC | S |
| **ORF1b** | 1264 | -36.19 | 0.035 | 0.0 | 0.0 | -36.69 | 1.0 | CCT, CCG, CCC | CCC, CCT | CCC | P |
| **S** | 84 | -7.03 | 0.044 | 0.0 | 0.002 | -11.24 | 0.995 | AAT, AAC | AAC | AAC | N |
| | 91 | -6.67 | 0.048 | 0.0 | 0.003 | -9.912 | 0.994 | GAC, GAT | GAT | GAT | D |
| | 96 | -6.64 | 0.037 | 0.0 | 0.001 | -13.699 | 0.998 | GCA, GCT, GCC | GCT | GCT | A |
| | 163 | -9.14 | 0.036 | 0.011 | 0.01 | -20.429 | 0.994 | TTC, TTT ǀ TAC, TAT | TTT | TTT | F ǀ Y, F |
| | 264 | -8.54 | 0.038 | 0.0 | 0.003 | -14.104 | 0.997 | AAA, AAG | AAG | AAG | K |
| | 331 | -10.08 | 0.010 | 0.0 | 0.0 | -21.509 | 0.999 | TTT, TTC | TTT | TTT | F |
| | 347 | -8.54 | 0.034 | 0.0 | 0.003 | -14.111 | 0.997 | GAG, GAA | GAG | GAG | E |
| | 348 | -9.00 | 0.019 | 0.0 | 0.0 | -19.52 | 0.999 | AGA, CGA | AGG | AGA, AGG | R |
| | 422 | -6.98 | 0.044 | 0.0 | 0.005 | -9.268 | 0.994 | TTT, TTC | TTC | TTC | F |
| | 430 | -6.71 | 0.048 | 0.0 | 0.008 | -7.138 | 0.991 | AAC, AAT | AAT | AAT | N |
| | 460 | -6.98 | 0.044 | 0.0 | 0.004 | -9.368 | 0.993 | GAC, GAT | GAC | GAC | D |
| | 560 | -6.67 | 0.048 | 0.0 | 0.003 | -9.912 | 0.994 | GAC, GAT | GAT | GAT | D |
| | 624 | -6.64 | 0.037 | 0.0 | 0.0 | -22.918 | 0.999 | GCG, GCT | GCT | GCT | A |
| | 733 | -6.71 | 0.048 | 0.0 | 0.008 | -7.171 | 0.991 | GAC, GAT | GAT | GAT | D |
| | 746 | -7.03 | 0.044 | 0.0 | 0.001 | -12.131 | 0.995 | AGT, AGC | AGC | AGC | S |
| | 808 | -6.98 | 0.044 | 0.0 | 0.004 | -9.368 | 0.993 | GAC, GAT | GAC | GAC | D |
| | 902 | -6.71 | 0.048 | 0.0 | 0.008 | -7.138 | 0.991 | AAC, AAT | AAT | AAT | N |
| | 913 | -6.98 | 0.044 | 0.0 | 0.004 | -9.33 | 0.994 | AAC, AAT | AAC | AAC | N |
| | 1136 | -10.45 | 0.009 | 0.0 | 0.0 | -19.424 | 0.999 | TTT, TTC | TTC | TTC | F |
| | 1150 | -6.64 | 0.037 | 0.0 | 0.0 | -22.91 | 0.999 | CCA, CCT | CCA | CCA | P |
| | 1175 | -7.49 | 0.038 | 0.0 | 0.0 | -21.364 | 0.999 | AAC, AAT | AAT | AAT | N |
| | 1201 | -6.64 | 0.037 | 0.0 | 0.0 | -24.817 | 1.0 | CCA, CCT, CCC | CCT | CCT | P |
| | 1247 | -6.67 | 0.048 | 0.0 | 0.007 | -8.162 | 0.991 | GAC, GAT | GAT | GAT | D |
| | 1254 | -8.66 | 0.037 | 0.0 | 0.002 | -16.394 | 0.997 | AAA, AAG | AAG | AAG | K |
| **ORF3a** | 9 | -17.05 | 0.037 | 0.0 | 0.03 | -28.454 | 0.999 | ACA, ACG | ACT | ACT | T |
| **M** | 124 | -36.87 | 0.031 | 0.0 | 0.006 | -38.601 | 0.998 | AGG, CGA | CGG | CGG | R |
| **N** | 277 | -53.40 | 0.010 | 0.0 | 0.021 | -32.85 | 0.991 | AGG | GAC, GCC | GAC | R |

Sites under natural selection within the MERS-CoV CDSs

| CDS | Test | Negative/Purifyng | | | Positive/Diversifying | | |
|---|---|---|---|---|---|---|---|
| | | Sites | Model | Ratio dN/dS | Sites | Model | Ratio dN/dS |
| ORF1a | SLAC | 65 | GTR | 0.164 | 1 | GTR | 0.164 |
| | FEL | 495 | GTR | 0.133 | 3 | GTR | 0.133 |
| | MEME | | | | 32 | GTR | 0.133 |
| | FUBAR | 1064 | GTR | | 9 | GTR | |
| ORF1b | SLAC | 29 | GTR | 0.0754 | 1 | GTR | 0.0754 |
| | FEL | 190 | GTR | 0.0580 | 2 | GTR | 0.0580 |
| | MEME | | | | 12 | GTR | 0.0580 |
| | FUBAR | 857 | GTR | | 4 | GTR | |
| S | SLAC | 31 | GTR | 0.173 | 1 | GTR | 0.173 |
| | FEL | 183 | GTR | 0.114 | 2 | GTR | 0.114 |
| | MEME | | | | 10 | GTR | 0.114 |
| | FUBAR | 137 | GTR | | 4 | GTR | |
| ORF3 | SLAC | 0 | GTR | 0.366 | | | |
| | FEL | 7 | GTR | 0.346 | | | |
| | MEME | | | | 0 | GTR | 0.346 |
| | FUBAR | 1 | GTR | | 1 | GTR | |
| ORF4a | SLAC | 0 | GTR | 0.270 | | | |
| | FEL | **10** | GTR | 0.185 | | | |
| | MEME | | | | 1 | GTR | 0.185 |
| | FUBAR | 4 | GTR | | 1 | GTR | |
| ORF4b | SLAC | 2 | GTR | 0.281 | | | |
| | FEL | 29 | GTR | 0.223 | | | |
| | MEME | | | | 0 | GTR | 0.223 |
| | FUBAR | 10 | GTR | | 1 | GTR | |
| ORF5 | SLAC | 2 | GTR | 0.207 | | | |
| | FEL | 22 | GTR | 0.163 | | | |
| | MEME | | | | 0 | GTR | 0.163 |
| | FUBAR | 12 | GTR | | 2 | GTR | |
| E | SLAC | 0 | GTR | 0.209 | | | |
| | FEL | 6 | GTR | 0.199 | | | |
| | MEME | | | | 0 | GTR | 0.199 |
| | FUBAR | 0 | GTR | | | | |
| M | SLAC | 2 | GTR | 0.0575 | | | |
| | FEL | 37 | GTR | 0.0367 | | | |
| | MEME | | | | 2 | GTR | 0.0367 |
| | FUBAR | 12 | GTR | | 1 | GTR | |
| ORF8b | SLAC | 0 | GTR | 3.09 | | | |
| | FEL | 0 | GTR | 3.24 | | | |
| | MEME | | | | 0 | GTR | 3.24 |
| | FUBAR | 0 | GTR | | | | |
| N | SLAC | 7 | GTR | 0.171 | 1 | GTR | 0.171 |
| | FEL | 38 | GTR | 0.143 | 1 | GTR | 0.143 |
| | MEME | | | | 1 | GTR | 0.143 |
| | FUBAR | 21 | GTR | | 1 | GTR | |

Positive
No sites

| Negative/Purifyng | | | | | | | | | | | |
|---|---|---|---|---|---|---|---|---|---|---|---|
| **Gene** | **Gene codon positio** | **SLAC** | | **FEL** | | **FUBAR** | | **Inferred substitution** | | | **Amino acid** |
| | | **dN-dS** | **P[dN/dS < 1]** | **dN/dS** | **p-value** | **dN-dS** | **Prob[dS>dN]** | **Bat** | **Intermediate** | **Human** | |
| **ORF1a** | 47 | -1.34 | 0.00045 | 0.0 | 0.0 | -47.93 | 1.0 | CCA | CCA, CCG | CCA | P |
| | 88 | -115.47 | 0.0013 | 0.0 | 0.0 | -44.53 | 1.0 | GTC, GTT | GTG, GTA | GTG, GTA | V |
| | 103 | -602.34 | 0.045 | 0.0 | 0.007 | -4.366 | 0.984 | TTT | TTC | TTC, TTT | F |
| | 118 | -1.13 | 0.0017 | 0.0 | 0.0 | -46.699 | 1.0 | CTC, CTT | CTG, TTG, CT | CTG, TTG, CTT | L |
| | 132 | -6.02 | 0.045 | 0.0 | 0.007 | -4.37 | 0.984 | TTT | TTT, TTC | TTC | F |
| | 175 | -6.12 | 0.043 | 0.0 | 0.004 | -5.945 | 0.982 | GAC | GAT | GAT, GAC | D |
| | 301 | -577.37 | 0.037 | 0.0 | 0.001 | -6.926 | 0.991 | CGT | CGC, CGT | CGC, CGT | R |
| | 320 | -9.03 | 0.0096 | 0.0 | 0.001 | -10.951 | 0.993 | ACA | ACC | ACC | T |
| | 334 | -6.02 | 0.045 | 0.0 | 0.005 | -4.354 | 0.987 | CAT | CAC | CAT, CAC | H |
| | 370 | -7.69 | 0.012 | 0.0 | 0.001 | -20.344 | 0.996 | GTT | GTC, GTT | GTC, GTT | V |
| | 413 | -7.74 | 0.033 | 0.0 | 0.002 | -9.541 | 0.987 | GAG | GAA | GAA, GAG | E |
| | 414 | -577.37 | 0.037 | 0.0 | 0.0 | -19.819 | 0.997 | CGT | CGT, CGC | CGT, CGC | R |
| | 431 | -577.37 | 0.037 | 0.0 | 0.001 | -26.006 | 0.996 | GTA | GTG, GTA | GTG, GTA | V |
| | 444 | -1.40 | 0.0010 | 0.0 | 0.0 | -43.962 | 1.0 | TAC | TAT, TAC | TAT, TAC | Y |
| | 471 | -7.69 | 0.012 | 0.0 | 0.001 | -18.804 | 0.995 | GTT | GTC, GTT | GTC, GTT | V |
| | 669 | -9.03 | 0.0096 | 0.0 | 0.001 | -11.086 | 0.993 | AAT, AAC | AAT, AAC | AAC | N |
| | 733 | -8.78 | 0.0072 | 0.0 | 0.001 | -22.138 | 0.998 | ATT, ATA | ATC | ATC, ATT | I |
| | 834 | -7.70 | 0.049 | 0.029 | 0.001 | -38.791 | 0.995 | ACT \| | TTT, TTC | TTC, TTT | T \| F |
| | 864 | -577.37 | 0.037 | 0.0 | 0.001 | -14.845 | 0.993 | GGC | GGC | GGC, GGA, GGT | G |
| | 949 | -9.62 | 0.0041 | 0.0 | 0.0 | -40.53 | 1.0 | GCA | GCT, GCA | GCT | A |
| | 1323 | -602.36 | 0.045 | 0.0 | 0.007 | -4.403 | 0.981 | GAT | GAC | GAT, GAC | D |
| | 1402 | -6.64 | 0.044 | 0.0 | 0.0 | -20.142 | 0.994 | TGT | TGT | TGC, TGT | C |
| | 1429 | -120.76 | 0.0020 | 0.0 | 0.0 | -27.903 | 0.999 | CAT | CAC | CAC, CAT | H |
| | 1568 | -1.54 | 0.0031 | 0.005 | 0.0 | -46.063 | 1.0 | CAG \| | GAG, GAA | GAG | Q \| E |
| | 1669 | -6.12 | 0.043 | 0.0 | 0.003 | -5.79 | 0.988 | CAC | CAC, CAT | CAT | H |
| | 1800 | -1.20 | 0.0020 | 0.0 | 0.0 | -27.036 | 0.998 | GAT | GAT, GAC | GAC | D |
| | 1827 | -6.02 | 0.045 | 0.0 | 0.007 | -4.409 | 0.981 | GAT | GAT, GAC | GAC | D |
| | 1915 | -7.69 | 0.012 | 0.0 | 0.001 | -19.261 | 0.996 | GGT | GGT, GGC | GGT, GGC | G |
| | 1944 | -976.23 | 0.0076 | 0.0 | 0.0 | -26.7 | 0.998 | AAT | AAT, AAC | AAT, AAC | N |
| | 2018 | -9.03 | 0.0096 | 0.0 | 0.001 | -10.989 | 0.993 | TTT | TTC | TTC, TTT | F |
| | 2035 | -6.14 | 0.043 | 0.0 | 0.003 | -7.01 | 0.983 | GAT | GAT, GAC | GAT, GAC | D |
| | 2083 | -1.90 | 0.00024 | 0.0 | 0.0 | -45.551 | 1.0 | AAA | AAA, AAG | AAA, AAG | K |
| | 2258 | -9.62 | 0.0050 | 0.0 | 0.0 | -36.355 | 1.0 | TCG | TCA, TCC | TCA, TCC | S |
| | 2275 | -1.20 | 0.0028 | 0.0 | 0.0 | -26.385 | 0.998 | TGT | TGC, TGT | TGC | C |
| | 2280 | -6.12 | 0.043 | 0.0 | 0.004 | -5.838 | 0.982 | GAC | GAT | GAT, GAC | D |
| | 2284 | -1.09 | 0.0067 | 0.012 | 0.0 | -34.856 | 0.998 | CGT \| | CAC, CAT | CAC | R \| H |
| | 2299 | -1.34 | 0.00045 | 0.0 | 0.0 | -48.115 | 1.0 | GTT | GTG, GTT | GTG, GTT | V |
| | 2358 | -181.04 | 0.0000092 | 0.0 | 0.0 | -44.109 | 1.0 | TTT | TTT, TTC | TTC, TTT | F |
| | 2644 | -6.02 | 0.045 | 0.0 | 0.007 | -4.405 | 0.981 | GAT | GAC, GAT | GAC | D |
| | 2972 | -9.17 | 0.0092 | 0.0 | 0.0 | -18.368 | 0.996 | AGC | AGT, AGC | AGT, AGC | S |
| | 2992 | -9.97 | 0.011 | 0.0 | 0.0 | -37.526 | 0.999 | TAT | TAT, TAC | TAT | Y |
| | 3021 | -9.62 | 0.0041 | 0.0 | 0.0 | -40.176 | 1.0 | GGC, GGA | GGC, GGT | GGC, GGT | G |
| | 3036 | -6.02 | 0.0453 | 0.0 | 0.007 | -4.367 | 0.984 | TTT | TTC | TTT, TTC | F |
| | 3056 | -1.25 | 0.0025 | 0.015 | 0.0 | -45.329 | 1.0 | GGC \| | GCG, GCA | GCG, GCA | G \| A |
| | 3171 | -6.61 | 0.037 | 0.0 | 0.0 | -17.431 | 0.992 | GAT | GAC, GAT | GAT, GAC | D |
| | 3243 | -6.02 | 0.045 | 0.0 | 0.005 | -4.374 | 0.986 | AGT | AGC | AGC, AGT | S |
| | 3376 | -577.37 | 0.037 | 0.0 | 0.001 | -14.85 | 0.993 | GGC | GGC, GGT | GGC | G |
| | 3476 | -903.92 | 0.014 | 0.0 | 0.001 | -11.298 | 0.99 | TAT | TAC, TAT | TAC, TAT | Y |
| | 3618 | -7.34 | 0.015 | 0.0 | 0.0 | -29.756 | 0.999 | CTA | CTC, CTT | CTC | L |
| | 3625 | -819.00 | 0.036 | 0.03 | 0.017 | -13.807 | 0.955 | TGT \| | TTC, TTT | TTC, TTT | C \| F |
| | 3674 | -7.69 | 0.012 | 0.0 | 0.0 | -18.538 | 0.998 | CCT | CCC | CCT, CCC | P |
| | 3716 | -9.03 | 0.012 | 0.0 | 0.0 | -11.006 | 0.992 | TGT | TGC, TGT | TGC, TGT | C |
| | 3795 | -9.04 | 0.012 | 0.0 | 0.0 | -11.313 | 0.992 | TGT | TGC | TGC, TGT | C |
| | 3875 | -9.80 | 0.012 | 0.0 | 0.0 | -40.415 | 1.0 | TTA | CTC, CTA | CTC, CTA | L |
| | 3900 | -6.12 | 0.043 | 0.0 | 0.004 | -5.936 | 0.982 | GAC | GAT | GAT, GAC | D |
| | 3907 | -115.47 | 0.0013 | 0.0 | 0.0 | -40.101 | 1.0 | CCT | CCT, CCC | CCC | P |
| | 3956 | -1.07 | 0.0072 | 0.024 | 0.003 | -32.256 | 0.995 | AGT \| | ACC, ACG, A | ACC, ACG | S \| T |
| | 3959 | -7.50 | 0.035 | 0.0 | 0.003 | -8.803 | 0.986 | GAA | GAG, GAA | GAG | E |
| | 4024 | -8.39 | 0.012 | 0.0 | 0.0 | -24.954 | 0.997 | GAC, GAT | GAC, GAT | GAC, GAT | D |
| | 4045 | -6.02 | 0.045 | 0.0 | 0.007 | -4.417 | 0.981 | GAT | GAC | GAC, GAT | D |
| | 4068 | -5.59 | 0.044 | 0.0 | 0.002 | -19.444 | 0.997 | CTT | CTG, TTG | CTG | L |
| | 4094 | -1.50 | 0.00043 | 0.0 | 0.0 | -38.925 | 1.0 | AAT | AAT, AAC | AAT, AAC | N |
| | 4107 | -6.12 | 0.043 | 0.0 | 0.004 | -5.901 | 0.985 | AAC | AAT | AAT, AAC | N |
| | 4245 | -7.69 | 0.012 | 0.0 | 0.0 | -32.988 | 0.999 | GGT | GGG | GGG, GGT | G |
| | 4284 | -6.02 | 0.045 | 0.0 | 0.007 | -4.358 | 0.984 | TTT | TTC | TTC, TTT | F |

<table>
<tr><td>ORF1b</td><td>69</td><td>-1.37</td><td>0.016</td><td>0.0</td><td>0.002</td><td>-13.555</td><td>0.999</td><td>ATA</td><td>ATC, ATT</td><td>ATC, ATT</td><td>I</td></tr>
<tr><td></td><td>175</td><td>-1.17</td><td>0.040</td><td>0.0</td><td>0.002</td><td>-24.765</td><td>1.0</td><td>TTA</td><td>CTG, TTG</td><td>CTG, TTG</td><td>L</td></tr>
<tr><td></td><td>359</td><td>-3.17</td><td>0.0000050</td><td>0.0</td><td>0.0</td><td>-49.12</td><td>1.0</td><td>GTG</td><td>GTG, GTT</td><td>GTG, GTT</td><td>V</td></tr>
<tr><td></td><td>533</td><td>-2.81</td><td>0.00015</td><td>0.0</td><td>0.0</td><td>-48.006</td><td>1.0</td><td>GTC</td><td>GTT, GTC</td><td>GTT</td><td>V</td></tr>
<tr><td></td><td>557</td><td>-241.81</td><td>0.00084</td><td>0.0</td><td>0.0</td><td>-37.041</td><td>1.0</td><td>TTT</td><td>TTT, TTC</td><td>TTC</td><td>F</td></tr>
<tr><td></td><td>651</td><td>-1.11</td><td>0.044</td><td>0.0</td><td>0.0</td><td>-36.513</td><td>1.0</td><td>TTG</td><td>TTG, TTA</td><td>TTG, TTA</td><td>L</td></tr>
<tr><td></td><td>684</td><td>-114.86</td><td>0.041</td><td>0.0</td><td>0.0</td><td>-19.561</td><td>0.999</td><td>GAT</td><td>GAT</td><td>GAT, GAC</td><td>D</td></tr>
<tr><td></td><td>736</td><td>-1.44</td><td>0.014</td><td>0.0</td><td>0.001</td><td>-12.595</td><td>0.999</td><td>TTT</td><td>TTT, TTC</td><td>TTT, TTC</td><td>F</td></tr>
<tr><td></td><td>845</td><td>-2.75</td><td>0.00083</td><td>0.0</td><td>0.0</td><td>-44.319</td><td>1.0</td><td>TAT</td><td>TAC, TAT</td><td>TAC, TAT</td><td>Y</td></tr>
<tr><td></td><td>1017</td><td>-176.13</td><td>0.0041</td><td>0.0</td><td>0.0</td><td>-30.298</td><td>1.0</td><td>CGT</td><td>CGC, CGT</td><td>CGC</td><td>R</td></tr>
<tr><td></td><td>1065</td><td>-1.40</td><td>0.012</td><td>0.0</td><td>0.001</td><td>-31.087</td><td>1.0</td><td>GTC</td><td>GTA, GTG</td><td>GTA, GTG, GTT</td><td>V</td></tr>
<tr><td></td><td>1146</td><td>-1.72</td><td>0.0085</td><td>0.0</td><td>0.0</td><td>-34.714</td><td>1.0</td><td>CAT</td><td>CAT, CAC</td><td>CAT, CAC</td><td>H</td></tr>
<tr><td></td><td>1150</td><td>-1.40</td><td>0.012</td><td>0.0</td><td>0.0</td><td>-41.844</td><td>1.0</td><td>GGG, GGT</td><td>GGG</td><td>GGG, GGT</td><td>G</td></tr>
<tr><td></td><td>1152</td><td>-1.05</td><td>0.037</td><td>0.0</td><td>0.003</td><td>-16.826</td><td>0.999</td><td>GCA</td><td>GCG</td><td>GCG, GCT</td><td>A</td></tr>
<tr><td></td><td>1154</td><td>-1.44</td><td>0.019</td><td>0.0</td><td>0.002</td><td>-12.643</td><td>0.999</td><td>TAT</td><td>TAC</td><td>TAC, TAT</td><td>Y</td></tr>
<tr><td></td><td>1308</td><td>-1.05</td><td>0.037</td><td>0.0</td><td>0.003</td><td>-18.997</td><td>0.999</td><td>GTA, GTT</td><td>GTG</td><td>GTG, GTT</td><td>V</td></tr>
<tr><td></td><td>1383</td><td>-1.40</td><td>0.012</td><td>0.0</td><td>0.0</td><td>-37.855</td><td>1.0</td><td>GGT</td><td>GGT, GGC</td><td>GGT, GGC</td><td>G</td></tr>
<tr><td></td><td>1399</td><td>-4.93</td><td>0.00015</td><td>0.0</td><td>0.0</td><td>-49.086</td><td>1.0</td><td>TAT</td><td>TAC, TAT</td><td>TAC, TAT</td><td>Y</td></tr>
<tr><td></td><td>1451</td><td>-1.45</td><td>0.019</td><td>0.0</td><td>0.002</td><td>-12.932</td><td>0.999</td><td>TAT</td><td>TAC, TAT</td><td>TAC</td><td>Y</td></tr>
<tr><td></td><td>1453</td><td>-176.13</td><td>0.0041</td><td>0.0</td><td>0.0</td><td>-32.453</td><td>1.0</td><td>CTT</td><td>CTT, CTC</td><td>CTT, CTC</td><td>L</td></tr>
<tr><td></td><td>1612</td><td>-2.46</td><td>0.00045</td><td>0.0</td><td>0.0</td><td>-47.727</td><td>1.0</td><td>GCT</td><td>GCA, GCG</td><td>GCA, GCG</td><td>A</td></tr>
<tr><td></td><td>1708</td><td>-1.05</td><td>0.037</td><td>0.0</td><td>0.0</td><td>-27.274</td><td>1.0</td><td>GCT</td><td>GCA, GCC, G[illegible]</td><td>GCA, GCC, GCT</td><td>A</td></tr>
<tr><td></td><td>1812</td><td>-2.79</td><td>0.0015</td><td>0.008</td><td>0.0</td><td>-47.231</td><td>1.0</td><td>AAA, AAG</td><td>AGA, AGG</td><td>AGA, AGG</td><td>K | R</td></tr>
<tr><td></td><td>2051</td><td>-144.73</td><td>0.014</td><td>0.0</td><td>0.001</td><td>-12.432</td><td>0.999</td><td>CAT</td><td>CAC, CAT</td><td>CAC</td><td>H</td></tr>
<tr><td></td><td>2120</td><td>-1.49</td><td>0.0097</td><td>0.0</td><td>0.0</td><td>-36.379</td><td>1.0</td><td>ATC</td><td>ATC, ATT</td><td>ATC, ATT</td><td>I</td></tr>
<tr><td></td><td>2151</td><td>-1.45</td><td>0.014</td><td>0.0</td><td>0.001</td><td>-12.618</td><td>0.999</td><td>TTT</td><td>TTC, TTT</td><td>TTC, TTT</td><td>F</td></tr>
<tr><td></td><td>2351</td><td>-2.41</td><td>0.0013</td><td>0.0</td><td>0.0</td><td>-36.759</td><td>1.0</td><td>TAT</td><td>TAC, TAT</td><td>TAC, TAT</td><td>Y</td></tr>
<tr><td></td><td>2381</td><td>-1.05</td><td>0.037</td><td>0.0</td><td>0.003</td><td>-10.207</td><td>0.999</td><td>GCT</td><td>GCC</td><td>GCC, GCT</td><td>A</td></tr>
<tr><td></td><td>2403</td><td>-2.11</td><td>0.0013</td><td>0.0</td><td>0.0</td><td>-45.099</td><td>1.0</td><td>ACT</td><td>ACA, ACC</td><td>ACA, ACC</td><td>T</td></tr>
<tr><td>S</td><td>90</td><td>-1.22</td><td>0.0015</td><td>0.01</td><td>0.0</td><td>-46.435</td><td>1.0</td><td>TTA |</td><td>GTT, GTC</td><td>GTT, GTC</td><td>L | V</td></tr>
<tr><td></td><td>273</td><td>-4.39</td><td>0.044</td><td>0.0</td><td>0.002</td><td>-12.525</td><td>0.996</td><td>TCT</td><td>TCA, TCT</td><td>TCA, TCT</td><td>S</td></tr>
<tr><td></td><td>299</td><td>-2.28</td><td>0.000000071</td><td>0.0</td><td>0.0</td><td>-48.955</td><td>1.0</td><td>TAC</td><td>TAT, TAC</td><td>TAT, TAC</td><td>Y</td></tr>
<tr><td></td><td>319</td><td>-4.52</td><td>0.046</td><td>0.0</td><td>0.007</td><td>-3.837</td><td>0.983</td><td>TTT</td><td>TTC</td><td>TTC, TTT</td><td>F</td></tr>
<tr><td></td><td>391</td><td>-4.60</td><td>0.044</td><td>0.0</td><td>0.007</td><td>-5.692</td><td>0.978</td><td>TTT, TTC</td><td>TTT</td><td>TTT</td><td>F</td></tr>
<tr><td></td><td>451</td><td>-1.29</td><td>0.00026</td><td>0.0</td><td>0.0</td><td>-46.889</td><td>1.0</td><td>TAC</td><td>TAC, TAT</td><td>TAC, TAT</td><td>Y</td></tr>
<tr><td></td><td>476</td><td>-6.87</td><td>0.026</td><td>0.0</td><td>0.001</td><td>-12.409</td><td>0.992</td><td>AAG</td><td>AAA</td><td>AAA, AAG</td><td>K</td></tr>
<tr><td></td><td>483</td><td>-4.39</td><td>0.037</td><td>0.0</td><td>0.001</td><td>-13.115</td><td>0.997</td><td>ACT</td><td>ACT, ACA</td><td>ACT, ACA</td><td>T</td></tr>
<tr><td></td><td>689</td><td>-7.32</td><td>0.019</td><td>0.07</td><td>0.002</td><td>-40.426</td><td>0.999</td><td>ACT |</td><td>TCT, TCC</td><td>TCT, TCC | TTC</td><td>T | S | F</td></tr>
<tr><td></td><td>704</td><td>-8.20</td><td>0.036</td><td>0.003</td><td>0.0</td><td>-44.386</td><td>1.0</td><td>CGC |</td><td>AAA, AAG</td><td>AAA, AAG</td><td>R | K</td></tr>
<tr><td></td><td>711</td><td>-8.78</td><td>0.0017</td><td>0.0</td><td>0.0</td><td>-40.116</td><td>1.0</td><td>GGA</td><td>GGT, GGC</td><td>GGT, GGC</td><td>G</td></tr>
<tr><td></td><td>751</td><td>-450.26</td><td>0.043</td><td>0.0</td><td>0.0065</td><td>-28.693</td><td>0.959</td><td></td><td>CTC, CTT</td><td>CTC, CTT | TTC</td><td>L | F</td></tr>
<tr><td></td><td>773</td><td>-5.85</td><td>0.012</td><td>0.0</td><td>0.0</td><td>-21.35</td><td>0.999</td><td>CCA</td><td>CCT, CCC</td><td>CCT, CCC</td><td>P</td></tr>
<tr><td></td><td>777</td><td>-4.60</td><td>0.044</td><td>0.0</td><td>0.007</td><td>-5.811</td><td>0.974</td><td>GAC, GAT</td><td>GAT</td><td>GAT</td><td>D</td></tr>
<tr><td></td><td>794</td><td>-4.60</td><td>0.044</td><td>0.0</td><td>0.007</td><td>-5.692</td><td>0.978</td><td>TTT, TTC</td><td>TTT</td><td>TTT</td><td>F</td></tr>
<tr><td></td><td>817</td><td>-6.37</td><td>0.014</td><td>0.0</td><td>0.0</td><td>-16.533</td><td>0.998</td><td>TGC, TGT</td><td>TGC, TGT</td><td>TGC, TGT</td><td>C</td></tr>
<tr><td></td><td>820</td><td>-4.52</td><td>0.046</td><td>0.0</td><td>0.007</td><td>-3.835</td><td>0.983</td><td>TTT</td><td>TTC</td><td>TTT, TTC</td><td>F</td></tr>
<tr><td></td><td>909</td><td>-4.39</td><td>0.037</td><td>0.0</td><td>0.0</td><td>-12.863</td><td>0.997</td><td>CCC, CCA</td><td>CCT</td><td>CCC, CCT</td><td>P</td></tr>
<tr><td></td><td>953</td><td>-182.09</td><td>0.000012</td><td>0.0</td><td>0.0</td><td>-47.405</td><td>1.0</td><td>TAC</td><td>TAC, TAT</td><td>TAC, TAT</td><td>Y</td></tr>
<tr><td></td><td>954</td><td>-5.85</td><td>0.012</td><td>0.0</td><td>0.0</td><td>-26.197</td><td>1.0</td><td>ACA, ACG</td><td>ACC, ACT</td><td>ACC, ACT</td><td>T</td></tr>
<tr><td></td><td>982</td><td>-1.32</td><td>0.0000056</td><td>0.0</td><td>0.0</td><td>-46.869</td><td>1.0</td><td>ATC</td><td>ATC, ATT</td><td>ATC, ATT</td><td>I</td></tr>
<tr><td></td><td>1027</td><td>-6.66</td><td>0.028</td><td>0.0</td><td>0.002</td><td>-10.799</td><td>0.991</td><td>AAA</td><td>AAG</td><td>AAA, AAG</td><td>K</td></tr>
<tr><td></td><td>1037</td><td>-6.66</td><td>0.036</td><td>0.0</td><td>0.002</td><td>-10.903</td><td>0.991</td><td>CAA</td><td>CAG</td><td>CAG, CAA</td><td>Q</td></tr>
<tr><td></td><td>1046</td><td>-6.31</td><td>0.013</td><td>0.0</td><td>0.001</td><td>-23.184</td><td>1.0</td><td>TTG</td><td>CTA</td><td>CTA, TTA</td><td>L</td></tr>
<tr><td></td><td>1084</td><td>-5.25</td><td>0.027</td><td>0.0</td><td>0.001</td><td>-13.686</td><td>0.998</td><td>TTG</td><td>CTA, TTA</td><td>CTA, TTA</td><td>L</td></tr>
<tr><td></td><td>1087</td><td>-4.90</td><td>0.039</td><td>0.0</td><td>0.0</td><td>-17.954</td><td>0.996</td><td>TTT</td><td>TTT</td><td>TTT, TTC</td><td>F</td></tr>
<tr><td></td><td>1142</td><td>-1.50</td><td>0.000031</td><td>0.0</td><td>0.0</td><td>-47.643</td><td>1.0</td><td>TTC</td><td>TTT, TTC</td><td>TTT, TTC</td><td>F</td></tr>
<tr><td></td><td>1206</td><td>-7.33</td><td>0.040</td><td>0.03</td><td>0.001</td><td>-37.655</td><td>0.999</td><td>ATT</td><td>ATC</td><td>ATC, ATT</td><td>I</td></tr>
<tr><td></td><td>1237</td><td>-4.53</td><td>0.046</td><td>0.0</td><td>0.008</td><td>-3.885</td><td>0.979</td><td>GAT</td><td>GAC</td><td>GAC, GAT</td><td>D</td></tr>
<tr><td></td><td>1287</td><td>-6.79</td><td>0.014</td><td>0.0</td><td>0.002</td><td>-8.564</td><td>0.994</td><td>TAT</td><td>TAC, TAT</td><td>TAC</td><td>Y</td></tr>
<tr><td></td><td>1310</td><td>-6.79</td><td>0.0099</td><td>0.0</td><td>0.001</td><td>-8.522</td><td>0.996</td><td>TTT</td><td>TTC</td><td>TTC, TTT</td><td>F</td></tr>
<tr><td>ORF4b</td><td>132</td><td>-3.45</td><td>0.039</td><td>0.0</td><td>0.003</td><td>-12.222</td><td>0.97</td><td>TTT, TTC</td><td>TTT</td><td>TTT</td><td>F</td></tr>
<tr><td></td><td>231</td><td>-3.09</td><td>0.037</td><td>0.0</td><td>0.002</td><td>-14.667</td><td>0.993</td><td>GTT</td><td>GTC, GTT</td><td>GTC</td><td>V</td></tr>
<tr><td>ORF5</td><td>81</td><td>-4.58</td><td>0.046</td><td>0.0</td><td>0.003</td><td>-11.698</td><td>0.98</td><td>AAT</td><td>AAT, AAC</td><td>AAC</td><td>N</td></tr>
<tr><td></td><td>114</td><td>-4.64</td><td>0.045</td><td>0.0</td><td>0.002</td><td>-15.152</td><td>0.984</td><td>GAC</td><td>GAC, GAT</td><td>GAT</td><td>D</td></tr>
<tr><td>M</td><td>37</td><td>-584.02</td><td>0.042</td><td>0.0</td><td>0.001</td><td>-17.388</td><td>0.998</td><td>GGT, GGC</td><td>GGA, GGT</td><td>GGA</td><td>G</td></tr>
<tr><td></td><td>155</td><td>-6.23</td><td>0.043</td><td>0.0</td><td>0.003</td><td>-9.959</td><td>0.986</td><td>TTT</td><td>TTC, TTT</td><td>TTC</td><td>F</td></tr>
<tr><td>N</td><td>46</td><td>-8.32</td><td>0.037</td><td>0.0</td><td>0.002</td><td>-20.441</td><td>0.999</td><td>GGC, GGT</td><td>GGG</td><td>GGG, GGT</td><td>G</td></tr>
<tr><td></td><td>50</td><td>-845.44</td><td>0.047</td><td>0.0</td><td>0.001</td><td>-15.929</td><td>0.991</td><td>CAT, CAC</td><td>CAC, CAT</td><td>CAC</td><td>H</td></tr>
<tr><td></td><td>100</td><td>-8.93</td><td>0.042</td><td>0.0</td><td>0.004</td><td>-9.117</td><td>0.986</td><td>TTT</td><td>TTC</td><td>TTT, TTC</td><td>F</td></tr>
<tr><td></td><td>155</td><td>-162.22</td><td>0.0071</td><td>0.0</td><td>0.0</td><td>-29.584</td><td>1.0</td><td>AAA</td><td>AAA, AAG</td><td>AAA, AAG</td><td>K</td></tr>
<tr><td></td><td>195</td><td>-8.32</td><td>0.037</td><td>0.0</td><td>0.002</td><td>-15.334</td><td>0.997</td><td>AAT</td><td>AAC</td><td>AAC</td><td>N</td></tr>
<tr><td></td><td>263</td><td>-8.94</td><td>0.042</td><td>0.0</td><td>0.004</td><td>-9.238</td><td>0.986</td><td>TTT</td><td>TTT, TTC</td><td>TTC</td><td>F</td></tr>
<tr><td></td><td>397</td><td>-9.71</td><td>0.045</td><td>0.054</td><td>0.018</td><td>-29.618</td><td>0.99</td><td>GCG |</td><td>CCA, CCT</td><td>CCA, CCT</td><td>A | P</td></tr>
</table>

Sites under natural selection within the SARS-CoV-2 CDSs

| CDS | Test | Negative/Purifyng | | | Positive/Diversifying | | |
|---|---|---|---|---|---|---|---|
| | | Sites | Model | Ratio dN/dS | Sites | Model | Ratio dN/dS |
| ORF1a | SLAC | 35 | GTR | 0.136 | 1 | GTR | 0.136 |
| | FEL | 451 | GTR | 0.118 | 1 | GTR | 0.118 |
| | MEME | | | | 47 | GTR | 0.118 |
| | FUBAR | 1182 | GTR | | 1 | GTR | |
| ORF1b | SLAC | 15 | GTR | 0.0595 | 1 | GTR | 0.0595 |
| | FEL | 240 | GTR | 0.0464 | 1 | GTR | 0.0464 |
| | MEME | | | | 13 | GTR | 0.0464 |
| | FUBAR | 719 | GTR | | 2 | GTR | |
| S | SLAC | 20 | GTR | 0.173 | 1 | GTR | 0.173 |
| | FEL | 270 | GTR | 0.142 | 2 | GTR | 0.142 |
| | MEME | | | | 23 | GTR | 0.142 |
| | FUBAR | 431 | GTR | | 1 | GTR | |
| ORF3a | SLAC | 2 | GTR | 0.371 | | | |
| | FEL | 21 | GTR | 0.330 | | | |
| | MEME | | | | 3 | GTR | 0.330 |
| | FUBAR | 16 | GTR | | 1 | GTR | |
| E | SLAC | 0 | GTR | 0.300 | | | |
| | FEL | 1 | GTR | 0.288 | | | |
| | MEME | | | | 0 | GTR | 0.288 |
| | FUBAR | 1 | GTR | | | | |
| M | SLAC | 4 | GTR | 0.0527 | | | |
| | FEL | 24 | GTR | 0.0408 | | | |
| | MEME | | | | 0 | GTR | 0.0408 |
| | FUBAR | 18 | GTR | | | | |
| ORF6 | SLAC | 0 | GTR | 0.349 | | | |
| | FEL | 0 | GTR | 0.331 | | | |
| | MEME | | | | 0 | GTR | 0.331 |
| | FUBAR | 0 | GTR | | | | |
| ORF7a | SLAC | 0 | GTR | 0.194 | | | |
| | FEL | 7 | GTR | 0.182 | | | |
| | MEME | | | | 0 | GTR | 0.182 |
| | FUBAR | 5 | GTR | | | | |
| ORF7b | SLAC | 0 | GTR | 0.355 | | | |
| | FEL | 1 | GTR | 0.312 | | | |
| | MEME | | | | 0 | GTR | 0.312 |
| | FUBAR | 1 | GTR | | | | |
| ORF8 | SLAC | | | | | | |
| | FEL | | | | | | |
| | MEME | | | | | | |
| | FUBAR | | | | | | |
| N | SLAC | 10 | GTR | 0.371 | 1 | GTR | 0.371 |
| | FEL | 25 | GTR | 0.175 | 1 | GTR | 0.328 |
| | MEME | | | | 5 | GTR | 0.328 |
| | FUBAR | 26 | GTR | | 2 | GTR | |
| ORF9b | SLAC | 0 | GTR | 1.33 | | | |
| | FEL | 2 | GTR | 1.30 | | | |
| | MEME | | | | 0 | GTR | 1.30 |
| | FUBAR | | | | 2 | GTR | |
| ORF9c | | | | | | | |
| ORF10 | SLAC | 0 | GTR | 1.35 | 1 | GTR | 1.35 |
| | FEL | 0 | GTR | 1.30 | 1 | GTR | 1.40 |
| | MEME | | | | 1 | GTR | 1.40 |
| | FUBAR | | | | 1 | GTR | |

Positive
No sites
No report

| Negative/Purifyng | | | | | | | | | | | |
|---|---|---|---|---|---|---|---|---|---|---|---|
| Gene | Gene codon position | SLAC | | FEL | | FUBAR | | Inferred substitution | | | Amino acid |
| | | dN-dS | P[dN/dS < 1] | dN/dS | p-value | dN-dS | Prob[dS>dN] | Bat | Intermediate | Human | |
| ORF1a | 142 | -1.21 | 0.038 | 0.0 | 0.003 | -9.684 | 0.999 | TCC, TCG | TCC, TCA | TCA | S |
| | 180 | -1.21 | 0.041 | 0.0 | 0.002 | -12.906 | 0.999 | GGA | GGG, GGA | GGA | G |
| | 206 | -1.21 | 0.037 | 0.0 | 0.002 | -11.142 | 0.999 | GCT, GCC | GCG, GCA | GCA | A |
| | 225 | -1.60 | 0.021 | 0.0 | 0.002 | -13.368 | 0.999 | AAA | AAG | AAA, AAG | K |
| | 491 | -1.21 | 0.037 | 0.0 | 0.004 | -10.04 | 0.999 | GTG, GTA | GTC | GTG, GTT | V |
| | 599 | -1.21 | 0.037 | 0.0 | 0.002 | -7.354 | 0.999 | GCA, GCT | GCA, GCT, GCC | GCC, GCT | A |
| | 802 | -1.13 | 0.045 | 0.0 | 0.001 | -11.704 | 0.999 | CTT, CTA | CTT | CTT, CTA, CTC | L |
| | 875 | -1.21 | 0.037 | 0.0 | 0.005 | -8.527 | 0.999 | GTA, GTT | GTC, GTG | GTG | V |
| | 924 | -1.43 | 0.022 | 0.0 | 0.0 | -13.835 | 0.999 | TTT, TTC | TTC, TTT | TTC. TTT | F |
| | 1253 | -1.39 | 0.024 | 0.0 | 0.01 | -5.655 | 0.997 | AAC, AAT | AAT, AAC | AAT, AAC | N |
| | 1331 | -1.21 | 0.037 | 0.0 | 0.001 | -14.446 | 1.0 | CCT, CCA | CCT, CCG, | CCG | P |
| | 1427 | -1.62 | 0.012 | 0.0 | 0.001 | -9.076 | 1.0 | ACC, ACT | ACC, ACT | ACC, ACT | T |
| | 1536 | -1.21 | 0.037 | 0.0 | 0.001 | -17.719 | 0.999 | GTG, GTT | GTT, GTA | GTG, GTA | V |
| | 1762 | -1.21 | 0.037 | 0.0 | 0.002 | -7.44 | 0.999 | ACT, ACA | ACT, ACC | ACT, ACC | T |
| | 1908 | -1.35 | 0.026 | 0.0 | 0.006 | -5.934 | 0.998 | TTT, TTC | TTT, TTC | TTT, TTC | F |
| | 1936 | -1.25 | 0.033 | 0.0 | 0.016 | -4.87 | 0.997 | ATC | ATT, ATC | ATT, ATC | I |
| | 2008 | -1.21 | 0.037 | 0.0 | 0.012 | -5.106 | 0.998 | ACC, ACT | ACC, ACT | ACC, ACT | T |
| | 2335 | -1.54 | 0.018 | 0.0 | 0.001 | -9.018 | 0.999 | TTT | TTT, TTC | TTT, TTC | F |
| | 2339 | -138.06 | 0.025 | 0.0 | 0.011 | -5.214 | 0.998 | TTC | TTT, TTC | TTT, TTC | F |
| | 2386 | -1.47 | 0.020 | 0.0 | 0.003 | -7.416 | 0.999 | ATT | ATC, ATA | ATC, ATT | I |
| | 2422 | -1.21 | 0.037 | 0.0 | 0.003 | -7.375 | 0.999 | GTT | GTC, GTT | GTC, GTT | V |
| | 2501 | -2.02 | 0.0041 | 0.0 | 0.001 | -10.173 | 1.0 | TCC | TCC, TCT | TCC, TCT | S |
| | 2527 | -1.39 | 0.024 | 0.0 | 0.01 | -5.657 | 0.997 | AAT, AAC | AAT, AAC | AAC, AAT | N |
| | 2626 | -1.21 | 0.037 | 0.0 | 0.018 | -4.765 | 0.997 | TCC | TCC, TCT | TCC | S |
| | 2709 | -1.39 | 0.024 | 0.0 | 0.01 | -5.66 | 0.997 | AAC, AAT | AAC, AAT | AAC, AAT | N |
| | 2840 | -1.54 | 0.018 | 0.0 | 0.001 | -9.324 | 0.999 | AGT | AGC, AGT | AGC, AGT | S |
| | 3019 | -1.39 | 0.024 | 0.0 | 0.007 | -5.605 | 0.998 | TTT, TTC | TTT, TTC | TTT, TTC | F |
| | 3048 | -162.86 | 0.027 | 0.0 | 0.002 | -9.3 | 0.997 | ATA, ATT | ATA, ATT | ATA | I |
| | 3322 | -1.21 | 0.037 | 0.0 | 0.009 | -5.313 | 0.998 | CTT | CTC | CTC, CTT | L |
| | 3339 | -1.62 | 0.012 | 0.0 | 0.003 | -6.97 | 0.999 | CTC, CTT | CTC, CTT | CTC, CTT | L |
| | 3378 | -1.21 | 0.037 | 0.0 | 0.009 | -7.402 | 0.999 | GTG | GTA, GTG | GTT, GTA | V |
| | 3731 | -1.21 | 0.037 | 0.0 | 0.013 | -5.079 | 0.998 | GCC, GCT | GCC, GCT | GCC, GCT | A |
| | 3832 | -1.21 | 0.037 | 0.0 | 0.008 | -5.6 | 0.998 | CCC, CCA | CCT, CCC | CCC | P |
| | 3854 | -1.75 | 0.0091 | 0.0 | 0.0 | -12.623 | 1.0 | ATT | ATC, ATT | ATC, ATT | I |
| | 4059 | -1.21 | 0.037 | 0.0 | 0.003 | -7.016 | 0.999 | CCA | CCG, CCC | CCT, CCC | P |
| ORF1b | 346 | -1.58 | 0.031 | 0.0 | 0.008 | -8.571 | 0.999 | TAC | TAC, TAT | TAC, TAT | Y |
| | 504 | -1.68 | 0.024 | 0.0 | 0.006 | -8.104 | 0.999 | CAT, CAC | CAT, CAC | CAT, CAC | H |
| | 657 | -2.44 | 0.0051 | 0.0 | 0.0 | -16.385 | 1.0 | TTT, TTC | TTT, TTC | TTT, TTC | F |
| | 887 | -1.58 | 0.031 | 0.0 | 0.009 | -8.558 | 0.999 | GTC | GTC | GTC, GTT | V |
| | 1261 | -1.46 | 0.037 | 0.0 | 0.011 | -7.263 | 0.999 | CTT, CTC | CTT, CTC | CTT, CTC | L |
| | 1318 | -1.46 | 0.037 | 0.0 | 0.01 | -11.116 | 1.0 | GTG | GTG, GTA | GTA, GTT | V |
| | 1326 | -1.68 | 0.024 | 0.0 | 0.008 | -8.112 | 0.999 | AAT, AAC | AAT, AAC | AAC, AAT | N |
| | 1468 | -1.46 | 0.037 | 0.0 | 0.009 | -7.398 | 0.999 | CCT | CCA, CCT | CCT | P |
| | 1495 | -1.78 | 0.020 | 0.0 | 0.005 | -8.702 | 0.999 | AAT | AAC | AAC, AAT | N |
| | 1687 | -1.46 | 0.037 | 0.0 | 0.003 | -9.745 | 1.0 | GTT | GTA, GTC | GTC, GTT | V |
| | 1919 | -1.46 | 0.037 | 0.0 | 0.002 | -9.828 | 1.0 | CTT | CTT, CTC | CTC, CTT | L |
| | 1945 | -1.68 | 0.024 | 0.0 | 0.008 | -8.023 | 0.998 | AAT, AAC | AAT, AAC | AAT, AAC | N |
| | 2496 | -2.44 | 0.0041 | 0.0 | 0.0 | -39.287 | 1.0 | GCA | GCG, GCC | GCG, GCC, GCT | A |
| | 2555 | -1.46 | 0.037 | 0.0 | 0.001 | -15.703 | 1.0 | GCA | GCT | GCT, GCC | A |
| | 2583 | -178.42 | 0.020 | 0.0 | 0.005 | -8.706 | 0.999 | AAT | AAC | AAC, AAT | N |
| S | 64 | -6.43 | 0.039 | 0.0 | 0.001 | -6.971 | 0.998 | TTT | TTT, TTC | TTC | F |
| | 290 | -937.79 | 0.022 | 0.0 | 0.002 | -10.014 | 0.997 | GAG | GAA | GAA | E |
| | 356 | -6.31 | 0.041 | 0.0 | 0.005 | -5.473 | 0.994 | TTC | TTT | TTT | F |
| | 419 | -6.82 | 0.022 | 0.0 | 0.001 | -7.103 | 0.999 | ATA, ATT | ATC, ATT | ATC, ATT | I |
| | 482 | -848.26 | 0.016 | 0.0 | 0.001 | -11.981 | 0.999 | TAT, TAC | TAT, TAC | TAT, TAC | Y |
| | 484 | -5.75 | 0.037 | 0.0 | 0.001 | -7.827 | 0.999 | GCA | GCT, GCC | GCT, GCC | A |
| | 501 | -6.57 | 0.033 | 0.0 | 0.0 | -20.518 | 1.0 | TTG, CTT | CTA, TTA | TTA, CTA | L |
| | 552 | -6.43 | 0.039 | 0.0 | 0.001 | -6.984 | 0.998 | TTT | TTC, TTT | TTC, TTT | F |
| | 636 | -6.31 | 0.041 | 0.0 | 0.006 | -5.598 | 0.993 | GAC | GAT | GAT | D |
| | 732 | -9.59 | 0.0041 | 0.0 | 0.0 | -27.923 | 1.0 | ACC | ACC | ACC, ACT | T |
| | 773 | -6.43 | 0.039 | 0.0 | 0.001 | -7.076 | 0.997 | AAT | AAT, AAC | AAT, AAC | N |
| | 787 | -5.75 | 0.037 | 0.0 | 0.001 | -6.936 | 0.999 | ACT | ACA, ACC | ACC, ACT | T |
| | 799 | -754.66 | 0.039 | 0.0 | 0.003 | -9.71 | 0.997 | AAA, AAG | AAA, AAG | AAA | K |
| | 828 | -937.79 | 0.022 | 0.0 | 0.002 | -10.014 | 0.997 | GAG | GAA | GAA | E |
| | 833 | -8.28 | 0.012 | 0.0 | 0.001 | -6.783 | 0.998 | AAT | AAT, AAC | AAC | N |
| | 864 | -6.61 | 0.037 | 0.0 | 0.0 | -11.338 | 0.998 | TTT, TTC | TTT | TTT, TTC | F |
| | 865 | -8.17 | 0.012 | 0.0 | 0.001 | -7.113 | 0.998 | AAC, AAT | AAC, AAT | AAC, AAT | N |
| | 988 | -6.49 | 0.038 | 0.0 | 0.001 | -9.037 | 0.996 | GAT | GAT, GAC | GAT, GAC | D |
| | 1125 | -5.75 | 0.037 | 0.0 | 0.001 | -9.889 | 0.999 | ACA, ACT | ACC, ACT | ACC, ACT | T |
| | 1259 | -66.17 | 0.046 | 0.0 | 0.0 | -11.603 | 0.998 | TGC, TGT | TGT | TGC, TGT | C |
| ORF3a | 7 | -1.30 | 0.031 | 0.0 | 0.012 | -11.483 | 0.994 | ATC, ATT | ATC, ATT | ATC, ATT | I |
| | 43 | -1.33 | 0.028 | 0.0 | 0.006 | -13.878 | 0.995 | TTC | TTC | TTC, TTT | F |
| M | 93 | -3.18 | 0.0022 | 0.0 | 0.0 | -39.662 | 1.0 | CTG, CTC | CTT, CTC, CTG | CTT, CTC, CTG | L |
| | 120 | -1.79 | 0.034 | 0.0 | 0.003 | -27.115 | 0.999 | CTC, CTA | TTG, CTC, CTT | CTC | L |
| | 135 | -3.83 | 0.0049 | 0.0 | 0.0 | -38.729 | 1.0 | GAA, GAG | GAA, GAG | GAA, GAG | E |
| | 138 | -1.72 | 0.037 | 0.0 | 0.009 | -16.63 | 0.997 | CTC, CTT | CTC, CTT | CTC | L |
| N | 8 | -2.13 | 0.010 | 0.0 | 0.007 | -16.598 | 0.996 | AAC | AAT | AAC, AAT | N |
| | 9 | -1.99 | 0.045 | 0.0 | 0.01 | -21.552 | 0.992 | CAA | CAG | CAG | Q |
| | 35 | -1.41 | 0.037 | 0.0 | 0.009 | -19.338 | 0.998 | GCA | GCT, GCG | GCC, GCG | A |
| | 110 | -1.96 | 0.013 | 0.0 | 0.001 | -15.622 | 0.998 | TTT | TTT, TTC | TTT, TTC | F |
| | 192 | -1.84 | 0.016 | 0.0 | 0.017 | -12.376 | 0.993 | AAC, AAT | AAC | AAC, AAT | N |
| | 214 | -165.66 | 0.045 | 0.138 | 0.045 | -16.173 | 0.98 | GGC, GGT | GGC, GGT | GGC, GGT \| TGC | G \| C |
| | 234 | -1.77 | 0.0028 | 0.018 | 0.001 | -33.084 | 0.978 | ATG | ATG | ATG \| ATC, ATT, ATA | M \| I |
| | 289 | -1.99 | 0.045 | 0.0 | 0.01 | -22.028 | 0.992 | CAG, CAA | CAG, CAA | CAG | Q |
| | 348 | -1.43 | 0.048 | 0.0 | 0.022 | -10.381 | 0.975 | GAT, GAC | GAT | GAT, GAC | D |
| | 374 | -1.99 | 0.030 | 0.0 | 0.008 | -21.56 | 0.992 | AAA | AAA, AAG | AAG | K |

Material_S1_SARS-metadata

| strain | specie | virus | genbank_accession | date | region | country | region_exposure | country_exposure | source | segment | length | host |
|---|---|---|---|---|---|---|---|---|---|---|---|---|
| **B1-AS-CHA** | Aselliscus stoliczkanus | SARS-CoV | KY417142 | 2014-05-12 | Asia | China | Asia | China | Fecal | genome | 29725 | Bat |
| **B2-RS-CHA** | Rhinolophus sinicus | SARS-CoV | KY417143 | 2012-09-18 | Asia | China | Asia | China | Urine and fecal | genome | 29725 | Bat |
| **B3-RS-CHA** | Rhinolophus sinicus | SARS-CoV | KY417147 | 2013-04-17 | Asia | China | Asia | China | Urine and fecal | genome | 29725 | Bat |
| **B4-RS-CHA** | Rhinolophus sinicus | SARS-CoV | KY417149 | 2013-04-17 | Asia | China | Asia | China | Urine and fecal | genome | 29725 | Bat |
| **I1-PL-CHA** | Paguma larvata | SARS-CoV | AY545916 | 2013-07-05 | Asia | China | Asia | China | | genome | 29707 | Intermediate |
| **I2-PL-CHA** | Paguma larvata | SARS-CoV | AY545918 | 2013-07-05 | Asia | China | Asia | China | | genome | 29707 | Intermediate |
| **I3-MM-CHA** | Meles meles | SARS-CoV | AY545919 | 2013-07-05 | Asia | China | Asia | China | | genome | 29707 | Intermediate |
| **H1-USA** | Homo sapiens | SARS-CoV | MK062179 | 2017-05-27 | North America | USA | North America | USA | Cell culture | genome | 29727 | Human |
| **H2-USA** | Homo sapiens | SARS-CoV | MK062180 | 2017-05-28 | North America | USA | North America | USA | Cell culture | genome | 29727 | Human |
| **H3-USA** | Homo sapiens | SARS-CoV | MK062181 | 2017-05-29 | North America | USA | North America | USA | Cell culture | genome | 29727 | Human |
| **H4-USA** | Homo sapiens | SARS-CoV | MK062182 | 2017-05-30 | North America | USA | North America | USA | Cell culture | genome | 29727 | Human |
| **H5-USA** | Homo sapiens | SARS-CoV | AY714217 | 2003-06-25 | North America | USA | North America | USA | Oronasopharynx | genome | 29727 | Human |
| **H6-TWN** | Homo sapiens | SARS-CoV | AY502930 | 2003-03-15 | Asia | Taiwan | Asia | Taiwan | Throat swab | genome | 29727 | Human |
| **H7-TWN** | Homo sapiens | SARS-CoV | AY502929 | 2003-03-15 | Asia | Taiwan | Asia | Taiwan | Throat swab | genome | 29727 | Human |
| **H8-TWN** | Homo sapiens | SARS-CoV | AY502928 | 2003-03-15 | Asia | Taiwan | Asia | Taiwan | Throat swab | genome | 29727 | Human |
| **H9-TWN** | Homo sapiens | SARS-CoV | AY502923 | 2003-03-15 | Asia | Taiwan | Asia | Taiwan | Throat swab | genome | 29727 | Human |
| **H10-TWN** | Homo sapiens | SARS-CoV | AY291451 | 2003-03-15 | Asia | Taiwan | Asia | Taiwan | Throat swab | genome | 29727 | Human |
| **H11-CHA** | Homo sapiens | SARS-CoV | AY310120 | 2003-06-25 | Asia | China | Asia | China | | genome | 29727 | Human |
| **H12-CHA** | Homo sapiens | SARS-CoV | AY278491 | 2003-03-01 | Asia | Hong Kong | Asia | Hong Kong | Cell culture | genome | 29727 | Human |
| **H13-CHA** | Homo sapiens | SARS-CoV | AY278487 | 2003-06-25 | Asia | China | Asia | China | | genome | 29727 | Human |
| **H14-CAD-RE** | Homo sapiens | SARS-CoV | NC_004718 | 2003-06-25 | North America | Canada | North America | Canada | Cell culture | genome | 29727 | Human |

Material_S1_MERS-metadata

| strain | specie | virus | genbank_accession | date | region | country | region_exposure | country_exposure | source | segment | length | host |
|---|---|---|---|---|---|---|---|---|---|---|---|---|
| **B1-HS-ITY** | Hypsugo savii | MERS-CoV | MG596802 | 2014-07-03 | Europe | Italy | Europe | Italy | Pools of viscera | genome | 30027 | Bat |
| **B2-PK-ITY** | Pipistrellus kuhlii | MERS-CoV | MG596803 | 2014-07-03 | Europe | Italy | Europe | Italy | Pools of viscera | genome | 30028 | Bat |
| **I1-CD-SDA** | Camelus dromedarius | MERS-CoV | MN654975 | 2016-07-01 | Asia | Saudi Arabia | Asia | Saudi Arabia | Oronasopharynx | genome | 30106 | Intermediate |
| **I2-CD-SDA** | Camelus dromedarius | MERS-CoV | MN654988 | 2016-07-02 | Asia | Saudi Arabia | Asia | Saudi Arabia | Oronasopharynx | genome | 30106 | Intermediate |
| **I3-CD-SDA** | Camelus dromedarius | MERS-CoV | MT226600 | 2017-02-01 | Asia | Saudi Arabia | Asia | Saudi Arabia | Oronasopharynx | genome | 30106 | Intermediate |
| **I4-CD-SDA** | Camelus dromedarius | MERS-CoV | MT226602 | 2017-02-01 | Asia | Saudi Arabia | Asia | Saudi Arabia | Oronasopharynx | genome | 30106 | Intermediate |
| **I5-CD-SDA** | Camelus dromedarius | MERS-CoV | MT226605 | 2017-02-01 | Asia | Saudi Arabia | Asia | Saudi Arabia | Oronasopharynx | genome | 30106 | Intermediate |
| **I6-CD-SDA** | Camelus dromedarius | MERS-CoV | MT226604 | 2017-02-01 | Asia | Saudi Arabia | Asia | Saudi Arabia | Oronasopharynx | genome | 30106 | Intermediate |
| **I7-C-SDA** | Camelus | MERS-CoV | KT368830 | 2014-12-01 | Asia | Saudi Arabia | Asia | Saudi Arabia | Oronasopharynx | genome | 30106 | Intermediate |
| **I8-CD-SDA** | Camelus dromedarius | MERS-CoV | KJ713297 | 2013-11-01 | Asia | Saudi Arabia | Asia | Saudi Arabia | Oronasopharynx | genome | 30106 | Intermediate |
| **I9-C-SDA** | Camelus | MERS-CoV | KT368824 | 2013-11-01 | Asia | Saudi Arabia | Asia | Saudi Arabia | Oronasopharynx | genome | 30106 | Intermediate |
| **I10-CD-UAE** | Camelus dromedarius | MERS-CoV | MF598637 | 2015-03-01 | Asia | United Arab Emirates | Asia | United Arab Emirates | Oronasopharynx | genome | 30106 | Intermediate |
| **I11-CD-UAE** | Camelus dromedarius | MERS-CoV | MF598649 | 2015-03-01 | Asia | United Arab Emirates | Asia | United Arab Emirates | Oronasopharynx | genome | 30106 | Intermediate |
| **I12-CD-UAE** | Camelus dromedarius | MERS-CoV | MF598635 | 2015-03-01 | Asia | United Arab Emirates | Asia | United Arab Emirates | Oronasopharynx | genome | 30106 | Intermediate |
| **I13-CD-UAE** | Camelus dromedarius | MERS-CoV | MF598653 | 2015-03-01 | Asia | United Arab Emirates | Asia | United Arab Emirates | Oronasopharynx | genome | 30106 | Intermediate |
| **I14-CD-UAE** | Camelus dromedarius | MERS-CoV | MF598656 | 2015-03-01 | Asia | United Arab Emirates | Asia | United Arab Emirates | Oronasopharynx | genome | 30106 | Intermediate |
| **I15-CD-UAE** | Camelus dromedarius | MERS-CoV | MF598629 | 2015-03-01 | Asia | United Arab Emirates | Asia | United Arab Emirates | Oronasopharynx | genome | 30106 | Intermediate |
| **I16-C-OMN** | Camelus | MERS-CoV | KY673149 | 2015-01-01 | Asia | Oman | Asia | Oman | Oronasopharynx | genome | 30106 | Intermediate |
| **I17-CD-UAE** | Camelus dromedarius | MERS-CoV | MF598621 | 2015-03-01 | Asia | United Arab Emirates | Asia | United Arab Emirates | Oronasopharynx | genome | 30106 | Intermediate |
| **I18-CD-UAE** | Camelus dromedarius | MERS-CoV | MF598696 | 2015-03-01 | Asia | United Arab Emirates | Asia | United Arab Emirates | Oronasopharynx | genome | 30106 | Intermediate |
| **I19-C-UAE** | Camelus | MERS-CoV | KY581695 | 2014-06-01 | Asia | United Arab Emirates | Asia | United Arab Emirates | Oronasopharynx | genome | 30106 | Intermediate |
| **I20-CD-UAE** | Camelus dromedarius | MERS-CoV | MF598654 | 2015-03-01 | Asia | United Arab Emirates | Asia | United Arab Emirates | Oronasopharynx | genome | 30106 | Intermediate |
| **I21-C-SDA** | Camelus | MERS-CoV | KT368825 | 2014-07-01 | Asia | Saudi Arabia | Asia | Saudi Arabia | Oronasopharynx | genome | 30106 | Intermediate |
| **I22-C-SDA** | Camelus | MERS-CoV | KT368890 | 2015-03-01 | Asia | Saudi Arabia | Asia | Saudi Arabia | Oronasopharynx | genome | 30106 | Intermediate |
| **I23-CD-UAE** | Camelus dromedarius | MERS-CoV | MF598669 | 2015-03-01 | Asia | United Arab Emirates | Asia | United Arab Emirates | Oronasopharynx | genome | 30106 | Intermediate |
| **I24-CD-UAE** | Camelus dromedarius | MERS-CoV | MF598641 | 2015-03-01 | Asia | United Arab Emirates | Asia | United Arab Emirates | Oronasopharynx | genome | 30106 | Intermediate |
| **I25-CD-UAE** | Camelus dromedarius | MERS-CoV | MF598679 | 2015-03-01 | Asia | United Arab Emirates | Asia | United Arab Emirates | Oronasopharynx | genome | 30106 | Intermediate |
| **I26-CD-UAE** | Camelus dromedarius | MERS-CoV | MF598599 | 2015-03-01 | Asia | United Arab Emirates | Asia | United Arab Emirates | Oronasopharynx | genome | 30106 | Intermediate |
| **I27-CD-UAE** | Camelus dromedarius | MERS-CoV | MF598638 | 2015-03-01 | Asia | United Arab Emirates | Asia | United Arab Emirates | Oronasopharynx | genome | 30106 | Intermediate |
| **I28-CD-UAE** | Camelus dromedarius | MERS-CoV | MF598652 | 2015-03-01 | Asia | United Arab Emirates | Asia | United Arab Emirates | Oronasopharynx | genome | 30106 | Intermediate |
| **I29-CD-UAE** | Camelus dromedarius | MERS-CoV | MF598676 | 2015-03-01 | Asia | United Arab Emirates | Asia | United Arab Emirates | Oronasopharynx | genome | 30106 | Intermediate |
| **I30-CD-UAE** | Camelus dromedarius | MERS-CoV | MF598597 | 2015-03-01 | Asia | United Arab Emirates | Asia | United Arab Emirates | Oronasopharynx | genome | 30106 | Intermediate |
| **I31-CD-UAE** | Camelus dromedarius | MERS-CoV | MF598644 | 2015-03-01 | Asia | United Arab Emirates | Asia | United Arab Emirates | Oronasopharynx | genome | 30106 | Intermediate |
| **I32-CD-UAE** | Camelus dromedarius | MERS-CoV | MF598624 | 2015-03-01 | Asia | United Arab Emirates | Asia | United Arab Emirates | Oronasopharynx | genome | 30106 | Intermediate |
| **I33-CD-UAE** | Camelus dromedarius | MERS-CoV | MF598695 | 2015-03-01 | Asia | United Arab Emirates | Asia | United Arab Emirates | Oronasopharynx | genome | 30106 | Intermediate |

| | | | | | | | | | | | | |
|---|---|---|---|---|---|---|---|---|---|---|---|---|
| **I34-CD-UAE** | Camelus dromedarius | MERS-CoV | MF598651 | 2015-03-01 | Asia | United Arab Emirates | Asia | United Arab Emirates | Oronasopharynx | genome | 30106 | Intermediate |
| **I35-CD-UAE** | Camelus dromedarius | MERS-CoV | MF598609 | 2015-03-01 | Asia | United Arab Emirates | Asia | United Arab Emirates | Oronasopharynx | genome | 30106 | Intermediate |
| **I36-CD-UAE** | Camelus dromedarius | MERS-CoV | MF598712 | 2015-03-01 | Asia | United Arab Emirates | Asia | United Arab Emirates | Oronasopharynx | genome | 30106 | Intermediate |
| **I37-CD-UAE** | Camelus dromedarius | MERS-CoV | MF598616 | 2015-03-01 | Asia | United Arab Emirates | Asia | United Arab Emirates | Oronasopharynx | genome | 30106 | Intermediate |
| **I38-CD-UAE** | Camelus dromedarius | MERS-CoV | MF598702 | 2015-03-01 | Asia | United Arab Emirates | Asia | United Arab Emirates | Oronasopharynx | genome | 30106 | Intermediate |
| **I39-CD-UAE** | Camelus dromedarius | MERS-CoV | MF598632 | 2015-03-01 | Asia | United Arab Emirates | Asia | United Arab Emirates | Oronasopharynx | genome | 30106 | Intermediate |
| **I40-CD-UAE** | Camelus dromedarius | MERS-CoV | MF598617 | 2015-03-01 | Asia | United Arab Emirates | Asia | United Arab Emirates | Oronasopharynx | genome | 30106 | Intermediate |
| **I41-CD-UAE** | Camelus dromedarius | MERS-CoV | MF598668 | 2015-03-01 | Asia | United Arab Emirates | Asia | United Arab Emirates | Oronasopharynx | genome | 30106 | Intermediate |
| **I42-CD-UAE** | Camelus dromedarius | MERS-CoV | MF598701 | 2015-03-01 | Asia | United Arab Emirates | Asia | United Arab Emirates | Oronasopharynx | genome | 30106 | Intermediate |
| **I43-CD-UAE** | Camelus dromedarius | MERS-CoV | MF598620 | 2015-03-01 | Asia | United Arab Emirates | Asia | United Arab Emirates | Oronasopharynx | genome | 30106 | Intermediate |
| **I44-CD-UAE** | Camelus dromedarius | MERS-CoV | MF598594 | 2015-03-01 | Asia | United Arab Emirates | Asia | United Arab Emirates | Oronasopharynx | genome | 30106 | Intermediate |
| **I45-CD-UAE** | Camelus dromedarius | MERS-CoV | MF598643 | 2015-03-01 | Asia | United Arab Emirates | Asia | United Arab Emirates | Oronasopharynx | genome | 30106 | Intermediate |
| **H1-USA** | Homo sapiens | MERS-CoV | KP223131 | 2014-06-01 | North America | USA | North America | USA | Lung, Oronasopharynx | genome | 30106 | Human |
| **H2-USA** | Homo sapiens | MERS-CoV | MK039552 | 2014-04-21 | Asia | Jordan | Asia | Jordan | Lung, Oronasopharynx | genome | 30106 | Human |
| **H3-SDA** | Homo sapiens | MERS-CoV | KF186567 | 2013-05-09 | Asia | Saudi Arabia | Asia | Saudi Arabia | | genome | 30106 | Human |
| **H4-OMN** | Homo sapiens | MERS-CoV | KY673148 | 2015-01-06 | Asia | Oman | Asia | Oman | Oronasopharynx | genome | 30106 | Human |
| **H5-UAE** | Homo sapiens | MERS-CoV | KP209310 | 2014-03-07 | Asia | United Arab Emirates | Asia | United Arab Emirates | Lung, Oronasopharynx | genome | 30106 | Human |
| **H6-UAE** | Homo sapiens | MERS-CoV | KY581686 | 2014-04-10 | Asia | United Arab Emirates | Asia | United Arab Emirates | Lung, Oronasopharynx | genome | 30106 | Human |
| **H7-UAE** | Homo sapiens | MERS-CoV | KP209307 | 2014-04-10 | Asia | United Arab Emirates | Asia | United Arab Emirates | Lung, Oronasopharynx | genome | 30106 | Human |
| **H8-UAE** | Homo sapiens | MERS-CoV | KY581694 | 2014-01-01 | Asia | United Arab Emirates | Asia | United Arab Emirates | Lung, Oronasopharynx | genome | 30106 | Human |
| **H9-OMN** | Homo sapiens | MERS-CoV | KT156561 | 2013-12-28 | Asia | Oman | Asia | Oman | Oronasopharynx | genome | 30106 | Human |
| **H10-UAE** | Homo sapiens | MERS-CoV | KY581684 | 2013-07-10 | Asia | United Arab Emirates | Asia | United Arab Emirates | Lung, Oronasopharynx | genome | 30106 | Human |
| **H11-UAE** | Homo sapiens | MERS-CoV | KP209312 | 2013-11-15 | Asia | United Arab Emirates | Asia | United Arab Emirates | Lung | genome | 30106 | Human |
| **H12-UAE** | Homo sapiens | MERS-CoV | KY581687 | 2013-12-23 | Asia | United Arab Emirates | Asia | United Arab Emirates | Lung, Oronasopharynx | genome | 30106 | Human |
| **H13-OMN** | Homo sapiens | MERS-CoV | KT156560 | 2013-10-28 | Asia | Oman | Asia | Oman | Oronasopharynx | genome | 30106 | Human |
| **H14-SDA** | Homo sapiens | MERS-CoV | MH013216 | 2015-10-15 | Asia | Saudi Arabia | Asia | Saudi Arabia | | genome | 30106 | Human |
| **H15-SDA** | Homo sapiens | MERS-CoV | MG912603 | 2017-06-12 | Asia | Saudi Arabia | Asia | Saudi Arabia | Oronasopharynx | genome | 30106 | Human |
| **H16-SDA** | Homo sapiens | MERS-CoV | MG546331 | 2015-10-15 | Asia | Saudi Arabia | Asia | Saudi Arabia | Oronasopharynx | genome | 30106 | Human |
| **H17-SKA** | Homo sapiens | MERS-CoV | MH029552 | 2015-08-26 | Asia | Saudi Arabia | Asia | Saudi Arabia | Oronasopharynx | genome | 30106 | Human |
| **H18-SKA** | Homo sapiens | MERS-CoV | KX034099 | 2015-06-26 | Asia | South Korea | Asia | South Korea | Lung, Oronasopharynx | genome | 30106 | Human |
| **H19-SKA** | Homo sapiens | MERS-CoV | KX034095 | 2015-06-04 | Asia | South Korea | Asia | South Korea | Lung, Oronasopharynx | genome | 30106 | Human |
| **H20-SDA** | Homo sapiens | MERS-CoV | KT026454 | 2015-03-01 | Asia | Saudi Arabia | Asia | Saudi Arabia | Oronasopharynx | genome | 30106 | Human |
| **H21-SDA** | Homo sapiens | MERS-CoV | MN120513 | 2019-03-27 | Asia | Saudi Arabia | Asia | Saudi Arabia | | genome | 30106 | Human |
| **H22-SDA** | Homo sapiens | MERS-CoV | MN120514 | 2019-03-27 | Asia | Saudi Arabia | Asia | Saudi Arabia | | genome | 30106 | Human |
| **H23-SDA** | Homo sapiens | MERS-CoV | MK462249 | 2018-07-09 | Asia | Saudi Arabia | Asia | Saudi Arabia | Oronasopharynx | genome | 30106 | Human |
| **H24-SDA** | Homo sapiens | MERS-CoV | MK483839 | 2018-08-16 | Asia | Saudi Arabia | Asia | Saudi Arabia | Oronasopharynx | genome | 30106 | Human |
| **H25-SDA** | Homo sapiens | MERS-CoV | MK462250 | 2018-07-12 | Asia | Saudi Arabia | Asia | Saudi Arabia | Oronasopharynx | genome | 30106 | Human |

| | | | | | | | | | | | | |
|---|---|---|---|---|---|---|---|---|---|---|---|---|
| **H26-SDA** | Homo sapiens | MERS-CoV | MK462248 | 2018-06-13 | Asia | Saudi Arabia | Asia | Saudi Arabia | Oronasopharynx | genome | 30106 | Human |
| **H27-SDA** | Homo sapiens | MERS-CoV | MN365232 | 2019-01-28 | Asia | Saudi Arabia | Asia | Saudi Arabia | Oronasopharynx | genome | 30106 | Human |
| **H28-SDA** | Homo sapiens | MERS-CoV | MN723544 | 2018-08-30 | Asia | Saudi Arabia | Asia | Saudi Arabia | | genome | 30106 | Human |
| **H29-SDA** | Homo sapiens | MERS-CoV | MK462254 | 2018-08-31 | Asia | Saudi Arabia | Asia | Saudi Arabia | Oronasopharynx | genome | 30106 | Human |
| **H30-SDA** | Homo sapiens | MERS-CoV | MK462251 | 2018-07-18 | Asia | Saudi Arabia | Asia | Saudi Arabia | Oronasopharynx | genome | 30106 | Human |
| **H31-SDA** | Homo sapiens | MERS-CoV | MK462247 | 2018-06-08 | Asia | Saudi Arabia | Asia | Saudi Arabia | Oronasopharynx | genome | 30106 | Human |
| **H32-SDA** | Homo sapiens | MERS-CoV | MK462256 | 2018-09-15 | Asia | Saudi Arabia | Asia | Saudi Arabia | Oronasopharynx | genome | 30106 | Human |
| **H33-SDA** | Homo sapiens | MERS-CoV | MK462252 | 2018-08-03 | Asia | Saudi Arabia | Asia | Saudi Arabia | Oronasopharynx | genome | 30106 | Human |
| **H34-SDA** | Homo sapiens | MERS-CoV | MK462244 | 2017-08-07 | Asia | Saudi Arabia | Asia | Saudi Arabia | Oronasopharynx | genome | 30106 | Human |
| **H35-SDA-RE** | Homo sapiens | MERS-CoV | NC_019843 | 2012-06-13 | Asia | Saudi Arabia | Asia | Saudi Arabia | Oronasopharynx | genome | 30106 | Human |

Material_S1_SARS2-metadata

| strain | specie | virus | gisaid_epi_isl | date | region | country | region_exposure | country_exposure | source | segment | length | host | pango_lineage | clade |
|---|---|---|---|---|---|---|---|---|---|---|---|---|---|---|
| **B1-RS-CMD** | Rhinolophus shameli | SARS-CoV-2 | EPI_ISL_852604 | 2010-12-06 | Asia | Cambodia | Asia | Cambodia | Feces | genome | 29767 | Bat | A | S |
| **B2-RS-CMD** | Rhinolophus shameli | SARS-CoV-2 | EPI_ISL_852605 | 2010-12-06 | Asia | Cambodia | Asia | Cambodia | Feces | genome | 29767 | Bat | A | S |
| **B3-RA-CHA** | Rhinolophus affinis | SARS-CoV-2 | EPI_ISL_402131 | 2013-07-24 | Asia | China | Asia | China | Feces | genome | 29844 | Bat | B.1.177 | S |
| **I1-MJ-CHA** | Manis javanica | SARS-CoV-2 | EPI_ISL_410721 | 2019-07-11 | Asia | China | Asia | China | Lung | genome | 29728 | Intermediate | A | S |
| **I2-PL-USA** | Panthera leo | SARS-CoV-2 | EPI_ISL_566038 | 2020-04-04 | North America | USA | North America | USA | Feces | genome | 29774 | Intermediate | B | V |
| **I3-PL-USA** | Panthera leo | SARS-CoV-2 | EPI_ISL_566044 | 2020-04-04 | North America | USA | North America | USA | Feces | genome | 29774 | Intermediate | B | V |
| **I4-EV-USA** | Environment | SARS-CoV-2 | EPI_ISL_434677 | 2020-03-25 | North America | USA | North America | USA | Air | genome | 29774 | Intermediate | A | S |
| **I5-MA-CHA** | Mesocricetus auratus | SARS-CoV-2 | | 2020-05-15 | Asia | China | Asia | China | Oronasopharynx | genome | 29774 | Intermediate | B.39 | |
| **I6-MA-CHA** | Mesocricetus auratus | SARS-CoV-2 | | 2020-05-15 | Asia | China | Asia | China | Oronasopharynx | genome | 29774 | Intermediate | B | |
| **I7-MA-CHA** | Mesocricetus auratus | SARS-CoV-2 | | 2020-05-15 | Asia | China | Asia | China | Oronasopharynx | genome | 29774 | Intermediate | B | |
| **I8-MA-CHA** | Mesocricetus auratus | SARS-CoV-2 | | 2020-05-15 | Asia | China | Asia | China | Oronasopharynx | genome | 29774 | Intermediate | B | |
| **I9-EV-CHA** | Environment | SARS-CoV-2 | EPI_ISL_408515 | 2020-01-01 | Asia | China | Asia | China | Huanan Seafood Market | genome | 29774 | Intermediate | B | L |
| **I10-EV-CHA** | Environment | SARS-CoV-2 | EPI_ISL_408514 | 2020-01-01 | Asia | China | Asia | China | Huanan Seafood Market | genome | 29774 | Intermediate | B | L |
| **I11-NV-LIT** | Neovison vison | SARS-CoV-2 | EPI_ISL_851056 | 2020-11-01 | Europe | Lithuania | Europe | Lithuania | Throat swab | genome | 29774 | Intermediate | B.1.1.464¬† | GR |
| **I12-NV-LIT** | Neovison vison | SARS-CoV-2 | EPI_ISL_851057 | 2020-11-01 | Europe | Lithuania | Europe | Lithuania | Throat swab | genome | 29774 | Intermediate | B.1.1.464¬† | GR |
| **I13-NV-DMK** | Neovison vison | SARS-CoV-2 | EPI_ISL_683175 | 2020-11-13 | Europe | Denmark | Europe | Denmark | Throat swab | genome | 29774 | Intermediate | B.1.1.219 | GR |
| **I14-NV-DMK** | Neovison vison | SARS-CoV-2 | EPI_ISL_641422 | 2020-10-16 | Europe | Denmark | Europe | Denmark | Throat swab | genome | 29774 | Intermediate | B.1.536 | G |
| **I15-NV-DMK** | Neovison vison | SARS-CoV-2 | EPI_ISL_683023 | 2020-11-04 | Europe | Denmark | Europe | Denmark | Throat swab | genome | 29774 | Intermediate | B.1.536 | G |
| **I16-NV-DMK** | Neovison vison | SARS-CoV-2 | EPI_ISL_683005 | 2020-11-05 | Europe | Denmark | Europe | Denmark | Throat swab | genome | 29774 | Intermediate | B.1.536 | G |
| **I17-NV-POL** | Neovison vison | SARS-CoV-2 | EPI_ISL_732949 | 2020-11-17 | Europe | Poland | Europe | Poland | Throat swab | genome | 29774 | Intermediate | B.1.1 | GR |
| **I18-NV-POL** | Neovison vison | SARS-CoV-2 | EPI_ISL_732955 | 2020-11-17 | Europe | Poland | Europe | Poland | Throat swab | genome | 29774 | Intermediate | B.1.1 | GR |
| **I19-NV-LIT** | Neovison vison | SARS-CoV-2 | EPI_ISL_851058 | 2020-11-01 | Europe | Lithuania | Europe | Lithuania | Throat swab | genome | 29774 | Intermediate | B.1.343 | GH |
| **I20-NV-LIT** | Neovison vison | SARS-CoV-2 | EPI_ISL_851060 | 2020-11-01 | Europe | Lithuania | Europe | Lithuania | Throat swab | genome | 29774 | Intermediate | B.1.343 | GH |
| **I21-FC-RUS** | Felis catus | SARS-CoV-2 | EPI_ISL_811147 | 2021-01-06 | Asia | Russia | Asia | Russia | Oropharyngeal swab | genome | 29774 | Intermediate | B.1.1.317 | GR |
| **I22-NV-DMK** | Neovison vison | SARS-CoV-2 | EPI_ISL_641414 | 2020-10-26 | Europe | Denmark | Europe | Denmark | Throat swab | genome | 29774 | Intermediate | B.1.1.170 | GR |
| **I23-NV-DMK** | Neovison vison | SARS-CoV-2 | EPI_ISL_683215 | 2020-10-23 | Europe | Denmark | Europe | Denmark | Throat swab | genome | 29774 | Intermediate | B.1.1.170¬† | GR |
| **I24-NV-NTL** | Neovison vison | SARS-CoV-2 | EPI_ISL_577802 | 2020-09-02 | Europe | Netherlands | Europe | Netherlands | Throat swab | genome | 29774 | Intermediate | B.1.22 | GH |
| **I25-NV-DMK** | Neovison vison | SARS-CoV-2 | EPI_ISL_683216 | 2020-10-23 | Europe | Denmark | Europe | Denmark | Throat swab | genome | 29774 | Intermediate | B.1.1.294 | GR |
| **I26-NV-DMK** | Neovison vison | SARS-CoV-2 | EPI_ISL_683218 | 2020-10-23 | Europe | Denmark | Europe | Denmark | Throat swab | genome | 29774 | Intermediate | B.1.1.294 | GR |
| **I27-NV-NTL** | Neovison vison | SARS-CoV-2 | EPI_ISL_431778 | 2020-04-24 | Europe | Netherlands | Europe | Netherlands | Lung | genome | 29774 | Intermediate | B.1.8 | G |
| **I28-NV-NTL** | Neovison vison | SARS-CoV-2 | EPI_ISL_522992 | 2020-04-28 | Europe | Netherlands | Europe | Netherlands | Throat swab | genome | 29774 | Intermediate | B.1.8 | G |
| **I29-NV-NTL** | Neovison vison | SARS-CoV-2 | EPI_ISL_447625 | 2020-04-29 | Europe | Netherlands | Europe | Netherlands | Throat swab | genome | 29774 | Intermediate | B.1.8 | G |
| **I30-NV-NTL** | Neovison vison | SARS-CoV-2 | EPI_ISL_523009 | 2020-06-07 | Europe | Netherlands | Europe | Netherlands | Throat swab | genome | 29774 | Intermediate | B.1.8 | G |
| **I31-NV-NTL** | Neovison vison | SARS-CoV-2 | EPI_ISL_523006 | 2020-06-07 | Europe | Netherlands | Europe | Netherlands | Throat swab | genome | 29774 | Intermediate | B.1.8 | G |
| **I32-CL-ITY** | Canis lupus familiaris | SARS-CoV-2 | EPI_ISL_730652 | 2020-11-04 | Europe | Italy | Europe | Italy | Nasopharyngeal swab | genome | 29774 | Intermediate | B.1.177 | GV |
| **I33-NV-NTL** | Neovison vison | SARS-CoV-2 | EPI_ISL_523083 | 2020-07-26 | Europe | Netherlands | Europe | Netherlands | Throat swab | genome | 29774 | Intermediate | B.1.149 | G |
| **I34-CL-USA** | Canis lupus familiaris | SARS-CoV-2 | EPI_ISL_699508 | 2020-07-28 | North America | USA | North America | USA | Respiratory swab | genome | 29774 | Intermediate | B.1.1 | GR |

| | | | | | | | | | | | | | | |
|---|---|---|---|---|---|---|---|---|---|---|---|---|---|---|
| **I35-NV-NTL** | Neovison vison | SARS-CoV-2 | EPI_ISL_523106 | 2020-05-30 | Europe | Netherlands | Europe | Netherlands | Throat swab | genome | 29774 | Intermediate | B.1.22 | GH |
| **I36-FC-USA** | Felis catus | SARS-CoV-2 | EPI_ISL_699507 | 2020-07-17 | North America | USA | North America | USA | Rectal swab | genome | 29774 | Intermediate | B.1.571 | GH |
| **I37-FC-USA** | Felis catus | SARS-CoV-2 | EPI_ISL_699506 | 2020-06-28 | North America | USA | North America | USA | Respiratory swab | genome | 29774 | Intermediate | B.1.234 | G |
| **I38-NV-NTL** | Neovison vison | SARS-CoV-2 | EPI_ISL_522995 | 2020-06-07 | Europe | Netherlands | Europe | Netherlands | Throat swab | genome | 29774 | Intermediate | B.1 | G |
| **I39-FC-FRE** | Felis catus | SARS-CoV-2 | EPI_ISL_483063 | 2020-05-14 | Europe | France | Europe | France | Home Environment | genome | 29774 | Intermediate | B.1.1.254 | GR |
| **I40-EV-CHA** | Environment | SARS-CoV-2 | EPI_ISL_469256 | 2020-06-11 | Asia | China | Asia | China | Xinfadi Wholesale Market | genome | 29774 | Intermediate | B.1.1 | GR |
| **I41-EV-CHA** | Environment | SARS-CoV-2 | EPI_ISL_430743 | 2020-03-14 | Asia | China | Asia | China | Enviromental swab | genome | 29774 | Intermediate | B.1 | G |
| **I42-PT-USA** | Panthera tigris jacksoni | SARS-CoV-2 | EPI_ISL_566040 | 2020-04-04 | North America | USA | North America | USA | Feces | genome | 29774 | Intermediate | B.1 | GH |
| **I43-FC-CHA** | Felis catus | SARS-CoV-2 | EPI_ISL_759858 | 2020-03-30 | Asia | China | Asia | China | | genome | 29774 | Intermediate | B.1 | G |
| **I44-EV-USA** | Environment | SARS-CoV-2 | EPI_ISL_455682 | 2020-04-23 | North America | USA | North America | USA | Air | genome | 29774 | Intermediate | B.1.595 | GH |
| **I45-EV-USA** | Environment | SARS-CoV-2 | EPI_ISL_477160 | 2020-10-16 | North America | USA | North America | USA | Hospital Air | genome | 29774 | Intermediate | B.1 | GH |
| **I46-PT-USA** | Panthera tigris jacksoni | SARS-CoV-2 | EPI_ISL_420293 | 2020-04-02 | North America | USA | North America | USA | Nasal swab | genome | 29774 | Intermediate | B.1 | GH |
| **I47-FC-BEL** | Felis catus | SARS-CoV-2 | EPI_ISL_487275 | 2020-03-11 | Europe | Belgium | Europe | Belgium | Vomit fluid | genome | 29774 | Intermediate | B.1 | G |
| **I48-MA-CHA** | Mesocricetus auratus | SARS-CoV-2 | | 2020-05-15 | Asia | China | Asia | China | Oronasopharynx | genome | 29774 | Intermediate | B.1 | |
| **H1-SKA** | Homo sapiens | SARS-CoV-2 | | 2020-03-11 | Asia | South Korea | Asia | South Korea | Oronasopharynx | genome | 29822 | Human | A | |
| **H2-ENG** | Homo sapiens | SARS-CoV-2 | | 2020-12-17 | Europe | England | Europe | England | Oropharyngeal swab | genome | 29822 | Human | B.1.1.7¬† | |
| **H3-ENG** | Homo sapiens | SARS-CoV-2 | | 2020-08-23 | Europe | England | Europe | England | Oropharyngeal swab | genome | 29822 | Human | B.1.177 | |
| **H4-CHA** | Homo sapiens | SARS-CoV-2 | | 2020-01-22 | Asia | China | Asia | China | Oropharyngeal swabs | genome | 29822 | Human | B | |
| **H5-CHA** | Homo sapiens | SARS-CoV-2 | | 2020-02-10 | Asia | China | Asia | China | Oronasopharynx | genome | 29822 | Human | B.5 | |
| **H6-CHA** | Homo sapiens | SARS-CoV-2 | | 2020-01-22 | Asia | China | Asia | China | Oronasopharynx | genome | 29822 | Human | B | |
| **H7-CHA** | Homo sapiens | SARS-CoV-2 | | 2020-01-22 | Asia | China | Asia | China | Oronasopharynx | genome | 29822 | Human | A | |
| **H8-CHA** | Homo sapiens | SARS-CoV-2 | | 2020-02-02 | Asia | China | Asia | China | Oronasopharynx | genome | 29822 | Human | B | |
| **H9-CHA-RE** | Homo sapiens | SARS-CoV-2 | | 2019-12-01 | Asia | China | Asia | China | Oronasopharynx | genome | 29822 | Human | B | |
| **H10-ITY** | Homo sapiens | SARS-CoV-2 | | 2020-12-27 | Europe | Italy | Europe | Italy | Oronasopharynx | genome | 29822 | Human | B.1.1.7 | |
| **H11-PAL** | Homo sapiens | SARS-CoV-2 | EPI_ISL_1502574 | 2021-02-06 | Asia | Palestine | Asia | Palestine | Nasal swab | genome | 29822 | Human | B.1.1.7 | GR |
| **H12-LET** | Homo sapiens | SARS-CoV-2 | EPI_ISL_1590998 | 2021-03-17 | Europe | Latvia | Europe | Latvia | Nasal swab | genome | 29822 | Human | B.1.177.79 | GV |
| **H13-LET** | Homo sapiens | SARS-CoV-2 | EPI_ISL_1590430 | 2021-03-09 | Europe | Latvia | Europe | Latvia | Nasal swab | genome | 29822 | Human | S.1 | GR |
| **H14-LET** | Homo sapiens | SARS-CoV-2 | EPI_ISL_1590432 | 2021-03-09 | Europe | Latvia | Europe | Latvia | Nasal swab | genome | 29822 | Human | S.1 | GR |
| **H15-MYA** | Homo sapiens | SARS-CoV-2 | EPI_ISL_1424467 | 2021-03-02 | Asia | Malaysia | Asia | Malaysia | Nasal swab | genome | 29822 | Human | B.1.466.2 | GH |
| **H16-MYA** | Homo sapiens | SARS-CoV-2 | EPI_ISL_1424470 | 2021-03-01 | Asia | Malaysia | Asia | Malaysia | Nasal swab | genome | 29822 | Human | B.1.466.2 | GH |
| **H17-USA** | Homo sapiens | SARS-CoV-2 | | 2021-02-13 | North America | USA | North America | USA | Oronasopharynx | genome | 29822 | Human | B.1.1.519 | |
| **H18-ENG** | Homo sapiens | SARS-CoV-2 | | 2020-12-19 | Europe | England | Europe | England | Oropharyngeal swab | genome | 29822 | Human | B.1.1.519 | |
| **H19-TUS** | Homo sapiens | SARS-CoV-2 | EPI_ISL_635062 | 2020-11-02 | Africa | Tunisia | Africa | Tunisia | Nasopharyngeal s | genome | 29822 | Human | B.1.160 | GH |
| **H20-ENG** | Homo sapiens | SARS-CoV-2 | | 2020-09-18 | Europe | England | Europe | England | Oropharyngeal swab | genome | 29822 | Human | B.1.160 | |
| **H21-LET** | Homo sapiens | SARS-CoV-2 | EPI_ISL_1590520 | 2021-03-09 | Europe | Latvia | Europe | Latvia | Nasal swab | genome | 29822 | Human | B.1.1.374 | GR |
| **H22-LET** | Homo sapiens | SARS-CoV-2 | EPI_ISL_1591003 | 2021-03-17 | Europe | Latvia | Europe | Latvia | Nasal swab | genome | 29822 | Human | B.1.1.374 | GR |
| **H23-USA** | Homo sapiens | SARS-CoV-2 | | 2021-02-18 | North America | USA | North America | USA | Oronasopharynx | genome | 29822 | Human | B.1.561 | |
| **H24-BRL** | Homo sapiens | SARS-CoV-2 | EPI_ISL_1358305 | 2021-03-02 | South America | Brazil | South America | Brazil | Nasopharyngeal swab | genome | 29822 | Human | B.1.1.28 | GR |
| **H25-EGP** | Homo sapiens | SARS-CoV-2 | | 2020-07-12 | Africa | Egypt | Africa | Egypt | Oronasopharynx | genome | 29822 | Human | B.1.561 | |
| **H26-BRL** | Homo sapiens | SARS-CoV-2 | EPI_ISL_1078991 | 2021-01-22 | South America | Brazil | South America | Brazil | Nasopharyngeal swab | genome | 29822 | Human | B.1.1.28 | GR |

| | | | | | | | | | | | | | | |
|---|---|---|---|---|---|---|---|---|---|---|---|---|---|---|
| **H27-COL** | Homo sapiens | SARS-CoV-2 | EPI_ISL_1235687 | 2021-01-30 | South America | Colombia | South America | Colombia | Oropharyngeal swab | genome | 29822 | Human | B.1.111 | GH |
| **H28-USA** | Homo sapiens | SARS-CoV-2 | | 2021-02-16 | North America | USA | North America | USA | Oronasopharynx | genome | 29822 | Human | B.1.2 | |
| **H29-USA** | Homo sapiens | SARS-CoV-2 | | 2021-03-08 | North America | USA | North America | USA | Oronasopharynx | genome | 29822 | Human | B.1.1.274 | |
| **H30-USA** | Homo sapiens | SARS-CoV-2 | | 2021-02-22 | North America | USA | North America | USA | Oronasopharynx | genome | 29822 | Human | B.1 | |
| **H31-BRL** | Homo sapiens | SARS-CoV-2 | EPI_ISL_1293079 | 2021-02-02 | South America | Brazil | South America | Brazil | Nasopharyngeal swab | genome | 29822 | Human | P.2 | GH |
| **H32CLE** | Homo sapiens | SARS-CoV-2 | EPI_ISL_1167710 | 2020-11-15 | South America | Chile | South America | Chile | Nasopharyngeal swab | genome | 29822 | Human | B.1.1.1¬† | GR |
| **H33-CLE** | Homo sapiens | SARS-CoV-2 | EPI_ISL_1167701 | 2020-10-11 | South America | Chile | South America | Chile | Nasopharyngeal swab | genome | 29822 | Human | C.26¬† | GR |
| **H34-USA** | Homo sapiens | SARS-CoV-2 | | 2021-02-24 | North America | USA | North America | USA | Oronasopharynx | genome | 29822 | Human | B.1.478 | |
| **H35-USA** | Homo sapiens | SARS-CoV-2 | | 2021-03-12 | North America | USA | North America | USA | Oronasopharynx | genome | 29822 | Human | B.1.429 | |
| **H36-USA** | Homo sapiens | SARS-CoV-2 | | 2021-03-04 | North America | USA | North America | USA | Oronasopharynx | genome | 29822 | Human | B.1.2 | |
| **H37-LET** | Homo sapiens | SARS-CoV-2 | EPI_ISL_1591001 | 2021-03-17 | Europe | Latvia | Europe | Latvia | Nasal swab | genome | 29822 | Human | B.1.177¬† | GV |
| **H38-LET** | Homo sapiens | SARS-CoV-2 | EPI_ISL_1590465 | 2021-03-16 | Europe | Latvia | Europe | Latvia | Nasal swab | genome | 29822 | Human | B.1.177¬† | GV |
| **H39-BRL** | Homo sapiens | SARS-CoV-2 | EPI_ISL_1121317 | 2021-02-09 | South America | Brazil | South America | Brazil | Nasopharyngeal swab | genome | 29822 | Human | B.1.1.28 | GR |
| **H40-BRL** | Homo sapiens | SARS-CoV-2 | EPI_ISL_1533705 | 2021-02-21 | South America | Brazil | South America | Brazil | Nasopharyngeal swab | genome | 29822 | Human | P.2 | GR |
| **H41-USA** | Homo sapiens | SARS-CoV-2 | | 2021-02-22 | North America | USA | North America | USA | Oronasopharynx | genome | 29822 | Human | B.1.36.31 | |
| **H42-BRL** | Homo sapiens | SARS-CoV-2 | EPI_ISL_1445177 | 2021-03-10 | South America | Brazil | South America | Brazil | Nasopharyngeal swab | genome | 29822 | Human | B.1.1¬† | GR |
| **H43-USA** | Homo sapiens | SARS-CoV-2 | | 2021-04-02 | North America | USA | North America | USA | Oronasopharynx | genome | 29822 | Human | B.1 | |
| **H44-SEN** | Homo sapiens | SARS-CoV-2 | EPI_ISL_1167174 | 2021-01-25 | Africa | Senegal | Africa | Senegal | Oropharyngeal swab | genome | 29822 | Human | B.1.1.420 | O |
| **H45-LET** | Homo sapiens | SARS-CoV-2 | EPI_ISL_1590519 | 2021-03-09 | Europe | Latvia | Europe | Latvia | Nasal swab | genome | 29822 | Human | B.1.177 | GV |
| **H46-USA** | Homo sapiens | SARS-CoV-2 | | 2021-02-19 | North America | USA | North America | USA | Oronasopharynx | genome | 29822 | Human | B.1.561 | |
| **H47-EGP** | Homo sapiens | SARS-CoV-2 | | 2020-07-04 | Africa | Egypt | Africa | Egypt | Oronasopharynx | genome | 29822 | Human | B.1 | |
| **H48-BRL** | Homo sapiens | SARS-CoV-2 | EPI_ISL_1079159 | 2021-02-03 | South America | Brazil | South America | Brazil | Nasopharyngeal swab | genome | 29822 | Human | N.9¬† | GR |
| **H49-PER** | Homo sapiens | SARS-CoV-2 | EPI_ISL_1111317 | 2021-01-17 | South America | Peru | South America | Peru | Oropharyngeal swab | genome | 29822 | Human | B.1.1.1 | GR |
| **H50-PER** | Homo sapiens | SARS-CoV-2 | EPI_ISL_1111348 | 2021-01-16 | South America | Peru | South America | Peru | Oropharyngeal swab | genome | 29822 | Human | C.4 | GR |
| **H51-ITY** | Homo sapiens | SARS-CoV-2 | | 2020-12-10 | Europe | Italy | Europe | Italy | Oronasopharynx | genome | 29822 | Human | B.1.258 | |
| **H52-LET** | Homo sapiens | SARS-CoV-2 | EPI_ISL_1590935 | 2021-03-09 | Europe | Latvia | Europe | Latvia | Nasal swab | genome | 29822 | Human | B.1.1.374 | GR |
| **H53-LET** | Homo sapiens | SARS-CoV-2 | EPI_ISL_1590995 | 2021-03-16 | Europe | Latvia | Europe | Latvia | Nasal swab | genome | 29822 | Human | B.1.177.12¬† | GV |
| **H54-STD** | Homo sapiens | SARS-CoV-2 | | 2020-10-14 | Europe | Scotland | Europe | Scotland | Oropharyngeal swab | genome | 29822 | Human | B.1.177.57 | |
| **H55-USA** | Homo sapiens | SARS-CoV-2 | | 2021-03-03 | North America | USA | North America | USA | Oronasopharynx | genome | 29822 | Human | B.1.1.432 | |
| **H56-GHA** | Homo sapiens | SARS-CoV-2 | EPI_ISL_1018073 | 2021-01-10 | Africa | Ghana | Africa | Ghana | Nasopharyngeal swab | genome | 29822 | Human | L.3¬† | GR |
| **H57-BRL** | Homo sapiens | SARS-CoV-2 | EPI_ISL_1445201 | 2021-03-15 | South America | Brazil | South America | Brazil | Nasopharyngeal swab | genome | 29822 | Human | B.1.1.28 | GR |
| **H58-COL** | Homo sapiens | SARS-CoV-2 | EPI_ISL_1577390 | 2021-01-08 | South America | Colombia | South America | Colombia | Oropharyngeal swab | genome | 29822 | Human | B.1.1.348 | GR |
| **H59-COL** | Homo sapiens | SARS-CoV-2 | EPI_ISL_1577026 | 2020-10-27 | South America | Colombia | South America | Colombia | Oropharyngeal swab | genome | 29822 | Human | B.1.1.348 | GR |
| **H60-COL** | Homo sapiens | SARS-CoV-2 | EPI_ISL_1576835 | 2020-10-25 | South America | Colombia | South America | Colombia | Oropharyngeal swab | genome | 29822 | Human | B.1.1.348 | GR |
| **H61-LET** | Homo sapiens | SARS-CoV-2 | EPI_ISL_1590933 | 2021-03-09 | Europe | Latvia | Europe | Latvia | Nasal swab | genome | 29822 | Human | B.1.1.429 | GR |
| **H62-LET** | Homo sapiens | SARS-CoV-2 | EPI_ISL_1590947 | 2021-03-15 | Europe | Latvia | Europe | Latvia | Nasal swab | genome | 29822 | Human | Z.1¬† | GV |
| **H63-LET** | Homo sapiens | SARS-CoV-2 | EPI_ISL_1590894 | 2021-03-09 | Europe | Latvia | Europe | Latvia | Nasal swab | genome | 29822 | Human | B.1.177 | GV |
| **H64-CJD** | Homo sapiens | SARS-CoV-2 | | 2020-11-29 | Asia | West Blank | Asia | West Blank | Oronasopharynx | genome | 29822 | Human | B.1.1.50 | |
| **H65-ENG** | Homo sapiens | SARS-CoV-2 | | 2020-08-28 | Europe | England | Europe | England | Oropharyngeal swab | genome | 29822 | Human | B.1.367 | |
| **H66-USA** | Homo sapiens | SARS-CoV-2 | | 2021-03-10 | North America | USA | North America | USA | Oronasopharynx | genome | 29822 | Human | B.1.427 | |

| | | | | | | | | | | | | | | | |
|---|---|---|---|---|---|---|---|---|---|---|---|---|---|---|---|
| **H67-ENG** | Homo sapiens | SARS-CoV-2 | | 2020-09-07 | Europe | England | Europe | England | Oropharyngeal swab | genome | 29822 | Human | B.1.218 | |
| **H68-ENG** | Homo sapiens | SARS-CoV-2 | | 2020-12-17 | Europe | England | Europe | England | Oropharyngeal swab | genome | 29822 | Human | B.1.408 | |
| **H69-LET** | Homo sapiens | SARS-CoV-2 | EPI_ISL_1590991 | 2021-03-16 | Europe | Latvia | Europe | Latvia | Nasal swab | genome | 29822 | Human | B.1.177.79 | GV |
| **H70-ENG** | Homo sapiens | SARS-CoV-2 | | 2020-11-12 | Europe | England | Europe | England | Oropharyngeal swab | genome | 29822 | Human | B.1.258.3 | |
| **H71-SPN** | Homo sapiens | SARS-CoV-2 | | 2021-02-07 | Europe | Spain | Europe | Spain | Oronasopharynx | genome | 29822 | Human | B.1.177 | |
| **H72-LET** | Homo sapiens | SARS-CoV-2 | EPI_ISL_1590874 | 2021-03-10 | Europe | Latvia | Europe | Latvia | Nasal swab | genome | 29822 | Human | U.3¬† | GV |
| **H73-COL** | Homo sapiens | SARS-CoV-2 | EPI_ISL_1235686 | 2021-01-26 | South America | Colombia | South America | Colombia | Nasopharyngeal swab | genome | 29822 | Human | B.1.111 | GH |
| **H74-ENG** | Homo sapiens | SARS-CoV-2 | | 2020-11-02 | Europe | England | Europe | England | Oropharyngeal swab | genome | 29822 | Human | B.1 | |
| **H75-ENG** | Homo sapiens | SARS-CoV-2 | | 2020-09-25 | Europe | England | Europe | England | Oropharyngeal swab | genome | 29822 | Human | AD.2¬† | |
| **H76-MYA** | Homo sapiens | SARS-CoV-2 | EPI_ISL_1424065 | 2021-01-11 | Asia | Malaysia | Asia | Malaysia | Left lung | genome | 29822 | Human | B.1.524¬† | G |
| **H77-ENG** | Homo sapiens | SARS-CoV-2 | | 2020-11-01 | Europe | England | Europe | England | Oropharyngeal swab | genome | 29822 | Human | B.1.1.303 | |
| **H78-ENG** | Homo sapiens | SARS-CoV-2 | | 2020-11-12 | Europe | England | Europe | England | Oropharyngeal swab | genome | 29822 | Human | B.1.1.170 | |
| **H79-ENG** | Homo sapiens | SARS-CoV-2 | | 2020-11-12 | Europe | England | Europe | England | Oropharyngeal swab | genome | 29822 | Human | B.1.177 | |
| **H80-TUS** | Homo sapiens | SARS-CoV-2 | EPI_ISL_699657 | 2020-03-16 | Africa | Tunisia | Africa | Tunisia | Nasopharyngeal swab | genome | 29822 | Human | B.1.177 | GV |
| **H81-EGP** | Homo sapiens | SARS-CoV-2 | EPI_ISL_1141525 | 2020-06-08 | Africa | Egypt | Africa | Egypt | Oropharyngeal swab | genome | 29822 | Human | C.36 | GR |
| **H82-ENG** | Homo sapiens | SARS-CoV-2 | | 2020-12-18 | Europe | England | Europe | England | Oropharyngeal swab | genome | 29822 | Human | B.1.568 | |
| **H83-ENG** | Homo sapiens | SARS-CoV-2 | | 2020-09-19 | Europe | England | Europe | England | Oropharyngeal swab | genome | 29822 | Human | B.1.2 | |
| **H84-LET** | Homo sapiens | SARS-CoV-2 | EPI_ISL_1590957 | 2021-03-16 | Europe | Latvia | Europe | Latvia | Nasal swab | genome | 29822 | Human | B.1.1.67 | GR |
| **H85-CZH** | Homo sapiens | SARS-CoV-2 | EPI_ISL_1588539 | 2021-03-15 | Europe | Czech Repub | Europe | Czech Republic | Nasopharyngeal swab | genome | 29822 | Human | B.1.258 | G |
| **H86-LET** | Homo sapiens | SARS-CoV-2 | EPI_ISL_1590463 | 2021-03-09 | Europe | Latvia | Europe | Latvia | Nasal swab | genome | 29822 | Human | B.1.1.141 | GR |
| **H87-PER** | Homo sapiens | SARS-CoV-2 | EPI_ISL_593774 | 2020-04-07 | South America | Peru | South America | Peru | Nasopharyngeal swab | genome | 29822 | Human | C.14 | GR |
| **H88-EGP** | Homo sapiens | SARS-CoV-2 | EPI_ISL_1109627 | 2020-06-13 | Africa | Egypt | Africa | Egypt | Oropharyngeal swab | genome | 29822 | Human | B.1¬† | GH |
| **H89-CLE** | Homo sapiens | SARS-CoV-2 | EPI_ISL_445359 | 2020-03-16 | South America | Chile | South America | Chile | Throat swab | genome | 29822 | Human | B.1.1¬† | GR |
| **H90-JPN** | Homo sapiens | SARS-CoV-2 | | 2020-06-01 | Asia | Japan | Asia | Japan | Oronasopharynx | genome | 29822 | Human | B.1.1.283 | |
| **H91-CLE** | Homo sapiens | SARS-CoV-2 | EPI_ISL_445362 | 2020-03-17 | South America | Chile | South America | Chile | Throat swab | genome | 29822 | Human | B.1.1.33¬† | GR |
| **H92-CLE** | Homo sapiens | SARS-CoV-2 | EPI_ISL_445370 | 2020-04-05 | South America | Chile | South America | Chile | Throat swab | genome | 29822 | Human | B.1.1.33 | GR |
| **H93-CHA** | Homo sapiens | SARS-CoV-2 | | 2020-01-22 | Asia | China | Asia | China | Lung, Oronasopharynx | genome | 29822 | Human | B | |
| **H94-CHA** | Homo sapiens | SARS-CoV-2 | | 2020-01-03 | Asia | China | Asia | China | Lung, Oronasopharynx | genome | 29822 | Human | B | |
| **H95-MRO** | Homo sapiens | SARS-CoV-2 | EPI_ISL_451400 | 2020-04-23 | Asia | China | Asia | China | Nasopharyngeal swab | genome | 29822 | Human | B.1.528 | G |
| **H96-COL** | Homo sapiens | SARS-CoV-2 | EPI_ISL_445219 | 2020-04-02 | Asia | China | Asia | China | Nasopharyngeal swab | genome | 29822 | Human | B.1 | GH |
| **H97-CLE** | Homo sapiens | SARS-CoV-2 | EPI_ISL_445295 | 2020-03-21 | Asia | China | Asia | China | Throat swab | genome | 29822 | Human | B.1¬† | G |
| **H98-COL** | Homo sapiens | SARS-CoV-2 | EPI_ISL_456153 | 2020-04-22 | Asia | China | Asia | China | Nasopharyngeal swab | genome | 29822 | Human | B.1¬† | GH |

Material_S2_SARS-RNAz

| Host | ORF | Locus_ID | Strand | Start | End | P | Type |
|---|---|---|---|---|---|---|---|
| **Human** | 5UTR | locus1 | Forward | 1 | 160 | 0.995962 | Unique |
| **Bat** | 5UTR | locus3 | Forward | 41 | 160 | 0.996679 | Unique |
| **Human** | ORF1a | locus5 | Forward | 161 | 280 | 0.997249 | Shared |
| **Bat** | ORF1a | locus7 | Forward | 161 | 280 | 0.997273 | Shared |
| **Intermediate** | ORF1a | locus5 | Forward | 681 | 800 | 0.995714 | Unique |
| **Human** | ORF1a | locus8 | Forward | 721 | 1040 | 0.998574 | Shared |
| **Bat** | ORF1a | locus9 | Forward | 721 | 1040 | 0.998187 | Shared |
| **Intermediate** | ORF1a | locus7 | Forward | 881 | 1000 | 0.997827 | Unique |
| **Intermediate** | ORF1a | locus9 | Forward | 1041 | 1200 | 0.998238 | Unique |
| **Intermediate** | ORF1a | locus18 | Forward | 1921 | 2160 | 0.996397 | Unique |
| **Bat** | ORF1a | locus15 | Forward | 2121 | 2640 | 0.997865 | Unique |
| **Intermediate** | ORF1a | locus19 | Forward | 2281 | 2400 | 0.99832 | Unique |
| **Human** | ORF1a | locus17 | Forward | 2481 | 2640 | 0.998672 | Unique |
| **Bat** | ORF1a | locus16 | Forward | 2681 | 2880 | 0.998776 | Unique |
| **Human** | ORF1a | locus21 | Forward | 2721 | 2880 | 0.995648 | Unique |
| **Intermediate** | ORF1a | locus26 | Forward | 2761 | 3400 | 0.998638 | Unique |
| **Bat** | ORF1a | locus20 | Forward | 2881 | 3040 | 0.997901 | Unique |
| **Human** | ORF1a | locus23 | Forward | 2881 | 3280 | 0.998598 | Unique |
| **Bat** | ORF1a | locus22 | Forward | 2961 | 3080 | 0.99586 | Unique |
| **Bat** | ORF1a | locus26 | Forward | 3201 | 3320 | 0.998272 | Unique |
| **Intermediate** | ORF1a | locus27 | Forward | 3601 | 4040 | 0.99602 | Unique |
| **Bat** | ORF1a | locus28 | Forward | 3681 | 3840 | 0.996087 | Unique |
| **Bat** | ORF1a | locus32 | Forward | 3801 | 4160 | 0.998684 | Unique |
| **Human** | ORF1a | locus28 | Forward | 4001 | 4160 | 0.996808 | Unique |
| **Intermediate** | ORF1a | locus33 | Forward | 4121 | 4520 | 0.997281 | Unique |
| **Bat** | ORF1a | locus34 | Forward | 4161 | 4280 | 0.997831 | Unique |
| **Human** | ORF1a | locus30 | Forward | 4161 | 4400 | 0.996347 | Unique |
| **Human** | ORF1a | locus32 | Forward | 4361 | 4480 | 0.99584 | Unique |
| **Intermediate** | ORF1a | locus35 | Forward | 4441 | 5040 | 0.996635 | Unique |

| | | | | | | | |
|---|---|---|---|---|---|---|---|
| **Bat** | ORF1a | locus38 | Forward | 4481 | 4680 | 0.997886 | Unique |
| **Intermediate** | ORF1a | locus37 | Forward | 4961 | 5080 | 0.996133 | Unique |
| **Human** | ORF1a | locus41 | Forward | 5001 | 5120 | 0.996077 | Unique |
| **Human** | ORF1a | locus43 | Forward | 5041 | 5160 | 0.998508 | Common |
| **Intermediate** | ORF1a | locus41 | Forward | 5041 | 5160 | 0.995901 | Common |
| **Bat** | ORF1a | locus41 | Forward | 5041 | 5160 | 0.996496 | Common |
| **Intermediate** | ORF1a | locus43 | Forward | 5081 | 5200 | 0.995648 | Unique |
| **Intermediate** | ORF1a | locus45 | Forward | 5121 | 5240 | 0.997907 | Unique |
| **Human** | ORF1a | locus47 | Forward | 5121 | 5320 | 0.997646 | Shared |
| **Bat** | ORF1a | locus45 | Forward | 5121 | 5320 | 0.998331 | Shared |
| **Intermediate** | ORF1a | locus47 | Forward | 5161 | 5280 | 0.997848 | Unique |
| **Intermediate** | ORF1a | locus49 | Forward | 5201 | 5320 | 0.998351 | Unique |
| **Bat** | ORF1a | locus51 | Forward | 5641 | 5760 | 0.998148 | Unique |
| **Intermediate** | ORF1a | locus52 | Forward | 5961 | 6240 | 0.99813 | Unique |
| **Human** | ORF1a | locus54 | Forward | 6001 | 6280 | 0.998638 | Shared |
| **Bat** | ORF1a | locus53 | Forward | 6001 | 6280 | 0.998728 | Shared |
| **Human** | ORF1a | locus60 | Forward | 6921 | 7040 | 0.998618 | Unique |
| **Human** | ORF1a | locus62 | Forward | 6961 | 7080 | 0.995901 | Unique |
| **Bat** | ORF1a | locus58 | Forward | 7361 | 7640 | 0.99532 | Unique |
| **Human** | ORF1a | locus67 | Forward | 7721 | 7840 | 0.995714 | Unique |
| **Intermediate** | ORF1a | locus72 | Forward | 8561 | 8800 | 0.995648 | Unique |
| **Human** | ORF1a | locus76 | Forward | 8601 | 8880 | 0.998238 | Shared |
| **Bat** | ORF1a | locus66 | Forward | 8601 | 8880 | 0.998098 | Shared |
| **Human** | ORF1a | locus78 | Forward | 9121 | 9280 | 0.996133 | Unique |
| **Intermediate** | ORF1a | locus78 | Forward | 10801 | 10920 | 0.99805 | Unique |
| **Intermediate** | ORF1a | locus80 | Forward | 10841 | 11040 | 0.996133 | Unique |
| **Human** | ORF1a | locus87 | Forward | 10881 | 11080 | 0.998128 | Unique |
| **Bat** | ORF1a | locus76 | Forward | 10881 | 11120 | 0.998682 | Unique |
| **Intermediate** | ORF1a | locus82 | Forward | 10961 | 11080 | 0.998608 | Unique |
| **Human** | ORF1a | locus89 | Forward | 11001 | 11120 | 0.998637 | Unique |
| **Bat** | ORF1a | locus81 | Forward | 11641 | 11760 | 0.996563 | Unique |
| **Human** | ORF1a | locus95 | Forward | 11641 | 11840 | 0.995777 | Unique |

| | | | | | | | |
|---|---|---|---|---|---|---|---|
| **Intermediate** | ORF1a | locus90 | Forward | 13241 | 13360 | 0.998647 | Unique |
| **Intermediate** | ORF1a | locus92 | Forward | 13281 | 13400 | 0.996397 | Unique |
| **Human** | NA | locus98 | Forward | 13281 | 13440 | 0.997911 | Unique |
| **Human** | ORF1b | locus101 | Forward | 13641 | 14200 | 0.996849 | Shared |
| **Bat** | ORF1b | locus85 | Forward | 13641 | 14200 | 0.996933 | Shared |
| **Intermediate** | ORF1b | locus95 | Forward | 13681 | 14040 | 0.996188 | Unique |
| **Intermediate** | ORF1b | locus96 | Forward | 14081 | 14200 | 0.997827 | Unique |
| **Human** | ORF1b | locus103 | Forward | 14361 | 14600 | 0.997249 | Shared |
| **Bat** | ORF1b | locus87 | Forward | 14361 | 14600 | 0.996838 | Shared |
| **Human** | ORF1b | locus106 | Forward | 14801 | 14920 | 0.997183 | Unique |
| **Intermediate** | ORF1b | locus106 | Forward | 16321 | 16440 | 0.998113 | Unique |
| **Intermediate** | ORF1b | locus110 | Forward | 17001 | 17200 | 0.998185 | Unique |
| **Bat** | ORF1b | locus94 | Forward | 17041 | 17160 | 0.998455 | Unique |
| **Human** | ORF1b | locus115 | Forward | 17121 | 17240 | 0.996589 | Unique |
| **Human** | ORF1b | locus119 | Forward | 17441 | 17560 | 0.996397 | Shared |
| **Bat** | ORF1b | locus97 | Forward | 17441 | 17560 | 0.996365 | Shared |
| **Bat** | ORF1b | locus104 | Forward | 19361 | 19480 | 0.997921 | Unique |
| **Human** | ORF1b | locus131 | Forward | 19841 | 19960 | 0.996242 | Unique |
| **Intermediate** | ORF1b | locus130 | Forward | 19841 | 20000 | 0.997827 | Unique |
| **Human** | ORF1b | locus133 | Forward | 19881 | 20000 | 0.998532 | Unique |
| **Intermediate** | ORF1b | locus134 | Forward | 20161 | 20280 | 0.996495 | Unique |
| **Human** | ORF1b | locus137 | Forward | 20201 | 20320 | 0.997827 | Unique |
| **Human** | ORF1b | locus139 | Forward | 20441 | 20560 | 0.996542 | Unique |
| **Bat** | ORF1b | locus111 | Forward | 20721 | 20880 | 0.998866 | Unique |
| **Human** | ORF1b | locus142 | Forward | 20761 | 20880 | 0.998506 | Unique |
| **Intermediate** | ORF1b | locus146 | Forward | 21281 | 21400 | 0.996077 | Unique |
| **Human** | ORF1b | locus149 | Forward | 21321 | 21440 | 0.997313 | Shared |
| **Intermediate** | ORF1b | locus148 | Forward | 21321 | 21440 | 0.995648 | Shared |
| **Intermediate** | S | locus150 | Forward | 21841 | 22080 | 0.997249 | Unique |
| **Intermediate** | S | locus152 | Forward | 22001 | 22120 | 0.998641 | Unique |
| **Human** | S | locus159 | Forward | 22001 | 22160 | 0.996635 | Unique |
| **Intermediate** | S | locus154 | Forward | 22201 | 22320 | 0.996575 | Unique |

| | | | | | | | |
|---|---|---|---|---|---|---|---|
| **Bat** | S | locus116 | Forward | 22401 | 22520 | 0.993264 | Unique |
| **Intermediate** | S | locus159 | Forward | 22561 | 22760 | 0.996133 | Unique |
| **Human** | S | locus165 | Forward | 23001 | 23120 | 0.998645 | Unique |
| **Intermediate** | S | locus165 | Forward | 23321 | 23480 | 0.998591 | Unique |
| **Bat** | S | locus119 | Forward | 23321 | 23560 | 0.997862 | Unique |
| **Human** | S | locus169 | Forward | 23361 | 23560 | 0.996876 | Unique |
| **Intermediate** | S | locus169 | Forward | 23521 | 23640 | 0.997694 | Unique |
| **Human** | S | locus171 | Forward | 23561 | 23680 | 0.998171 | Unique |
| **Human** | S | locus173 | Forward | 23601 | 23720 | 0.998185 | Unique |
| **Intermediate** | S | locus173 | Forward | 23801 | 23920 | 0.998199 | Unique |
| **Human** | S | locus176 | Forward | 24121 | 24320 | 0.997183 | Unique |
| **Intermediate** | S | locus176 | Forward | 24841 | 24960 | 0.996295 | Unique |
| **Intermediate** | ORF3a | locus177 | Forward | 25281 | 25600 | 0.998033 | Unique |
| **Human** | ORF3a | locus181 | Forward | 25361 | 25480 | 0.99839 | Unique |
| **Human** | ORF3a | locus183 | Forward | 25441 | 25640 | 0.996271 | Unique |
| **Human** | ORF3a | locus185 | Forward | 25561 | 25680 | 0.997514 | Unique |
| **Bat** | ORF3a | locus127 | Forward | 25681 | 25960 | 0.997383 | Unique |
| **Human** | ORF3a | locus187 | Forward | 25681 | 26000 | 0.99757 | Shared |
| **Intermediate** | ORF3a | locus179 | Forward | 25681 | 26000 | 0.997489 | Shared |
| **Bat** | ORF3a | locus129 | Forward | 25881 | 26000 | 0.996612 | Unique |
| **Intermediate** | ORF3a | locus181 | Forward | 25921 | 26040 | 0.997557 | Shared |
| **Bat** | ORF3a | locus131 | Forward | 25921 | 26040 | 0.998429 | Shared |
| **Human** | ORF3a | locus191 | Forward | 25961 | 26080 | 0.99801 | Common |
| **Intermediate** | ORF3a | locus183 | Forward | 25961 | 26080 | 0.998487 | Common |
| **Bat** | ORF3a | locus133 | Forward | 25961 | 26080 | 0.99842 | Common |
| **Human** | ORF3b | locus193 | Forward | 26001 | 26120 | 0.998159 | Common |
| **Intermediate** | ORF3b | locus185 | Forward | 26001 | 26120 | 0.9984 | Common |
| **Bat** | ORF3b | locus135 | Forward | 26001 | 26120 | 0.998491 | Common |
| **Bat** | E | locus137 | Forward | 26041 | 26160 | 0.998685 | Unique |
| **Human** | E | locus195 | Forward | 26041 | 26200 | 0.998746 | Unique |
| **Intermediate** | E | locus187 | Forward | 26041 | 26320 | 0.99832 | Unique |
| **Human** | M | locus197 | Forward | 26121 | 26440 | 0.994943 | Unique |

| | | | | | | | |
|---|---|---|---|---|---|---|---|
| **Human** | M | locus199 | Forward | 26361 | 26480 | 0.994142 | Unique |
| **Bat** | ORF8b | locus150 | Forward | 27721 | 28000 | 0.998884 | Unique |
| **Intermediate** | N | locus199 | Forward | 28681 | 29000 | 0.998495 | Unique |
| **Bat** | N | locus155 | Forward | 28881 | 29000 | 0.997047 | Unique |
| **Bat** | N | locus157 | Forward | 28921 | 29080 | 0.994764 | Unique |
| **Intermediate** | N | locus200 | Forward | 29081 | 29200 | 0.995582 | Unique |

Material_S2_MERS-RNAz

| Host | ORF | Locus_ID | Strand | Start | End | P | Type |
|---|---|---|---|---|---|---|---|
| **Human** | 5UTR | locus2 | Forward | 81 | 200 | 0.99815 | Shared |
| **Intermediate** | 5UTR | locus2 | Forward | 81 | 200 | 0.997948 | Shared |
| **Human** | ORF1a | locus4 | Forward | 121 | 280 | 0.998428 | Shared |
| **Intermediate** | ORF1a | locus4 | Forward | 121 | 280 | 0.997858 | Shared |
| **Bat** | ORF1a | locus2 | Forward | 521 | 920 | 0.998757 | Unique |
| **Human** | ORF1a | locus9 | Forward | 721 | 960 | 0.998671 | Shared |
| **Intermediate** | ORF1a | locus9 | Forward | 721 | 960 | 0.99865 | Shared |
| **Human** | ORF1a | locus11 | Forward | 881 | 1000 | 0.998151 | Shared |
| **Intermediate** | ORF1a | locus11 | Forward | 881 | 1000 | 0.998157 | Shared |
| **Human** | ORF1a | locus13 | Forward | 921 | 1040 | 0.998639 | Shared |
| **Intermediate** | ORF1a | locus13 | Forward | 921 | 1040 | 0.998648 | Shared |
| **Human** | ORF1a | locus15 | Forward | 961 | 1080 | 0.998618 | Shared |
| **Intermediate** | ORF1a | locus15 | Forward | 961 | 1080 | 0.998628 | Shared |
| **Bat** | ORF1a | locus6 | Forward | 961 | 1200 | 0.996946 | Unique |
| **Human** | ORF1a | locus23 | Forward | 1161 | 1280 | 0.995978 | Unique |
| **Intermediate** | ORF1a | locus19 | Forward | 1161 | 1360 | 0.996092 | Unique |
| **Human** | ORF1a | locus25 | Forward | 1201 | 1360 | 0.996219 | Unique |
| **Bat** | ORF1a | locus10 | Forward | 1481 | 1640 | 0.998754 | Unique |
| **Bat** | ORF1a | locus12 | Forward | 1601 | 1840 | 0.998123 | Unique |
| **Bat** | ORF1a | locus14 | Forward | 1921 | 2040 | 0.996814 | Unique |
| **Human** | ORF1a | locus27 | Forward | 1961 | 2080 | 0.996565 | Shared |
| **Intermediate** | ORF1a | locus21 | Forward | 1961 | 2080 | 0.996655 | Shared |
| **Bat** | ORF1a | locus17 | Forward | 2121 | 2320 | 0.998564 | Unique |
| **Human** | ORF1a | locus28 | Forward | 2201 | 2320 | 0.997763 | Shared |
| **Intermediate** | ORF1a | locus22 | Forward | 2201 | 2320 | 0.997671 | Shared |
| **Human** | ORF1a | locus32 | Forward | 2361 | 2480 | 0.99601 | Shared |
| **Intermediate** | ORF1a | locus26 | Forward | 2361 | 2480 | 0.996467 | Shared |
| **Bat** | ORF1a | locus18 | Forward | 2361 | 2520 | 0.990368 | Unique |
| **Human** | ORF1a | locus37 | Forward | 2601 | 2800 | 0.997067 | Shared |

| | | | | | | | |
|---|---|---|---|---|---|---|---|
| **Intermediate** | ORF1a | locus31 | Forward | 2601 | 2800 | 0.981289 | Shared |
| **Bat** | ORF1a | locus26 | Forward | 3161 | 3280 | 0.998143 | Unique |
| **Bat** | ORF1a | locus28 | Forward | 3201 | 3440 | 0.99839 | Unique |
| **Bat** | ORF1a | locus30 | Forward | 3361 | 3480 | 0.996447 | Unique |
| **Bat** | ORF1a | locus32 | Forward | 3481 | 3760 | 0.998417 | Unique |
| **Human** | ORF1a | locus46 | Forward | 3561 | 3680 | 0.998221 | Shared |
| **Intermediate** | ORF1a | locus40 | Forward | 3561 | 3680 | 0.998532 | Shared |
| **Human** | ORF1a | locus51 | Forward | 3801 | 3920 | 0.995697 | Shared |
| **Intermediate** | ORF1a | locus45 | Forward | 3801 | 3920 | 0.996233 | Shared |
| **Bat** | ORF1a | locus38 | Forward | 3881 | 4240 | 0.998017 | Unique |
| **Human** | ORF1a | locus56 | Forward | 4281 | 4400 | 0.998188 | Shared |
| **Intermediate** | ORF1a | locus50 | Forward | 4281 | 4400 | 0.997926 | Shared |
| **Human** | ORF1a | locus58 | Forward | 4321 | 4480 | 0.997129 | Shared |
| **Intermediate** | ORF1a | locus52 | Forward | 4321 | 4480 | 0.997367 | Shared |
| **Bat** | ORF1a | locus42 | Forward | 4401 | 4560 | 0.997676 | Unique |
| **Human** | ORF1a | locus64 | Forward | 4681 | 4800 | 0.99757 | Shared |
| **Intermediate** | ORF1a | locus57 | Forward | 4681 | 4800 | 0.997425 | Shared |
| **Bat** | ORF1a | locus47 | Forward | 4841 | 5040 | 0.998157 | Unique |
| **Bat** | ORF1a | locus48 | Forward | 5121 | 5240 | 0.998508 | Unique |
| **Human** | ORF1a | locus70 | Forward | 5721 | 5840 | 0.998478 | Shared |
| **Intermediate** | ORF1a | locus63 | Forward | 5721 | 5840 | 0.998442 | Shared |
| **Bat** | ORF1a | locus57 | Forward | 5881 | 6040 | 0.996188 | Unique |
| **Human** | ORF1a | locus76 | Forward | 6081 | 6200 | 0.997347 | Shared |
| **Intermediate** | ORF1a | locus69 | Forward | 6081 | 6200 | 0.996601 | Shared |
| **Human** | ORF1a | locus78 | Forward | 6161 | 6320 | 0.997607 | Shared |
| **Intermediate** | ORF1a | locus71 | Forward | 6161 | 6320 | 0.993046 | Shared |
| **Bat** | ORF1a | locus65 | Forward | 6201 | 6320 | 0.997982 | Unique |
| **Human** | ORF1a | locus80 | Forward | 6241 | 6400 | 0.998671 | Shared |
| **Intermediate** | ORF1a | locus73 | Forward | 6241 | 6400 | 0.998299 | Shared |
| **Human** | ORF1a | locus82 | Forward | 6321 | 6440 | 0.998133 | Shared |
| **Intermediate** | ORF1a | locus75 | Forward | 6321 | 6440 | 0.998268 | Shared |
| **Bat** | ORF1a | locus80 | Forward | 7801 | 7920 | 0.997941 | Unique |

| | | | | | | | |
|---|---|---|---|---|---|---|---|
| **Human** | ORF1a | locus88 | Forward | 7961 | 8200 | 0.997408 | Shared |
| **Intermediate** | ORF1a | locus81 | Forward | 7961 | 8200 | 0.996242 | Shared |
| **Human** | ORF1a | locus95 | Forward | 9801 | 9920 | 0.996783 | Shared |
| **Intermediate** | ORF1a | locus88 | Forward | 9801 | 9920 | 0.9968360 | Shared |
| **Bat** | ORF1a | locus99 | Forward | 11561 | 11680 | 0.997114 | Unique |
| **Bat** | ORF1a | locus101 | Forward | 11881 | 12040 | 0.998701 | Unique |
| **Bat** | NA | locus105 | Forward | 13241 | 13480 | 0.997111 | Unique |
| **Human** | NA | locus117 | Forward | 13401 | 13520 | 0.998225 | Common |
| **Intermediate** | NA | locus111 | Forward | 13401 | 13520 | 0.998239 | Common |
| **Bat** | NA | locus107 | Forward | 13401 | 13520 | 0.997114 | Common |
| **Bat** | ORF1b | locus113 | Forward | 13521 | 13800 | 0.99774 | Unique |
| **Human** | ORF1b | locus131 | Forward | 14161 | 14320 | 0.996751 | Unique |
| **Intermediate** | ORF1b | locus126 | Forward | 14201 | 14320 | 0.996849 | Unique |
| **Human** | ORF1b | locus137 | Forward | 14361 | 14480 | 0.998371 | Shared |
| **Intermediate** | ORF1b | locus132 | Forward | 14361 | 14480 | 0.9984 | Shared |
| **Human** | ORF1b | locus139 | Forward | 14841 | 14960 | 0.997179 | Shared |
| **Intermediate** | ORF1b | locus134 | Forward | 14841 | 14960 | 0.997183 | Shared |
| **Bat** | ORF1b | locus126 | Forward | 15241 | 15440 | 0.998572 | Unique |
| **Human** | ORF1b | locus143 | Forward | 15321 | 15440 | 0.998157 | Shared |
| **Intermediate** | ORF1b | locus138 | Forward | 15321 | 15440 | 0.998033 | Shared |
| **Human** | ORF1b | locus145 | Forward | 15361 | 15480 | 0.998534 | Shared |
| **Intermediate** | ORF1b | locus140 | Forward | 15361 | 15480 | 0.998537 | Shared |
| **Bat** | ORF1b | locus127 | Forward | 15481 | 15720 | 0.998521 | Unique |
| **Human** | ORF1b | locus148 | Forward | 16001 | 16160 | 0.998426 | Shared |
| **Intermediate** | ORF1b | locus143 | Forward | 16001 | 16160 | 0.998426 | Shared |
| **Human** | ORF1b | locus155 | Forward | 16441 | 16560 | 0.997543 | Shared |
| **Intermediate** | ORF1b | locus150 | Forward | 16441 | 16560 | 0.997621 | Shared |
| **Human** | ORF1b | locus161 | Forward | 17081 | 17200 | 0.997784 | Shared |
| **Intermediate** | ORF1b | locus155 | Forward | 17081 | 17200 | 0.998645 | Shared |
| **Bat** | ORF1b | locus138 | Forward | 17201 | 17320 | 0.99791 | Unique |
| **Bat** | ORF1b | locus140 | Forward | 17241 | 17480 | 0.997024 | Unique |
| **Human** | ORF1b | locus164 | Forward | 17281 | 17400 | 0.996889 | Shared |

| | | | | | | | |
|---|---|---|---|---|---|---|---|
| **Intermediate** | ORF1b | locus158 | Forward | 17281 | 17400 | 0.996889 | Shared |
| **Bat** | ORF1b | locus145 | Forward | 17961 | 18080 | 0.998515 | Unique |
| **Bat** | ORF1b | locus151 | Forward | 18841 | 18960 | 0.998613 | Unique |
| **Bat** | ORF1b | locus153 | Forward | 19121 | 19360 | 0.998507 | Unique |
| **Human** | ORF1b | locus183 | Forward | 19281 | 19440 | 0.997941 | Shared |
| **Intermediate** | ORF1b | locus177 | Forward | 19281 | 19440 | 0.997942 | Shared |
| **Bat** | ORF1b | locus157 | Forward | 19361 | 19480 | 0.996823 | Unique |
| **Bat** | ORF1b | locus160 | Forward | 19721 | 19840 | 0.997413 | Unique |
| **Human** | ORF1b | locus197 | Forward | 20241 | 20360 | 0.99783 | Shared |
| **Intermediate** | ORF1b | locus190 | Forward | 20241 | 20360 | 0.997881 | Shared |
| **Bat** | ORF1b | locus171 | Forward | 20601 | 20720 | 0.995648 | Unique |
| **Human** | ORF1b | locus203 | Forward | 20841 | 21000 | 0.998666 | Shared |
| **Intermediate** | ORF1b | locus196 | Forward | 20841 | 21000 | 0.998568 | Shared |
| **Bat** | ORF1b | locus175 | Forward | 20841 | 21120 | 0.996077 | Unique |
| **Human** | S | locus209 | Forward | 21201 | 21520 | 0.998646 | Shared |
| **Intermediate** | S | locus202 | Forward | 21201 | 21520 | 0.998644 | Shared |
| **Bat** | ORF1b | locus177 | Forward | 21241 | 21400 | 0.998587 | Unique |
| **Bat** | S | locus179 | Forward | 21881 | 22000 | 0.997543 | Unique |
| **Human** | S | locus219 | Forward | 22001 | 22160 | 0.997902 | Shared |
| **Intermediate** | S | locus210 | Forward | 22001 | 22160 | 0.998044 | Shared |
| **Human** | S | locus223 | Forward | 22161 | 22280 | 0.996953 | Shared |
| **Intermediate** | S | locus214 | Forward | 22161 | 22280 | 0.996701 | Shared |
| **Bat** | S | locus180 | Forward | 22281 | 22400 | 0.995734 | Unique |
| **Bat** | S | locus186 | Forward | 22561 | 22680 | 0.998549 | Unique |
| **Intermediate** | S | locus220 | Forward | 22641 | 22840 | 0.99848 | Unique |
| **Human** | S | locus232 | Forward | 22721 | 22840 | 0.998706 | Unique |
| **Human** | S | locus234 | Forward | 22761 | 22880 | 0.997305 | Shared |
| **Intermediate** | S | locus222 | Forward | 22761 | 22880 | 0.998001 | Shared |
| **Bat** | S | locus190 | Forward | 22801 | 22920 | 0.997582 | Unique |
| **Bat** | S | locus194 | Forward | 23161 | 23320 | 0.998212 | Unique |
| **Bat** | S | locus196 | Forward | 23241 | 23360 | 0.996123 | Unique |
| **Human** | S | locus238 | Forward | 23401 | 23520 | 0.996942 | Shared |

| | | | | | | | |
|---|---|---|---|---|---|---|---|
| **Intermediate** | S | locus225 | Forward | 23401 | 23520 | 0.984869 | Shared |
| **Intermediate** | S | locus233 | Forward | 24561 | 24800 | 0.99642 | Unique |
| **Bat** | S | locus204 | Forward | 24721 | 24880 | 0.995961 | Unique |
| **Bat** | S | locus208 | Forward | 25001 | 25240 | 0.997945 | Unique |
| **Bat** | ORF4a | locus219 | Forward | 26041 | 26160 | 0.99562 | Unique |
| **Intermediate** | ORF4b | locus239 | Forward | 26161 | 26320 | 0.995688 | Unique |
| **Intermediate** | ORF4b | locus241 | Forward | 26321 | 26480 | 0.998648 | Unique |
| **Human** | ORF4b | locus253 | Forward | 26361 | 26480 | 0.998654 | Unique |
| **Bat** | ORF5 | locus226 | Forward | 26601 | 26920 | 0.997552 | Unique |
| **Bat** | ORF5 | locus228 | Forward | 26841 | 26960 | 0.997926 | Unique |
| **Human** | ORF5 | locus259 | Forward | 26841 | 27200 | 0.998486 | Shared |
| **Intermediate** | ORF5 | locus247 | Forward | 26841 | 27200 | 0.998303 | Shared |
| **Bat** | ORF5 | locus230 | Forward | 26881 | 27000 | 0.998605 | Unique |
| **Bat** | ORF5 | locus232 | Forward | 26921 | 27040 | 0.998066 | Unique |
| **Bat** | ORF5 | locus234 | Forward | 26961 | 27080 | 0.998508 | Unique |
| **Bat** | ORF5 | locus236 | Forward | 27001 | 27160 | 0.997909 | Unique |
| **Bat** | ORF5 | locus238 | Forward | 27081 | 27200 | 0.997983 | Unique |
| **Bat** | ORF5 | locus242 | Forward | 27161 | 27400 | 0.99678 | Unique |
| **Bat** | ORF5 | locus244 | Forward | 27361 | 27480 | 0.998263 | Unique |
| **Human** | E | locus266 | Forward | 27441 | 27600 | 0.99807 | Shared |
| **Intermediate** | E | locus254 | Forward | 27441 | 27600 | 0.998033 | Shared |
| **Human** | E | locus268 | Forward | 27521 | 27640 | 0.995714 | Shared |
| **Intermediate** | E | locus256 | Forward | 27521 | 27640 | 0.995753 | Shared |
| **Bat** | M | locus249 | Forward | 27761 | 27880 | 0.995628 | Unique |
| **Bat** | M | locus251 | Forward | 27801 | 27920 | 0.996953 | Unique |
| **Human** | NA | locus279 | Forward | 28281 | 28520 | 0.995631 | Shared |
| **Intermediate** | NA | locus266 | Forward | 28281 | 28520 | 0.995648 | Shared |
| **Human** | N | locus281 | Forward | 28441 | 28640 | 0.998236 | Shared |
| **Intermediate** | N | locus268 | Forward | 28441 | 28640 | 0.998263 | Shared |
| **Bat** | N | locus263 | Forward | 29121 | 29320 | 0.99846 | Unique |
| **Bat** | N | locus265 | Forward | 29361 | 29480 | 0.993477 | Unique |
| **Human** | N | locus285 | Forward | 29561 | 29680 | 0.987408 | Shared |

| | | | | | | | |
|---|---|---|---|---|---|---|---|
| **Intermediate** | N | locus274 | Forward | 29561 | 29680 | 0.985906 | Shared |

Material_S2_SARS2-RNAz

| Host | ORF | Locus_ID | Strand | Start | End | P | Type |
|---|---|---|---|---|---|---|---|
| **Bat** | 5UTR | locus1 | Forward | 1 | 120 | 0.99882 | Unique |
| **Human** | 5UTR | locus1 | Forward | 1 | 200 | 0.998042 | Unique |
| **Human** | ORF1a | locus4 | Forward | 281 | 400 | 0.990817 | Unique |
| **Intermediate** | ORF1a | locus3 | Forward | 561 | 960 | 0.997512 | Unique |
| **Human** | ORF1a | locus7 | Forward | 881 | 1000 | 0.998334 | Shared |
| **Intermediate** | ORF1a | locus5 | Forward | 881 | 1000 | 0.998333 | Shared |
| **Human** | ORF1a | locus9 | Forward | 921 | 1040 | 0.995626 | Shared |
| **Bat** | ORF1a | locus4 | Forward | 921 | 1040 | 0.998509 | Shared |
| **Intermediate** | ORF1a | locus10 | Forward | 1561 | 1680 | 0.996908 | Unique |
| **Bat** | ORF1a | locus11 | Forward | 1681 | 1920 | 0.997714 | Unique |
| **Human** | ORF1a | locus18 | Forward | 1921 | 2040 | 0.998514 | Unique |
| **Intermediate** | ORF1a | locus16 | Forward | 2201 | 2360 | 0.996414 | Unique |
| **Human** | ORF1a | locus19 | Forward | 2241 | 2400 | 0.997803 | Unique |
| **Intermediate** | ORF1a | locus19 | Forward | 2481 | 2600 | 0.997443 | Unique |
| **Intermediate** | ORF1a | locus23 | Forward | 2641 | 2840 | 0.996842 | Unique |
| **Human** | ORF1a | locus21 | Forward | 2681 | 2880 | 0.998543 | Unique |
| **Intermediate** | ORF1a | locus25 | Forward | 2761 | 2880 | 0.997208 | Unique |
| **Human** | ORF1a | locus25 | Forward | 2961 | 3080 | 0.997439 | Unique |
| **Intermediate** | ORF1a | locus27 | Forward | 3041 | 3160 | 0.997332 | Unique |
| **Human** | ORF1a | locus27 | Forward | 3041 | 3280 | 0.997875 | Unique |
| **Intermediate** | ORF1a | locus29 | Forward | 3361 | 3720 | 0.99812 | Unique |
| **Human** | ORF1a | locus28 | Forward | 3401 | 3560 | 0.998006 | Unique |
| **Human** | ORF1a | locus30 | Forward | 3481 | 3680 | 0.996693 | Unique |
| **Intermediate** | ORF1a | locus39 | Forward | 4801 | 4920 | 0.998782 | Unique |
| **Intermediate** | ORF1a | locus41 | Forward | 4841 | 5120 | 0.998568 | Unique |
| **Human** | ORF1a | locus42 | Forward | 4881 | 5000 | 0.997451 | Unique |
| **Human** | ORF1a | locus44 | Forward | 4921 | 5120 | 0.998653 | Unique |
| **Intermediate** | ORF1a | locus43 | Forward | 5041 | 5160 | 0.998074 | Unique |
| **Bat** | ORF1a | locus25 | Forward | 5201 | 5320 | 0.995898 | Unique |

| | | | | | | | |
|---|---|---|---|---|---|---|---|
| **Intermediate** | ORF1a | locus47 | Forward | 5281 | 5480 | 0.998738 | Unique |
| **Human** | ORF1a | locus54 | Forward | 5321 | 5480 | 0.998033 | Unique |
| **Human** | ORF1a | locus56 | Forward | 5401 | 5600 | 0.997922 | Shared |
| **Intermediate** | ORF1a | locus49 | Forward | 5401 | 5600 | 0.997942 | Shared |
| **Intermediate** | ORF1a | locus51 | Forward | 5521 | 5640 | 0.996647 | Unique |
| **Human** | ORF1a | locus60 | Forward | 5641 | 5760 | 0.996295 | Unique |
| **Human** | ORF1a | locus62 | Forward | 5681 | 5800 | 0.997338 | Shared |
| **Intermediate** | ORF1a | locus55 | Forward | 5681 | 5800 | 0.998408 | Shared |
| **Bat** | ORF1a | locus29 | Forward | 5721 | 5840 | 0.996842 | Unique |
| **Intermediate** | ORF1a | locus60 | Forward | 6041 | 6160 | 0.996993 | Unique |
| **Bat** | ORF1a | locus30 | Forward | 6081 | 6200 | 0.997544 | Unique |
| **Human** | ORF1a | locus70 | Forward | 6561 | 6680 | 0.997625 | Shared |
| **Intermediate** | ORF1a | locus65 | Forward | 6561 | 6680 | 0.998015 | Shared |
| **Intermediate** | ORF1a | locus67 | Forward | 6601 | 6720 | 0.998735 | Unique |
| **Intermediate** | ORF1a | locus69 | Forward | 6681 | 6800 | 0.997023 | Unique |
| **Human** | ORF1a | locus85 | Forward | 7801 | 8040 | 0.996676 | Unique |
| **Intermediate** | ORF1a | locus80 | Forward | 7881 | 8040 | 0.998678 | Unique |
| **Bat** | ORF1a | locus42 | Forward | 7961 | 8080 | 0.997344 | Unique |
| **Human** | ORF1a | locus87 | Forward | 8761 | 8920 | 0.996016 | Shared |
| **Intermediate** | ORF1a | locus82 | Forward | 8761 | 8920 | 0.998732 | Shared |
| **Bat** | ORF1a | locus45 | Forward | 8801 | 8960 | 0.998735 | Unique |
| **Intermediate** | ORF1a | locus85 | Forward | 9241 | 9360 | 0.997991 | Unique |
| **Human** | ORF1a | locus102 | Forward | 11201 | 11320 | 0.996215 | Unique |
| **Human** | ORF1a | locus104 | Forward | 11521 | 11640 | 0.998154 | Unique |
| **Intermediate** | ORF1a | locus100 | Forward | 12321 | 12560 | 0.995868 | Unique |
| **Human** | ORF1a | locus112 | Forward | 12841 | 13000 | 0.996958 | Unique |
| **Intermediate** | ORF1a | locus104 | Forward | 13321 | 13440 | 0.998171 | Unique |
| **Human** | NA | locus113 | Forward | 13321 | 13480 | 0.996529 | Unique |
| **Bat** | NA | locus67 | Forward | 13361 | 13480 | 0.997506 | Unique |
| **Human** | NA | locus115 | Forward | 13561 | 13680 | 0.998005 | Unique |
| **Intermediate** | ORF1b | locus107 | Forward | 13561 | 13760 | 0.995822 | Unique |
| **Human** | ORF1b | locus121 | Forward | 13721 | 13840 | 0.998623 | Shared |

| | | | | | | | | |
|---|---|---|---|---|---|---|---|---|
| **Bat** | ORF1b | locus72 | Forward | 13721 | 13840 | 0.99815 | Shared |
| **Human** | ORF1b | locus127 | Forward | 14521 | 14640 | 0.99744 | Unique |
| **Human** | ORF1b | locus128 | Forward | 14681 | 14840 | 0.996292 | Shared |
| **Intermediate** | ORF1b | locus113 | Forward | 14681 | 14840 | 0.995687 | Shared |
| **Bat** | ORF1b | locus79 | Forward | 14721 | 14880 | 0.995612 | Unique |
| **Human** | ORF1b | locus130 | Forward | 14761 | 14880 | 0.996073 | Unique |
| **Intermediate** | ORF1b | locus127 | Forward | 16961 | 17200 | 0.994855 | Unique |
| **Human** | ORF1b | locus138 | Forward | 17001 | 17200 | 0.998685 | Unique |
| **Bat** | ORF1b | locus87 | Forward | 17081 | 17360 | 0.998644 | Unique |
| **Human** | ORF1b | locus142 | Forward | 17481 | 17640 | 0.997599 | Unique |
| **Intermediate** | ORF1b | locus136 | Forward | 18201 | 18320 | 0.99607 | Unique |
| **Intermediate** | ORF1b | locus139 | Forward | 19321 | 19440 | 0.998695 | Unique |
| **Intermediate** | ORF1b | locus141 | Forward | 19361 | 19480 | 0.998613 | Shared |
| **Bat** | ORF1b | locus94 | Forward | 19361 | 19480 | 0.998668 | Shared |
| **Human** | ORF1b | locus150 | Forward | 19361 | 19520 | 0.998647 | Unique |
| **Human** | ORF1b | locus151 | Forward | 19401 | 19520 | 0.995753 | Commo |
| **Intermediate** | ORF1b | locus144 | Forward | 19401 | 19520 | 0.996525 | Commo |
| **Bat** | ORF1b | locus96 | Forward | 19401 | 19520 | 0.997026 | Commo |
| **Human** | ORF1b | locus152 | Forward | 20201 | 20360 | 0.998509 | Shared |
| **Intermediate** | ORF1b | locus145 | Forward | 20201 | 20360 | 0.99875 | Shared |
| **Intermediate** | ORF1b | locus147 | Forward | 20281 | 20520 | 0.996655 | Unique |
| **Bat** | ORF1b | locus101 | Forward | 20441 | 20600 | 0.998269 | Unique |
| **Human** | ORF1b | locus158 | Forward | 20801 | 20920 | 0.998271 | Unique |
| **Bat** | ORF1b | locus103 | Forward | 20801 | 21240 | 0.998766 | Unique |
| **Intermediate** | ORF1b | locus150 | Forward | 20921 | 21080 | 0.997263 | Unique |
| **Intermediate** | ORF1b | locus152 | Forward | 21081 | 21200 | 0.998404 | Unique |
| **Human** | ORF1b | locus160 | Forward | 21081 | 21240 | 0.998312 | Unique |
| **Intermediate** | S | locus158 | Forward | 21921 | 22040 | 0.996007 | Unique |
| **Bat** | S | locus108 | Forward | 22361 | 22600 | 0.995938 | Unique |
| **Human** | S | locus168 | Forward | 22481 | 22640 | 0.998582 | Unique |
| **Intermediate** | S | locus162 | Forward | 22521 | 22640 | 0.996589 | Unique |
| **Human** | S | locus176 | Forward | 22681 | 22800 | 0.998641 | Unique |

| | | | | | | | |
|---|---|---|---|---|---|---|---|
| **Intermediate** | S | locus168 | Forward | 22681 | 23000 | 0.99733 | Unique |
| **Human** | S | locus178 | Forward | 22721 | 22880 | 0.996028 | Unique |
| **Intermediate** | S | locus170 | Forward | 23001 | 23120 | 0.995534 | Unique |
| **Intermediate** | S | locus171 | Forward | 23641 | 23800 | 0.998515 | Unique |
| **Human** | S | locus180 | Forward | 23681 | 23800 | 0.998532 | Unique |
| **Bat** | S | locus112 | Forward | 23921 | 24040 | 0.998439 | Unique |
| **Bat** | S | locus114 | Forward | 23961 | 24080 | 0.998802 | Unique |
| **Bat** | ORF3a | locus118 | Forward | 25201 | 25440 | 0.997858 | Unique |
| **Human** | ORF3a | locus190 | Forward | 25401 | 25560 | 0.998332 | Unique |
| **Intermediate** | ORF3a | locus182 | Forward | 25641 | 25800 | 0.995939 | Unique |
| **Human** | ORF3a | locus192 | Forward | 25681 | 25800 | 0.997046 | Unique |
| **Bat** | ORF3a | locus121 | Forward | 25681 | 25840 | 0.998736 | Unique |
| **Intermediate** | ORF3a | locus184 | Forward | 25721 | 25840 | 0.998559 | Unique |
| **Human** | ORF3a | locus194 | Forward | 25761 | 25880 | 0.984922 | Shared |
| **Intermediate** | ORF3a | locus186 | Forward | 25761 | 25880 | 0.998631 | Shared |
| **Bat** | ORF3a | locus123 | Forward | 25761 | 25960 | 0.998832 | Unique |
| **Human** | ORF3a | locus196 | Forward | 25801 | 25920 | 0.996034 | Shared |
| **Intermediate** | ORF3a | locus188 | Forward | 25801 | 25920 | 0.998621 | Shared |
| **Human** | ORF3a | locus198 | Forward | 25841 | 26000 | 0.998386 | Shared |
| **Intermediate** | ORF3a | locus190 | Forward | 25841 | 26000 | 0.997869 | Shared |
| **Bat** | ORF3a | locus125 | Forward | 25881 | 26000 | 0.998946 | Unique |
| **Human** | ORF3a | locus200 | Forward | 25921 | 26040 | 0.998262 | Shared |
| **Intermediate** | ORF3a | locus192 | Forward | 25921 | 26040 | 0.995968 | Shared |
| **Bat** | ORF3a | locus127 | Forward | 25921 | 26120 | 0.998676 | Unique |
| **Human** | ORF3a | locus202 | Forward | 25961 | 26080 | 0.993777 | Unique |
| **Intermediate** | NA | locus194 | Forward | 25961 | 26200 | 0.992455 | Unique |
| **Human** | ORF3a | locus204 | Forward | 26001 | 26120 | 0.99577 | Unique |
| **Bat** | E | locus129 | Forward | 26121 | 26240 | 0.998296 | Unique |
| **Intermediate** | E | locus196 | Forward | 26121 | 26280 | 0.998641 | Unique |
| **Human** | E | locus210 | Forward | 26161 | 26280 | 0.998473 | Unique |
| **Bat** | E | locus131 | Forward | 26161 | 26360 | 0.998649 | Unique |
| **Human** | M | locus212 | Forward | 26201 | 26560 | 0.997338 | Unique |

| | | | | | | | |
|---|---|---|---|---|---|---|---|
| **Intermediate** | M | locus200 | Forward | 26441 | 26560 | 0.996856 | Unique |
| **Human** | M | locus214 | Forward | 26481 | 26600 | 0.998155 | Shared |
| **Intermediate** | M | locus202 | Forward | 26481 | 26600 | 0.998516 | Shared |
| **Human** | M | locus216 | Forward | 26521 | 26640 | 0.997636 | Unique |
| **Intermediate** | ORF7a | locus210 | Forward | 27361 | 27480 | 0.998621 | Unique |
| **Intermediate** | ORF8 | locus215 | Forward | 27841 | 28120 | 0.997663 | Unique |
| **Bat** | N | locus149 | Forward | 28161 | 28280 | 0.994912 | Unique |
| **Intermediate** | N | locus216 | Forward | 28161 | 28400 | 0.997703 | Unique |
| **Human** | N | locus232 | Forward | 28721 | 28840 | 0.995337 | Shared |
| **Intermediate** | N | locus219 | Forward | 28721 | 28840 | 0.994692 | Shared |
| **Intermediate** | N | locus220 | Forward | 28921 | 29080 | 0.998539 | Unique |
| **Human** | N | locus233 | Forward | 28961 | 29120 | 0.998651 | Unique |
| **Bat** | ORF10 | locus155 | Forward | 29481 | 29600 | 0.995642 | Unique |
